# Synthesis and characterization of tetraphenylammonium salts

**DOI:** 10.1038/s41467-022-30282-y

**Published:** 2022-05-09

**Authors:** Hikaru Fujita, Ozora Sasamoto, Shiori Kobayashi, Masanori Kitamura, Munetaka Kunishima

**Affiliations:** 1 Faculty of Pharmaceutical Sciences, Institute of Medical, Pharmaceutical, and Health Sciences, Kanazawa University, Kakuma-machi, Kanazawa, 920-1192 Japan; 2 Present Address: Faculty of Pharmaceutical Sciences, Matsuyama University, 4-2, Bunkyo-cho, Matsuyama, 790-8578 Japan

**Keywords:** Structure elucidation, Synthetic chemistry methodology

## Abstract

The phenyl (Ph) group is a representative substituent in the field of organic chemistry as benzene (the parent molecule) is of fundamental importance. Simple Ph-substituted compounds of common chemical elements are well known. However, extensive structural characterization of tetraphenylammonium (Ph_4_N^+^) salts has not been reported. Herein, the synthesis of Ph_4_N^+^ salts and their characterization data including the ^1^H and ^13^C nuclear magnetic resonance (NMR) spectra and the single-crystal X-ray structure have been presented. An intermolecular radical coupling reaction between an aryl radical and a triarylammoniumyl radical cation was conducted to synthesize the target moieties. The Ph_4_N^+^ salts described herein are the simplest tetraarylammonium (Ar_4_N^+^) salts known. The results reported herein can potentially help access the otherwise inaccessible non-bridged Ar_4_N^+^ salts, a new class of rigid and sterically hindered organic cations.

## Abstract (web summary)

Tetraphenylammonium (Ph_4_N^+^) salts remain synthetically inaccessible despite their simple chemical structure. Here, the authors have synthesized Ph_4_N^+^ salts and provided characterization including a single-crystal X-ray structure.

## Introduction

**Fig. 1:**
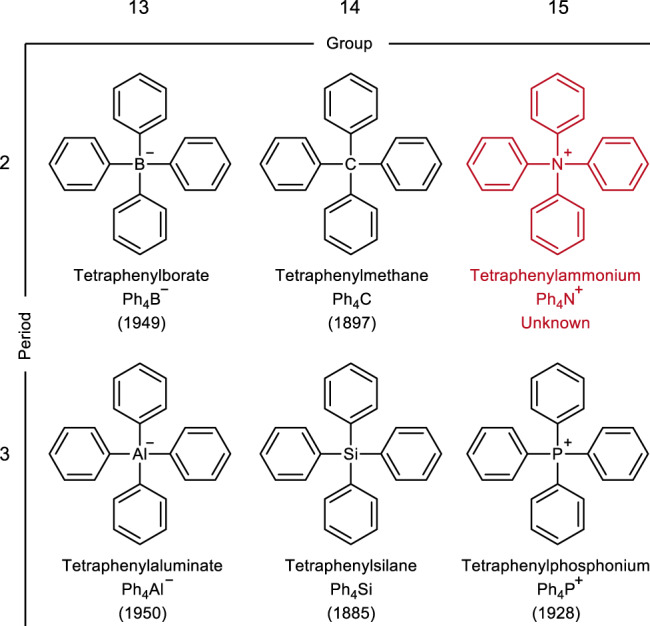
Structures of Ph_4_-substituted elements belonging to the groups 13–15, Ph_4_Z^0 ± 1^. The number in the parentheses indicates the year of synthesis (reported in the literature).

The elements in groups 13 (B and Al), 14 (C and Si), and 15 (N and P) typically form tetrahedral ions or molecules of the general formula R_4_Z^0 ± 1^, when four identical substituents (R_4_) are attached to the central element (Z). The charge on the atom Z depends on the group to which it belongs: −1, 0, and +1 for groups 13, 14, and 15, respectively. Compounds of type R_4_Z^0 ± 1^, bearing simple R substituents, are of special importance and considered benchmark compounds. The structural features, physical properties, and chemical reactivities of the other derivatives belonging to this class of compounds were compared with those of the benchmark compounds for a deeper understanding of the compound characteristics. Therefore, for a long time, organic chemists have focused on synthesizing such R_4_Z^0 ± 1^ compounds bearing simple R substituents. The compounds of the general formula R_4_Z^0 ± 1^ (R = Ph (Ph_4_B^−^, Ph_4_Al^−^, Ph_4_C, Ph_4_Si, and Ph_4_P^+^; Fig. 1))^1–6^ are known since long. Ph_4_Si was identified more than 130 years ago. The fundamental properties of these Ph_4_Z^0 ± 1^ compounds have been studied extensively over a long period of time. The results serve as references during the study of the corresponding tetraaryl-substituted compounds (Ar_4_Z^0 ± 1^). Quantum chemical calculations have been conducted for Ph_4_N^+^^7^. However, the experimental properties of this simple organic cation remain largely unknown. Though several researchers have reported the application prospects of the Ph_4_N^+^ salts (Supplementary Table 1), the synthetic route followed, and the compound characteristics have not been reported.

**Fig. 2:**
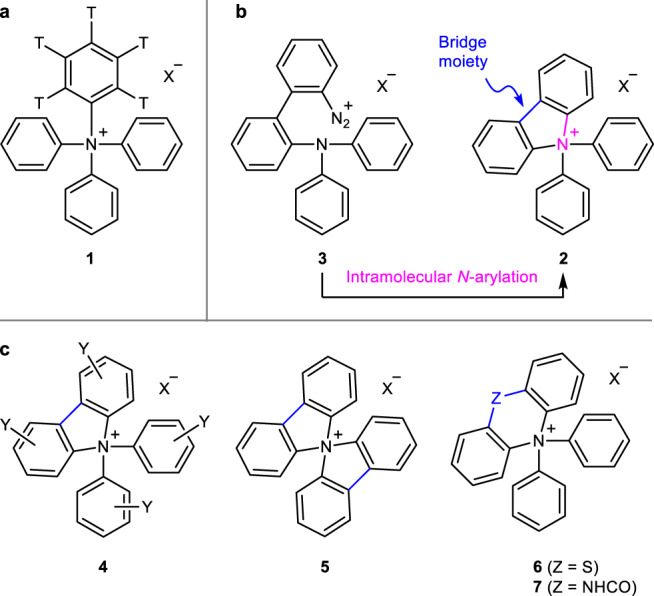
Ar_4_N^+^ salts reported in the literature. Counter anions are depicted in the general form X. **a** Pentatritiated Ph_4_N^+^ salts (**1**) reported without proper structural data. **b** Synthesis of (*N*,*N*-diphenyl)carbazolium salts (**2**) from precursor **3** following the process of intramolecular *N*-arylation. The bridge moiety indicated in blue must be removed to obtain the Ph_4_N^+^ salts. **c** Other bridged Ar_4_N^+^ salts such as (*N*,*N*-diphenyl)carbazolium salts (**4**) bearing various substituents Y on the aryl groups, spirobicarbazolium salts (**5**), sulfide-bridged Ar_4_N^+^ salts (**6**), and amide-bridged Ar_4_N^+^ salts (**7**) prepared following the intramolecular *N*-arylation strategy. The bridge moieties are indicated in blue.

It has been reported that the pentatritiated tetraphenylammonium salts (**1**; Fig. 2a) can be formed following the nuclear-chemical method via the tritium β-decay of hexatritiated benzene (C_6_T_6_)^8,9^. The isomorphic co-crystallization data and the radioactivity-based yields have been documented. However, the detailed synthetic procedure and data from experiments conducted for structure identification have not been reported. Bridged Ar_4_N^+^ salts are structurally similar to the Ph_4_N^+^ salts. In 1963, Nesmeyanov synthesized the (*N*,*N*-diphenyl)carbazolium salts (**2**; Fig. 2b) as the first examples of this class of compounds^10^. The key reaction step affording **2** from precursor **3** was the intramolecular *N*-arylation of triarylamine. The step proceeded via the decomposition of the spatially proximal aryldiazonium moiety. This process resulted in the formation of a 5-membered ring containing a nitrogen atom bearing four aromatic rings. Following the success of the method, a similar cyclization strategy was followed for the synthesis of various (*N*,*N*-diaryl)carbazolium salts^11–16^ (such as **4** and **5**; Fig. 2c), and sulfide- or amide-bridged Ar_4_N^+^ salts (**6**^17^ and **7**^18^, respectively). Recently, the quaternary ammonium structure of **2** was confirmed using the single-crystal X-ray diffraction technique^13,16^. However, none of these bridged Ar_4_N^+^ salts could be converted to Ph_4_N^+^ salts, as there is a dearth of efficient methods that can be used to remove the bridge moieties^17^.

Ar_4_N^+^ is a promising organic cation that can be used for developing surfactants, supporting electrolytes, phase-transfer catalysts, and anion-exchange membranes^13^. Also, it is potentially useful for industrial and biological studies. The wide application range of the cation can be attributed to the high chemical stability^14,15^ and unique rigid structure of the organic cation. Although the bridged Ar_4_N^+^ cations represented by **2** have been studied and characterized, non-bridged Ar_4_N^+^ cations have not been explored because of synthetic limitations.

Herein, we report a synthetic strategy for the preparation of Ph_4_N^+^ salts. To the best of our knowledge, this is the first report where the results of structural characteristics of the cation have been reported.

## Results and discussion

### Synthetic strategy

**Fig. 3:**
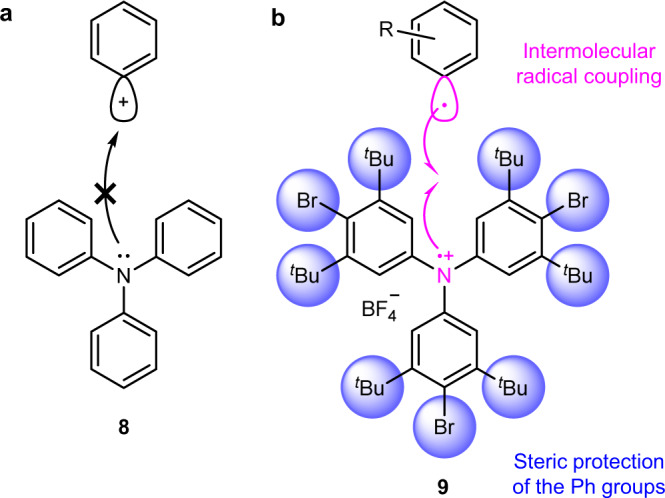
Synthetic strategy followed for the construction of a non-bridged Ar_4_N^+^ structure. **a** Direct *N*-phenylation of **8** with a Ph cation is difficult as the N atom is a weak nucleophile. **b** Intermolecular radical coupling reaction between an aryl radical and the triarylammoniumyl salt **9** bearing bulky protecting groups that exert steric hindrance and block the reactions at the Ph groups.

The direct *N*-phenylation of triphenylamine (**8**; Fig. 3a) using a Ph cation (or its synthetic equivalent) to form Ph_4_N^+^ is difficult because **8** is weakly nucleophilic (indicated by the low p*K*_aH_ value (−3.91)^19^ recorded during *N*-protonation). Ph_4_N^+^ salts could not be obtained by reacting **8** with a phenyldiazonium unit^10^. The *N*-phenylation of **8** using diphenyliodonium^20^ or the in-situ-generated benzyne^21^ unit was also unsuccessful. We designed the triarylammoniumyl salt (**9**; Fig. 3b) as a novel precursor that could be used for the synthesis of Ph_4_N^+^ to address the problem of low reactivity of **8**. In general, triarylamines can be oxidized to form the corresponding radical cations (referred to as triarylammoniumyls) that exhibit high reactivity. Triphenylammoniumyl easily dimerizes via the para positions of the Ph groups following the process of intermolecular radical coupling to afford tetraphenylbenzidine^22^. The results obtained from quantum chemical calculations revealed that the singly occupied molecular orbital of triphenylammoniumyl was spread over all the Ph rings and the central nitrogen atom^23^. Therefore, we expected that the intermolecular radical coupling reaction involving a triphenylammoniumyl unit and an aryl radical occurs via the nitrogen atom if the Ph group is hindered by steric protection. The tert-butyl and bromo groups were selected as the bulky protecting groups of **9** at the meta- and para-positions, respectively. These groups can exert a large extent of steric hindrance and can be removed at the later stages of the synthetic procedure.

### Synthesis

**Fig. 4:**
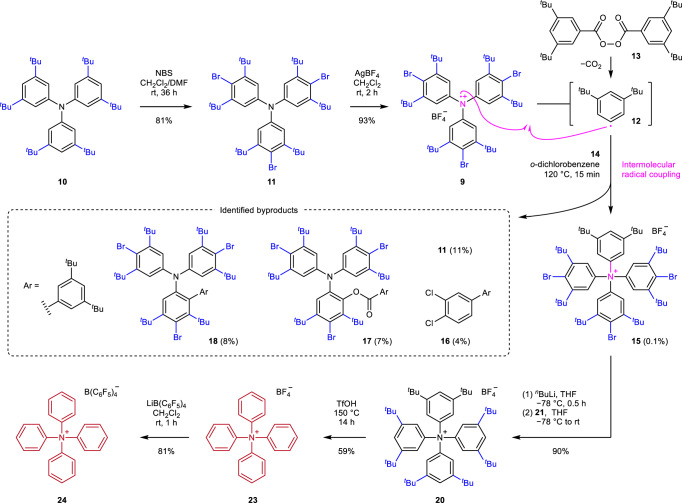
Synthetic scheme for the formation of the Ph_4_N^+^ salts 23 and 24. Triarylammoniumyl salt **9** was prepared from **10** over 2 steps. The intermolecular radical coupling reaction between **9** and aryl radical **12**, formed in situ following the thermolysis of the diacyl peroxide **13**, yielded the Ar_4_N^+^ salt (**15**) along with various byproducts (**11**, **16**, **17**, and **18**). Removal of the bromo and tert-butyl groups in **15** afforded **23**, whose counter anion was exchanged to obtain **24**.

The starting material used for the synthesis of the target was tris[(3,5-di-tert-butyl)phenyl]amine (**10**; Fig. 4), which was prepared over three steps starting from benzene: Friedel–Crafts reaction, dealkylative bromination, and palladium-catalyzed amination^24,25^. The para-brominated compound **11** was formed in 81% yield when **10** was treated with *N*-bromosuccinimide (NBS). The triarylamine **11** was then activated to form the triarylammoniumyl salt **9** following the one-electron oxidation process using AgBF_4_^26^. It was isolated as a monohydrate in 93% yield. Similar to other triarylammoniumyl salts^22^, **9** was a blue solid. The color could be attributed to the absorption over the visible region (λ_max_ = 797 nm in *o*-dichlorobenzene). Following this, we investigated the key intermolecular radical coupling reactions. Bis(3,5-di-tert-butyl)benzoyl peroxide (**13**) was used as the starting material for the in situ generation of the (3,5-di-tert-butyl)phenyl radical (**12**). The formation of the radical proceeded via the process of O–O homolysis, which was followed by the process of decarboxylation^27^. A mixture of **9** and **13** was heated to 120 °C in *o*-dichlorobenzene in the presence of (2,6-di-tert-butyl)pyridine (**14**; used as a base) until the characteristic blue color of **9** disappeared. The reaction conditions were selected from the results of the screening experiments (vide infra). The desired Ar_4_N^+^ salt **15** was successfully formed in a low yield (0.1%), which was then isolated using the normal-phase ion-pair chromatography technique^28^. Under these conditions, 4 g of **9** could be converted to 5 mg of **15**. The byproducts formed during the reaction were triarylamine **11** (11%), solvent adduct **16** (4% based on **13**), and sterically congested triarylamines **17** (7%) and **18** (8%) possessing ortho-[(3,5-di-tert-butyl)benzoyl]oxy and ortho-(3,5-di-tert-butyl)phenyl groups, respectively. The structures of **17** and **18** were determined using the single-crystal X-ray diffraction technique (Supplementary Tables 2 and 3, respectively). The formation of **17** and **18** indicated that the extent of steric protection provided by the meta-tert-butyl groups in **9** was not sufficient to efficiently inhibit the occurrence of the ortho-substitution reactions at the Ph rings. Supplementary Table 4 shows the process of reaction condition screening for the intermolecular radical coupling reaction conducted on a small scale using **9** (80–100 mg). When the reaction was conducted in *o*-dichlorobenzene in the presence of **14** (entry 1), the yield of the desired ammonium salt **15** was 0.12% (determined by ^1^H NMR spectroscopic analysis). Although the same yield (0.12%, entry 2) of **15** was obtained when the reaction was carried out in the absence of **14**, it was difficult to purify the product under these conditions as various byproducts were also formed during the process. The use of other solvents in combination with **14** afforded lower (entries 3–10) or undetectable (entries 11–14) yields of **15**, and a complex mixture of compounds which could not be purified or analyzed (entries 16–21). Thus, the reaction conditions presented in entry 1 were used to synthesize **15** from **9** (4 g). We also attempted the intermolecular radical coupling reaction involving **13** and tris[(3,5-di-tert-butyl)phenyl]ammoniumyl BF_4_^−^ (**19**; devoid of the *p*-bromo groups). The latter was prepared following the one-electron oxidation of **10**. However, the desired product tetrakis[(3,5-di-tert-butyl)phenyl]ammonium BF_4_^−^ (**20**) could not be isolated as the reaction yielded a complex mixture. Therefore, the removal of all the bromo groups in **15** was carried out following the process of bromine–lithium exchange using ^*n*^BuLi at −78 °C. The resulting product was protonated with (2,6-di-tert-butyl)pyridinium BF_4_^−^ salt (**21**), affording **20** in 90% yield. Since we selected diacyl peroxide **13** as the precursor of aryl radical **12** to introduce the (3,5-di-tert-butyl)phenyl group in **9**, the ammonium nitrogen of **20** was connected to four identical aryl groups. The counter anion of **20** was exchanged to prepare the corresponding B(C_6_F_5_)_4_^−^ salt (**22**), the structure of which was confirmed using the single-crystal X-ray diffraction technique (Supplementary Table 5). The final step toward the formation of Ph_4_N^+^ involved the dealkylation of the tert-butyl groups present on the aromatic rings of **20**. All the eight tert-butyl groups could be successfully removed when **20** was heated at 150 °C over a period of 14 h in a solvent amount of trifluoromethanesulfonic acid (TfOH)^29^. The reaction afforded Ph_4_N^+^ BF_4_^−^ (**23**) in 59% yield. This result indicates the high stability of Ph_4_N^+^ under extremely harsh acidic conditions. Following the process of counter anion exchange, the BF_4_^−^ salt (**23**) was converted to the B(C_6_F_5_)_4_^−^ salt (**24**; yield: 81%; white solid). The purity of the counter anion was confirmed using the ^19^F NMR spectroscopy technique.

### ^1^H and ^13^C NMR spectra

**Fig. 5:**
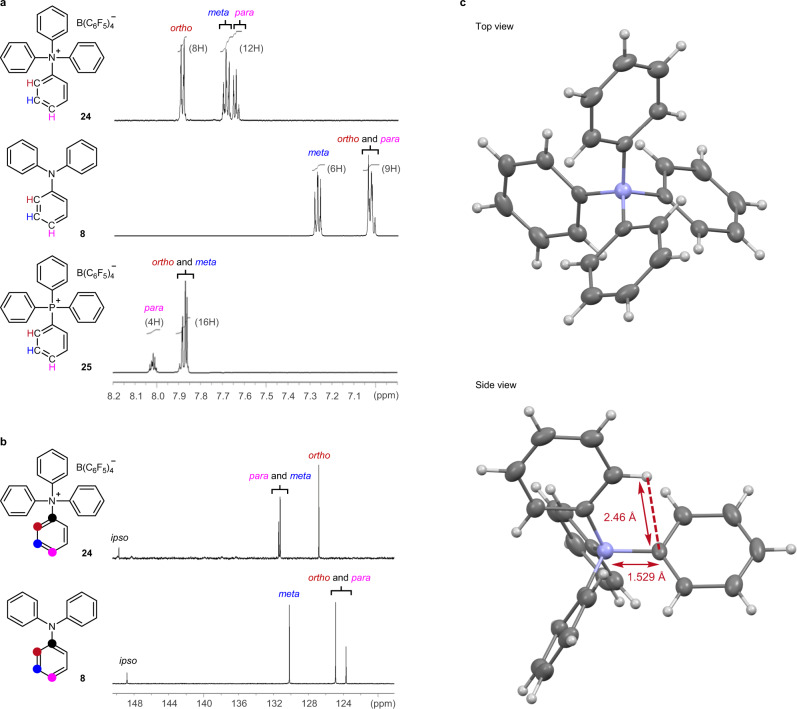
Structural characterization of Ph_4_N^+^. **a** Comparison of the ^1^H NMR spectral profiles (600 MHz, (CD_3_)_2_CO) recorded for **24**, **8**, and **25**. The number of protons is presented in the parentheses. **b** Comparison of the ^13^C NMR spectral profiles (150 MHz, (CD_3_)_2_CO) recorded for **24** and **8**. **c** Single-crystal X-ray structure of **24**. All ellipsoids are contoured at the 50% probability level. The counter anion was omitted for clarity.

The ^1^H NMR signals corresponding to **24** (spectra recorded in (CD_3_)_2_CO) appeared at approximately 7.89, 7.69, and 7.65 ppm. The signal corresponded to the ortho, meta, and para-protons present in the Ph ring, respectively. The ^13^C NMR signals corresponding to **24** (spectra recorded in (CD_3_)_2_CO) appeared at 149.7 (ipso), in the range of 131.4–131.2 (para and meta), and at 126.8 (ortho) ppm. The signals were assigned to the corresponding carbon atoms using the HMQC technique. The ^1^H and ^13^C NMR signals corresponding to **24** appeared downfield compared to the signals corresponding to **8** (Fig. 5a, b). The downfield shift can be attributed to the strong inductive effect exerted by the ammonium nitrogen in **24** and the loss of the resonance effect of the lone pair of electrons on nitrogen present in **8** following *N-*quaternization. The signals corresponding to the meta and para protons present in **24** (at ~7.69 and ~7.65 ppm, respectively) appeared at up-field compared to the signals corresponding to Ph_4_P^+^ B(C_6_F_5_)_4_^−^^30^ (**25**; ~7.88 and ~8.02 ppm for meta and para protons, respectively). The up-field shift may be attributed to the strong anisotropic effects observed in Ph_4_N^+^. The generation of the strong anisotropic effects can be attributed to the fact that Ph_4_N^+^ is smaller than Ph_4_P^+^. Similarly, we observed that the signals corresponding to the meta and para protons in Ph_4_C ((CD_3_)_2_CO; at ~7.29 and ~7.21 ppm, respectively) appeared up-field compared to the signals corresponding to Ph_4_Si (meta and para protons appear at ~7.44 and ~7.48 ppm, respectively; Supplementary Fig. 1)^31^.

### Single-crystal X-ray structure analysis

The single-crystal X-ray diffraction technique was used to analyze the structure of **24**. Analysis of the results proved the quaternary ammonium structure of **24** (Fig. 5c, top view). The counter anion B(C_6_F_5_)_4_^−^ was omitted for clarity (Supplementary Table 6). The Ph_4_N^+^ structure exhibited *S*_4_-like symmetry and not *D*_2*d*_-like symmetry. The result agreed well with the theoretically predicted result^32^. The N–C(sp^2^) bond length in **24** (present between the Ph_4_N^+^ nitrogen unit and the sp^2^ carbon atom) in the Ph group was 1.529 ± 0.003 Å (Fig. 5c, side view). This bond is longer than the N–C(sp^2^) bonds in (CH_3_)_3_PhN^+^, (CH_3_)_2_Ph_2_N^+^, (CH_3_)Ph_3_N^+^, and *N*,*N*-diphenylcarbazolium, which were 1.50^33^, 1.51^21^, 1.52^21^, and 1.51–1.52^16^ Å, respectively (data from Cambridge Crystallographic Data Center (CCDC) deposition numbers of 291166, 1433867, 1433868, and 1890475, respectively). The long N–C(sp^2^) bond in Ph_4_N^+^ indicates the presence of an unusually hindered environment around the ammonium nitrogen atom. The lengths of the bonds formed between the central atom and the sp^2^ carbons in Ph_4_C, Ph_4_B^−^, Ph_4_P^+^, Ph_4_Si, and Ph_4_Al^−^ were 1.56^34^, 1.64–1.66^35^, 1.79^36^, 1.88^37^, and 2.00–2.03^38^ Å, respectively. Thus, Ph_4_N^+^ is characterized by the most sterically congested environment among these Ph_4_Z^0 ± 1^, reflecting the difficulty faced during synthesis. The C–N bond length of Ph_4_N^+^ (1.529 Å) was ca. 8% longer than that of **8** (1.419 Å)^39^, whereas the C–P bond length of Ph_4_P^+^ (1.792 Å) was ca. 2% shorter than that of Ph_3_P (1.828 Å)^39^. The shortest distance between the ortho-hydrogen in the Ph unit and the ipso carbon in the adjacent Ph group in Ph_4_N^+^ was 2.46 Å (Fig. 5c, side view). The Van der Waals radii for hydrogen (1.00 Å) and carbon (1.77 Å) indicate that steric repulsion is generated^40^. The corresponding H–C distances in Ph_4_C, Ph_4_B^−^, and Ph_4_P^+^ were 2.54^34^, 2.59^35^, and 2.76^36^ Å, respectively, indicating that the extent of steric repulsion observed in these cases was lower than that observed in Ph_4_N^+^. The N–C(sp^2^) bond length in octa-tert-butylated Ph_4_N^+^ salt **22** was in the range of 1.53–1.54 Å and the H–C distance was 2.48 Å. These values are slightly higher than the corresponding values recorded for **24**. This suggested the generation of steric and electronic effects in the presence of the meta-tert-butyl groups in **22** present in the ammonium structure.

### Initial evaluation of alkaline stability

Alkaline stability of Ph_4_N^+^ salt **23** was compared with that of *N*,*N*-diphenylcarbazolium PF_6_^−^ salt **2-PF**_**6**_ by heating their solutions in CD_3_OD/D_2_O (3:1, containing 5 M KOH) to 80 °C. The ^1^H NMR monitoring revealed that **23** was gradually decomposed over 5 days (Supplementary Fig. 2) whereas **2-PF**_**6**_ almost completely disappeared in 24 h (Supplementary Fig. 4). Therefore, **23** exhibited higher alkaline stability than **2-PF**_**6**_. The rapid decrease of the signal assigned to the ortho-protons of **23** suggested that H/D exchange occurred during this experiment^14^. Octa-tert-butylated Ph_4_N^+^ salt **20** exhibited excellent alkaline stability. No apparent spectral change of **20** was observed under the same conditions for 30 days (Supplementary Fig. 5). The alkaline stability of **20** can be compared with that of sterically hindered imidazolium salts^41^, which were also inert under similar alkaline conditions.

In conclusion, we have synthesized Ph_4_N^+^ salts which are structurally simple non-bridged Ar_4_N^+^ salts. The ^1^H and ^13^C NMR spectra of the Ph_4_N^+^ salts have been presented. The single-crystal X-ray structure analysis revealed the long N–C bond present in this organic cation. The Ph_4_N^+^ salts described herein are essential benchmarks for non-bridged Ar_4_N^+^ salts. We have confirmed that the non-bridged Ar_4_N^+^ salts we prepared are highly stable under basic conditions. Our findings will encourage further synthetic study of this new class of organic cations which are attractive for diverse applications. The concept of the radical coupling reaction (between a triarylammoniumyl and an aryl radical) reported herein can potentially be applied to the synthesis of various non-bridged Ar_4_N^+^ salts. Further studies need to be conducted to achieve higher product yields.

## Methods

### Synthesis of tris[(4-bromo-3,5-di-tert-butyl)phenyl]amine (11)

Triarylamine **10** (5.0 g, 8.6 mmol) and NBS (5.0 g, 28 mmol) were added to a mixture of CH_2_Cl_2_/DMF (1:1, 42.0 ml) at room temperature. After 68 h, the reaction mixture was quenched with saturated aqueous Na_2_S_2_O_3_ (30 ml) and then extracted with CHCl_3_ (200 ml). The organic layer was washed with brine, dried (Na_2_SO_4_), and filtrated. The filtrate was concentrated under reduced pressure. The crude product was recrystallized from CHCl_3_/MeOH to afford a white solid (5.7 g, 81%). Mp: 246–247 °C; TLC (hexane): RF = 0.50; ^1^H NMR (600 MHz, CDCl_3_): *δ* 7.11 (s, 6H), 1.48 (s, 54H); ^13^C NMR (150 MHz, CDCl_3_): *δ* 150.0, 144.9, 121.8, 117.8, 38.4, 31.0; HRMS (ESI-TOF, *m*/*z*): [M]^+^ calcd for C_42_H_60_Br_3_N, 815.2276; found, 815.2278.

### Synthesis of tris[(4-bromo-3,5-di-tert-butyl)phenyl]ammoniumyl tetrafluoroborate monohydrate (9)

A mixture of triarylamine **11** (1.000 g, 1.22 mmol) and AgBF_4_ (260 mg, 1.34 mmol) in CH_2_Cl_2_ (12.5 ml) was stirred for 2 h at room temperature. The reaction mixture was then filtrated. The filtrate was concentrated under reduced pressure. The residue was washed with hexane to afford a blue solid (1,044 mg, 93%). Mp: 166 °C (decomp.); UV/vis: λ_max_ 797 nm (in *o*-dichlorobenzene); HRMS (ESI-TOF, *m*/*z*): [M − BF_4_ − H_2_O]^+^ calcd for C_42_H_60_Br_3_N, 815.2276; found, 815.2262; analysis (% calcd, % found for C_42_H_62_BBr_3_F_4_NO): C (54.63, 54.37), H (6.77, 6.48), N (1.52, 1.58).

### Synthesis of tris[(4-bromo-3,5-di-tert-butyl)phenyl][(3,5-di-tert-butyl)phenyl]ammonium tetrafluoroborate (15)

A mixture of triarylammoniumyl salt **9** (1.000 g, 1.08 mmol), diacyl peroxide **13** (1.550 g, 3.321 mmol), and pyridine **14** (250 μl, 1.11 mmol) in *o*-dichlorobenzene (8.5 ml) was heated to 120 °C for 15 min. Then, the reaction mixture was cooled to room temperature and filtered (CHCl_3,_ 20 ml). This reaction procedure was repeated another three times to obtain a set of four filtrate solutions. The combined filtrate solution was passed through NaBF_4_-treated silica gel to afford Fraction A (CHCl_3_ as an eluent, 350 ml) followed by Fraction B (CHCl_3_/MeOH = 90:10 as an eluent, 400 ml). Concentration of Fractions A and B under reduced pressure afforded Residues A and B, respectively. Residue B was purified by ion-pair column chromatography (NaBF_4_-treated silica gel, CHCl_3_/MeOH = 95:5) followed by preparative thin layer ion-pair chromatography [NaBF_4_-treated silica gel TLC plate, 0.50 mm thick, three times (CHCl_3_/MeOH = 90:10, hexane/EtOAc = 30:70, and hexane/EtOAc = 40:60 for the first, the second, and the third chromatography, respectively)] to afford **15** as an off-white solid (4.97 mg, 0.11%). TLC (NaBF_4_-treated silica gel, CHCl_3_/MeOH = 90:10): RF = 0.40; ^1^H NMR (600 MHz, CDCl_3_): *δ* 7.60 (t, *J* = 1.5 Hz, 1H), 7.47 (s, 6H), 7.23 (d, *J* = 1.5 Hz, 2H), 1.48 (s, 54H), 1.28 (s, 18H); ^1^H NMR (600 MHz, CD_3_OD): *δ* 7.78 (t, *J* = 1.4 Hz, 1H), 7.47 (s, 6H), 7.28 (d, *J* = 1.4 Hz, 2H), 1.48 (s, 54H), 1.28 (s, 18H); ^13^C NMR (150 MHz, CDCl_3_): *δ* 154.6, 152.9, 147.9, 146.3, 127.5, 124.8, 122.7, 119.3, 39.3, 35.9, 31.3, 30.6; ^19^F NMR (376 MHz, CDCl_3_): *δ* −154.4; HRMS (ESI-TOF, *m*/*z*): [M − BF_4_]^+^ calcd for C_56_H_81_Br_3_N, 1004.3919; found, 1004.3898.

Partial purification of Residue A by column chromatography (silica gel, hexane/CHCl_3_ = 100:0 to 90:10 to 80:20) afforded Fraction A1 (containing compounds **11**, **16**, and **18**) and Fraction A2 (containing compound **17**). Further purification of Fraction A1 using recycling preparative HPLC afforded **11** (404 mg, 11%), **16** (348 mg, 3.9% based on **13**), and **18** (366 mg, 8.4%). Further purification of Fraction A2 (silica gel, hexane/CHCl_3_ = 90:10) followed by recrystallization (hexane/CHCl_3_) afforded **17** (312 mg, 6.9%).

### Synthesis of tris[(3,5-di-tert-butyl)phenyl]ammoniumyl tetrafluoroborate dihydrate (19)

A mixture of triarylamine **10** (150 mg, 0.26 mmol) and AgBF_4_ (60 mg, 0.31 mmol) in CH_2_Cl_2_ (2.5 ml) was stirred for 1.5 h at room temperature. The reaction mixture was then filtrated. The filtrate was concentrated under reduced pressure. The residue was washed with hexane to afford a blue solid (155 mg, 85%). Mp: 170 °C (decomp.); UV/vis: λ_max_ 353 nm (in *o*-dichlorobenzene); HRMS (ESI-TOF, *m*/*z*): [M − BF_4_ − 2H_2_O]^+^ calcd for C_42_H_63_N, 581.4961; found, 581.4973; analysis (% calcd, % found for C_42_H_67_BF_4_NO_2_): C (71.57, 71.14), H (9.58, 9.18), N (1.99, 1.91).

### Synthesis of tetrakis[(3,5-di-tert-butyl)phenyl]ammonium tetrafluoroborate (20)

^*n*^BuLi solution in hexane (1.56 M, 110 μl, 172 μmol) was added to a solution of Ar_4_N^+^ salt **15** (5.8 mg, 5.3 μmol) in THF (1.7 ml) at −78 °C. After 25 min, the reaction mixture was quenched by adding **21** (47.0 mg, 168 μmol) under N_2_ flow. After 5 min, the reaction mixture was allowed to warm to room temperature and concentrated under reduced pressure. The residue was suspended in CH_2_Cl_2_ (10 ml). The resulting suspension was filtered, and the filtrate was concentrated under reduced pressure. The residue was purified by ion-pair column chromatography (NaBF_4_-treated silica gel, CHCl_3_/MeOH = 100:0 to 90:10) followed by preparative thin layer ion-pair chromatography (NaBF_4_-treated silica gel TLC plate, 0.50 mm thick, CHCl_3_/MeOH = 90:10) to afford an off-white solid (4.1 mg, 90%). TLC (NaBF_4_-treated silica gel, CHCl_3_/MeOH = 90:10): RF = 0.35; ^1^H NMR (600 MHz, CDCl_3_): *δ* 7.53–7.51 (m, 4H), 7.33–7.30 (m, 8H), 1.25 (s, 72H); ^1^H NMR (600 MHz, CD_3_OD): *δ* 7.67 (t, *J* = 1.6 Hz, 4H), 7.42 (d, *J* = 1.6 Hz, 8H), 1.26 (s, 72H); ^13^C NMR (100 MHz, CDCl_3_): *δ* 153.9, 148.5, 124.2, 119.7, 35.7, 31.3; ^19^F NMR (565 MHz, CDCl_3_): *δ* −154.6; HRMS (ESI-TOF, *m*/*z*): [M − BF_4_]^+^ calcd for C_56_H_84_N, 770.6604; found, 770.6587.

### Synthesis of tetrakis[(3,5-di-tert-butyl)phenyl]ammonium tetrakis(pentafluorophenyl)borate (22)

A solution of Ar_4_N^+^ salt **20** (1.47 mg, 1.7 μmol) and LiB(C_6_F_5_)_4_–Et_2_O (4.2 mg, 5.6 μmol as a 1:1 complex) in CH_2_Cl_2_ (500 μl) was stirred for 25 min at room temperature. The reaction mixture was directly purified by column chromatography (diol-functionalized silica gel, CH_2_Cl_2_) to afford an off-white solid (2.19 mg, 88%). TLC (CHCl_3_/acetone = 80:20): RF = 0.17; ^1^H NMR (600 MHz, CDCl_3_): *δ* 7.52 (t, *J* = 1.4 Hz, 4H), 7.29 (d, *J* = 1.4 Hz, 8H), 1.22 (s, 72H); ^13^C NMR (150 MHz, CDCl_3_): *δ* 153.9, 148.4, 124.2, 119.8, 35.6, 31.2; ^19^F NMR (565 MHz, CDCl_3_): *δ* −132.5, −163.5, −167.0; HRMS (ESI-TOF, *m*/*z*): [M − B(C_6_F_5_)_4_]^+^ calcd for C_56_H_84_N, 770.6604; found, 770.6589. Suitable crystals for X-ray analysis were obtained by recrystallization (acetone/MeOH).

### Synthesis of tetraphenylammonium tetrafluoroborate (23)

A solution of Ar_4_N^+^ salt **20** (3.92 mg, 4.6 μmol) in TfOH (2.0 ml) was heated to 150 °C for 14 h, and then cooled to room temperature. The reaction mixture was concentrated under reduced pressure, and the residue was dissolved in H_2_O (6 ml). The solution was neutralized with aqueous NaOH (0.5 M), saturated with NaBF_4_ (~3 g), and extracted with CHCl_3_ (10 × 5 ml). The organic layer was dried (Na_2_SO_4_) and filtrated. The filtrate was concentrated under reduced pressure. Preparative thin layer ion-pair chromatography (NaBF_4_-treated silica gel TLC plate, 0.5 mm thick, CHCl_3_/MeOH = 90:10) afforded an off-white solid (1.10 mg, 59%). TLC (NaBF_4_-treated silica gel, CHCl_3_/MeOH = 95:5): RF = 0.45; ^1^H NMR (600 MHz, CD_3_OD): *δ* 7.76–7.70 (m, 8H), 7.65–7.55 (m, 12H); ^13^C NMR (150 MHz, CD_3_OD): *δ* 150.0, 131.6, 131.4, 127.0; ^19^F NMR (565 MHz, CDCl_3_): *δ* −153.8; IR: 2958, 2920, 2851, 1733, 1477, 1452, 1259, 1091, 1043, 1022, 996, 799, 757, 692, 631, 491, 431, 412 cm^−1^; HRMS (ESI-TOF, *m*/*z*): [M − BF_4_]^+^ calcd for C_24_H_20_N, 322.1596; found, 322.1602.

### Synthesis of tetraphenylammonium tetrakis(pentafluorophenyl)borate (24)

A solution of Ar_4_N^+^ salt **23** (1.10 mg, 2.7 μmol) and LiB(C_6_F_5_)_4_–Et_2_O (4.1 mg, 5.4 μmol as a 1:1 complex) in CH_2_Cl_2_ (500 μl) was stirred for 1 h at room temperature. The reaction mixture was directly purified by column chromatography (diol-functionalized silica gel, CH_2_Cl_2_) to afford a white solid (2.17 mg, 81%). TLC (diol-functionalized silica gel, CHCl_3_): RF = 0.31; ^1^H NMR [600 MHz, (CD_3_)_2_CO]: *δ* 7.91–7.87 (m, 8H), 7.72–7.67 (m, 8H), 7.67–7.62 (m, 4H); ^13^C NMR [150 MHz, (CD_3_)_2_CO]: *δ* 149.7, 131.4, 131.2, 126.8; ^19^F NMR (565 MHz, CDCl_3_): *δ* −132.4, −163.1, −166.8; IR: 2955, 2917, 2850, 1712, 1643, 1513, 1462, 1275, 1086, 979, 775, 756, 746, 692, 661, 418 cm^−1^; HRMS (ESI-TOF, *m*/*z*): [M − B(C_6_F_5_)_4_]^+^ calcd for C_24_H_20_N, 332.1596; found, 332.1606. Suitable crystals for X-ray analysis were obtained by recrystallization (acetone/MeOH).

## Supplementary information

Supplementary Information

## Data Availability

The data supporting the findings of this study are available within the article and its Supplementary Information file. CCDC 2117248, 2117243, 2117249, and 2117251 contain the supplementary crystallographic data for compounds **17**, **18**, **22**, and **24**, respectively. These data can be obtained free of charge from The Cambridge Crystallographic Data Centre via www.ccdc.cam.ac.uk/structures.
