# Supplementary material for: Synthesis and characterization of tetraphenylammonium salts
Source: Nat Commun. 2022 May 9;13:2537. doi: 10.1038/s41467-022-30282-y (PMC9085870; doi:10.1038/s41467-022-30282-y)
Supplement: Supplementary file 1 — Supplementary Information [file 41467_2022_30282_MOESM1_ESM.pdf]

Supplementary Information

Synthesis and characterization of tetraphenylammonium salts

Hikaru Fujita*, Ozora Sasamoto, Shiori Kobayashi, Masanori Kitamura† and Munetaka Kunishima*

Faculty of Pharmaceutical Sciences, Institute of Medical, Pharmaceutical, and Health Sciences, Kanazawa University, Kakuma-machi, Kanazawa 920-1192, Japan
†Present address: Faculty of Pharmaceutical Sciences, Matsuyama University, 4-2, Bunkyo-cho, Matsuyama 790-8578, Japan

E-mail for Correspondence
Hikaru Fujita: hfujita@staff.kanazawa-u.ac.jp
Munetaka Kunishima: kunisima@p.kanazawa-u.ac.jp

Table of contents

1. Supplementary Tables 1–6 2

2. Supplementary Figures 1–5 9

3. Supplementary Methods

3.1. General information 14

3.2. Experimental procedure and characterization data 15

3.3. Experimental procedure for Supplementary Table 4 18

3.4. Experimental procedure for the attempted synthesis of 20 from 19. 61

3.5. Experimental procedure for the initial evaluation of alkaline stability. 62

4. Spectroscopic data

4.1. Absorption spectra 63

4.2. IR spectra 67

4.3. NMR spectra 70

5. Supplementary References 108

1. Supplementary Tables 1–6

Supplementary Table 1 | Summary of the results reported in the literature presenting the applications and uses of Ph_4N^+ salts.

Ref. number	Ph_4N^+ salts used in the references
Ref. 1	This paper presented the solubilities of Ph_4N^+ ClO_4^- in H_2O, EtOH, and benzene in Table 1. The authors reported that the Ph_4N^+ ClO_4^- salt was prepared by treating an aqueous solution of Ph_4N^+ Br^- or Ph_4N^+ I^- (commercially available) with perchloric acid. The solubility decreased when washed with cold water and in the absence of alcohol. The data on structural characterization for the Ph_4N^+ salts were not presented.
Ref. 2	The authors used Ph_4N^+ Cl^- as a supporting electrolyte while studying the 9,9'-azophenanthrene radical anions. The synthetic route for the preparation and the data on the structural characterization of the Ph_4N^+ salt were not presented.
Ref. 3	The authors used Ph_4N^+ Br^- as an additive to induce the interaction of the units with the phospholipid bilayers. The synthetic route for the preparation and the data on the structural characterization of the Ph_4N^+ salt were not presented.
Ref. 4	The authors studied the basic properties of the cobalt–iodine–ligand catalyst system following a methanol homologation reaction. The effect of Ph_4N^+ I^- as an additive was mentioned on page 318 of this paper. The synthetic route for the preparation and the data on the structural characterization of the Ph_4N^+ salt were not presented.
Ref. 5	In this short paper, the author mentioned the effect of the Ph_4N^+ ion on the phospholipid bilayers. The synthetic route for the preparation and the data on the structural characterization of the Ph_4N^+ ion were not presented.
Ref. 6	This paper reported the use of Ph_4N^+ Br^-, which was obtained from Aldrich as an inhibitor of the organic cation-H^+ exchange process. The data on the structural characterization of the Ph_4N^+ salt were not presented.
Ref. 7	The authors mentioned the use of Ph_4N^+ Cl^- as an ion-pair extracting agent for perruthenate on page 676. The synthetic route for the preparation and the data on the structural characterization of the Ph_4N^+ salt were not presented.
Ref. 8	The authors mentioned the use of Ph_4N^+ Cl^- as an ion-pair extracting agent for bismuthate on page 202. The synthetic route for the preparation and the data on the structural characterization of the Ph_4N^+ salt were not presented.
Ref. 9	This paper described a new voltammetric method for determining titanium(IV) using a carbon paste electrode modified with cetyltrimethylammonium bromide. The use of Ph_4N^+ Cl^- instead of cetyltrimethylammonium bromide has been mentioned on page 1919. The synthetic route for the preparation and the data on the structural characterization of the Ph_4N^+ salt were not presented.
Ref. 10	This paper described the voltammetric behavior of thiopurines at a silver electrode modified with cetyltrimethylammonium bromide. The use of Ph_4N^+ Br^- instead of cetyltrimethylammonium bromide has been mentioned on page 880 of this paper. The synthetic route for the preparation and the data on the structural characterization of the Ph_4N^+ salt were not presented.
Ref. 11	This paper reported Pd-catalyzed oxidative carbonylation of bisphenol A for the production of polycarbonate. Ph_4N^+ Br^- was used as an additive in this reaction (Table 1). The synthetic route for the preparation and the data on the structural characterization of the Ph_4N^+ salt were not presented.
Ref. 12	This paper reported the effects of Ph_4N^+ cation on the equilibrium of silicate oligomers in aqueous and non-aqueous alkaline silicate solutions. Ph_4N^+-containing silicate solutions were prepared by dissolving SiO_2 in aqueous Ph_4N^+ HO^-, which was obtained from Ph_4N^+ I^- obtained from Fluka Co (Basel and Lausanne, Switzerland). The data on the structural characterization of the Ph_4N^+ salt were not presented.
Ref. 13	This paper reported the effects of the Ph_4N^+ cation on the equilibrium distribution of aluminosilicate oligomers in aqueous and methanolic alkaline aluminosilicate solutions. Ph_4N^+-containing aluminate solutions were prepared by dissolving the powdered aluminum samples in Ph_4N^+ HO^- solutions. The author obtained Ph_4N^+ HO^- from Ph_4N^+ Br^- following the process of anion exchange. The data on the structural characterization of the Ph_4N^+ salt were not presented.
Ref. 14	This paper reported the effects of the Ph_4N^+ cation on the distribution of aluminophosphate species in aqueous and methanolic solutions. The authors used Ph_4N^+ Cl^-, purchased from Merck, as the source of the Ph_4N^+ cation. The data on the structural characterization of the Ph_4N^+ salt were not presented

Supplementary Table 2 | Summary of the crystal data for compound 17.

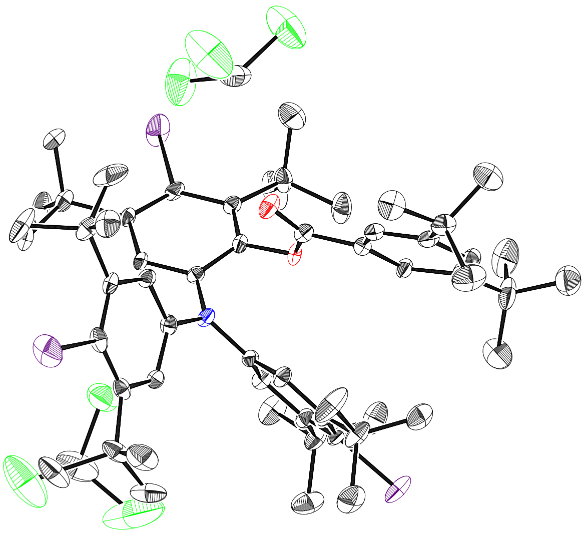

Thermal ellipsoids drawn at 50% probability. Hydrogen atoms and disorders are omitted for clarity.

CCDC registry	2117248
Formula	$C_{59}H_{82}Br_3Cl_6NO_2$
Formula Weight (g/mol)	1289.68
Crystal Dimensions (mm)	0.35×0.26×0.16
Crystal System	triclinic
Space Group	P −1
Temperature (K)	118(2)
a (Å)	10.8112(5)
b (Å)	13.8908(8)
c (Å)	22.4201(10)
α (°)	91.697(7)
β (°)	97.842(7)
γ (°)	105.920(8)
V ($Å^3$)	3199.6(3)
Number of reflections to determine final unit cell	19997
Min and Max 2θ for cell determination (°)	3.46, 54.94
Z	2
F(000)	1332
λ (Å, Mo Kα)	0.71075
μ (mm^{-1})	2.180
Max 2θ for data collection (°)	54.94
Measured fraction of data	0.993
Number of reflections measured	31459
Unique reflections measured	14517
R_{merge}	8.70%
Number of parameters in least-squares	699
R_1	0.0672
wR_2	0.1353
R_1 (all data)	0.1331
wR_2 (all data)	0.1611

Supplementary Table 3 | Summary of the crystal data for compound 18.

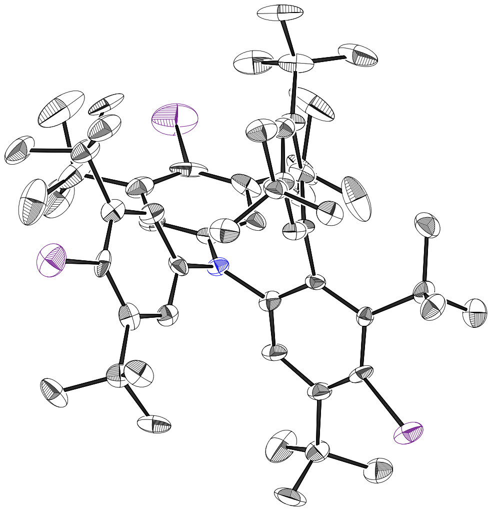

Thermal ellipsoids drawn at 50% probability. Hydrogen atoms are omitted for clarity.

CCDC registry	2117243
Formula	$C_{56}H_{80}Br_3N$
Formula Weight (g/mol)	1006.94
Crystal Dimensions (mm)	0.22×0.19×0.16
Crystal System	triclinic
Space Group	P −1
Temperature (K)	113(2)
a (Å)	13.4514(10)
b (Å)	19.3322(12)
c (Å)	21.2194(13)
α (°)	97.023(7)
β (°)	101.173(7)
γ (°)	94.784(7)
V (Å^3)	5340.2(6)
Number of reflections to determine final unit cell	24521
Min and Max 2θ for cell determination (°)	3.56, 54.96
Z	4
F(000)	2112
λ (Å, Mo Kα)	0.71075
μ (mm^{-1})	2.301
Max 2θ for data collection (°)	54.97
Measured fraction of data	0.990
Number of reflections measured	52173
Unique reflections measured	24250
R_{merge}	15.5%
Number of parameters in least-squares	1129
R_1	0.0812
wR_2	0.1766
R_1 (all data)	0.2144
wR_2 (all data)	0.2400

Supplementary Table 4 | Reaction conditions screening of the intermolecular radical coupling.

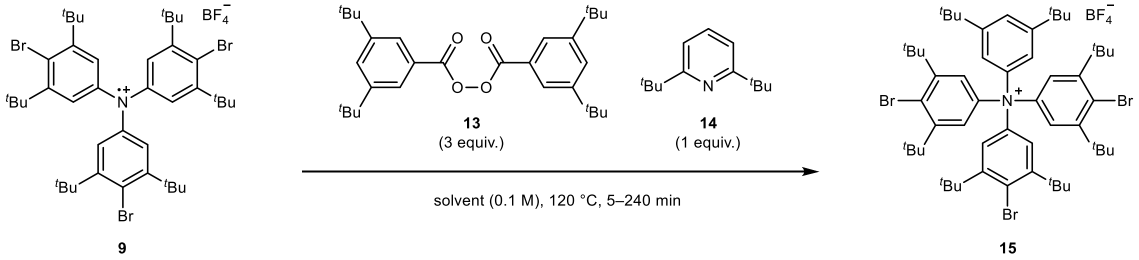

Entry	Solvent	Time (min)	Yield (%)*
1	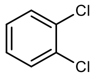	15	0.12
2[†]	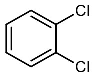	15	0.12
3	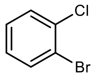	10	0.044
4	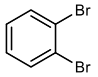	10	0.098
5	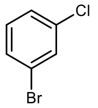	10	0.049
6	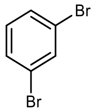	15	0.046
7	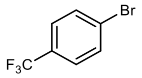	5	0.081
8	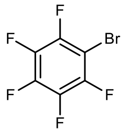	5	0.080
9	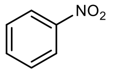	15	0.099
10	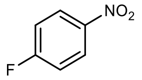	5	0.060
11	DMF	5	n.d.[‡]
12[§]	MeNO$_2$	120	n.d.[‡]
13[§]	1,4-dioxane	15	n.d.[‡]
14[§]	CCl$_4$	20	n.d.[‡]
15[‖]	MeCN	15	0.18
16[‖]	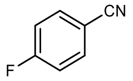	10	0.24
17[‖]	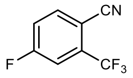	10	0.20
18[‖]	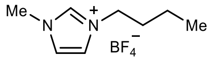	90	0.068

19[§]	1,2-dichloroethane	240	complex mixture[¶]
20[§]	acetic acid	15	complex mixture[¶]
21	benzonitrile	15	complex mixture[¶]

[*]Calculated by analyzing the ^{1}H NMR spectral profiles recorded using 1,3,5-trimethoxybenzene as the internal standard. [†]The reaction was carried out in the absence of **14**. It was difficult to purify the product **15** as various byproducts were also formed during the process. [‡]Not detected. [§]The reactions were conducted at 90 °C (Entries 12 and 13), 100 °C (Entry 14), 80 °C (Entry 19), and 110 °C (Entry 20). [‖]The product **15** could not be separated from the unidentified polar byproducts, which were probably derived from the solvents. [¶]The yield could not be determined because the reaction afforded a complex mixture of compounds which could not be identified.

Supplementary Table 5 | Summary of the crystal data for compound 22.

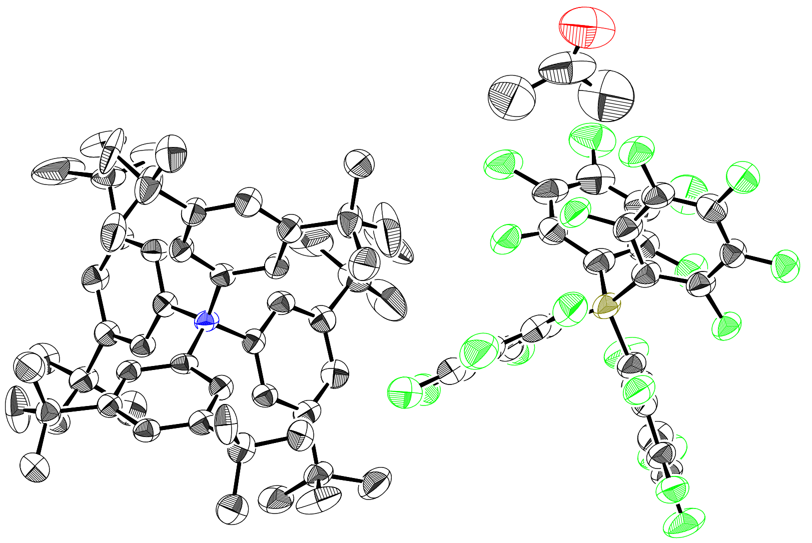

Thermal ellipsoids drawn at 50% probability. Hydrogen atoms and disorders are omitted for clarity.

CCDC registry	2117249
Formula	$C_{83}H_{90}BF_{20}NO$
Formula Weight (g/mol)	1508.36
Crystal Dimensions (mm)	0.35×0.29×0.15
Crystal System	triclinic
Space Group	P −1
Temperature (K)	123(2)
a (Å)	14.2167(11)
b (Å)	14.7943(13)
c (Å)	18.8043(17)
α (°)	91.320(6)
β (°)	95.357(7)
γ (°)	90.797(6)
V ($Å^3$)	3936.2(6)
Number of reflections to determine final unit cell	17489
Min and Max 2θ for cell determination (°)	3.44, 55.06
Z	2
F(000)	1576
λ (Å, Mo Kα)	0.71075
μ (mm^{-1})	0.107
Max 2θ for data collection (°)	54.97
Measured fraction of data	0.992
Number of reflections measured	38376
Unique reflections measured	17922
R_{merge}	9.69%
Number of parameters in least-squares	1074
R_1	0.0933
wR_2	0.2359
R_1 (all data)	0.2023
wR_2 (all data)	0.2904

Supplementary Table 6 | Summary of the crystal data for compound 24.

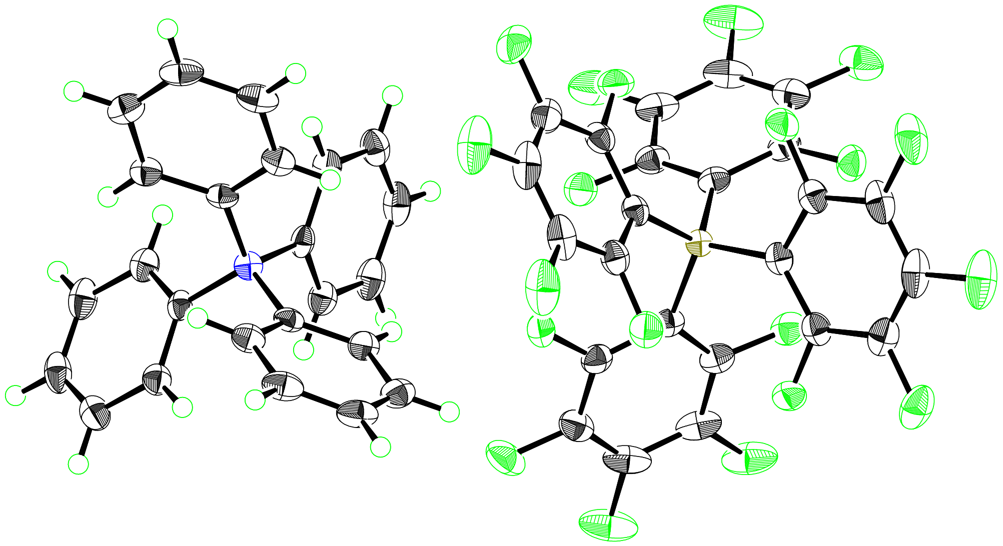

Thermal ellipsoids drawn at 50% probability.

CCDC registry	2117251
Formula	$C_{48}H_{20}BF_{20}N$
Formula Weight (g/mol)	1001.46
Crystal Dimensions (mm)	0.25×0.18×0.10
Crystal System	tetragonal
Space Group	I −4
Temperature (K)	123(2)
a (Å)	16.255(3)
b (Å)	16.255(3)
c (Å)	7.6655(8)
α (°)	90.000
β (°)	90.000
γ (°)	90.000
V (Å^3)	2025.5(8)
Number of reflections to determine final unit cell	6363
Min and Max 2θ for cell determination (°)	3.54, 54.70
Z	2
F(000)	1000
λ (Å, Mo Kα)	0.71075
μ (mm^{-1})	0.160
Max 2θ for data collection (°)	54.73
Measured fraction of data	0.999
Number of reflections measured	9782
Unique reflections measured	2303
R_{merge}	5.84%
Number of parameters in least-squares	158
R_1	0.0423
wR_2	0.0908
R_1 (all data)	0.0622
wR_2 (all data)	0.0988

2. Supplementary Figures 1–5

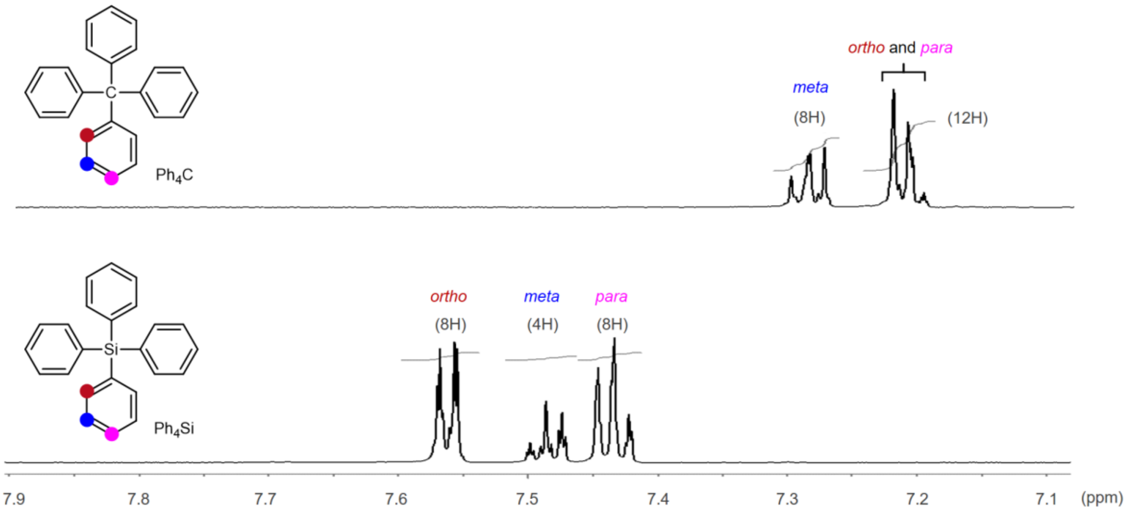

Supplementary Fig. 1 | Comparison of the ^{1}H NMR spectral profiles (600 MHz, $(CD_3)_2CO$) recorded for Ph_4C and Ph_4Si. The numbers of protons are presented in the parentheses.

a

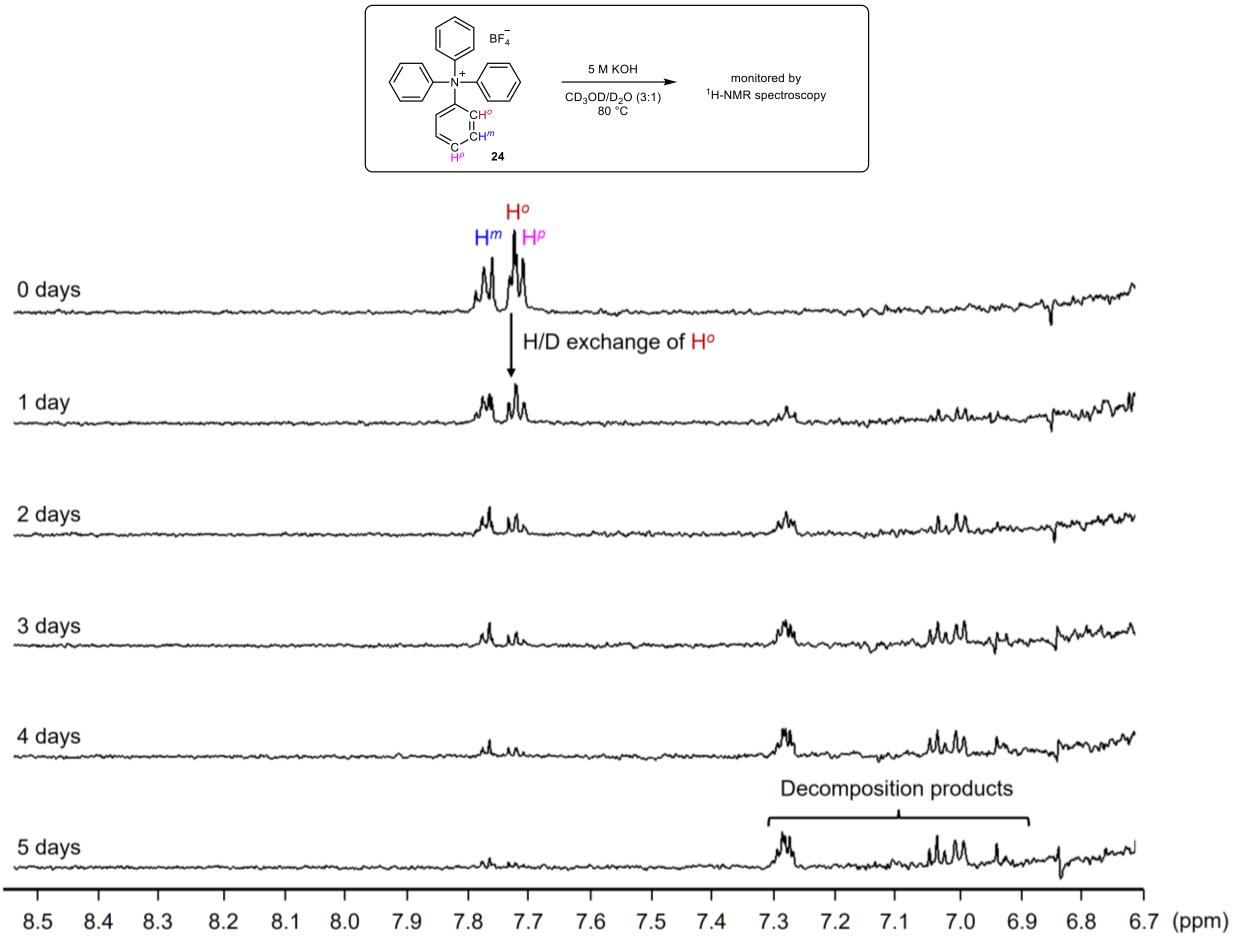

b

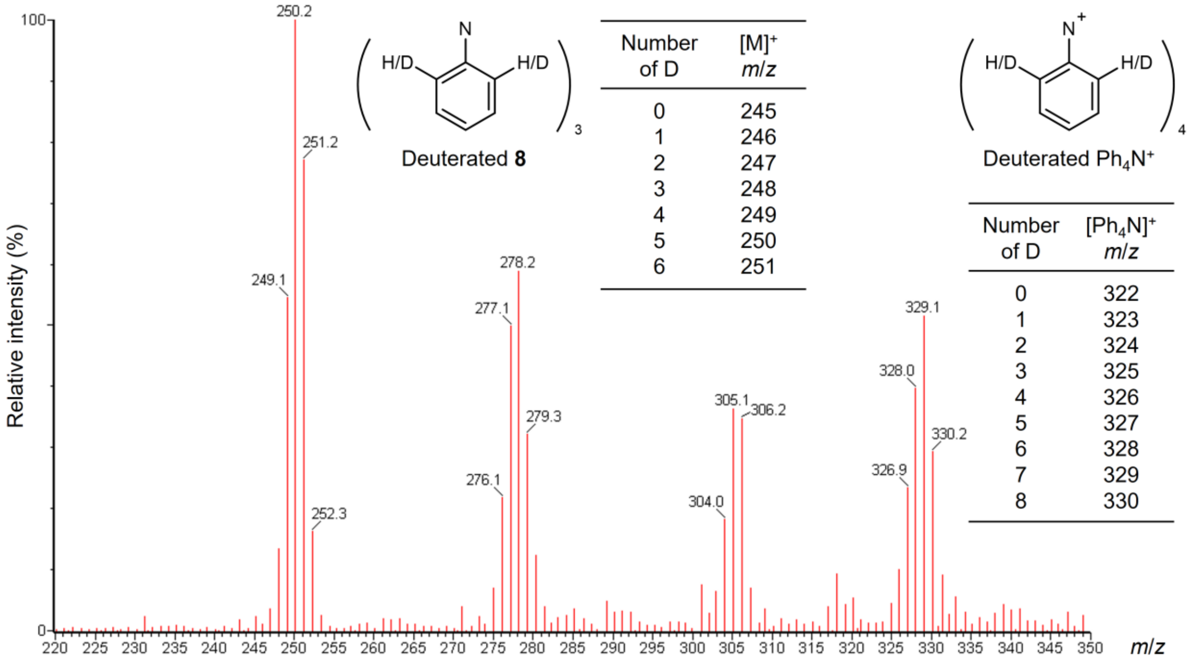

Number of D	$[M]^+$ *m/z*
0	245
1	246
2	247
3	248
4	249
5	250
6	251

Number of D	$[Ph_4N]^+$ *m/z*
0	322
1	323
2	324
3	325
4	326
5	327
6	328
7	329
8	330

Supplementary Fig. 2 | Alkaline stability of 24. a, ^{1}H NMR monitoring of **24** in CD_3OD/D_2O (3:1, containing 5 M KOH) at 80 °C. **b**, ESI-MS spectrum (positive-ion mode) of the organic extract obtained from the reaction mixture (day 5).

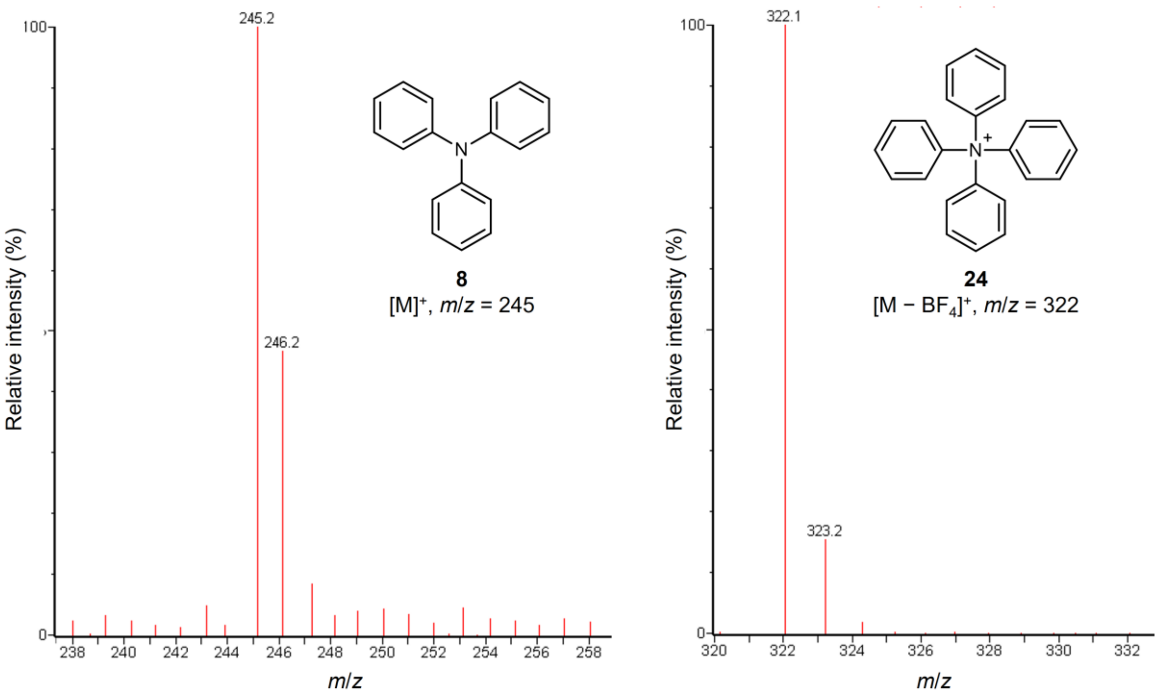

Supplementary Fig. 3 | ESI-MS spectra (positive-ion mode) of 8 and 24.

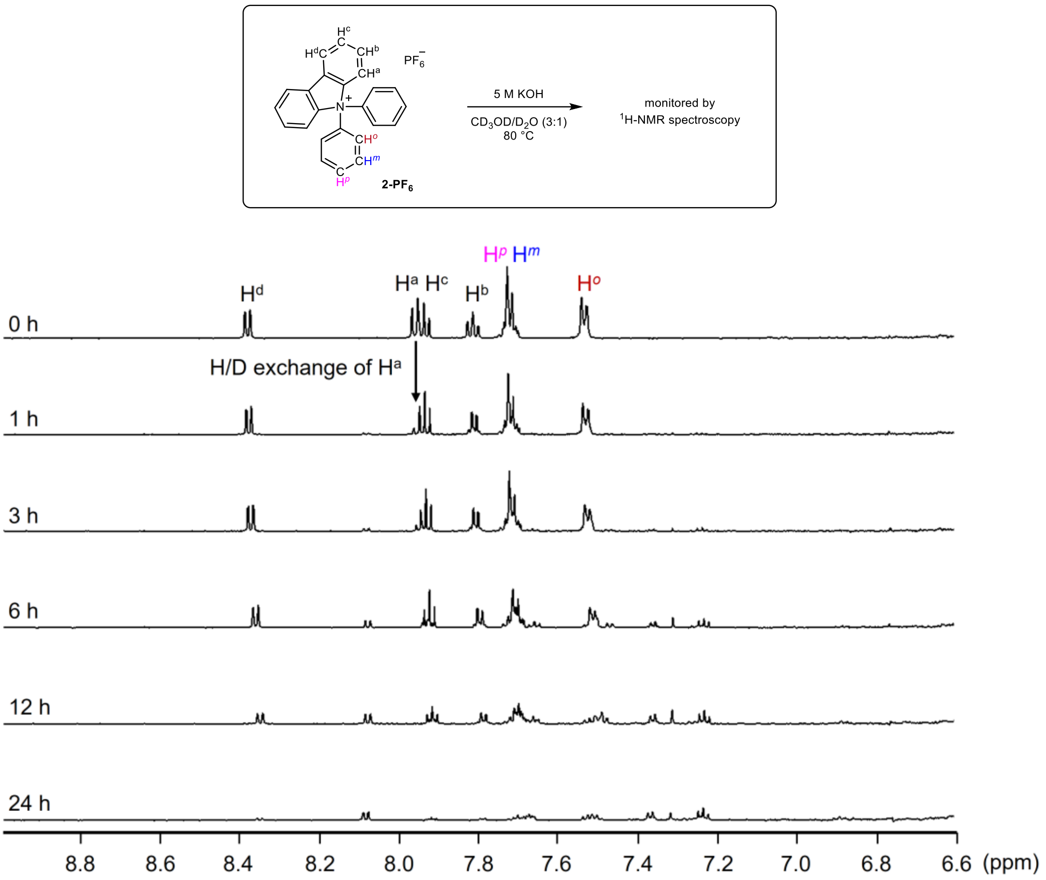

Supplementary Fig. 4 | Analysis of 2-PF$_6$ under alkaline conditions using ^{1}H NMR spectroscopy.

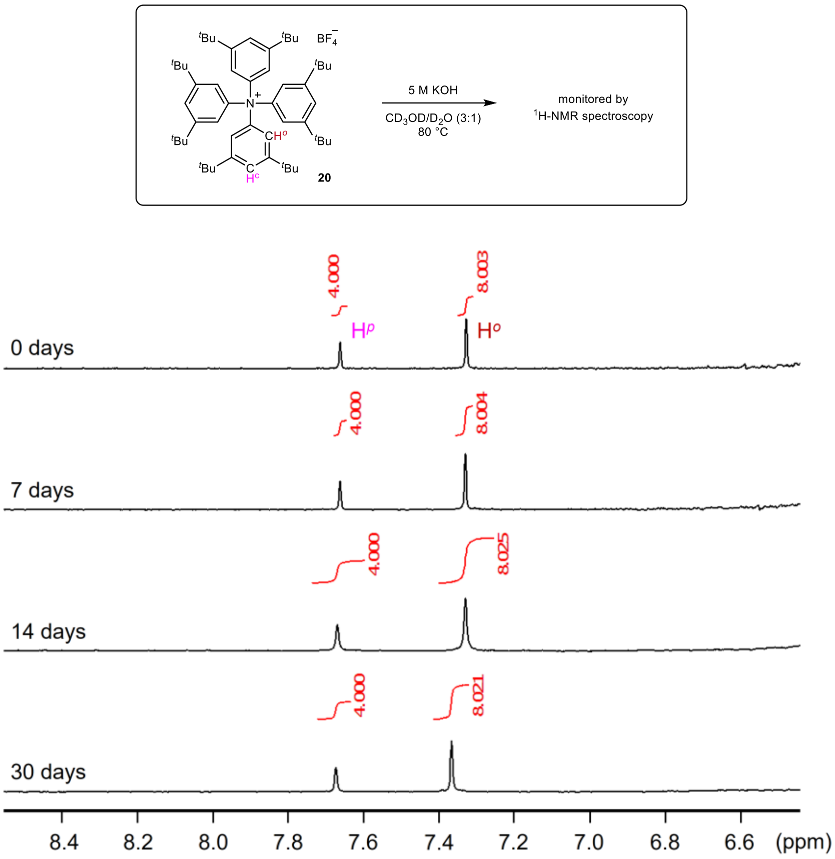

Supplementary Fig. 5 | Analysis of 20 under alkaline conditions using ^{1}H NMR spectroscopy.

3. Supplementary Methods

3.1. General information

NMR spectra were recorded on a JEOL JNM-ECS400 spectrometer [^{13}C NMR (100 MHz) and ^{19}F NMR (376 MHz)] or a JEOL JNM-ECA600 spectrometer [^{1}H NMR (600 MHz), ^{13}C NMR (150 MHz), and ^{19}F NMR (565 MHz)] at 20 °C unless otherwise noted. Chemical shifts for ^{1}H NMR are reported in parts per million (δ) relative to tetramethylsilane (δ 0.00) as the internal standard. Coupling constants (J) are reported in hertz (Hz). The following abbreviations are used for spin multiplicity: s = singlet, d = doublet, dd = double doublet, m = multiplet. Chemical shifts for ^{13}C NMR are reported in parts per million (δ) relative to the solvents [$CDCl_3$, δ 77.16; CD_3OD, δ 49.00; or $(CD_3)_2CO$; δ 29.84]. Chemical shifts for ^{19}F NMR are reported in parts per million (δ) relative to fluorobenzene (δ −113.0) as the external standard. IR spectra were measured with a Thermo Scientific iD7 ATR Accessory with a diamond crystal for the Thermo Scientific Nicolet iS5 FT-IR Spectrometer. Mass spectra were measured on a JMS-T100TD AccuTOF TLC (DART-MS and ESI-MS) or Micromass Zq2000 (ESI-MS) spectrometer. Melting points were determined with a Yanagimoto melting point apparatus and were uncorrected. Measurements for X-ray single-crystal structure analysis were made on a Rigaku R-AXIS RAPID diffractometer. The structures were solved by SHELXT[15] or SIR2014[16] and were refined using SHELXL[17] on Yadokari-XG 2009[18]. Analytical thin layer chromatography (TLC) was performed using glass plates precoated with 0.25 mm silica gel impregnated with a fluorescent indicator (254 nm). Preparative TLC separations were performed using glass plates precoated with 0.50 mm silica gel or diol-functionalized silica gel (Chromatorex DIOL TLC plates, Fuji Silysia Chemical) impregnated with a fluorescent indicator (254 nm). Flash chromatography was performed using silica gel (spherical, neutral, 40–100 mesh) or diol-functionalized silica gel (Chromatorex DIOL MB100–40/75, Fuji Silysia Chemical). $NaBF_4$-treated silica gel was prepared by treatment of silica gel (spherical, neutral, 40–100 mesh) with $NaBF_4$-saturated MeOH (2.5 ml per 1 g of silica gel) and washing with MeOH followed $CHCl_3$. $NaBF_4$-treated silica gel TLC plates were prepared by immersing Merck analytical plates (0.50 mm thick, precoated with silica gel 60 F254) in $NaBF_4$-saturated MeOH followed by air-drying. Recycling preparative HPLC was performed with Japan Analytical Industry LC-928 equipped with GPC columns Jaigel-1H and 2H. All reactions sensitive to oxygen or moisture were conducted under a nitrogen atmosphere. Reagents were commercial grades and were used without any purification unless otherwise noted. A lithium tetrakis(pentafluorophenyl)borate–ethyl ether complex [$LiB(C_6F_5)_4$–Et_2O] was purchased from Tokyo Chemical Industry. Known compounds [(2,6-di-*tert*-butyl)pyridinium tetrafluoroborate (**21**)[19] and *N*,*N′*-diphenylcarbazolium hexafluorophosphate (**2-PF$_6$**)[20]] were prepared according to the reported procedure.

3.2. Experimental procedure and characterization data

Bis(3,5-di-*tert*-butyl)benzoyl peroxide (13).

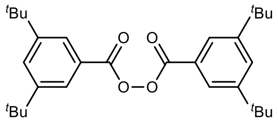

Following a general procedure,[21,22] 1,1'-carbonyldiimidazole (1.95 g, 12.0 mmol) was added to a solution of (3,5-di-*tert*-butyl)benzoic acid (2.33 g, 9.94 mmol) in CH_2Cl_2 (25 ml) at room temperature. After 16.5 h, the reaction mixture was quenched with excess H_2O, and then extracted with CH_2Cl_2. The organic layer was washed with brine, dried (Na_2SO_4), and filtered. The filtrate was concentrated under reduced pressure. The residue was dissolved in THF (10 ml), and the solution was treated with aqueous H_2O_2 (30 w/w%, 510 μl, 4.99 mmol) at room temperature. After 20 min, the mixture was concentrated under reduced pressure. The residue was washed with MeOH to afford a white solid (1.99 g, 86%). TLC (hexane/EtOAc = 90:10): RF = 0.50; ^{1}H NMR (600 MHz, $CDCl_3$): δ 7.91 (d, J = 1.9 Hz, 4H), 7.72 (t, J = 1.9 Hz, 2H), 1.37 (s, 36H); ^{13}C NMR (150 MHz, $CDCl_3$): δ 164.1, 151.8, 128.7, 125.3, 124.1, 35.2, 31.4; HRMS (DRAT-TOF, *m*/*z*): $[M + H]^+$ calcd for $C_{30}H_{43}O_4$, 467.3161; found, 467.3154.

3',5'-Di-*tert*-butyl-3,4-dichloro-1,1'-biphenyl (16)

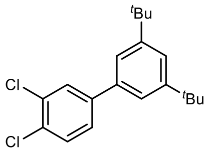

White solid; mp.: 107–108 °C; TLC (hexane): RF = 0.50; ^{1}H NMR (600 MHz, $CDCl_3$): δ 7.65 (d, J = 2.1 Hz, 1H), 7.49 (d, J = 8.3 Hz, 1H), 7.47 (t, J = 1.7 Hz, 1H), 7.41 (dd, J = 8.3, 2.1 Hz, 1H), 7.34 (d, J = 1.7 Hz, 2H), 1.37 (s, 18H); ^{13}C NMR (100 MHz, $CDCl_3$): δ 151.7, 142.7, 138.4, 132.8, 131.2, 130.7, 129.3, 126.9, 122.4, 121.6, 35.2, 31.6; HRMS (DART-TOF, *m*/*z*): $[M]^+$ calcd for $C_{20}H_{24}Cl_2$, 334.1255; found, 334.1255.

{4-Bromo-3,5-di-*tert*-butyl-2-[(3,5-di-*tert*-butyl)benzoyloxy]phenyl}bis[(4-bromo-3,5-di-*tert*-butyl)phenyl]amine (17).

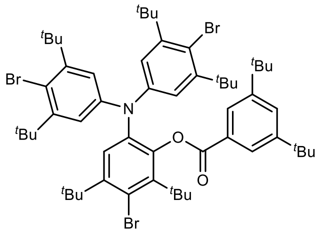

Colorless crystals; TLC (hexane): RF = 0.11; ^{1}H NMR (600 MHz, 343 K, C_6D_6): δ 7.96 (s, 2H), 7.64 (s, 1H), 7.58 (s, 1H), 7.44 (br s, 4H), 1.76 (s, 9H), 1.49 (s, 9H), 1.46 (s, 36H), 1.21 (s, 18H); ^{13}C NMR (150 MHz, $CDCl_3$): δ 164.4, 151.0, 149.4, 148.0, 144.9, 143.4, 139.2, 128.6, 128.1, 128.0, 127.8, 126.1, 124.3, 121.8,

40.1, 38.5, 38.2, 35.0, 33.2, 31.5, 31.2, 31.0; HRMS (ESI-TOF, *m*/*z*): $[M]^+$ calcd for $C_{57}H_{80}Br_3NO_2$, 1047.3739; found, 1047.3719. Suitable crystals for X-ray analysis were obtained by recrystallization (hexane/$CHCl_3$).

Bis[(4-bromo-3,5-di-*tert*-butyl)phenyl](5-bromo-3',4,5',6-tetra-*tert*-butyl-[1,1'-biphenyl]-2-yl)amine (18).

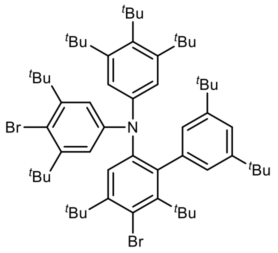

White solid; TLC (hexane): RF = 0.51; ^1H NMR (600 MHz, $CDCl_3$): δ 7.28 (s, 1H), 7.06 (t, J = 1.7 Hz, 1H), 6.72 (d, J = 1.7 Hz, 2H), 6.70 (s, 4H), 1.61 (s, 9H), 1.40 (s, 36H), 1.29 (s, 9H), 1.04 (s, 18H); ^{13}C NMR (100 MHz, $CDCl_3$): δ 151.7, 149.9, 149.02, 149.00, 144.4, 143.6, 142.3, 139.4, 128.4, 125.5, 125.0, 119.3, 118.2, 115.1, 41.1, 38.4, 38.2, 34.6, 33.7, 31.4, 31.0; HRMS (ESI-TOF, *m*/*z*): $[M]^+$ calcd for $C_{56}H_{80}Br_3N$, 1003.3841; found, 1003.3814. Suitable crystals for X-ray analysis were obtained by recrystallization (hexane/$CHCl_3$).

Tetraphenylammonium tetrakis(pentafluorophenyl)borate (24).

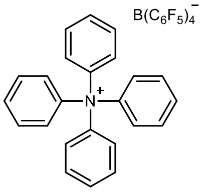

Ph_4N^+ salt **24** melted at 281–282 °C. After it was cooled to room temperature, a pale brown solid was obtained. TLC analysis detected partial decomposition of **24**. On the other hand, no decomposition was detected by TLC analysis after **24** was heated to 244 °C for 10 min. This result suggested that the thermal stability of **24** may be higher than Ph_4P^+ salt **25**, which was reported to be decomposed at 222–240 °C.[23]

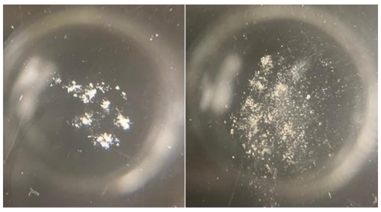

A sample of **24** After melting

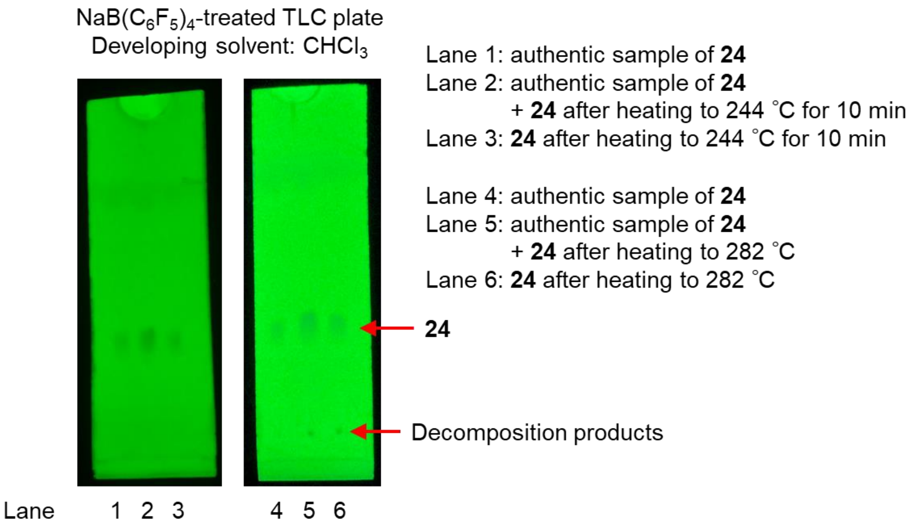

Tetraphenylphosphonium tetrakis(pentafluorophenyl)borate (25).[23]

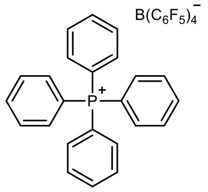

A solution of tetraphenylphosphonium chloride (30 mg, 80 μmol) and $LiB(C_6F_5)_4$–Et_2O (72 mg, 96 μmol as a 1:1 complex) in acetone (1.6 ml) was stirred for 15 min at room temperature. The reaction mixture was directly purified by column chromatography (silica gel, acetone) to afford a white solid (71 mg, 87%). TLC ($CHCl_3$): RF = 0.46; ^{1}H NMR [600 MHz, $(CD_3)_2CO$]: δ 8.05–7.98 (m, 4H), 7.92–7.84 (m, 16H); ^{13}C NMR [150 MHz, $(CD_3)_2CO$]: δ 136.4 (d, J = 2.9 Hz), 135.7 (d, J = 10.1 Hz), 131.4 (d, J = 12.9 Hz), 119.1 (d, J = 89.0 Hz); ^{19}F NMR (565 MHz, $CDCl_3$): δ −132.6, −162.9, −166.7; LRMS (ESI-TOF, m/z): $[M - B(C_6F_5)_4]^+$ 339.

3.3. Experimental procedure for Supplementary Table 4

General procedure followed for screening the conditions to conduct the intermolecular radical coupling reaction.

A mixture of triarylammoniumyl (**9**, 80.0–100 mg, 0.0866–0.108 mmol, 1.0 equiv.), diacyl peroxide (**13**, 124–154 mg, 0.266–0.330 mmol, 3.0 equiv.), and **14** (20–25 μl, 0.089–0.11 mmol, 1.0 equiv.) in a solvent (0.70–1.1 mL, 0.10–0.12 M) was heated in the temperature range of 80–120 °C for a time in the range of 5–240 min. Following this, the temperature of the reaction mixture was brought down to room temperature. Following the process of partial purification to remove byproducts including **11**, **16**, **17**, and **18**, the yield of **15** was determined using the ^{1}H NMR spectroscopy technique (600 MHz, CD_3OD). 1,3,5-Trimethoxybenzene was used as the internal standard (singlet at 6.07 ppm, corresponding to 3H, was considered the diagnostic signal). A singlet observed in the range of 7.46–7.47 ppm was used as the diagnostic product signal for calculating the yield. The signal corresponded to the *ortho* protons (6H) present on the brominated aromatic rings in **15**.

Purification method for entries 1–14, 16, 17, and 19–21: The reaction mixture was diluted with $CHCl_3$, following which it was filtered. The filtrate was passed through a $NaBF_4$-treated silica gel (eluent: $CHCl_3$/MeOH = 100:0 (to 90:10)). The eluent was concentrated under reduced pressure.

Purification method for entry 15: The reaction mixture was concentrated under reduced pressure. $CHCl_3$ was added to the residue, and the resulting suspension was filtered. The filtrate was passed through a $NaBF_4$-treated silica gel (eluent: $CHCl_3$/MeOH = 100:0 (to 90:10)). The eluent was concentrated under reduced pressure.

Purification method for entry 18: The reaction mixture was diluted with $CHCl_3$ (10 mL) and washed with H_2O (3 × 3–5 mL). The organic layer was dried over Na_2SO_4, following which it was filtered. The filtrate was concentrated under reduced pressure. The residue was suspended in hexane, and the suspension was passed through a $NaBF_4$-treated silica gel (eluent: hexane/$CHCl_3$/MeOH = 50:50:0 to 0:100:0 to 0:90:10). The eluent was concentrated under reduced pressure.

The ^{1}H NMR spectral profiles analyzed to calculate the yield of **15** are presented in the following pages. The recorded spectral profiles have been compared with the spectral profile recorded for pure **15**.

^{1}H NMR spectra used for the calculation of the yield of 15.

Reaction solvent:

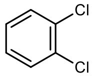

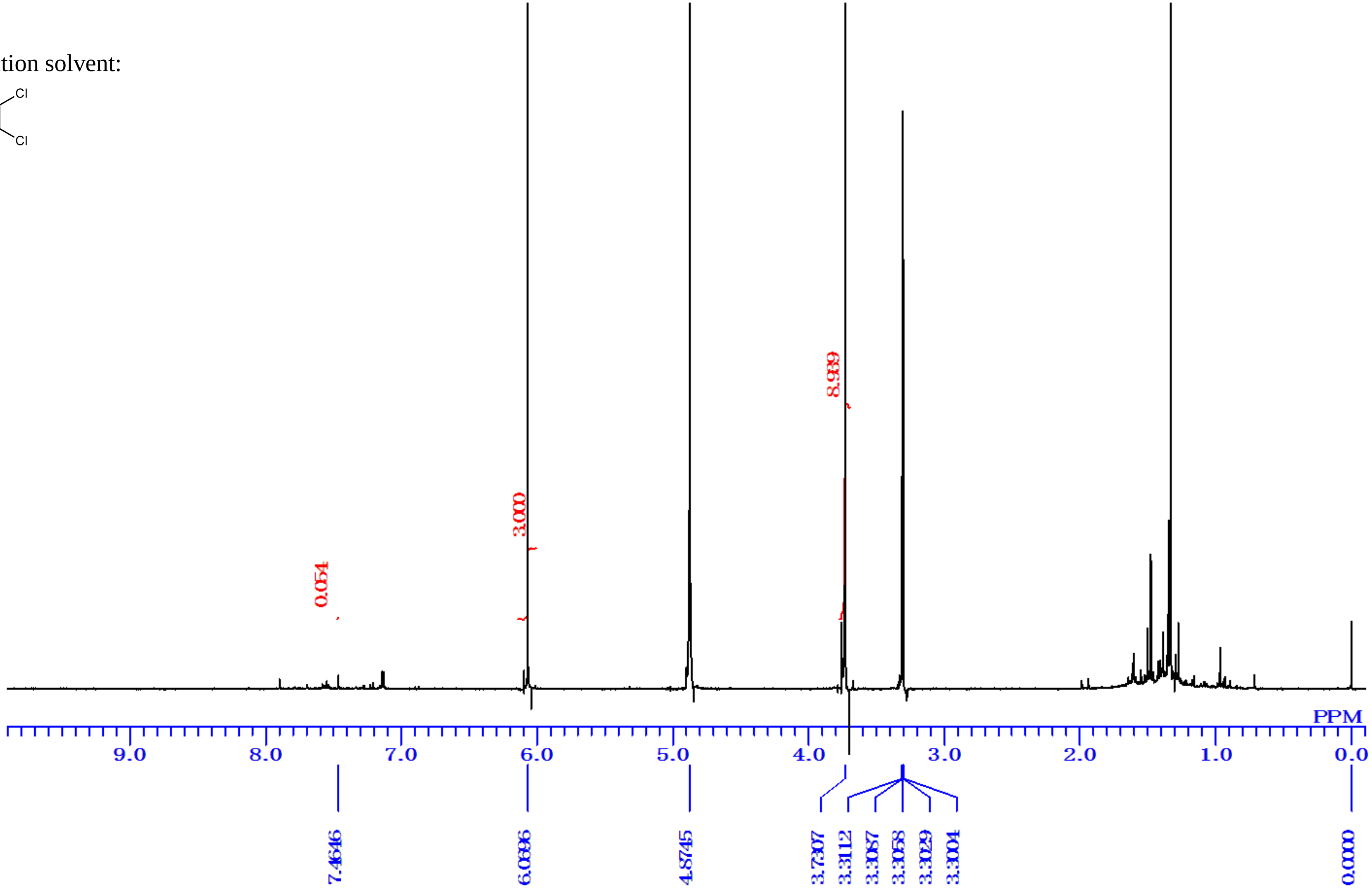

Supplementary Fig. 6 | ^{1}H NMR spectrum of the crude mixture (entry 1, 600 MHz, 20 °C, CD_3OD).

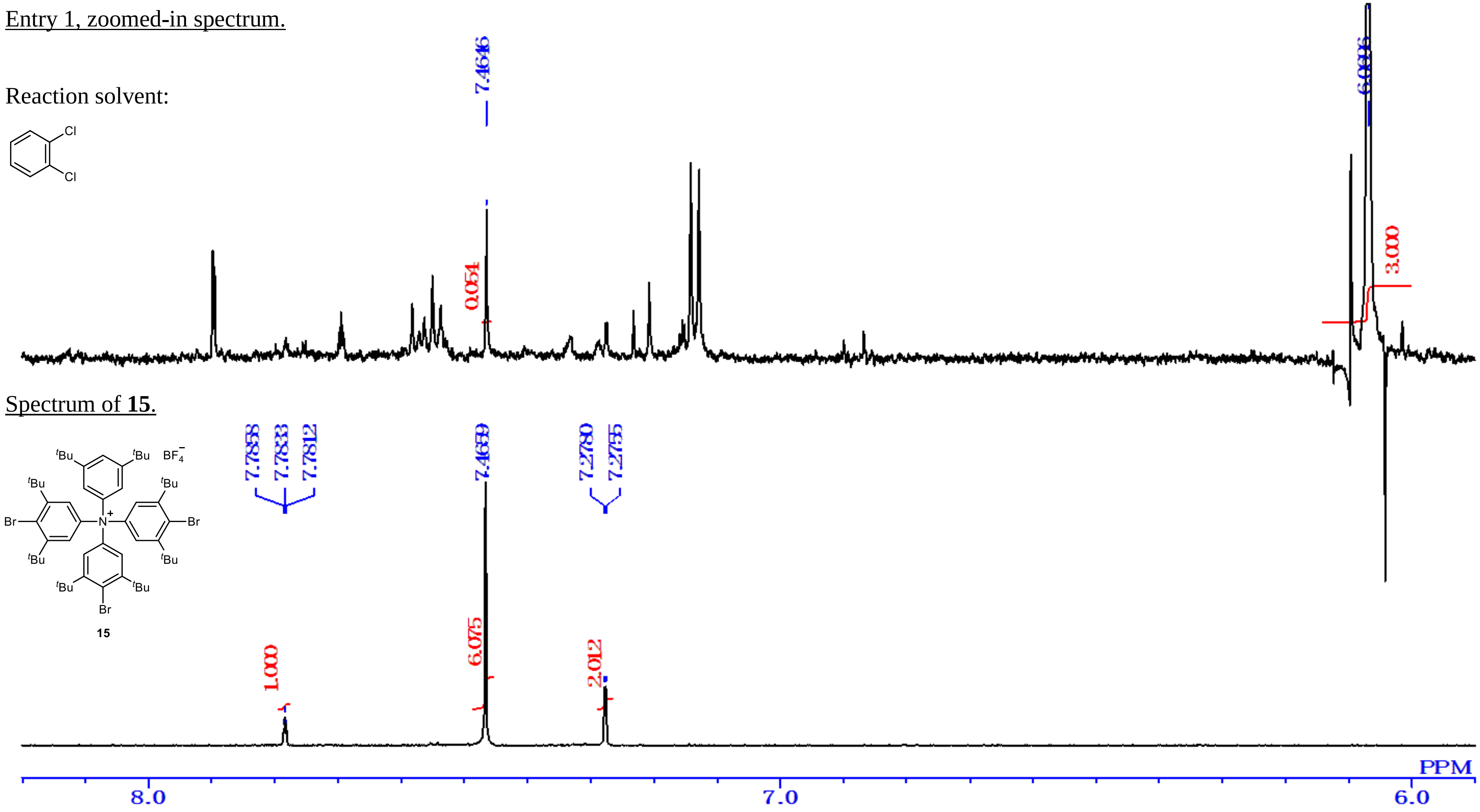

Supplementary Fig. 7 | Comparison of ^{1}H NMR spectra (600 MHz, 20 °C, CD_3OD) of the crude mixture (entry 1) and 15.

Reaction solvent:

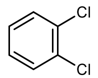

without **14**

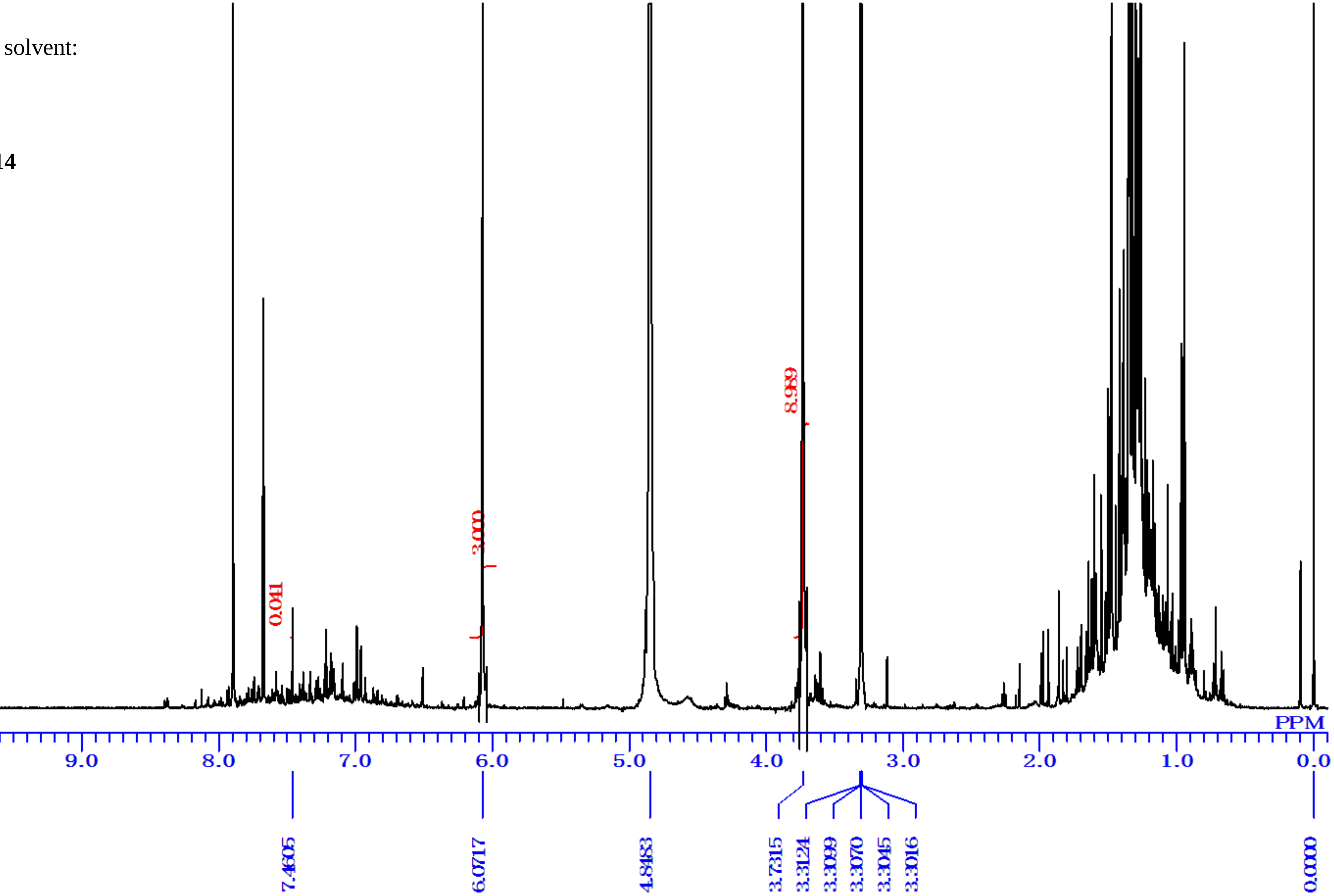

Supplementary Fig. 8 | ^{1}H NMR spectrum of the crude mixture (entry 2, 600 MHz, 20 °C, CD_3OD).

Entry 2, zoomed-in spectrum.

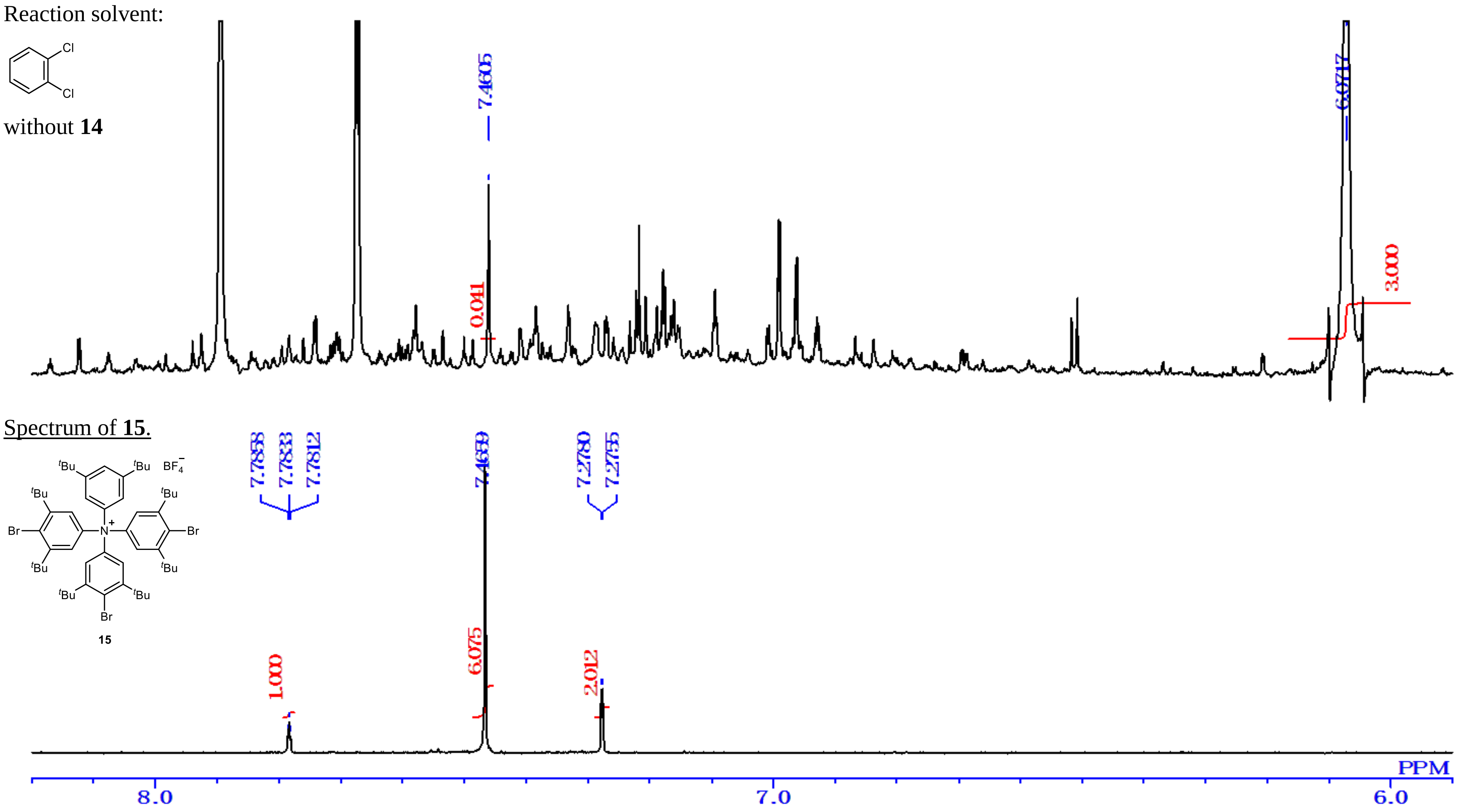

Supplementary Fig. 9 | Comparison of ^{1}H NMR spectra (600 MHz, 20 °C, CD_3OD) of the crude mixture (entry 2) and 15.

Reaction solvent:

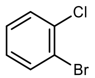

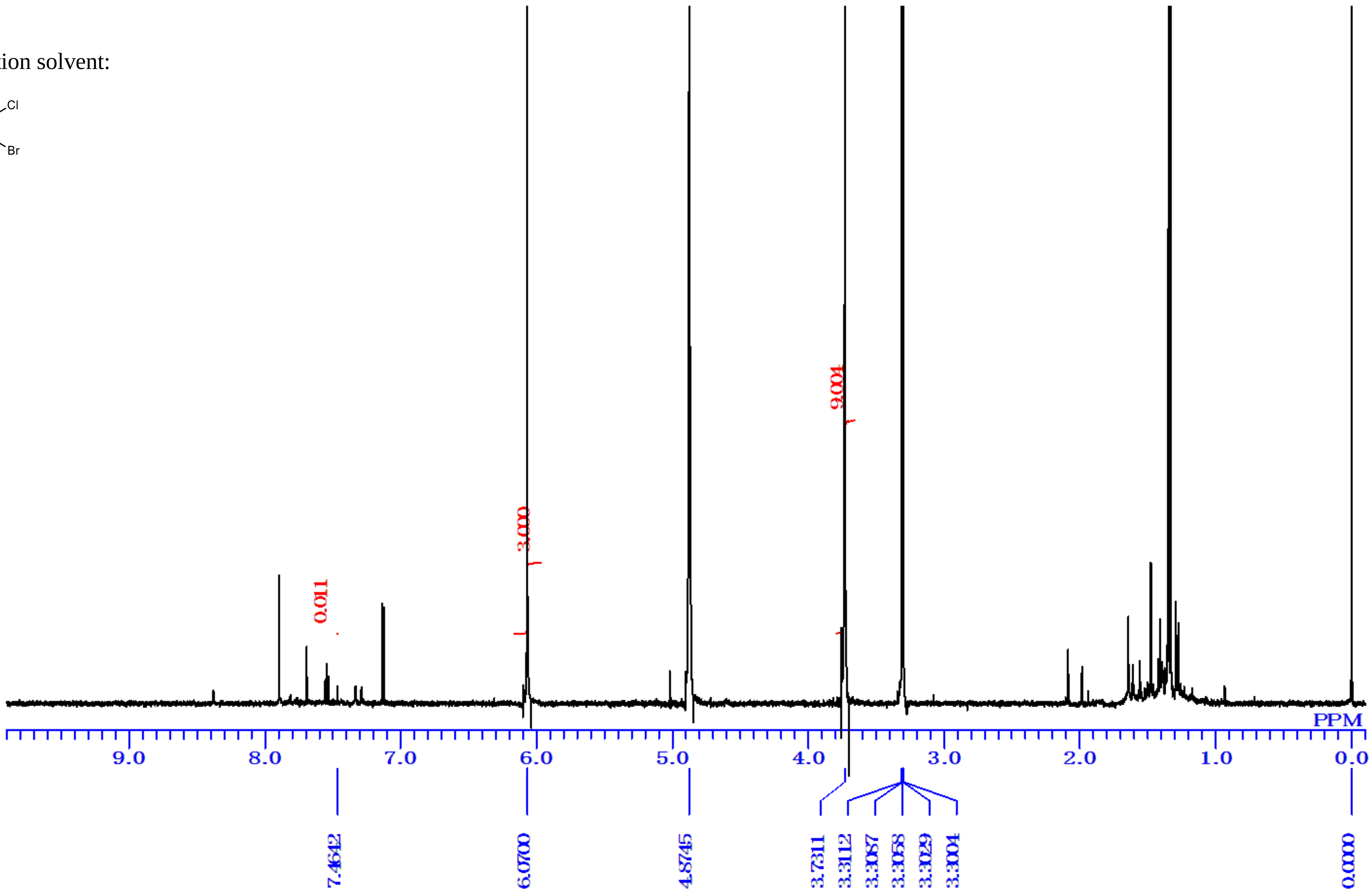

Supplementary Fig. 10 | ^{1}H NMR spectrum of the crude mixture (entry 3, 600 MHz, 20 °C, CD_3OD).

Entry 3, zoomed-in spectrum.

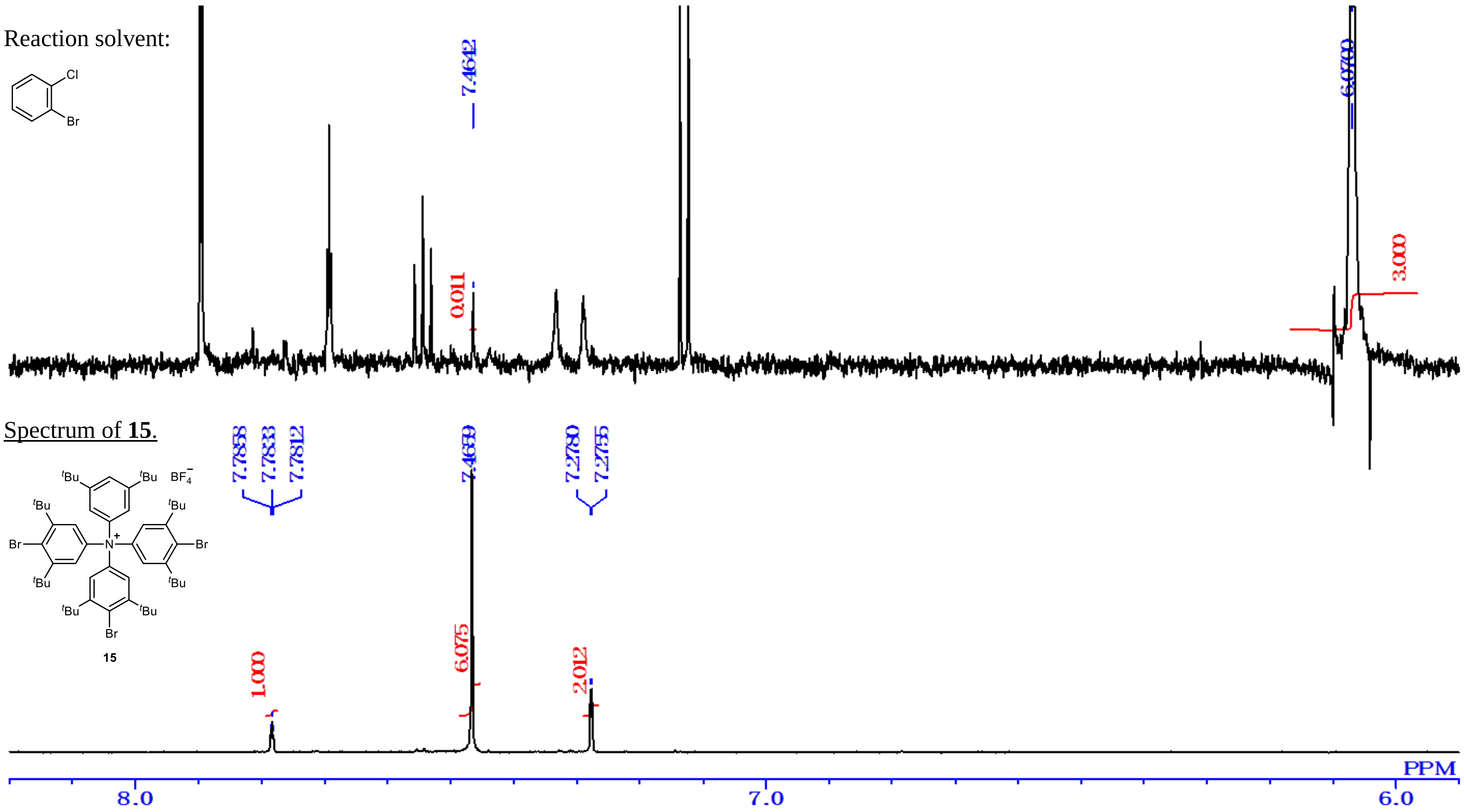

Supplementary Fig. 11 | Comparison of ^{1}H NMR spectra (600 MHz, 20 °C, CD₃OD) of the crude mixture (entry 3) and 15.

Reaction solvent:

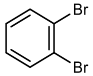

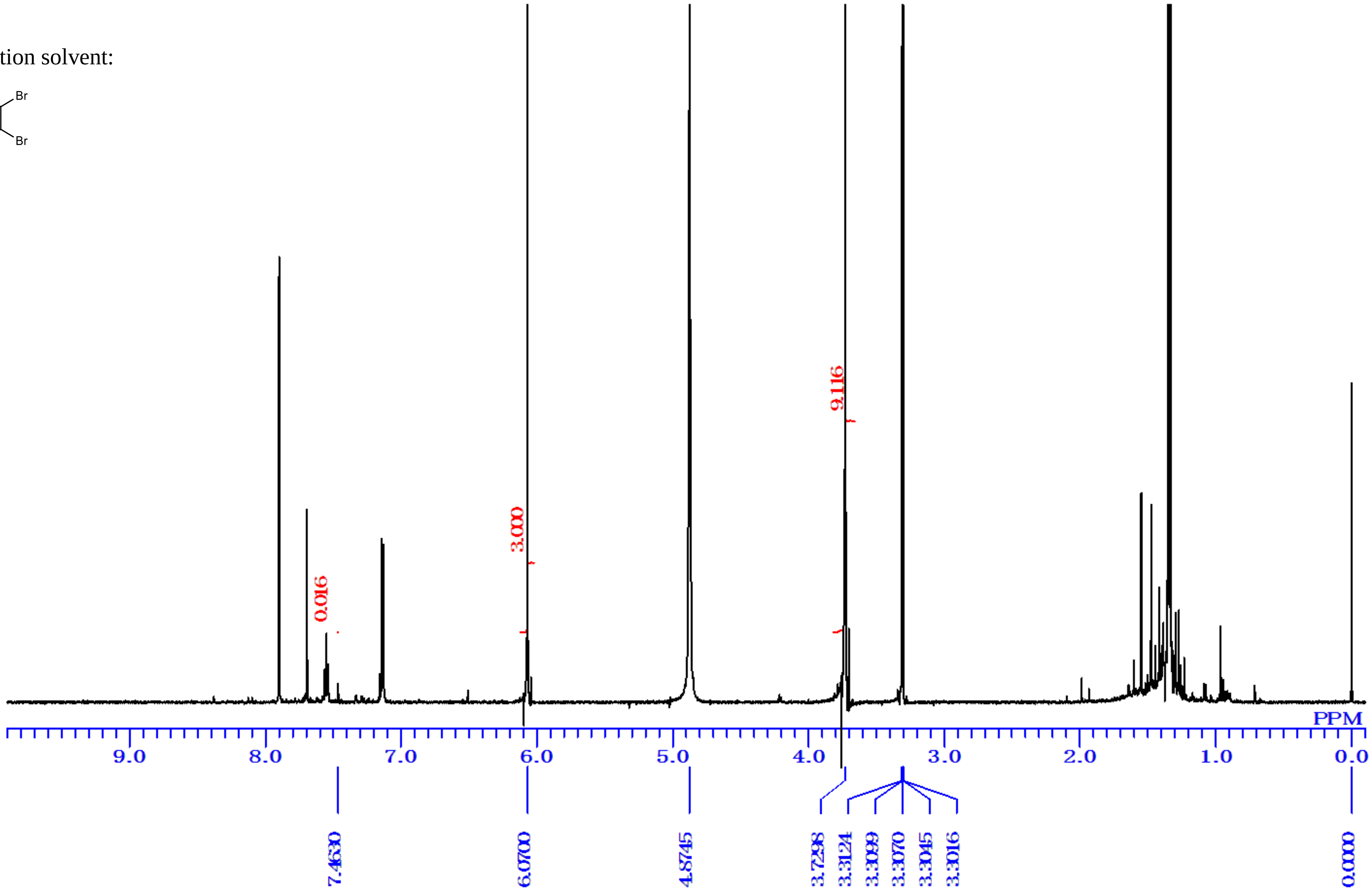

Supplementary Fig. 12 | ^{1}H NMR spectrum of the crude mixture (entry 4, 600 MHz, 20 °C, CD_3OD).

Entry 4, zoomed-in spectrum.

Reaction solvent:

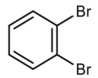

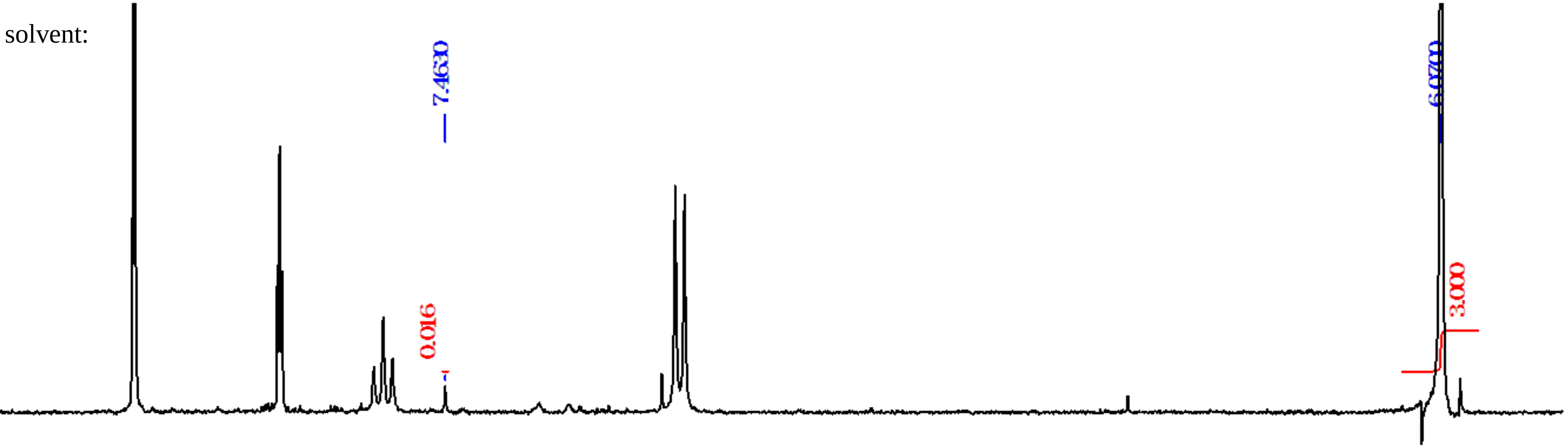

Spectrum of **15**.

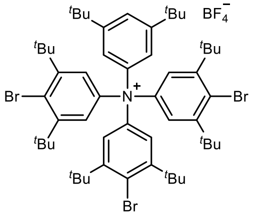

15

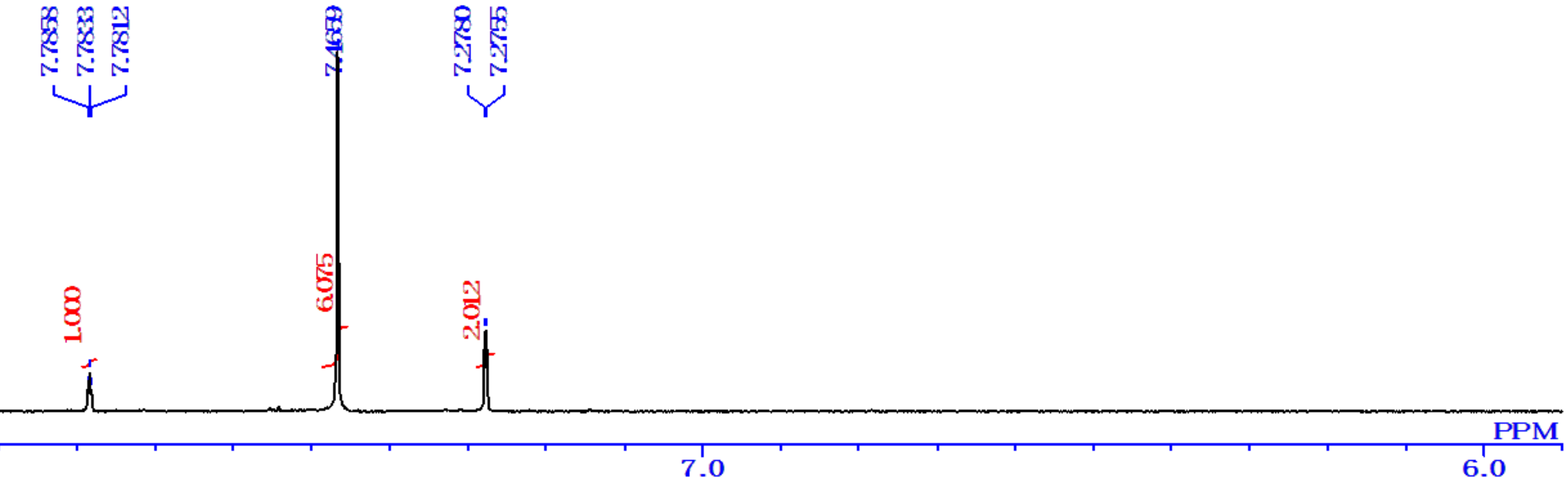

Supplementary Fig. 13 | Comparison of ^{1}H NMR spectra (600 MHz, 20 °C, CD_3OD) of the crude mixture (entry 4) and 15.

Reaction solvent:

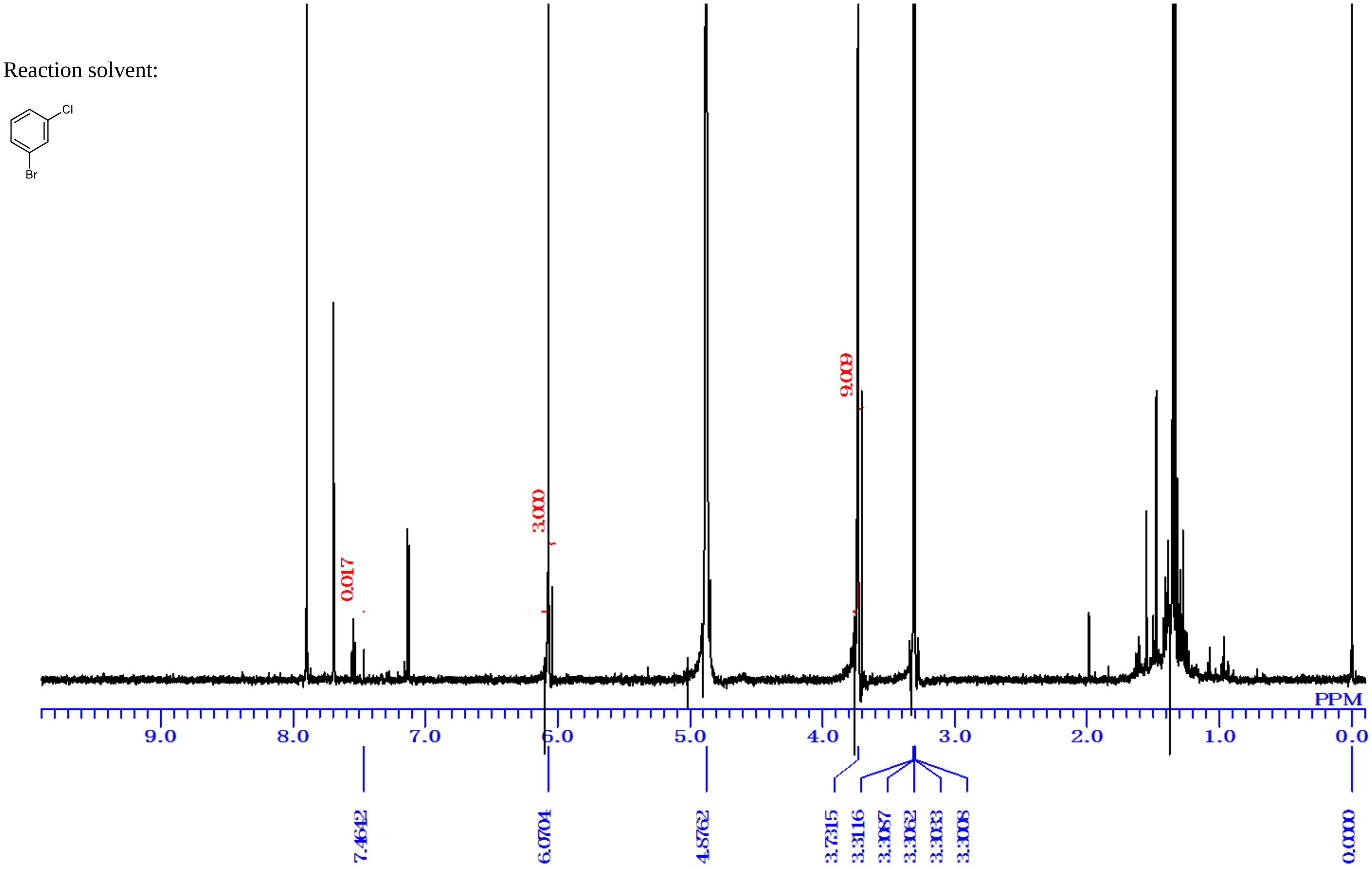

Supplementary Fig. 14 | ^{1}H NMR spectrum of the crude mixture (entry 5, 600 MHz, 20 °C, CD_3OD).

Entry 5, zoomed-in spectrum.

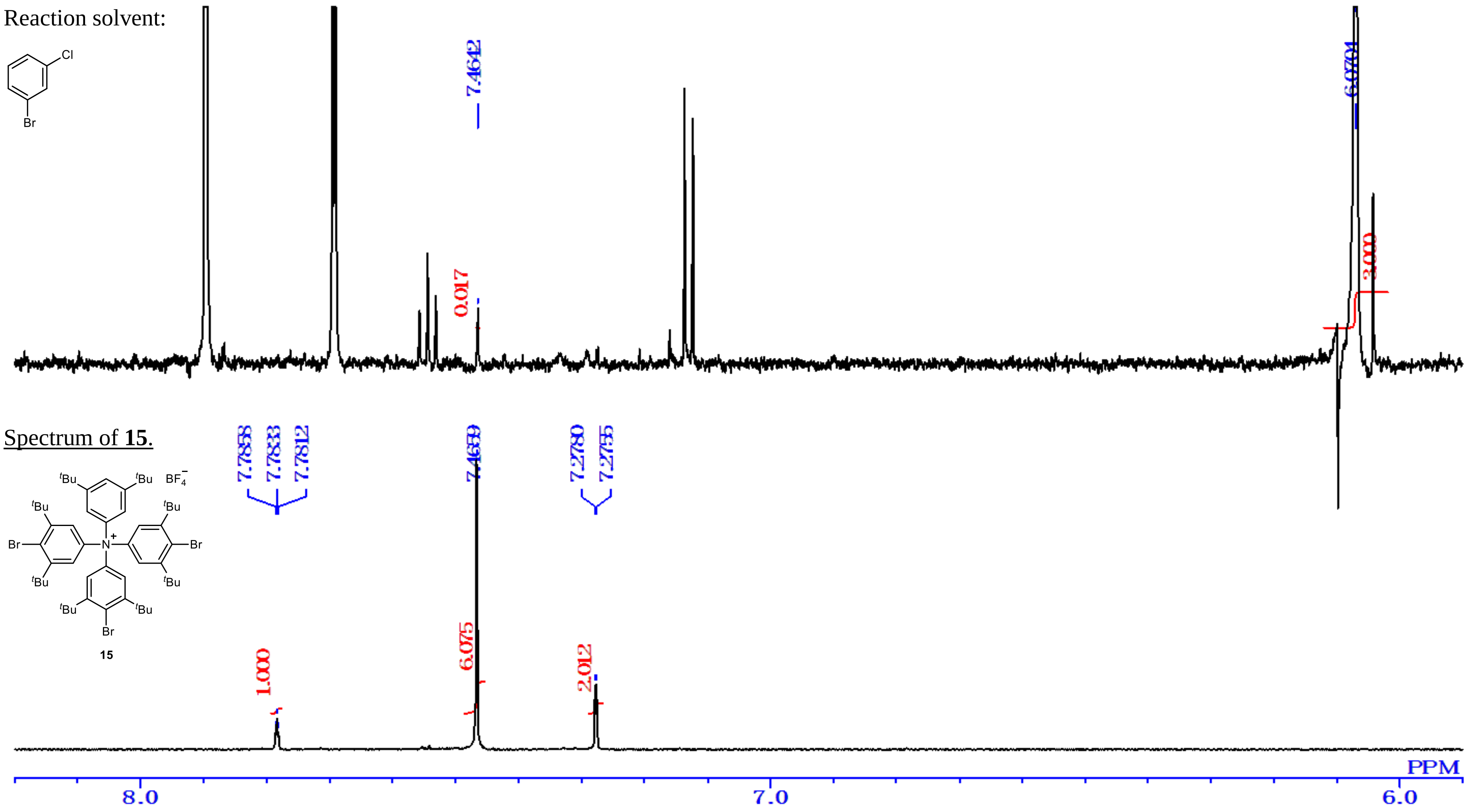

Supplementary Fig. 15 | Comparison of ^{1}H NMR spectra (600 MHz, 20 °C, CD_3OD) of the crude mixture (entry 5) and 15.

Reaction solvent:

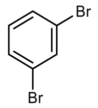

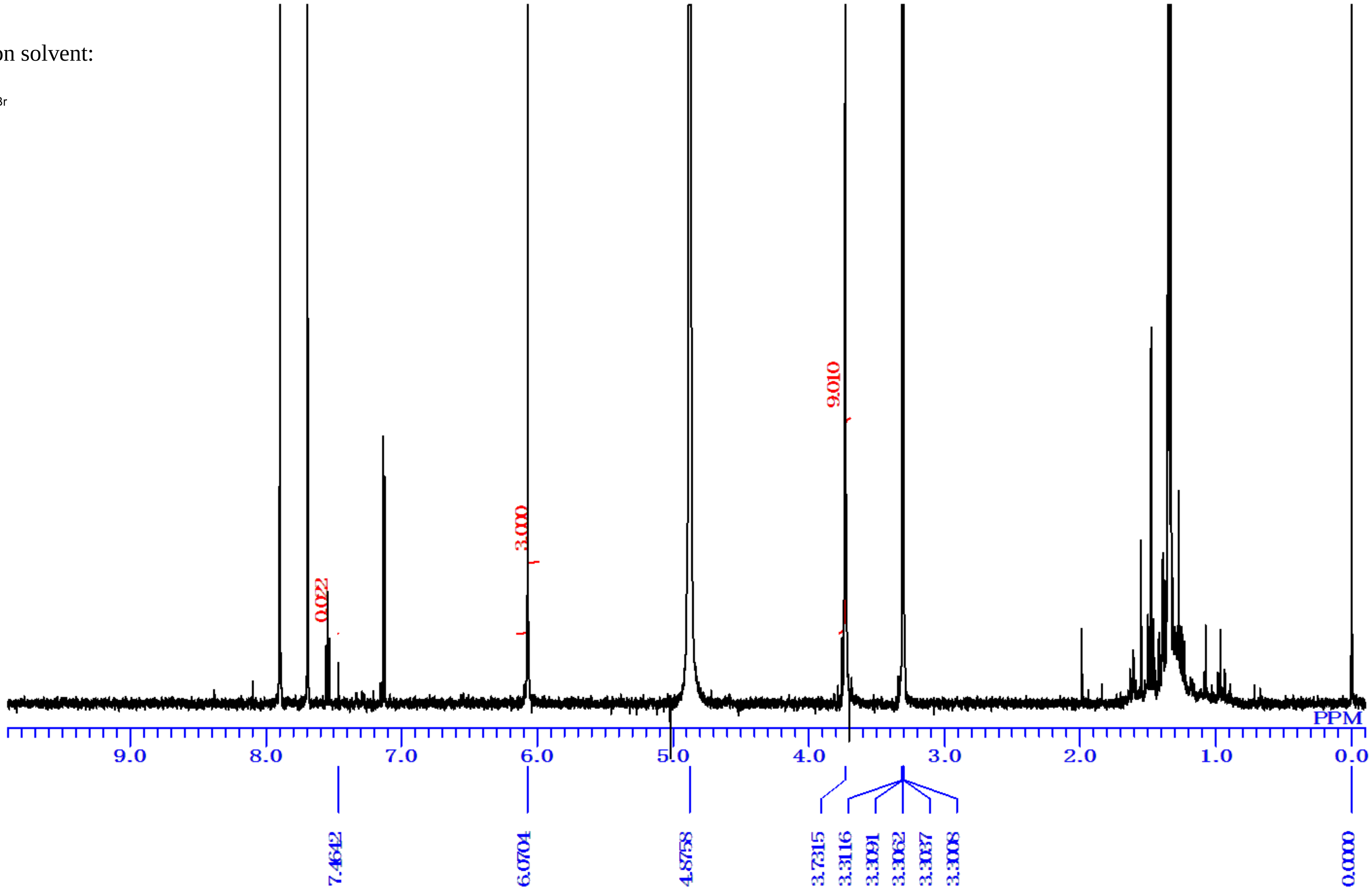

Supplementary Fig. 16 | ^{1}H NMR spectrum of the crude mixture (entry 6, 600 MHz, 20 °C, CD_3OD).

Entry 6, zoomed-in spectrum.

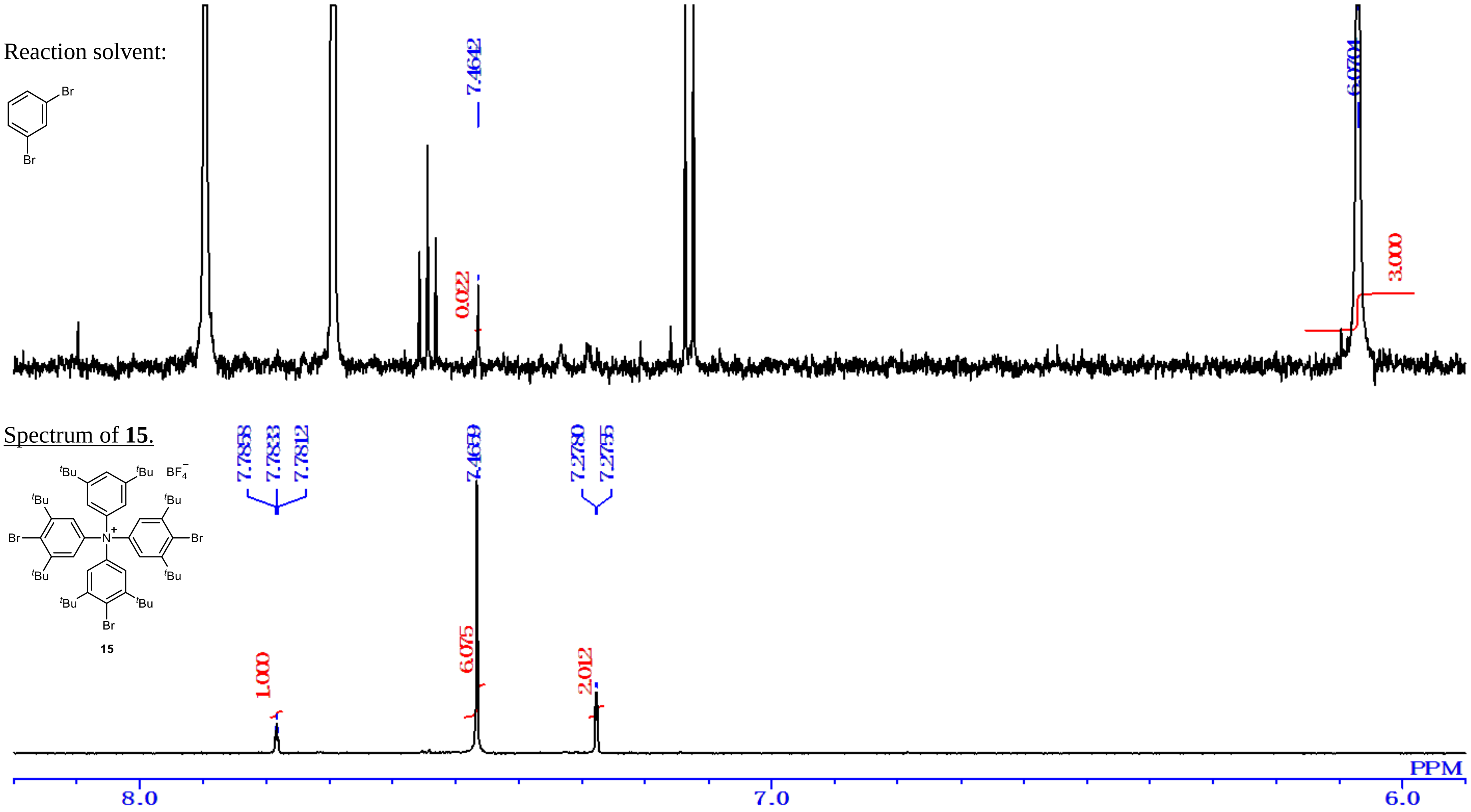

Supplementary Fig. 17 | Comparison of ^{1}H NMR spectra (600 MHz, 20 °C, CD_3OD) of the crude mixture (entry 6) and 15.

Reaction solvent:

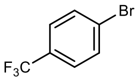

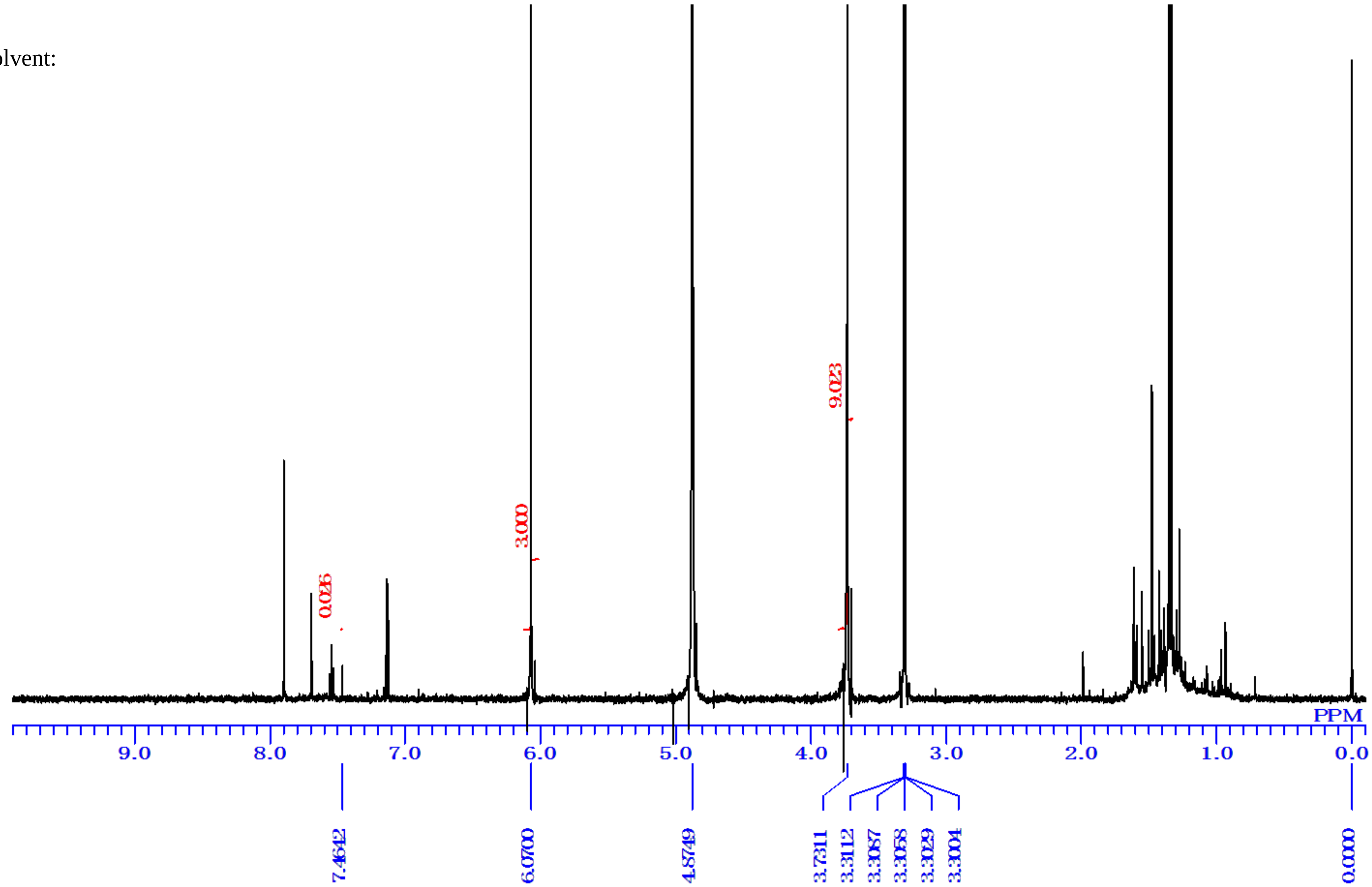

Supplementary Fig. 18 | ^{1}H NMR spectrum of the crude mixture (entry 7, 600 MHz, 20 °C, CD_3OD).

Entry 7, zoomed-in spectrum.

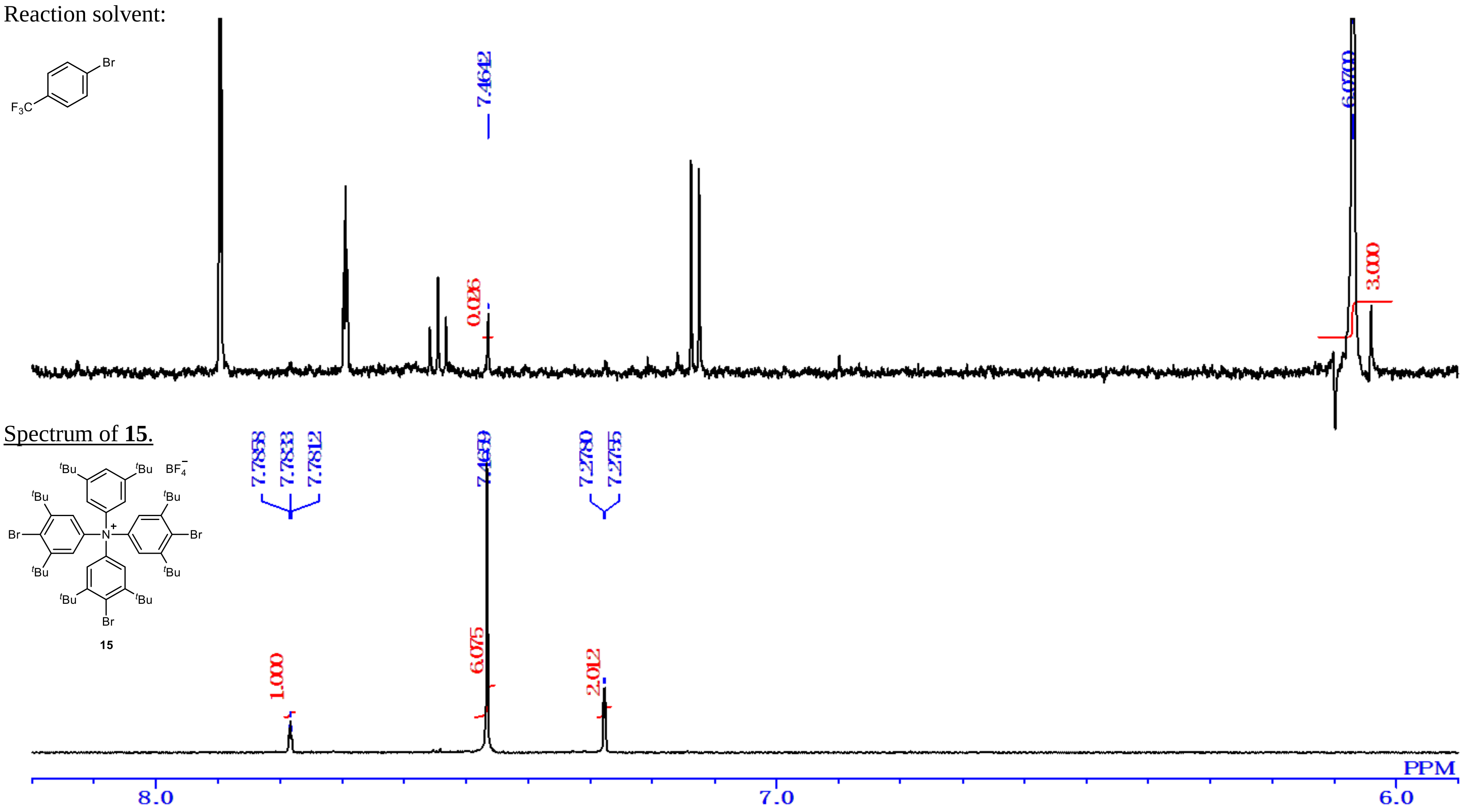

Supplementary Fig. 19 | Comparison of ^{1}H NMR spectra (600 MHz, 20 °C, CD_3OD) of the crude mixture (entry 7) and 15.

Reaction solvent:

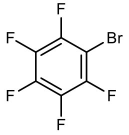

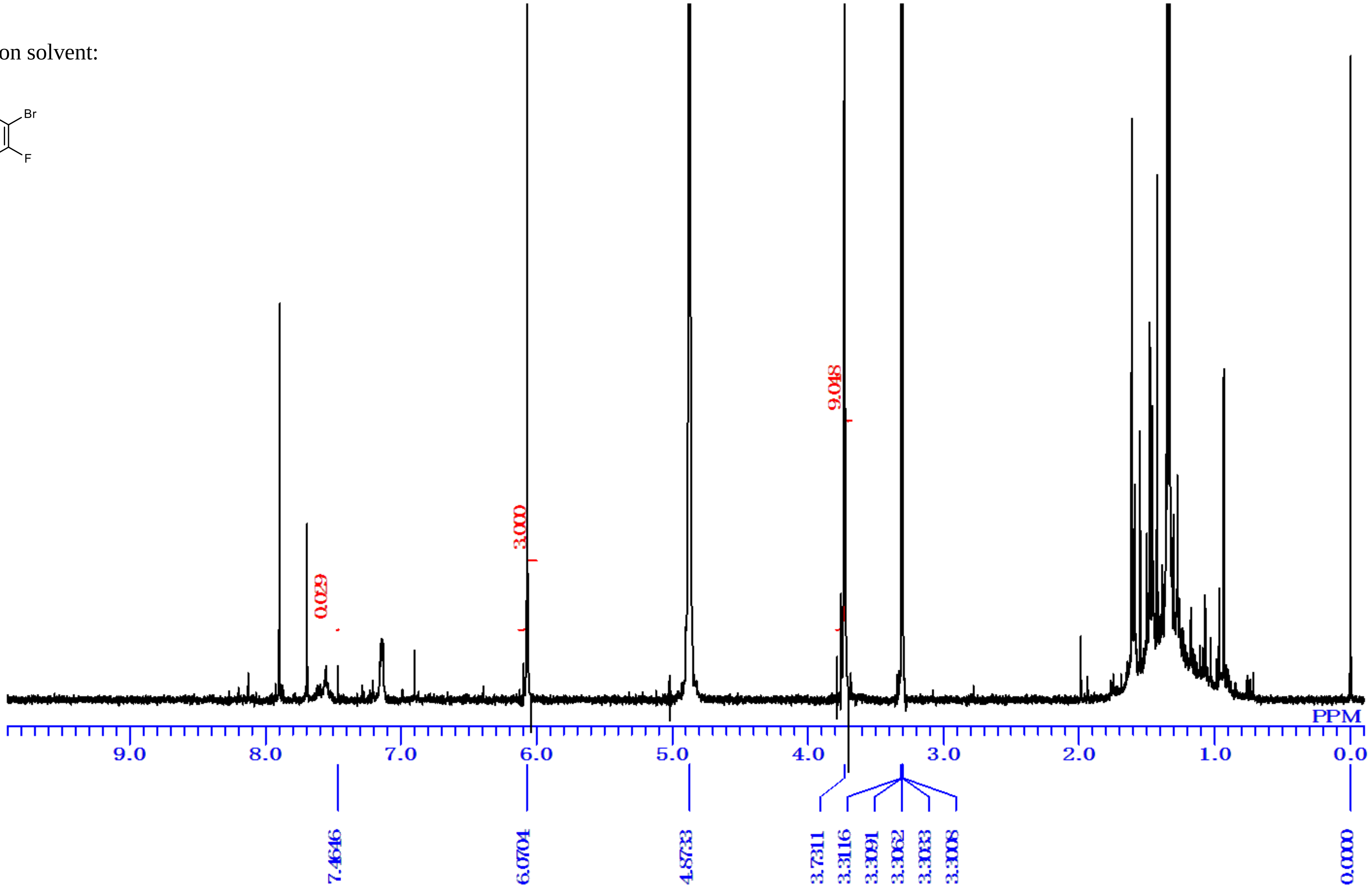

Supplementary Fig. 20 | ^{1}H NMR spectrum of the crude mixture (entry 8, 600 MHz, 20 °C, CD_3OD).

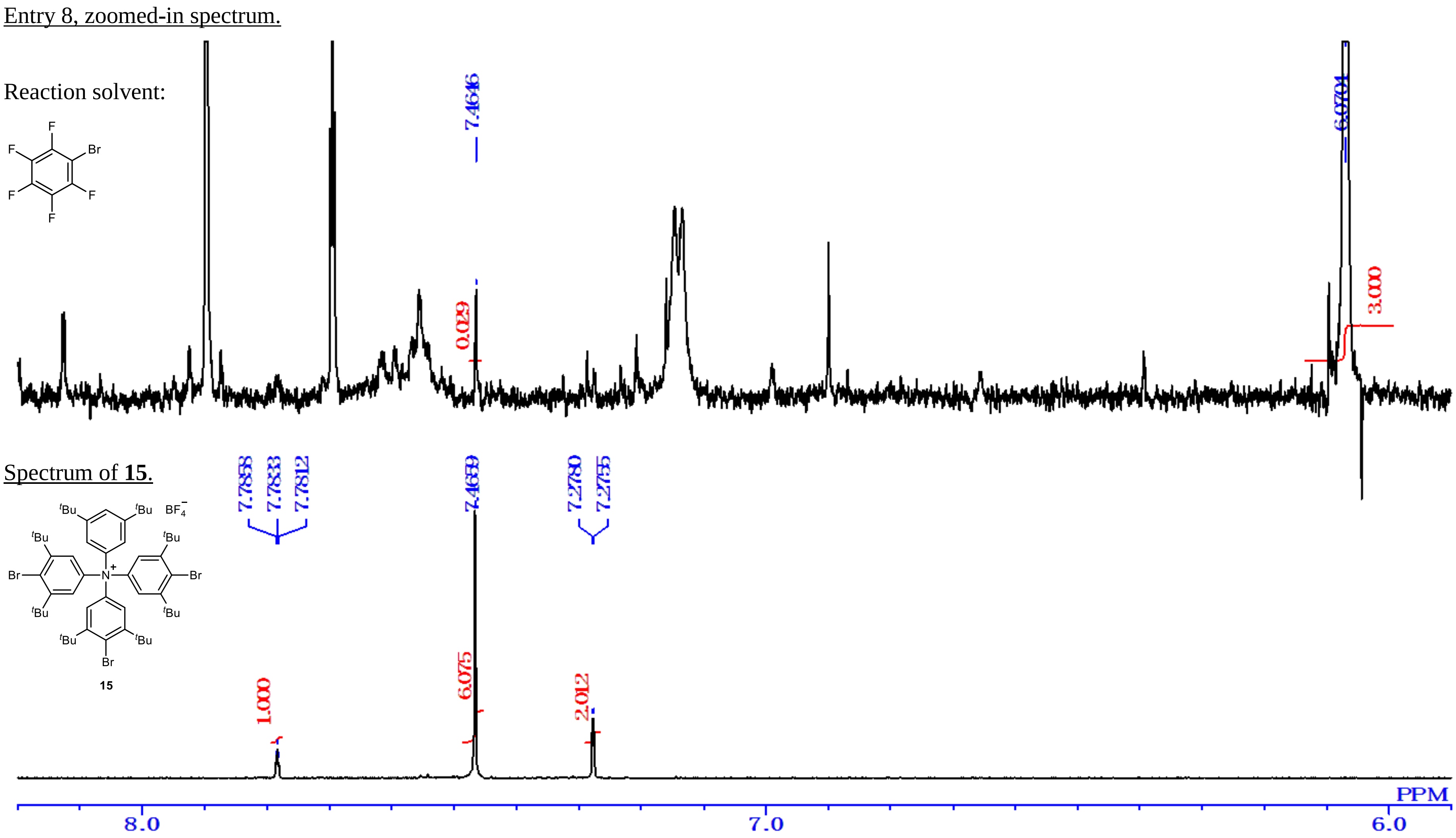

Supplementary Fig. 21 | Comparison of ^{1}H NMR spectra (600 MHz, 20 °C, CD_3OD) of the crude mixture (entry 8) and 15.

Reaction solvent:

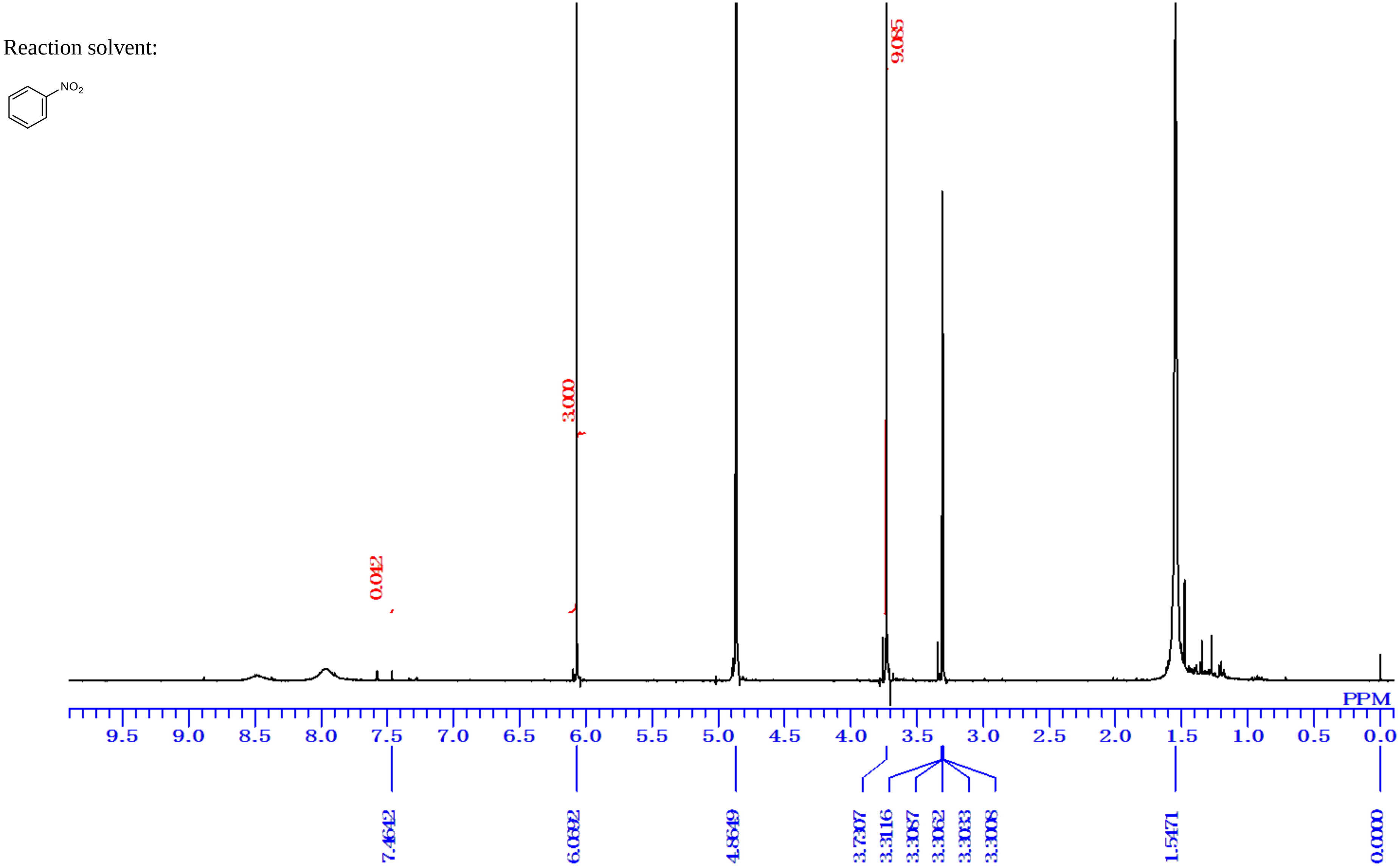

Supplementary Fig. 22 | ^{1}H NMR spectrum of the crude mixture (entry 9, 600 MHz, 20 °C, CD_3OD).

Entry 9, zoomed-in spectrum.

Reaction solvent:

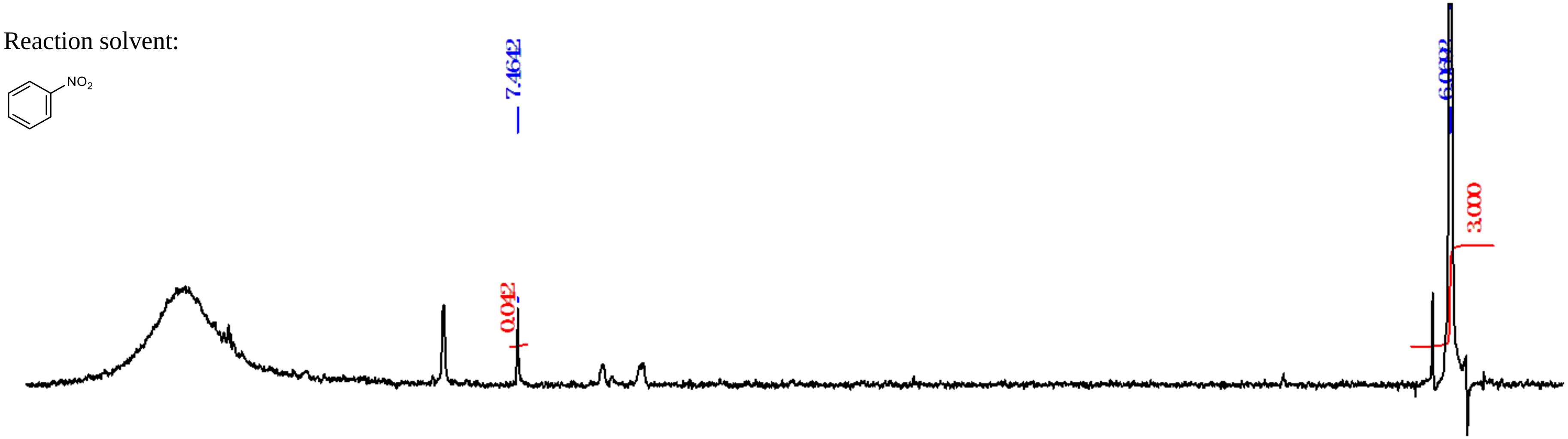

Spectrum of **15**.

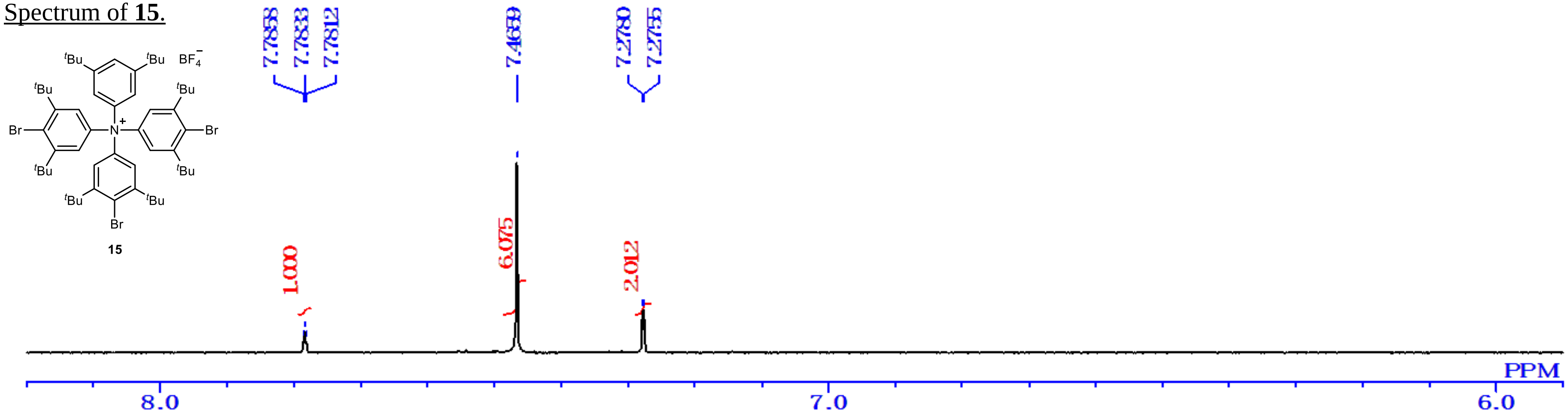

Supplementary Fig. 23 | Comparison of ^{1}H NMR spectra (600 MHz, 20 °C, CD_3OD) of the crude mixture (entry 9) and 15.

Reaction solvent:

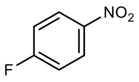

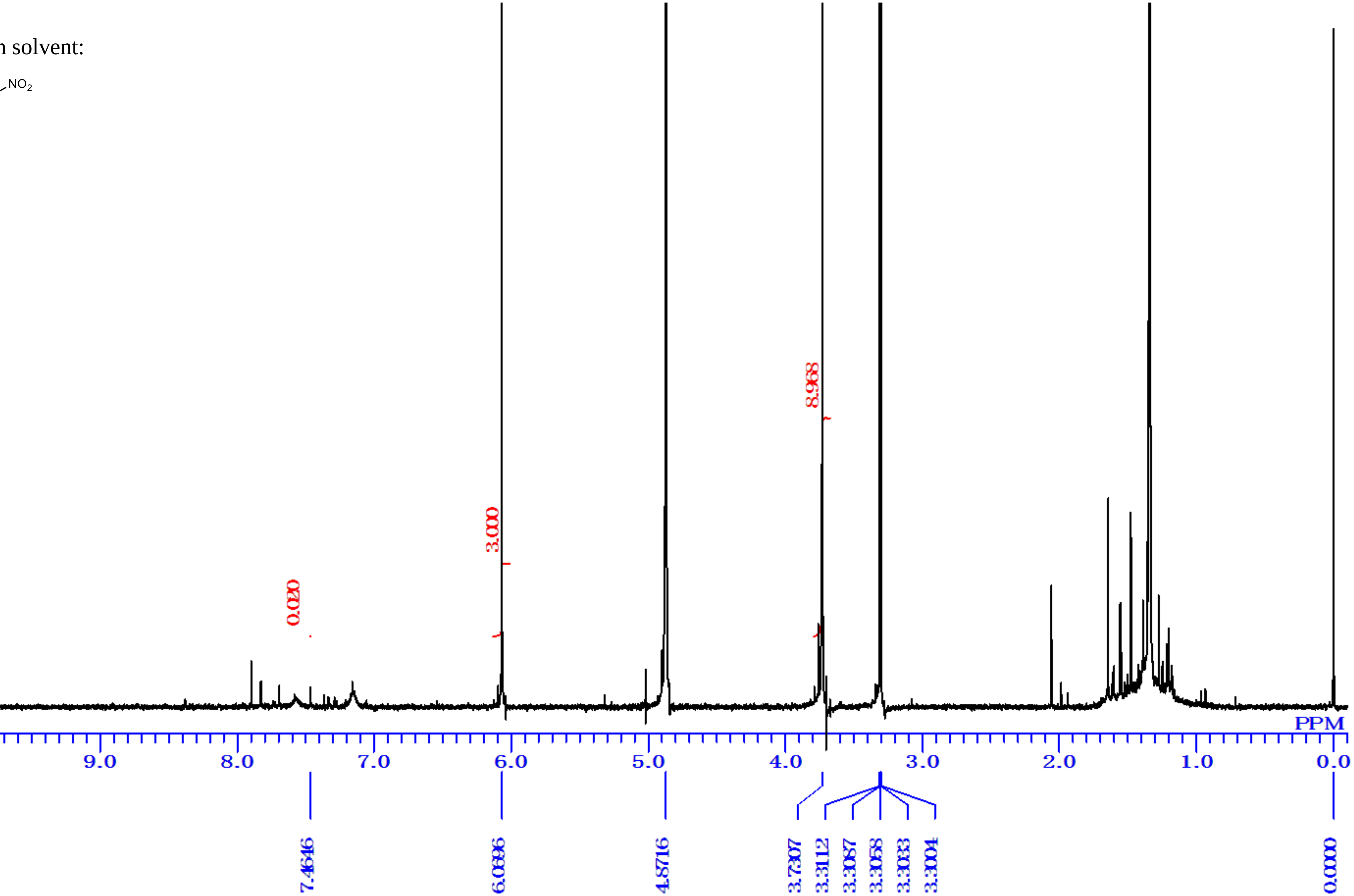

Supplementary Fig. 24 | ^{1}H NMR spectrum of the crude mixture (entry 10, 600 MHz, 20 °C, CD_3OD).

Entry 10, zoomed-in spectrum.

Reaction solvent:

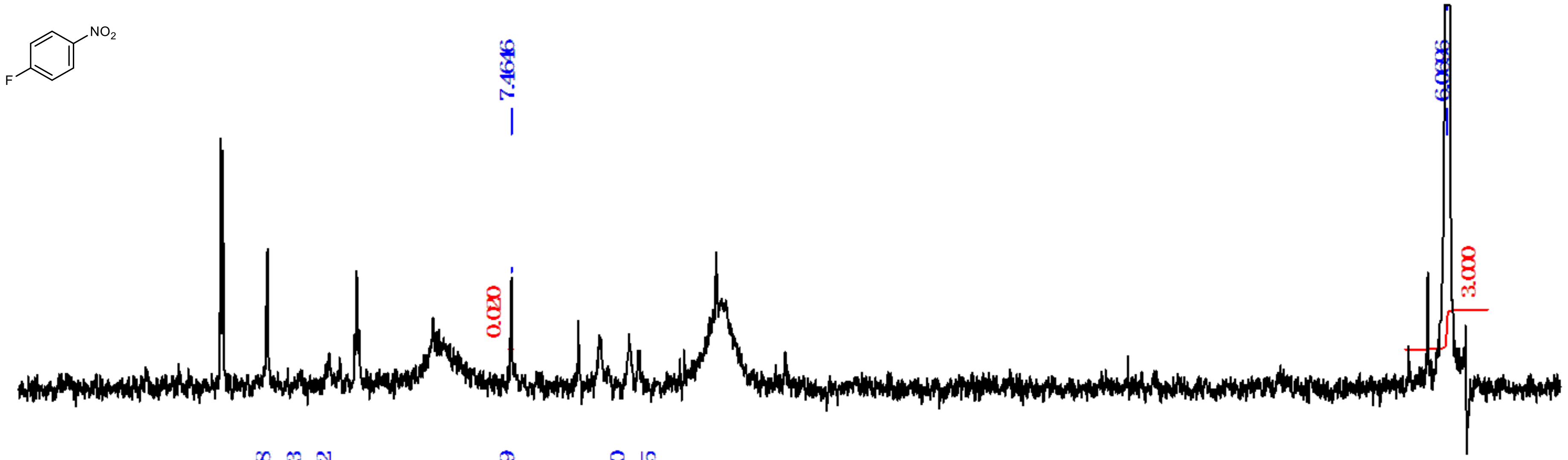

Spectrum of **15**.

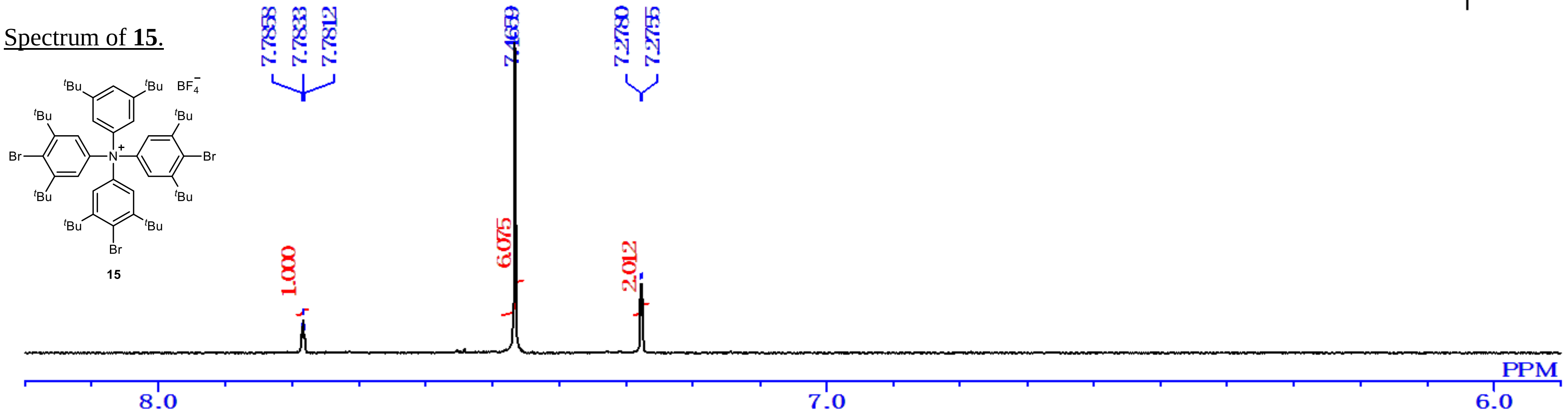

Supplementary Fig. 25 | Comparison of ^{1}H NMR spectra (600 MHz, 20 °C, CD_3OD) of the crude mixture (entry 10) and 15.

Reaction solvent:

DMF

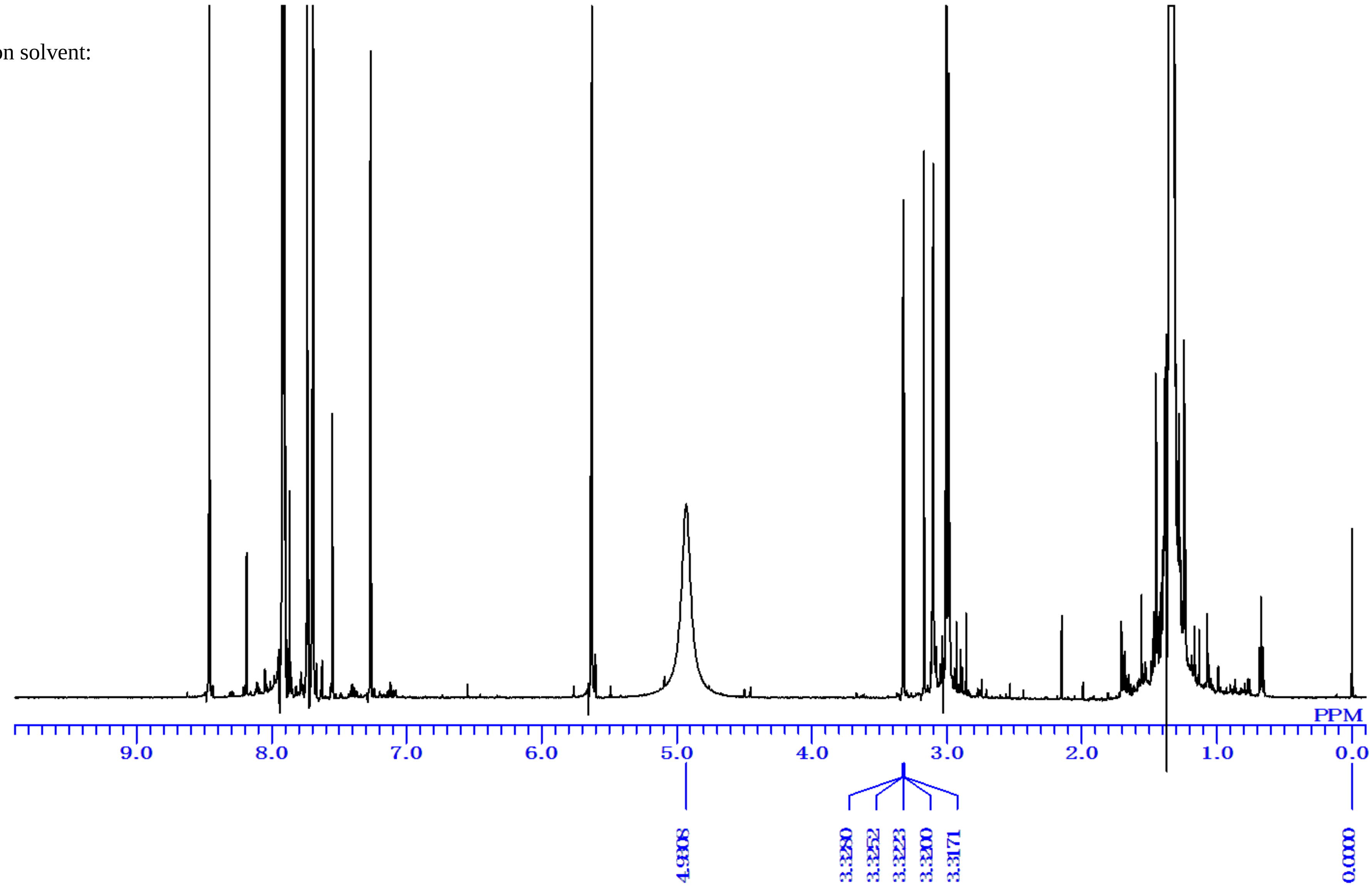

Supplementary Fig. 26 | ^{1}H NMR spectrum of the crude mixture (entry 11, 600 MHz, 20 °C, CD_3OD).

Entry 11, zoomed-in spectrum.

Reaction solvent:

DMF

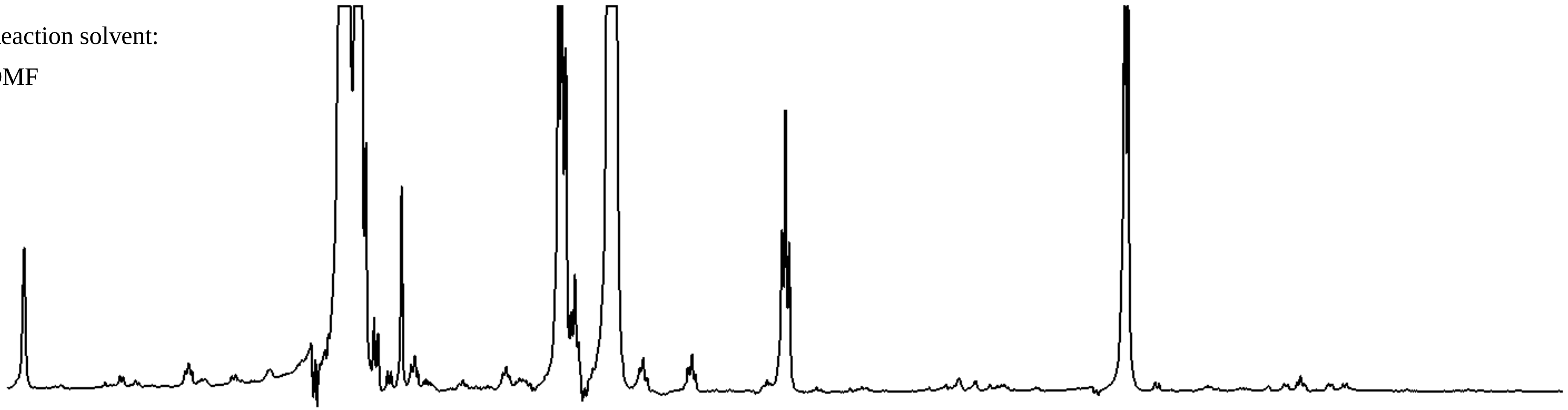

Spectrum of **15**.

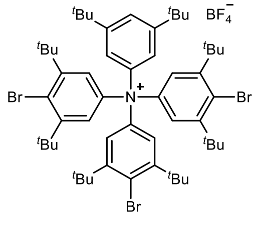

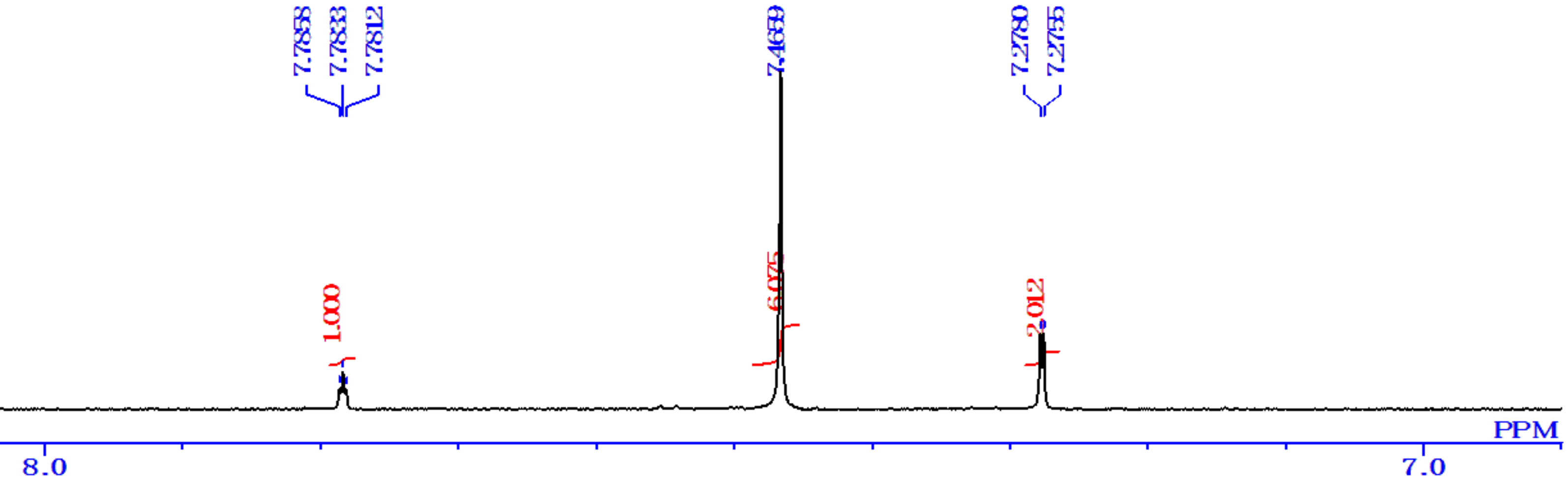

Supplementary Fig. 27 | Comparison of ^{1}H NMR spectra (600 MHz, 20 °C, CD_3OD) of the crude mixture (entry 11) and 15.

Reaction solvent:

$MeNO_2$

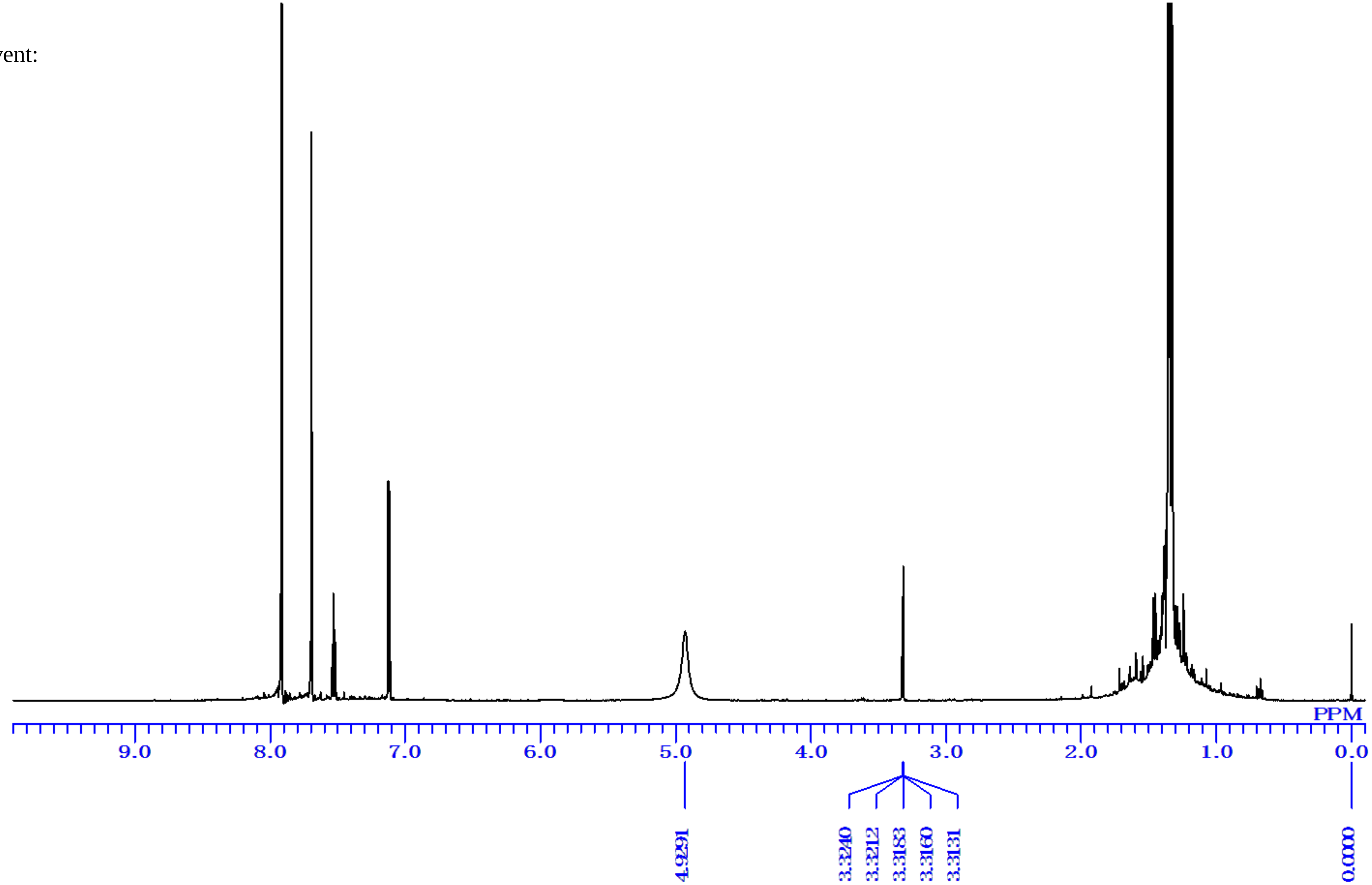

Supplementary Fig. 28 | ^{1}H NMR spectrum of the crude mixture (entry 12, 600 MHz, 20 °C, CD_3OD).

Entry 12, zoomed-in spectrum.

Reaction solvent:

$MeNO_2$

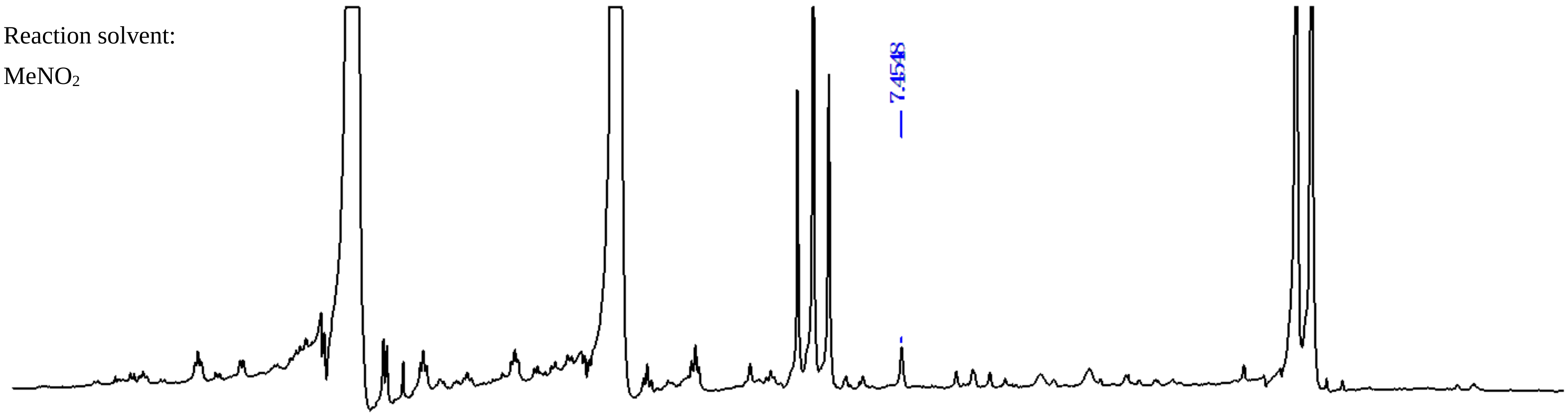

Spectrum of **15**.

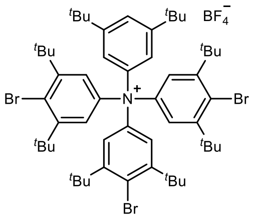

15

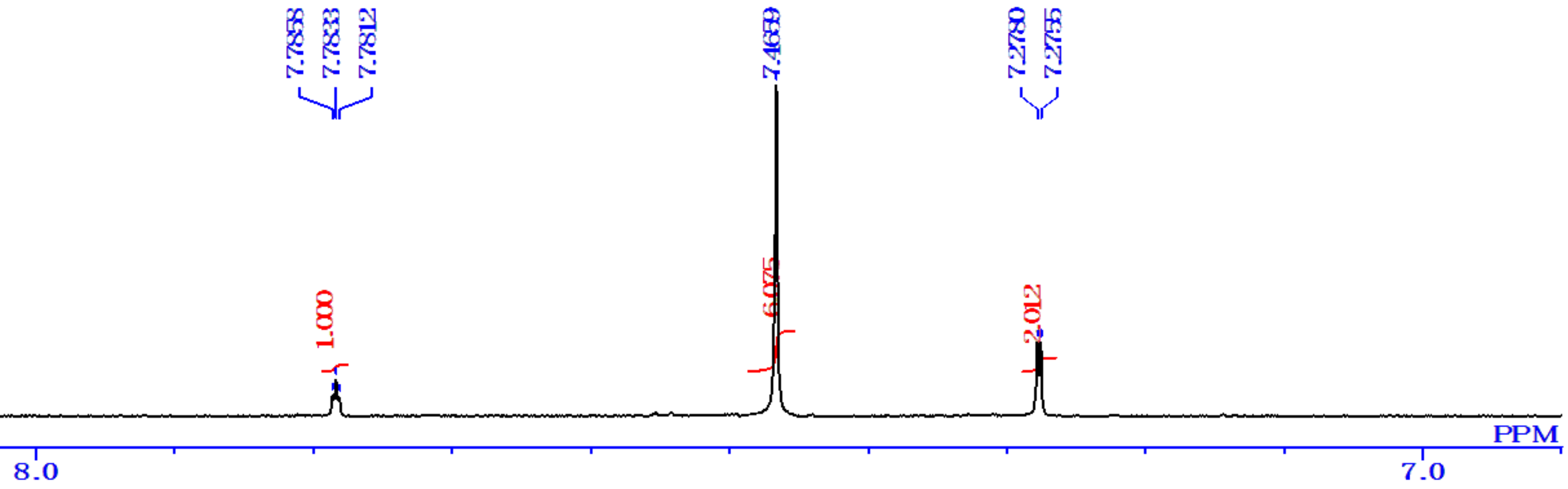

Supplementary Fig. 29 | Comparison of ^{1}H NMR spectra (600 MHz, 20 °C, CD_3OD) of the crude mixture (entry 12) and 15.

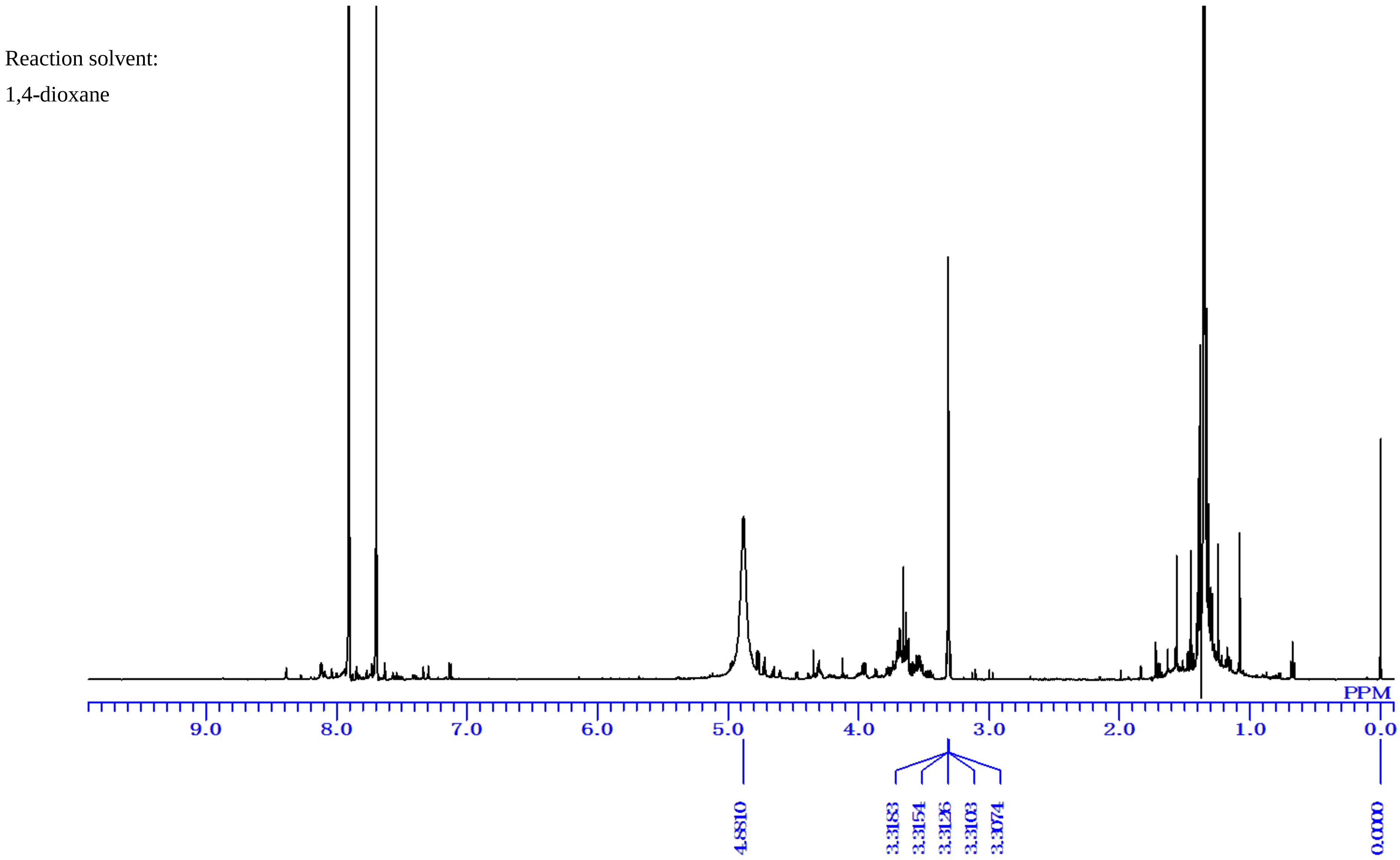

Supplementary Fig. 30 | ^{1}H NMR spectrum of the crude mixture (entry 13, 600 MHz, 20 °C, CD_3OD).

Entry 13, zoomed-in spectrum.

Reaction solvent:

1,4-dioxane

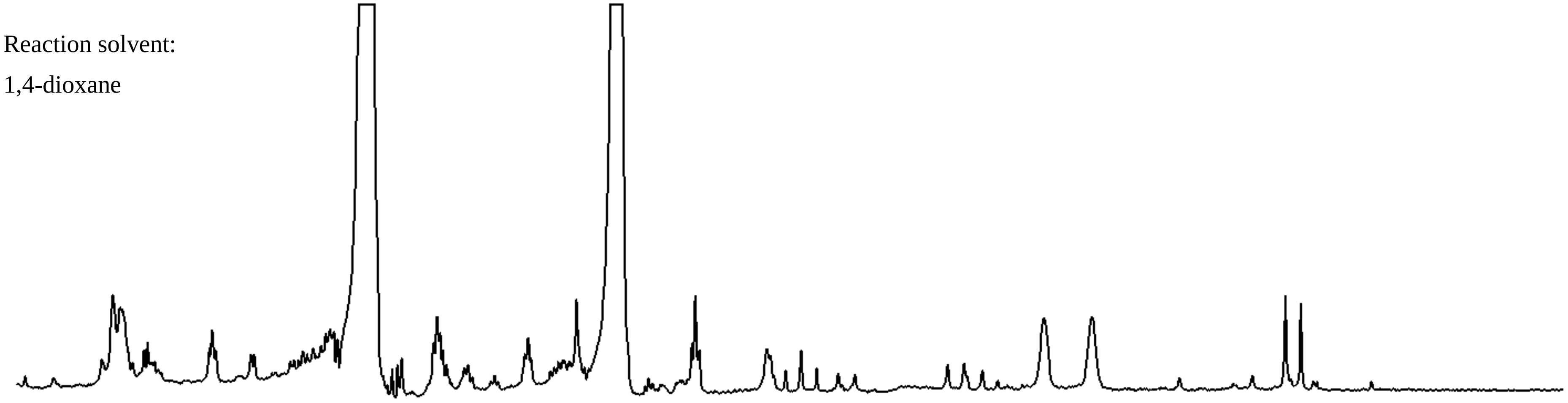

Spectrum of **15**.

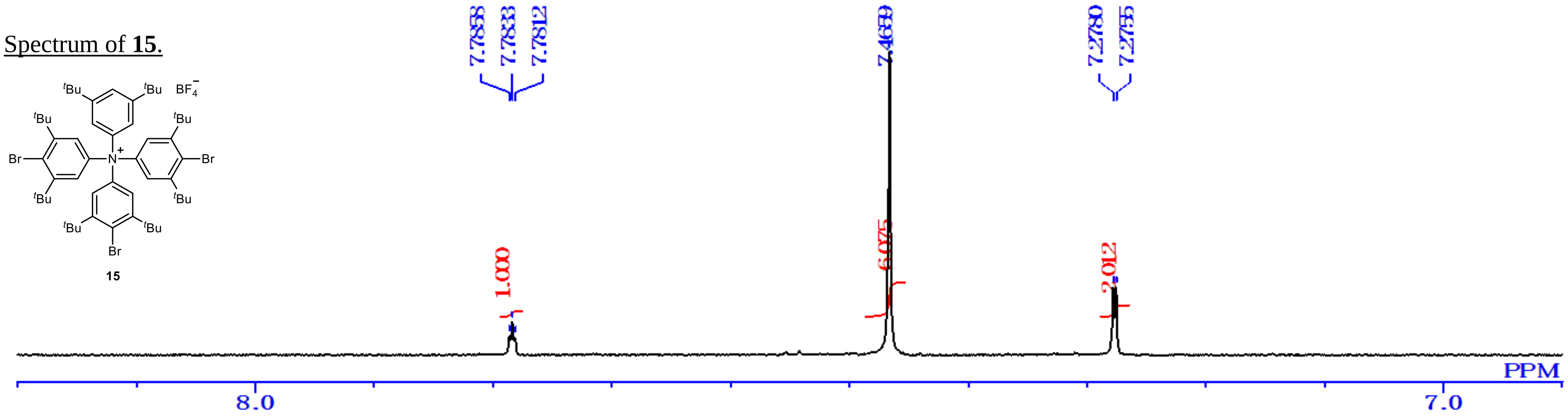

Supplementary Fig. 31 | Comparison of ^{1}H NMR spectra (600 MHz, 20 °C, CD_3OD) of the crude mixture (entry 13) and 15.

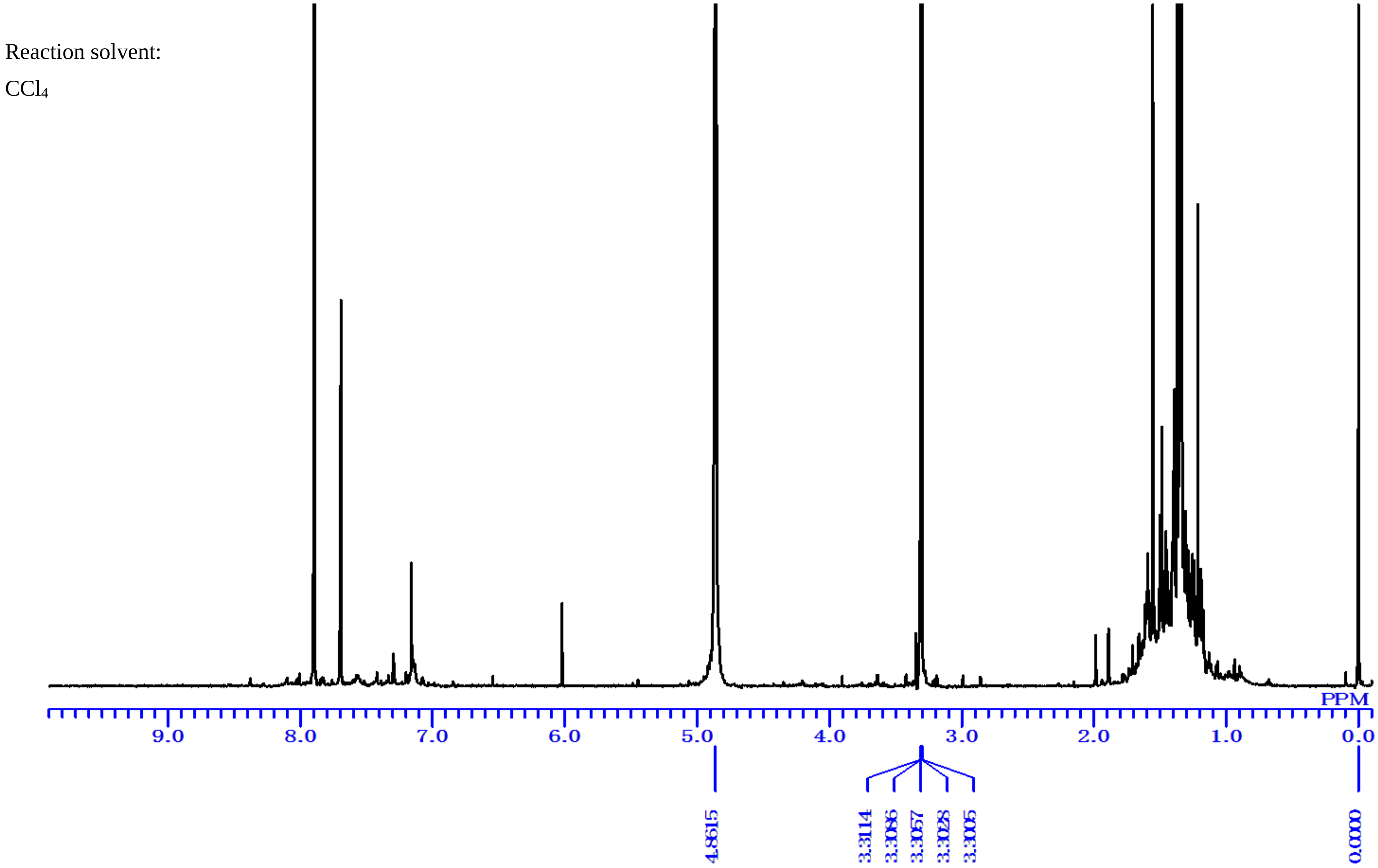

Supplementary Fig. 32 | ^{1}H NMR spectrum of the crude mixture (entry 14, 600 MHz, 20 °C, CD_3OD).

Entry 14, zoomed-in spectrum.

Reaction solvent:

CCl_4

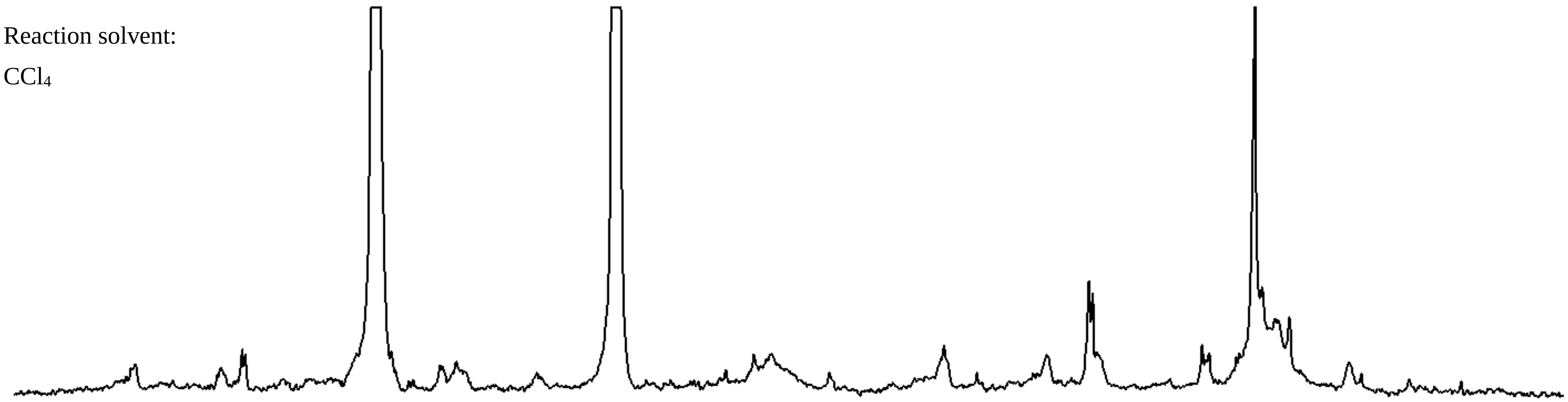

Spectrum of **15**.

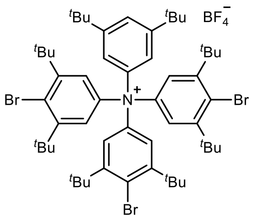

15

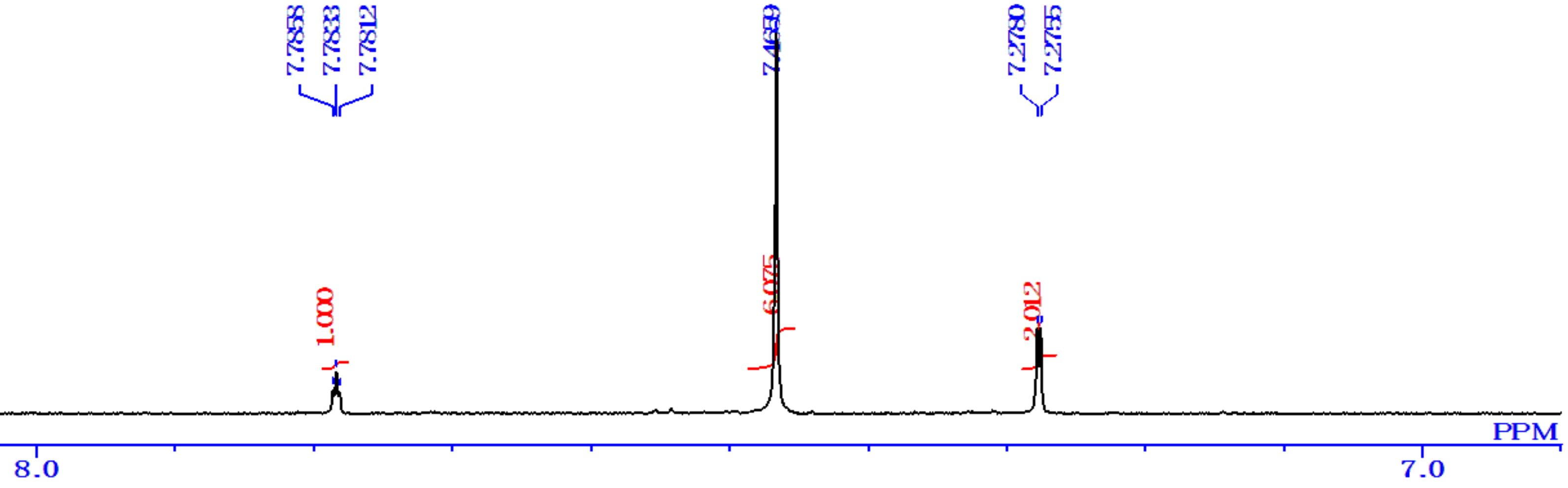

Supplementary Fig. 33 | Comparison of ^{1}H NMR spectra (600 MHz, 20 °C, CD_3OD) of the crude mixture (entry 14) and 15.

Reaction solvent:
MeCN

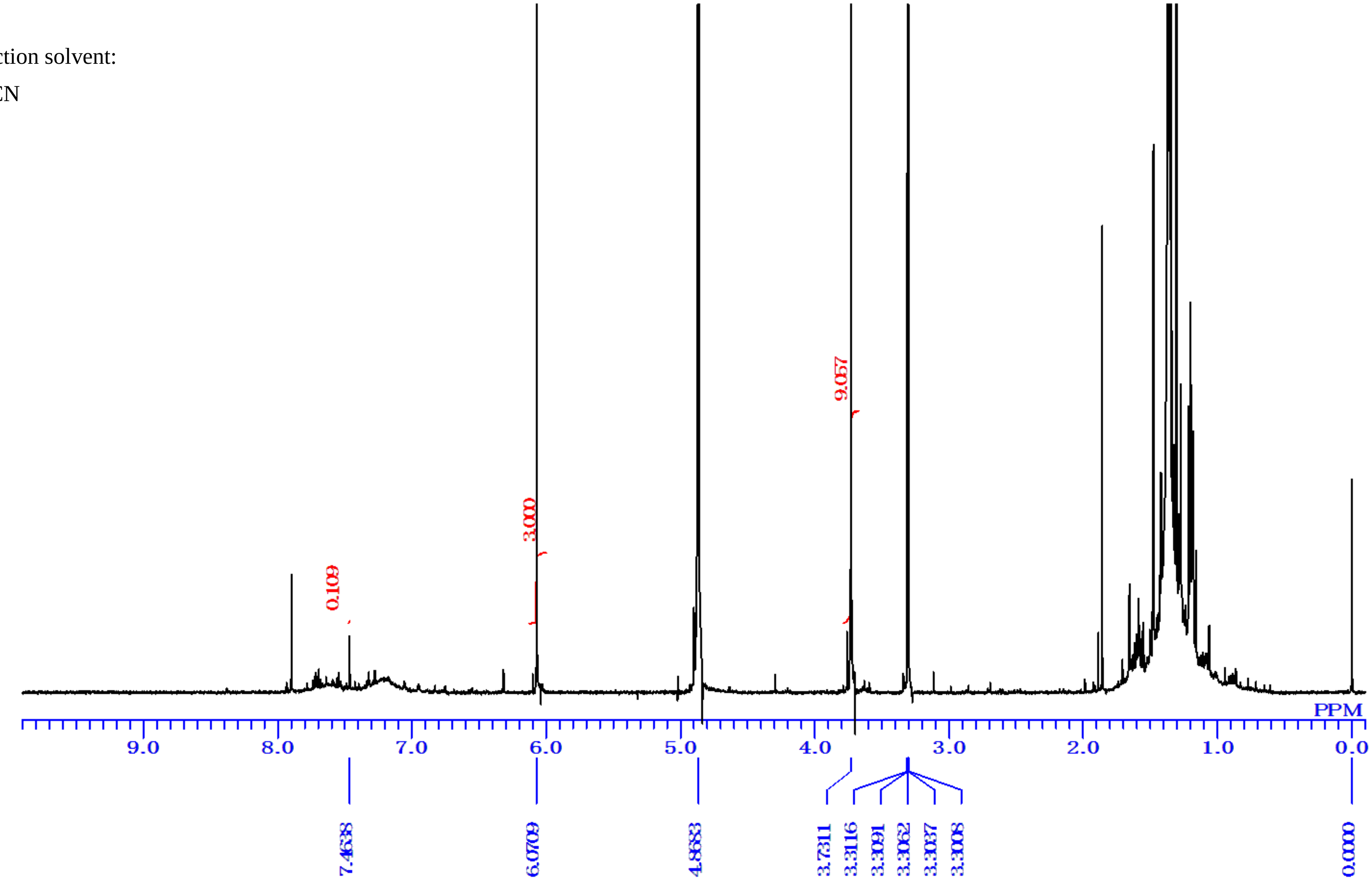

Supplementary Fig. 34 | ^{1}H NMR spectrum of the crude mixture (entry 15, 600 MHz, 20 °C, CD_3OD).

Entry 15, zoomed-in spectrum.

Reaction solvent:

MeCN

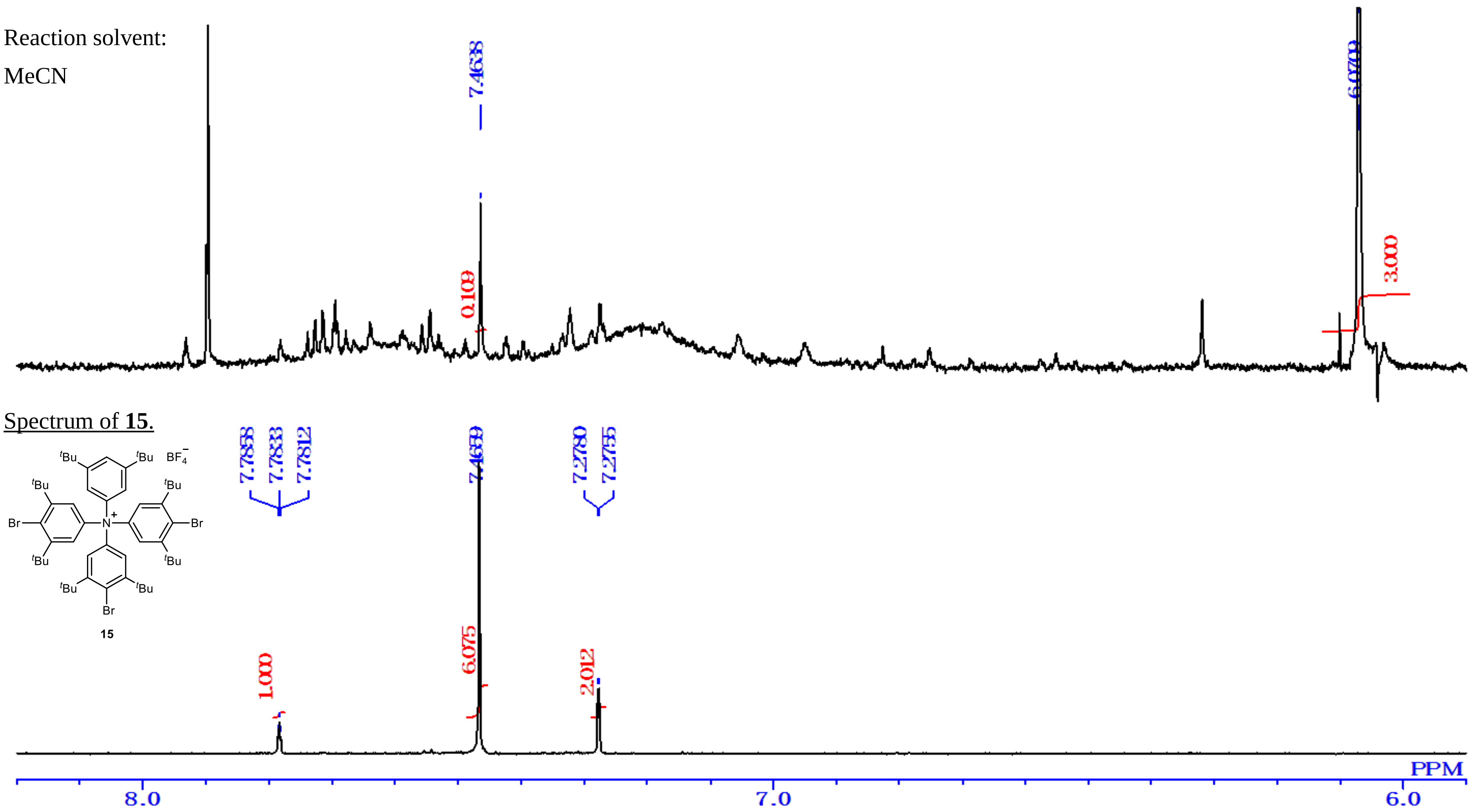

Supplementary Fig. 35 | Comparison of ^{1}H NMR spectra (600 MHz, 20 °C, CD_3OD) of the crude mixture (entry 15) and 15.

Reaction solvent:

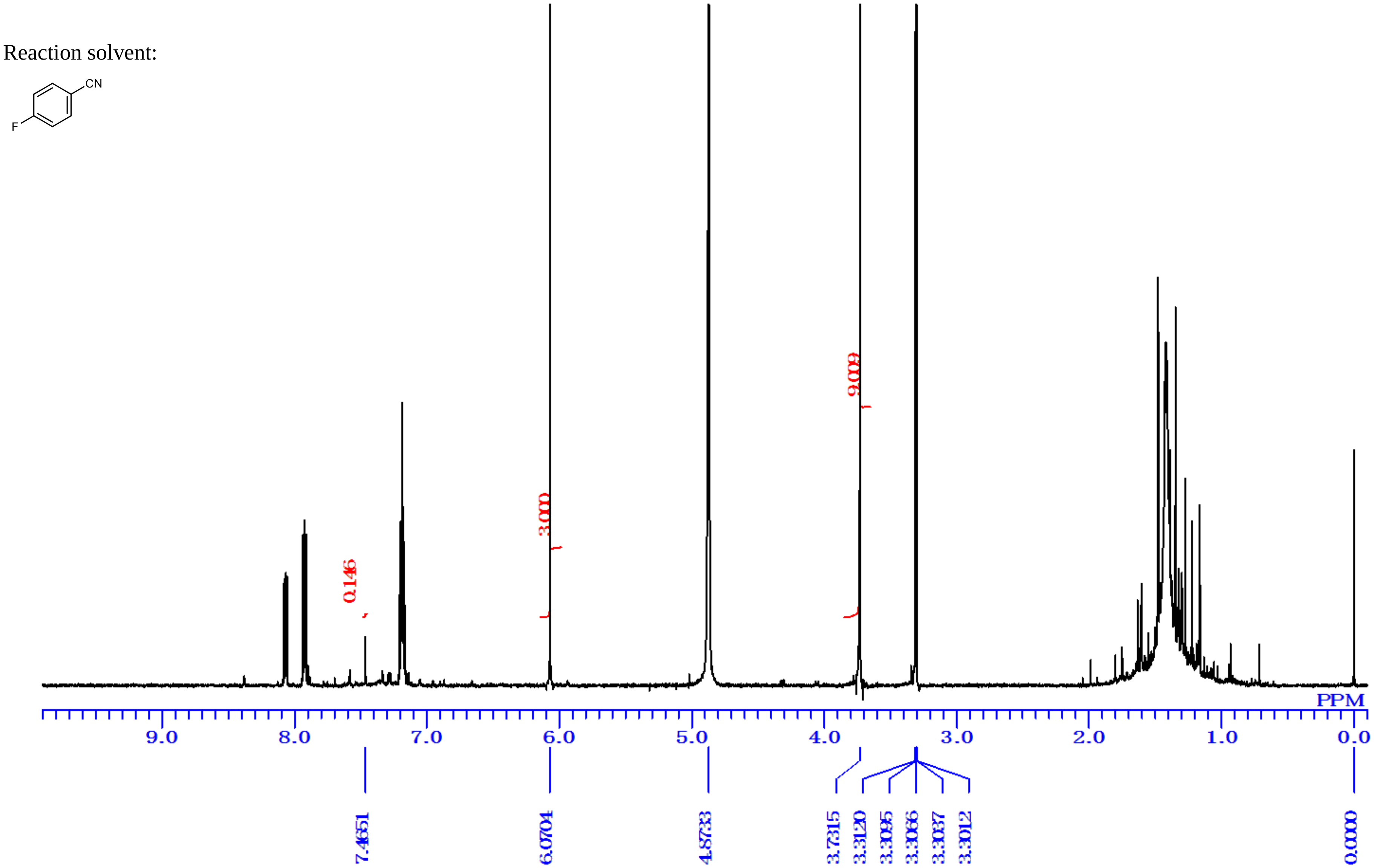

Supplementary Fig. 36 | ^{1}H NMR spectrum of the crude mixture (entry 16, 600 MHz, 20 °C, CD_3OD).

Entry 16, zoomed-in spectrum.

Reaction solvent:

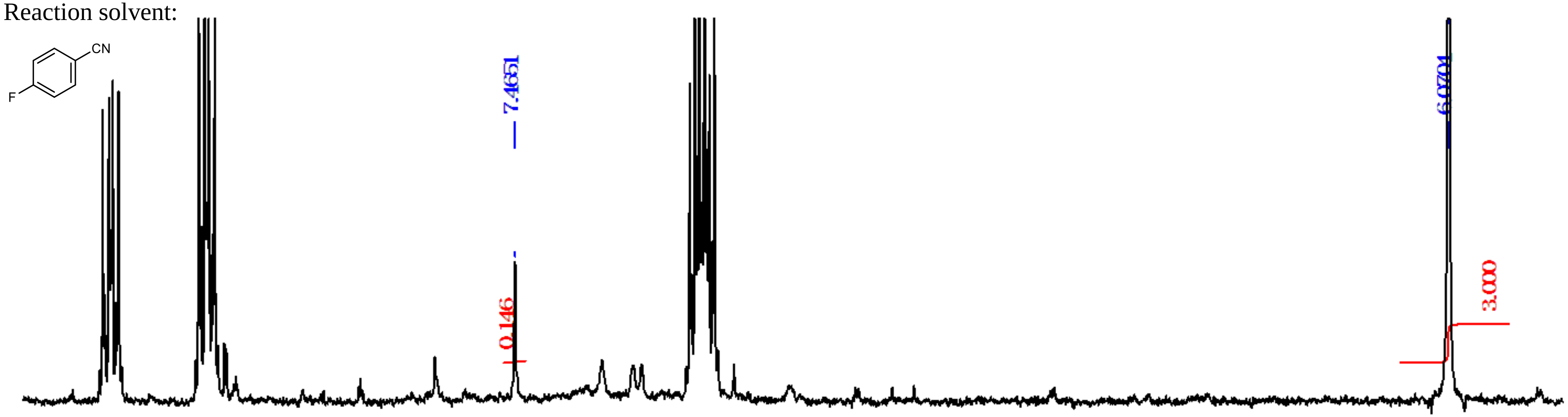

Spectrum of **15**.

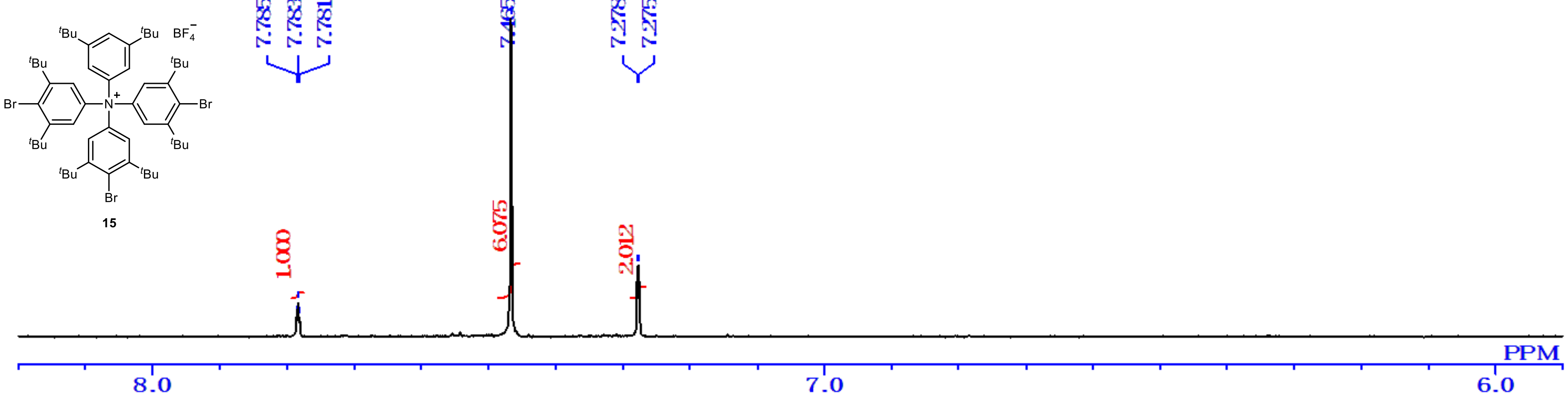

Supplementary Fig. 37 | Comparison of ^{1}H NMR spectra (600 MHz, 20 °C, CD_3OD) of the crude mixture (entry 16) and 15.

Reaction solvent:

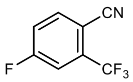

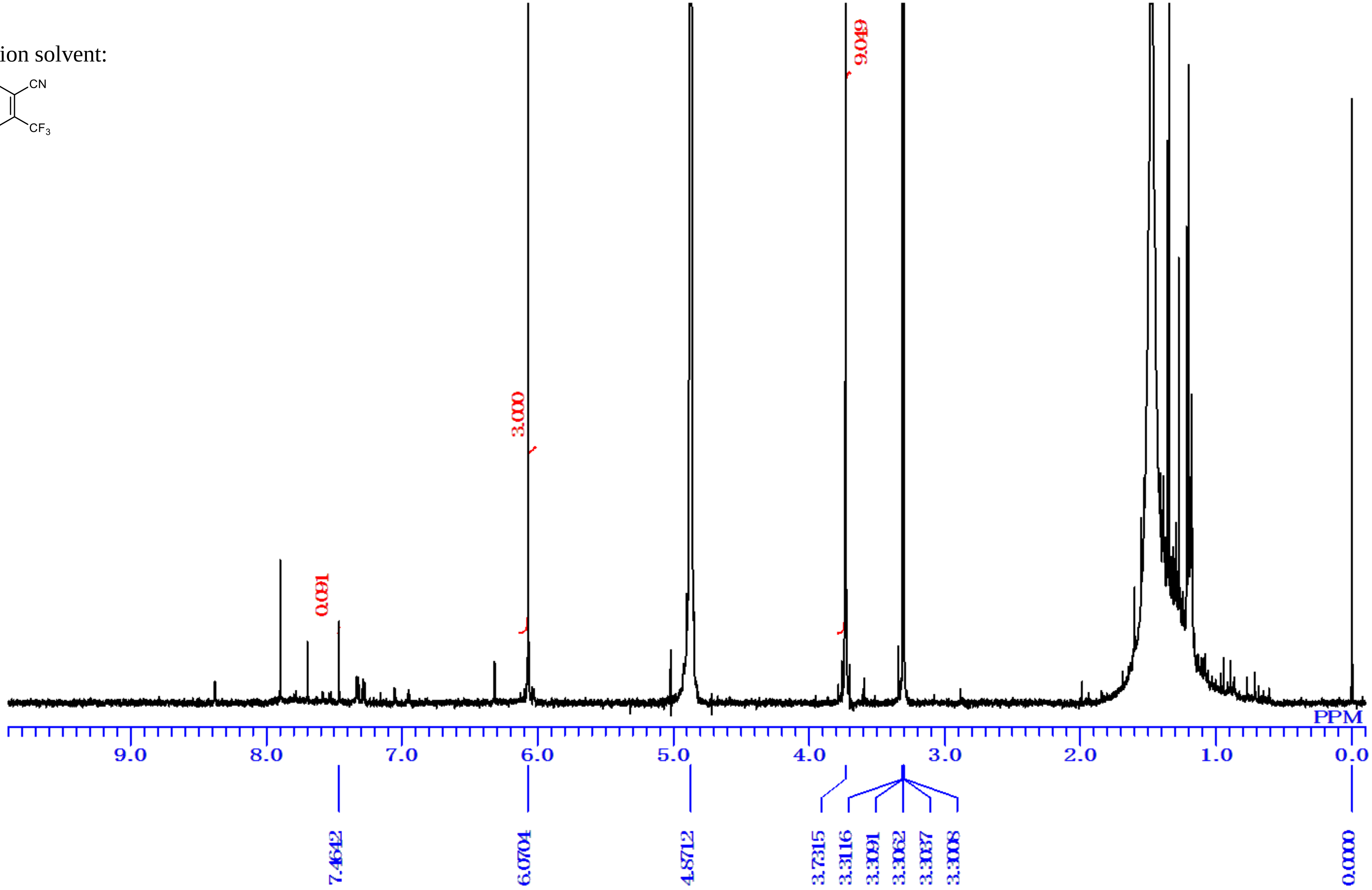

Supplementary Fig. 38 | ^{1}H NMR spectrum of the crude mixture (entry 17, 600 MHz, 20 °C, CD_3OD).

Entry 17, zoomed-in spectrum.

Reaction solvent:

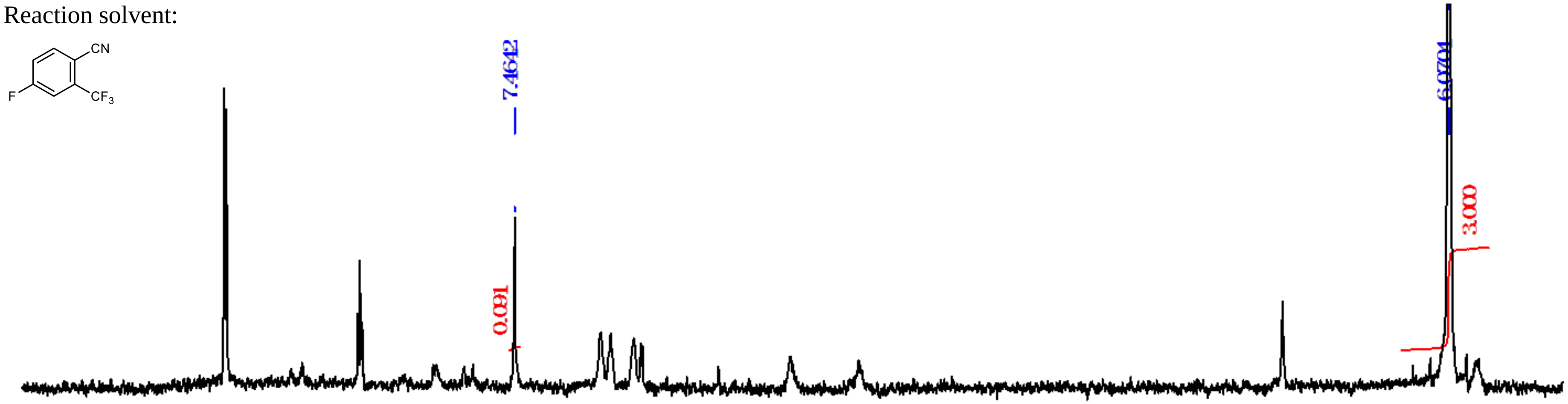

Spectrum of **15**.

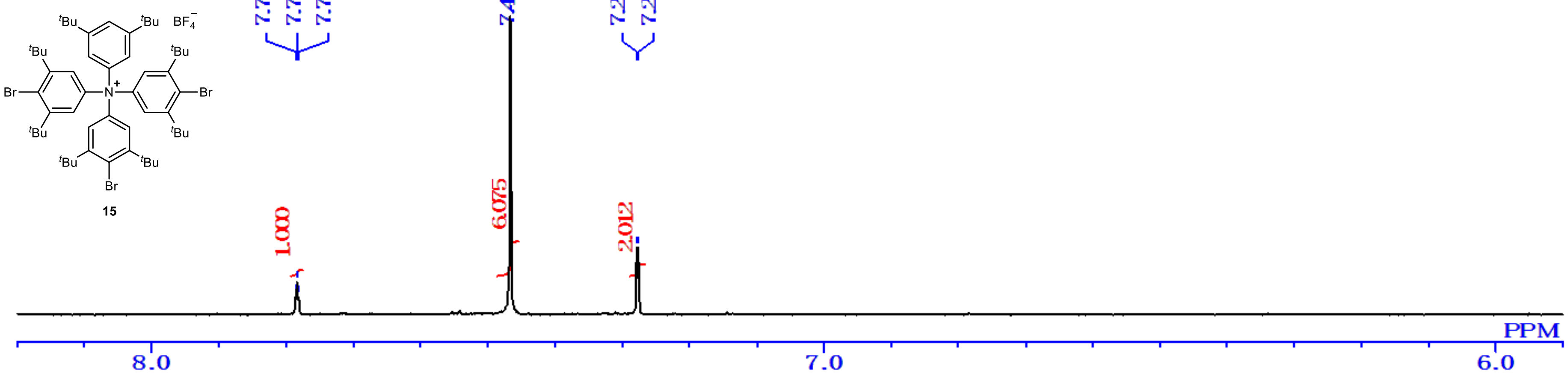

Supplementary Fig. 39 | Comparison of ^{1}H NMR spectra (600 MHz, 20 °C, CD_3OD) of the crude mixture (entry 17) and 15.

Reaction solvent:

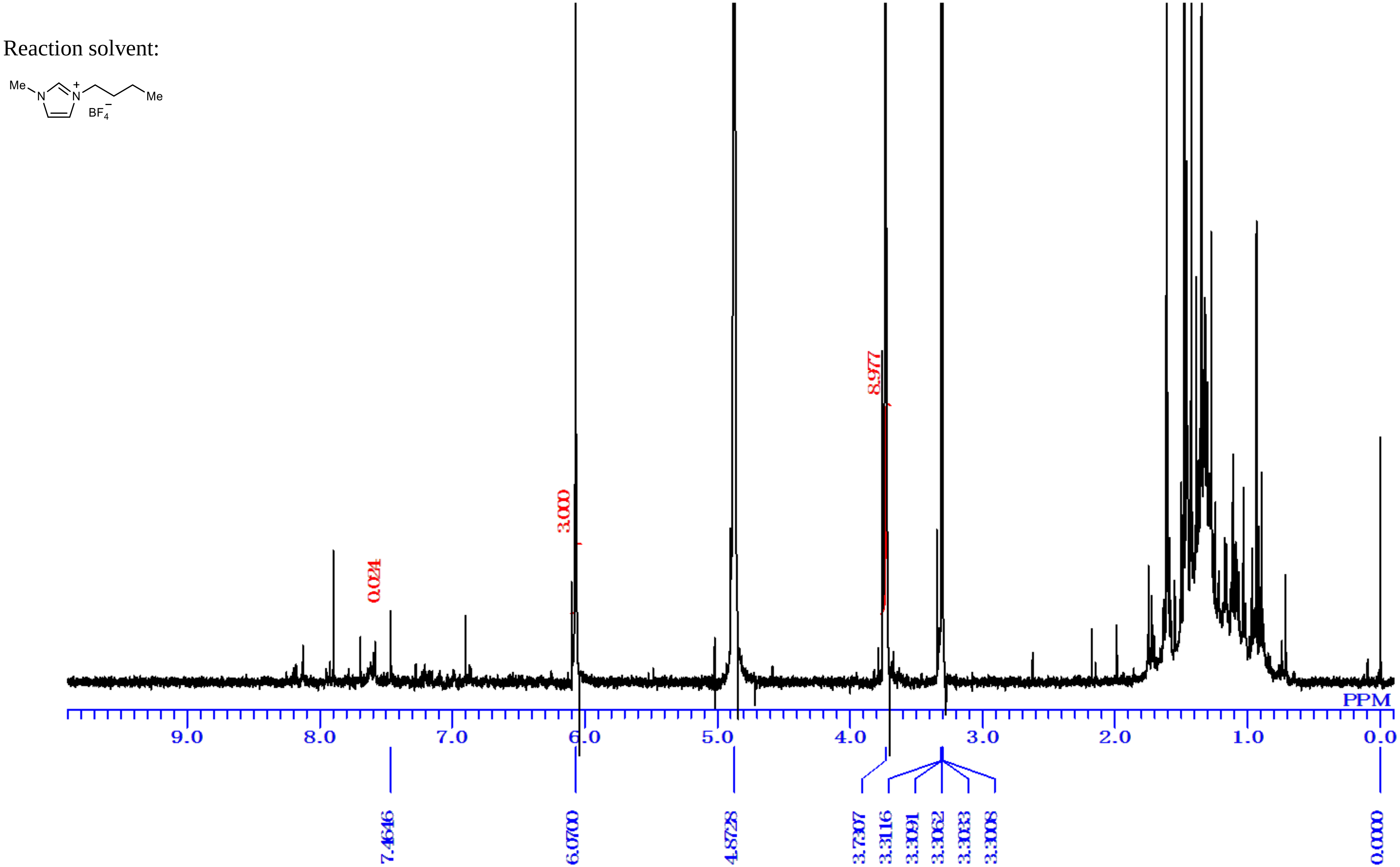

Supplementary Fig. 40 | ^{1}H NMR spectrum of the crude mixture (entry 18, 600 MHz, 20 °C, CD_3OD).

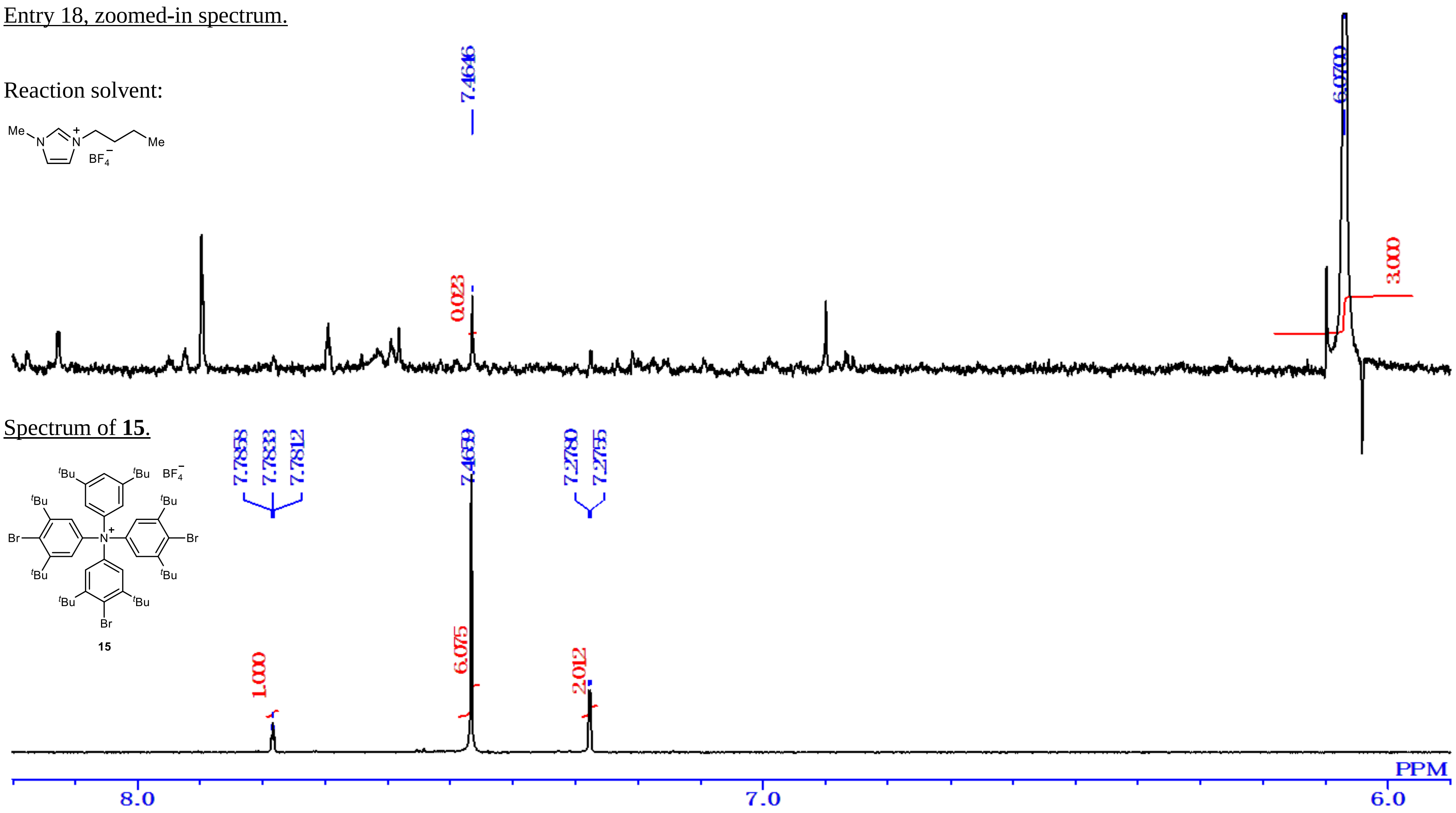

Supplementary Fig. 41 | Comparison of ^{1}H NMR spectra (600 MHz, 20 °C, CD_3OD) of the crude mixture (entry 18) and 15.

Reaction solvent:
1,2-dichloroethane

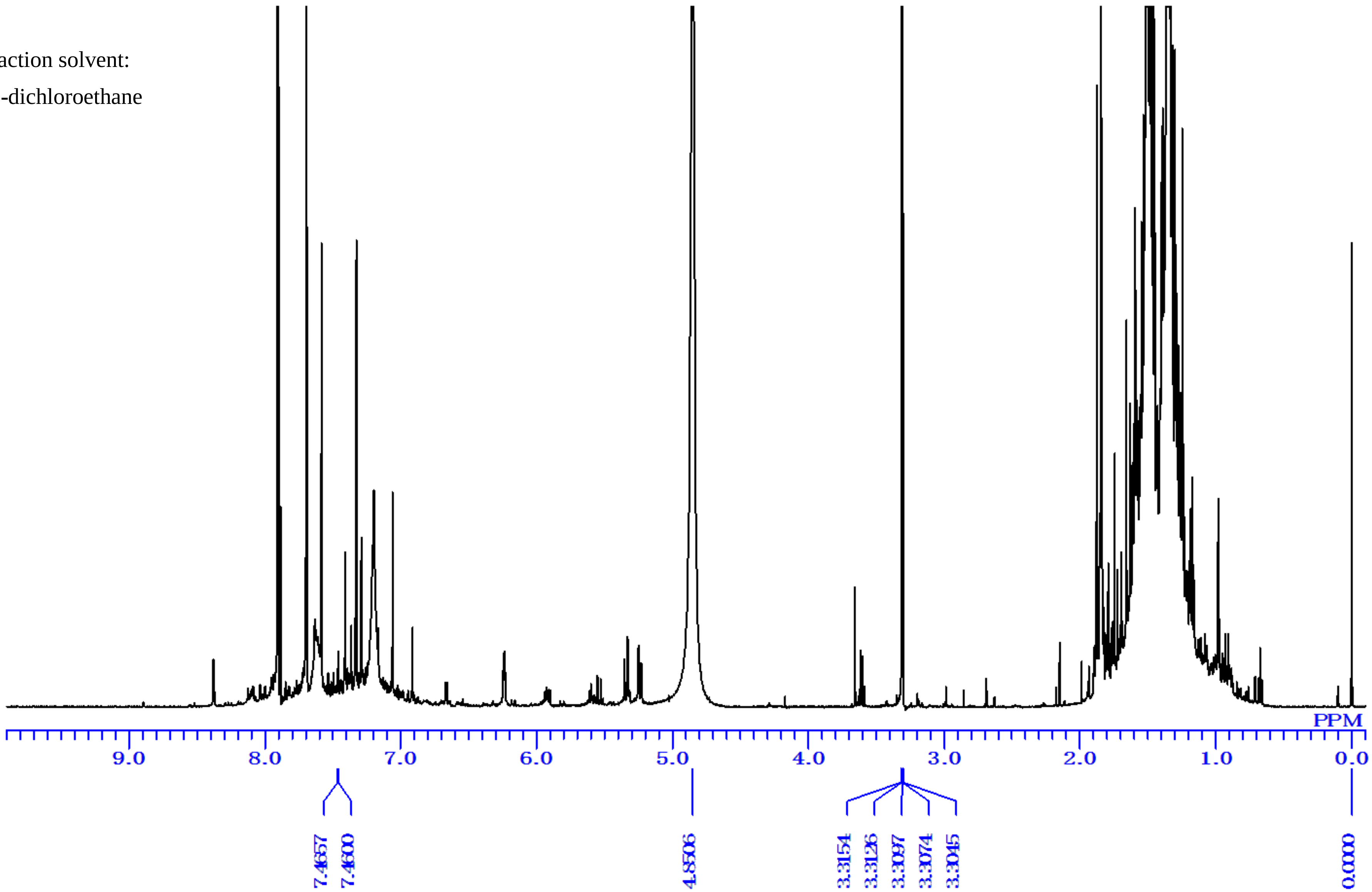

Supplementary Fig. 42 | ^{1}H NMR spectrum of the crude mixture (entry 19, 600 MHz, 20 °C, CD_3OD).

Entry 19, zoomed-in spectrum.

Reaction solvent:

1,2-dichloroethane

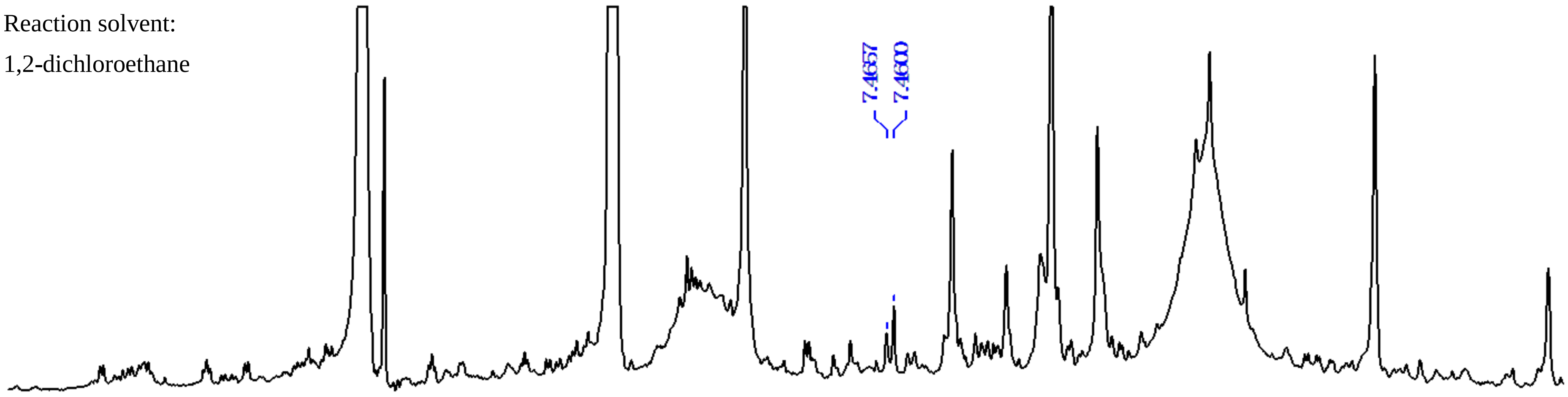

Spectrum of **15**.

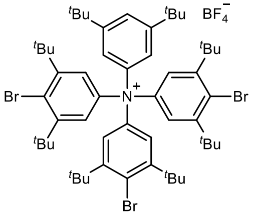

15

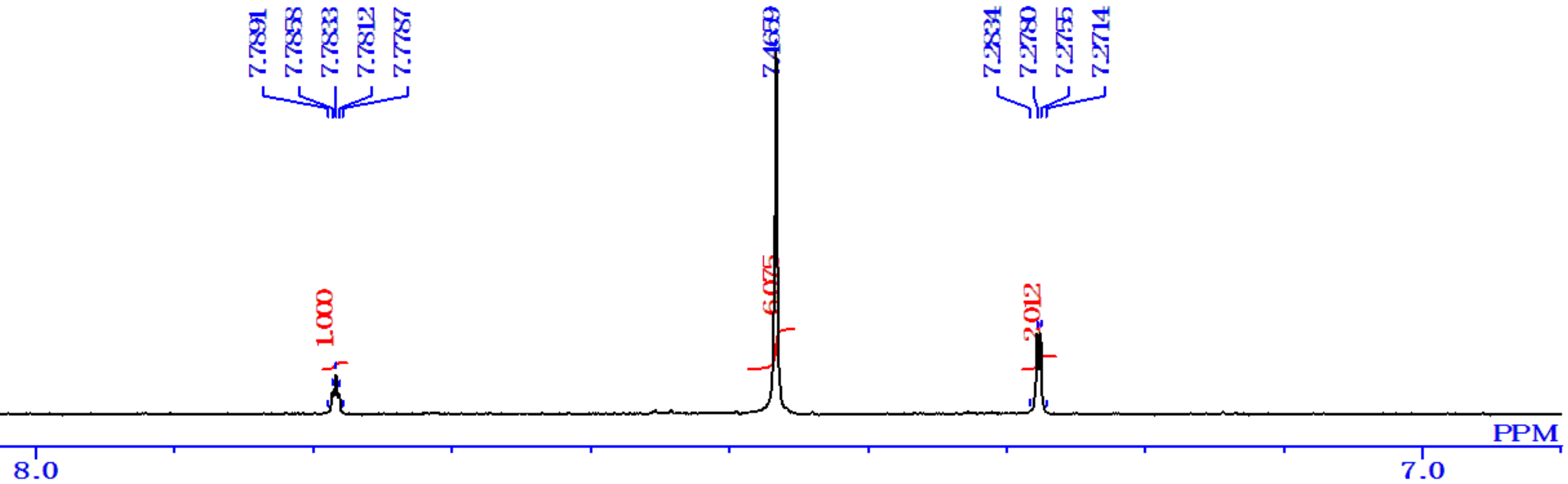

Supplementary Fig. 43 | Comparison of ^{1}H NMR spectra (600 MHz, 20 °C, CD_3OD) of the crude mixture (entry 19) and 15.

Reaction solvent:
acetic acid

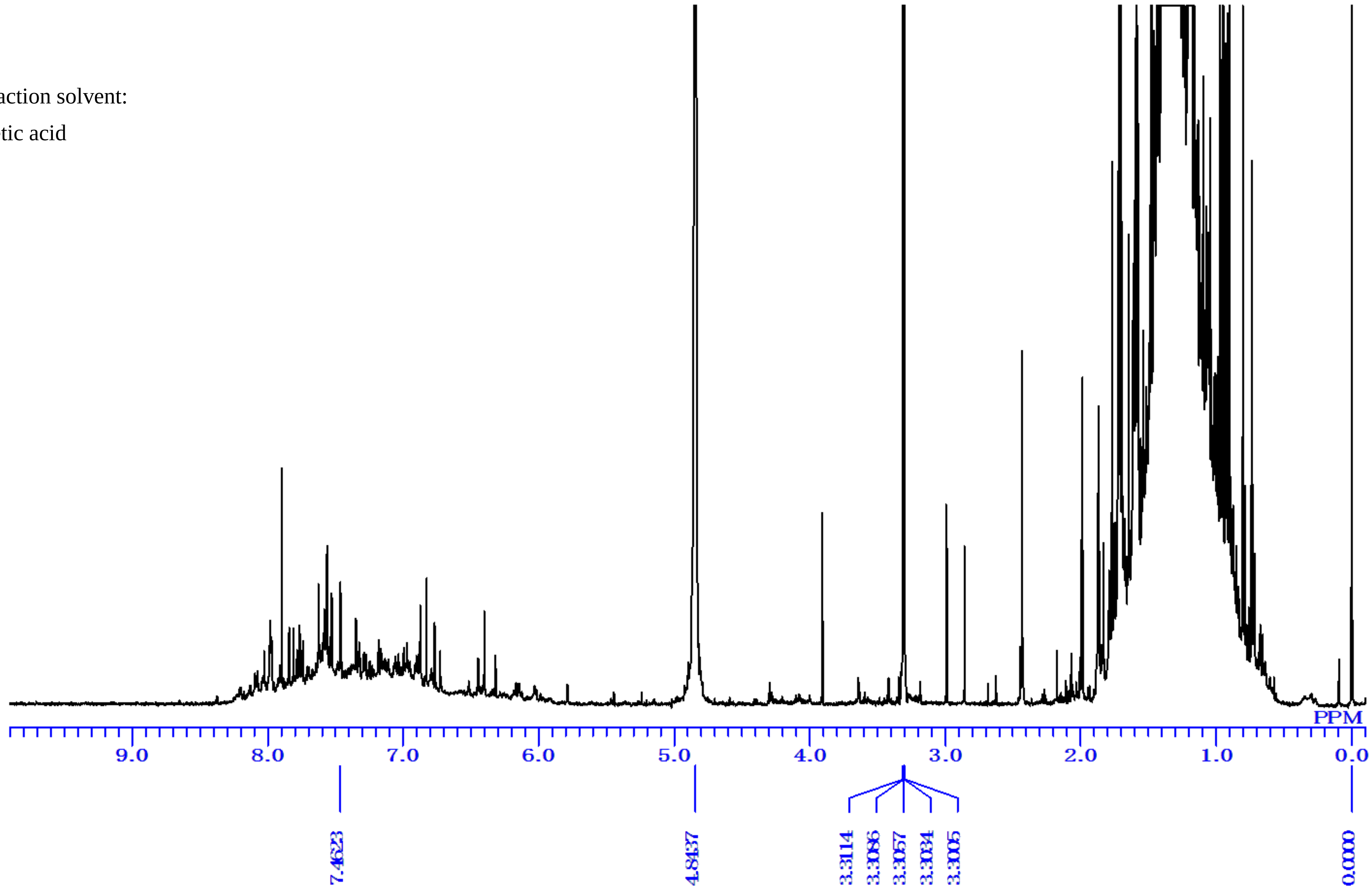

Supplementary Fig. 44 | ^{1}H NMR spectrum of the crude mixture (entry 20, 600 MHz, 20 °C, CD_3OD).

Entry 20, zoomed-in spectrum.

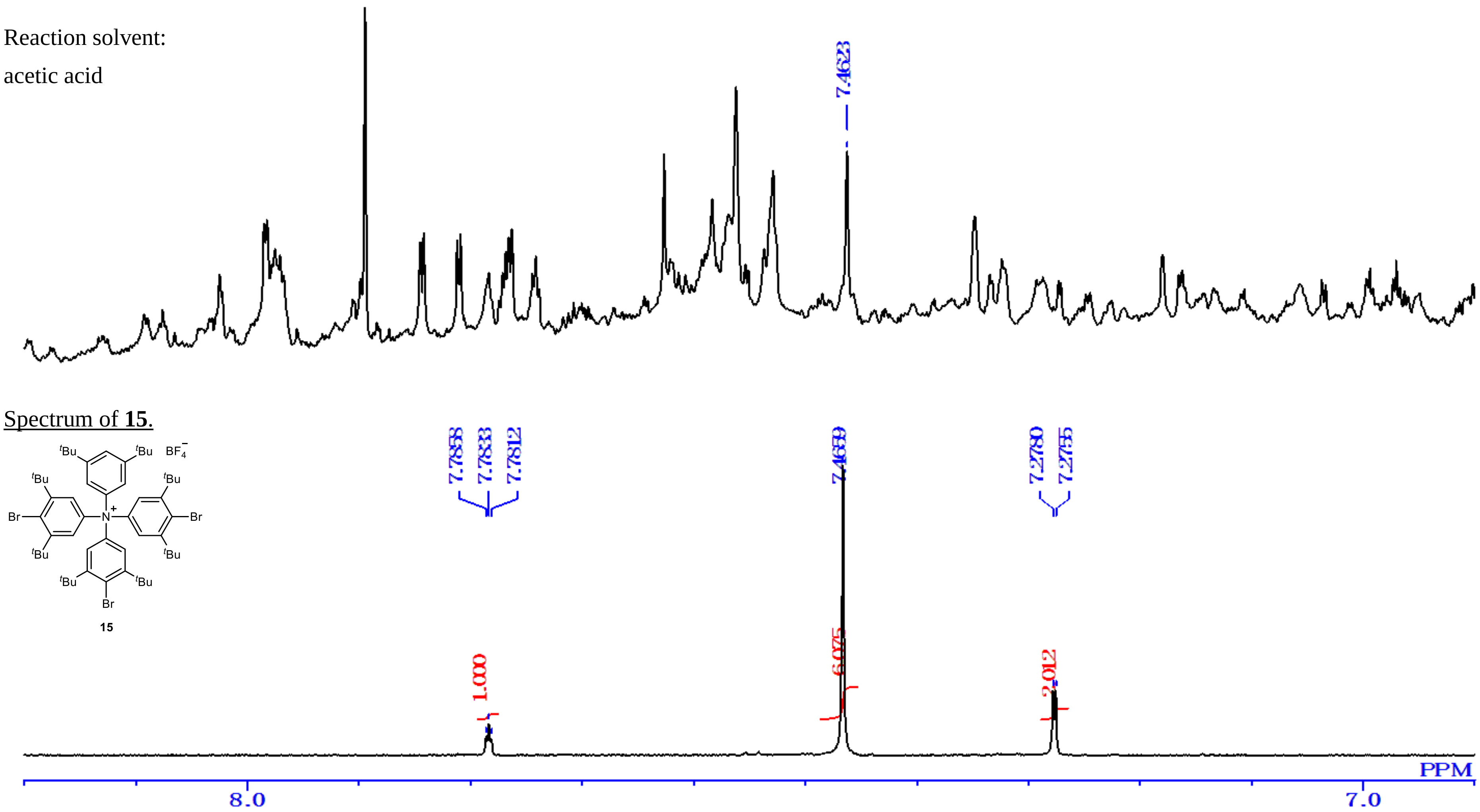

Supplementary Fig. 45 | Comparison of ^{1}H NMR spectra (600 MHz, 20 °C, CD_3OD) of the crude mixture (entry 20) and 15.

Reaction solvent:

benzonitrile

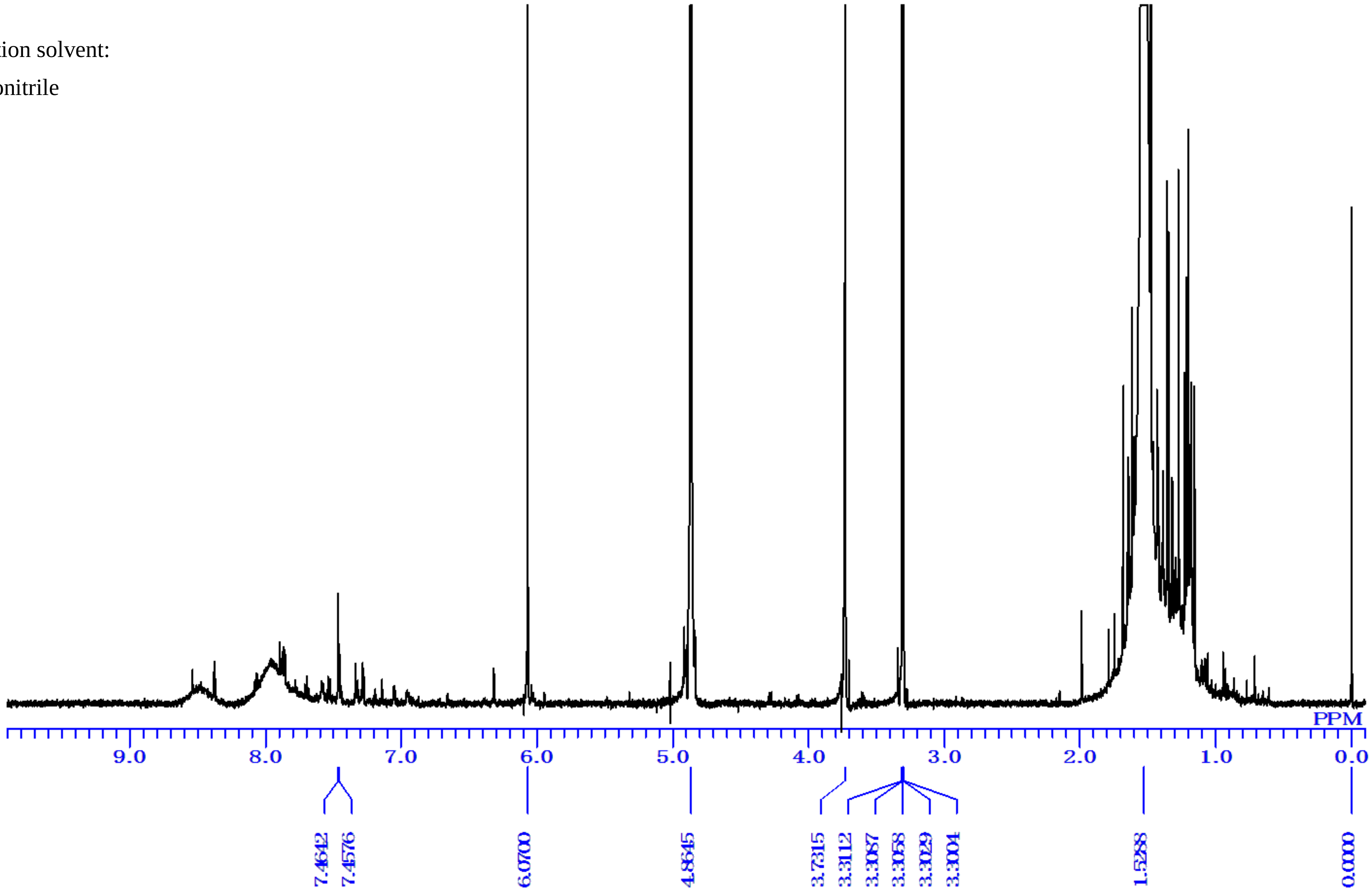

Supplementary Fig. 46 | ^{1}H NMR spectrum of the crude mixture (entry 21, 600 MHz, 20 °C, CD_3OD).

Entry 21, zoomed-in spectrum.

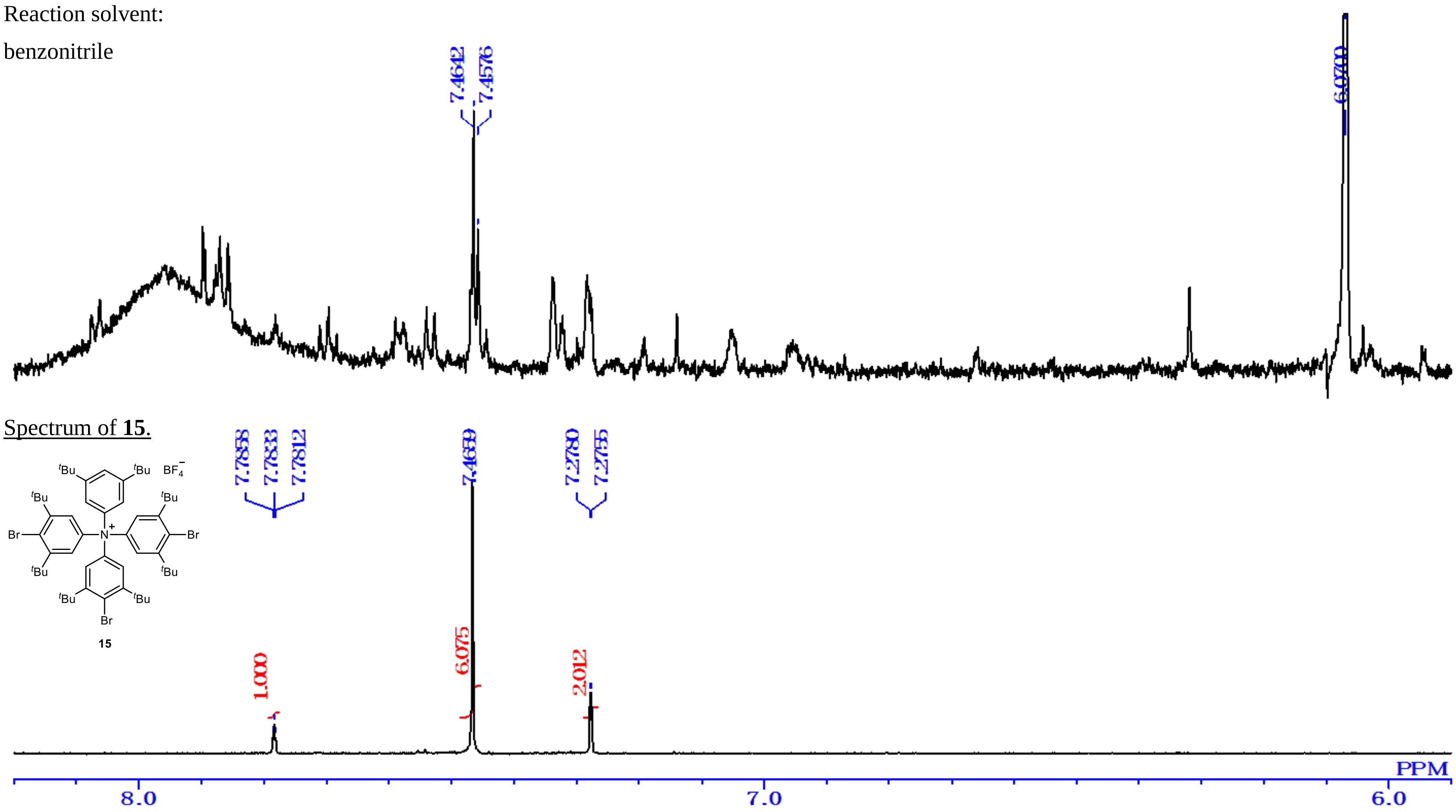

Supplementary Fig. 47 | Comparison of ^{1}H NMR spectra (600 MHz, 20 °C, CD_3OD) of the crude mixture (entry 21) and 15.

3.4. Experimental procedure for attempted synthesis of 20 from 19.

A mixture of triarylammoniumyl salt **19** (74.2 mg, 0.108 mmol), diacyl peroxide **13** (151 mg, 0.324 mmol), and **14** (24 µL, 0.11 mmol) in *o*-dichlorobenzene (1.1 ml) was heated to 120 °C for 15 min. Then, the reaction mixture was cooled to room temperature and was passed through $NaBF_4$-treated silica gel ($CHCl_3$/MeOH = 100:0 to 90:10). The eluent was concentrated under reduced pressure to afford a crude mixture, which was analyzed by ^1H NMR spectroscopy.

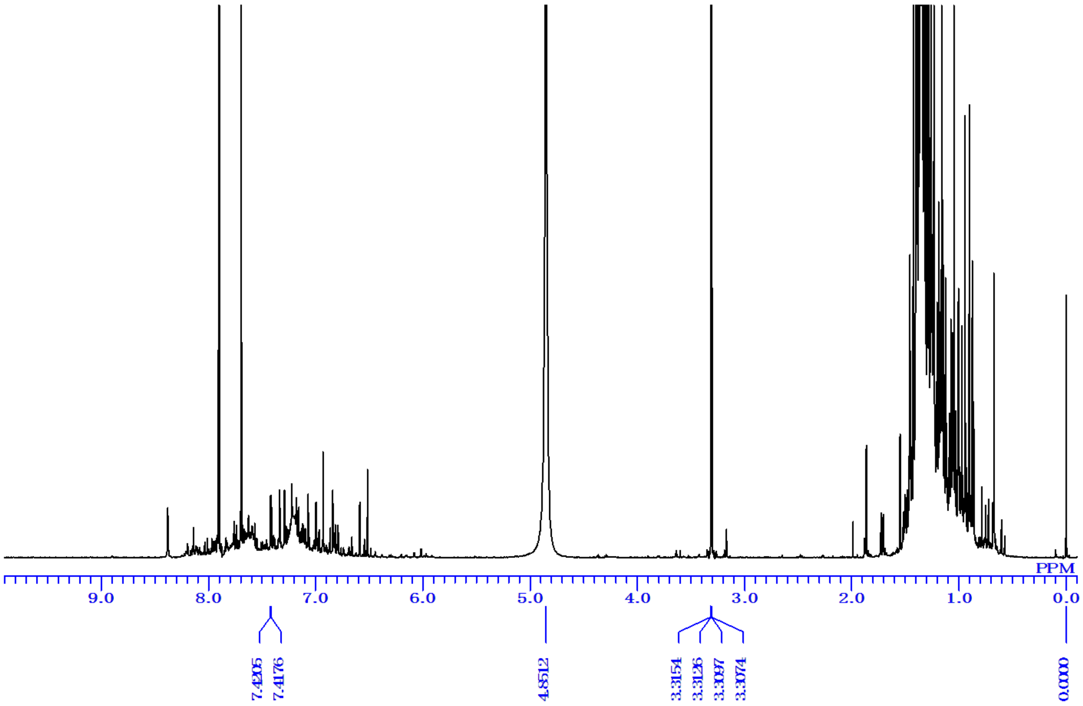

Supplementary Fig. 48 | ^1H NMR spectrum of the crude mixture (attempted synthesis of 20 from 19, 600 MHz, 20 °C, CD_3OD).

<u>Zoomed-in spectrum of the crude mixture.</u>

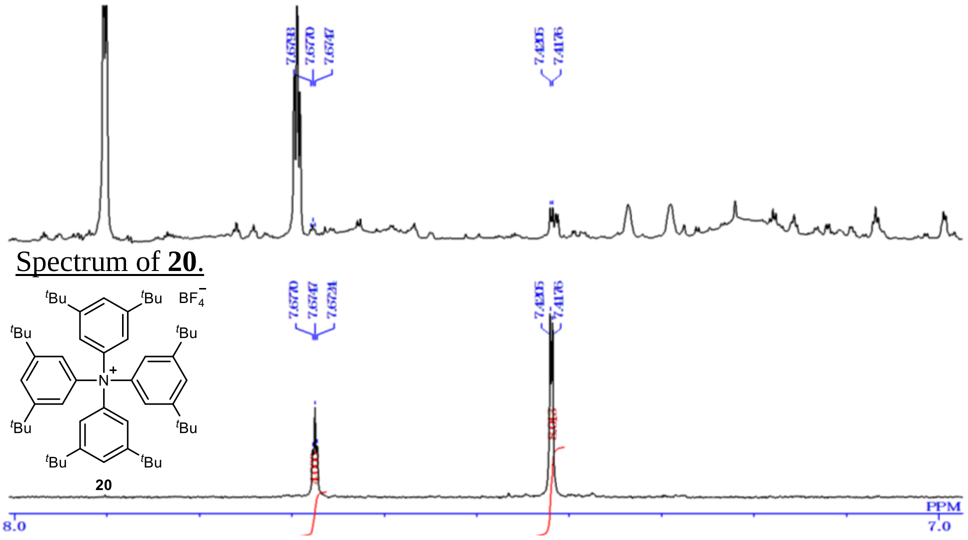

Supplementary Fig. 49 | Comparison of the ^1H NMR spectra (600 MHz, 20 °C, CD_3OD) of the crude mixture (attempted synthesis of 20 from 19) and pure 20.

3.5. Experimental procedure for the initial evaluation of alkaline stability.

Ammonium salts **20** (1 mg), **2-PF$_6$** (5 mg), or **23** (0.2 mg) was suspended in CD_3OD/D_2O (3:1, 620–640 μl, containing 5 M KOH). The resulting suspension was filtered and the filtrate was heated to 80 °C for 1–30 days in an NMR tube. The reaction mixture was quenched by the addition of aqueous HCl (1 M, 4 ml) and then extracted with Et_2O (5 ml). The organic layer was analyzed by ESI-MS.

4. Spectroscopic data

4.1. Absorption spectra

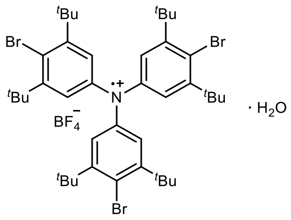

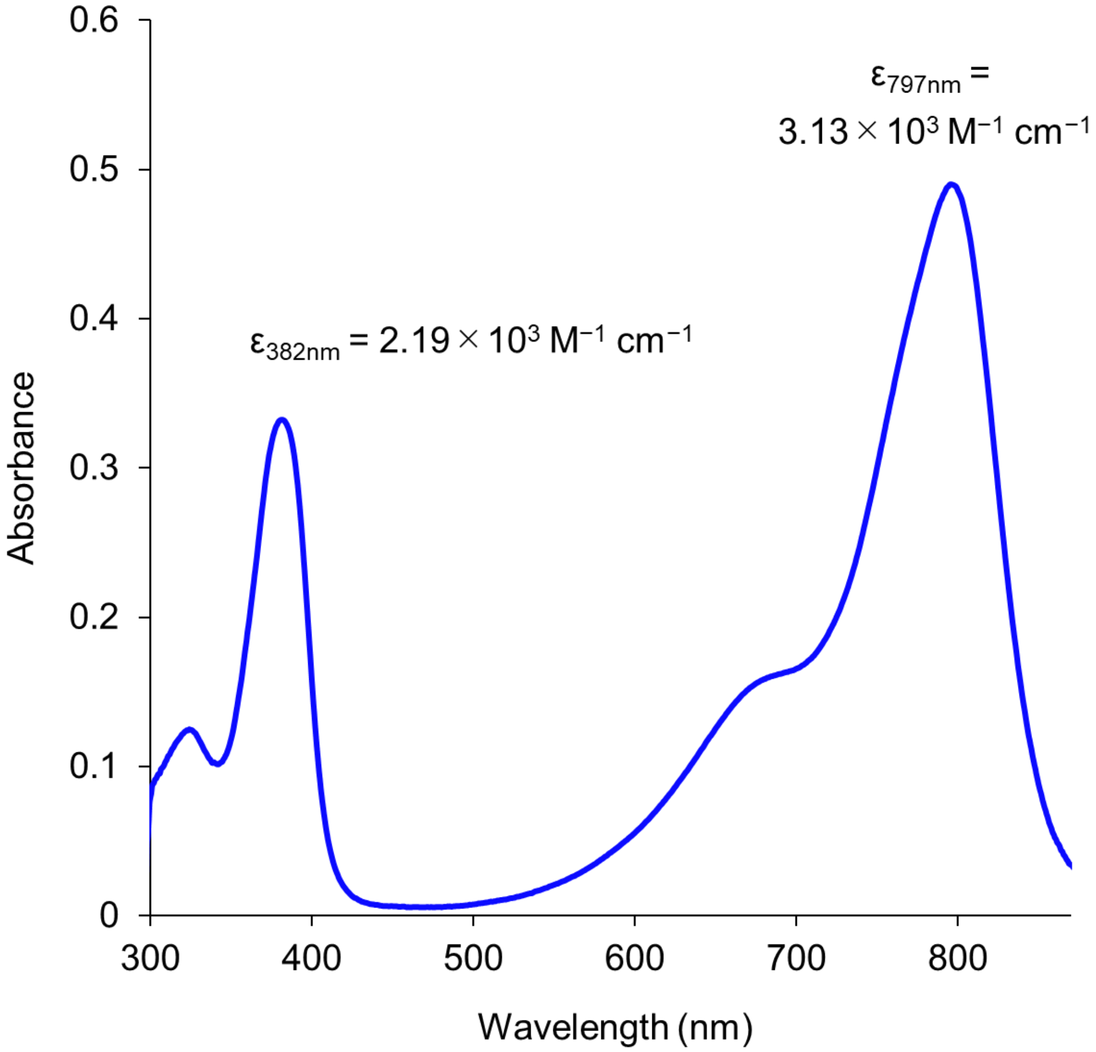

Supplementary Fig. 50 | Absorption spectrum of tris[(4-bromo-3,5-di-*tert*-butyl)phenyl]ammoniumyl tetrafluoroborate monohydrate (9) (16 μM, in *o*-dichlorobenzene).

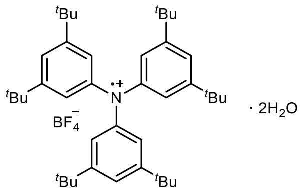

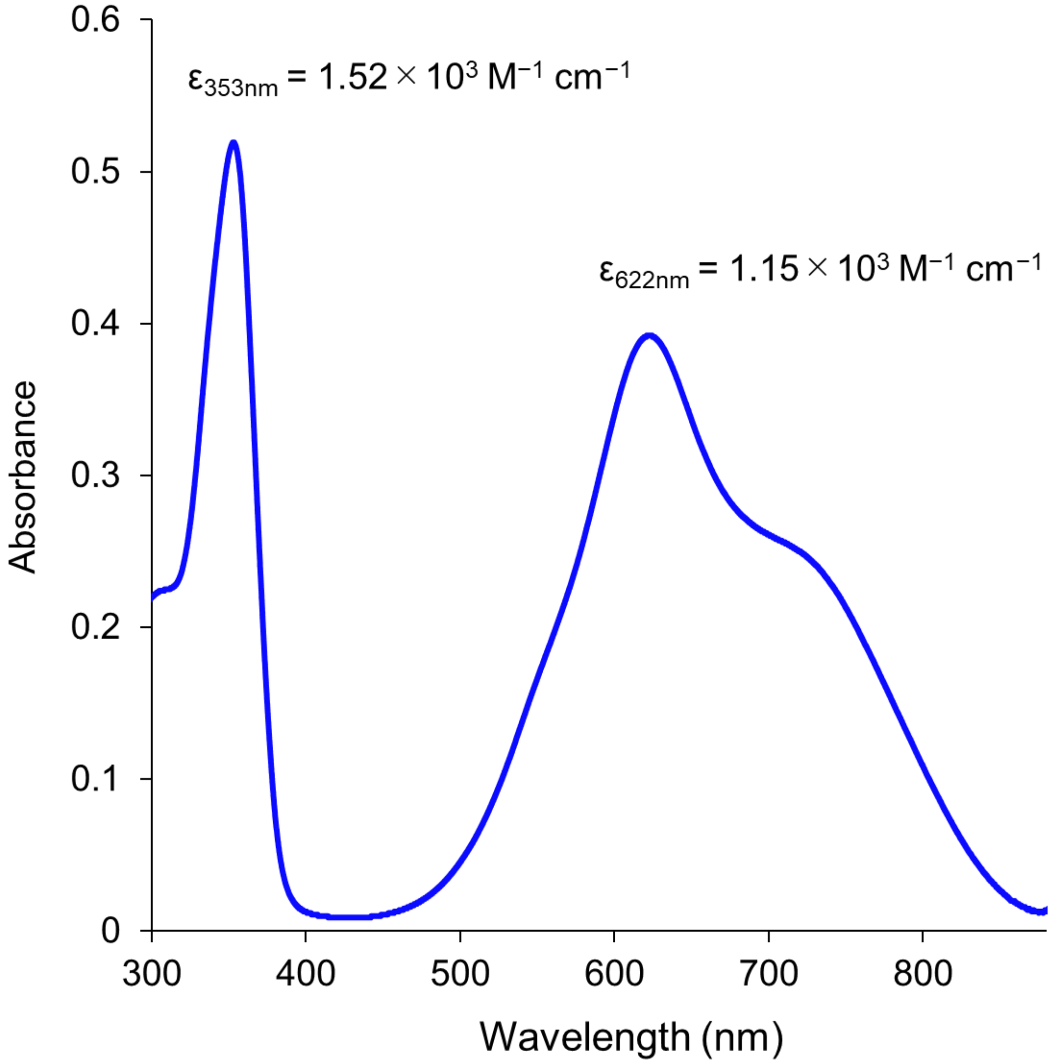

Supplementary Fig. 51 | Absorption spectrum of tris[(3,5-di-*tert*-butyl)phenyl]ammoniumyl tetrafluoroborate dihydrate (19) (35 μM, in *o*-dichlorobenzene).

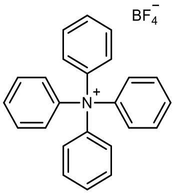

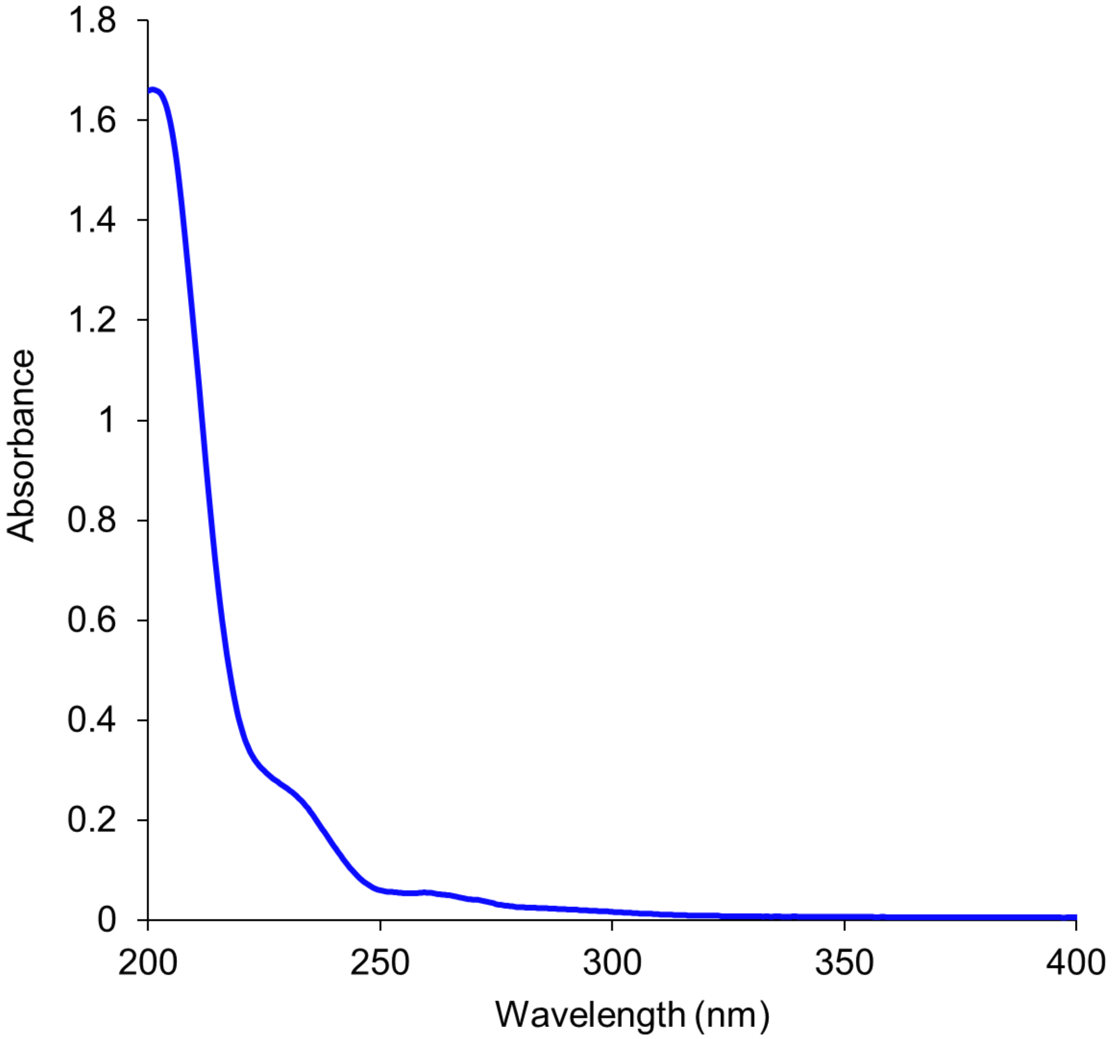

Supplementary Fig. 52 | Absorption spectrum of tetraphenylammonium tetrafluoroborate (23) (34 μM, in MeCN).

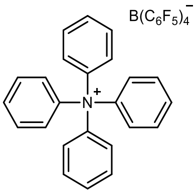

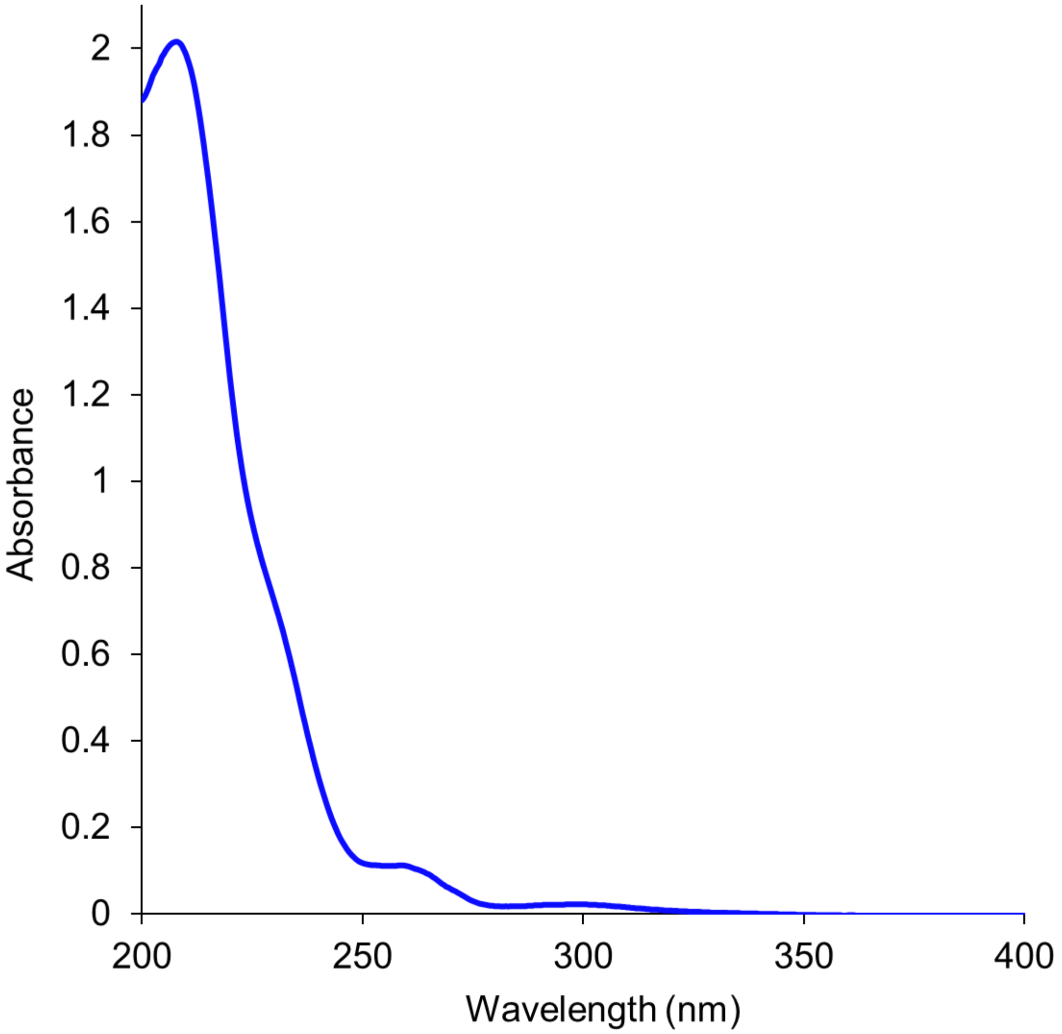

Supplementary Fig. 53 | Absorption spectrum of tetraphenylammonium tetrakis(pentafluorophenyl)borate (24) (18 μM, in MeCN).

4.2. IR spectra

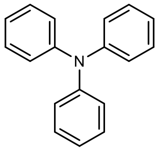

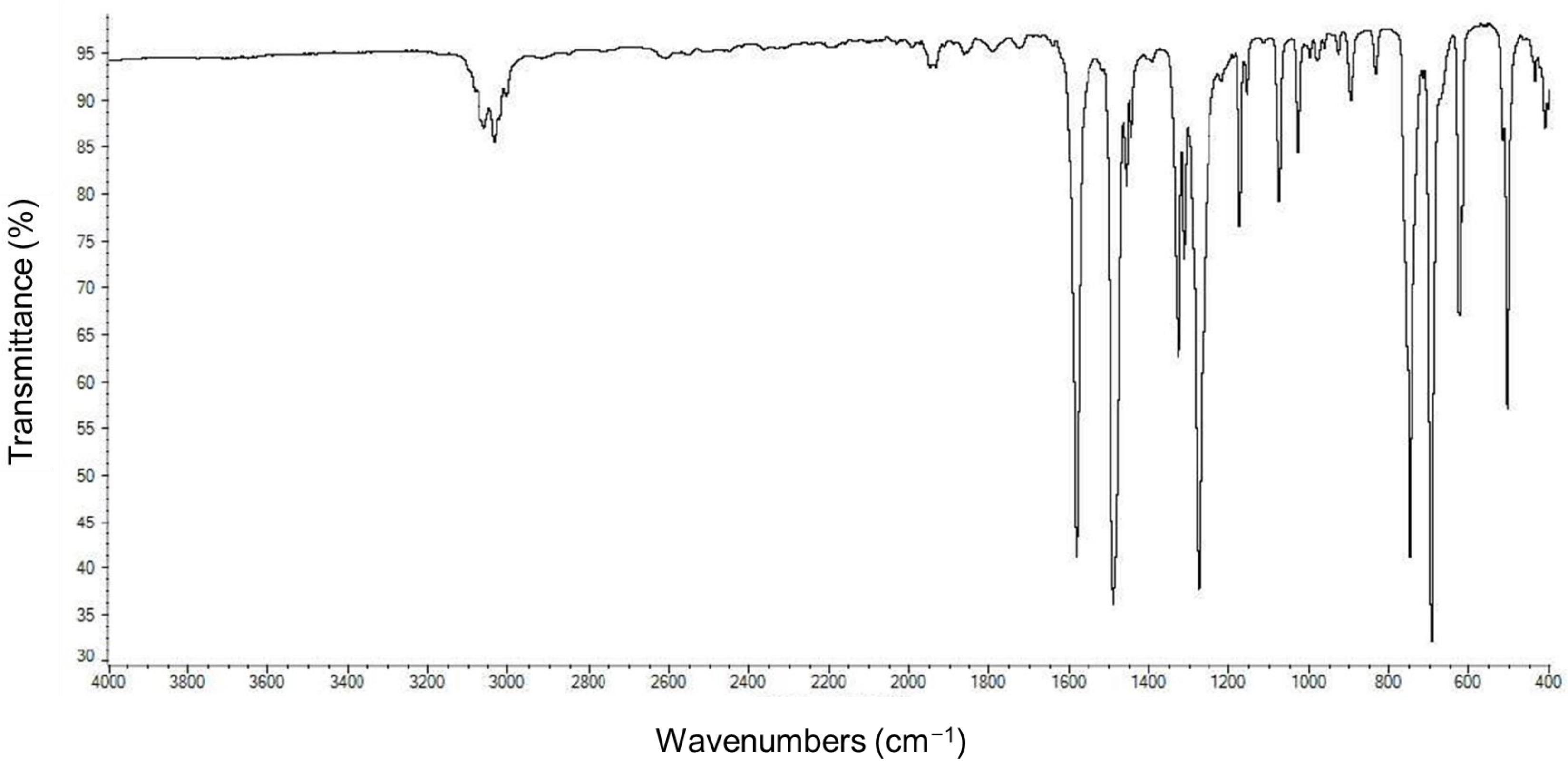

Supplementary Fig. 54 | IR spectrum of triphenylamine (8).

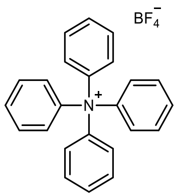

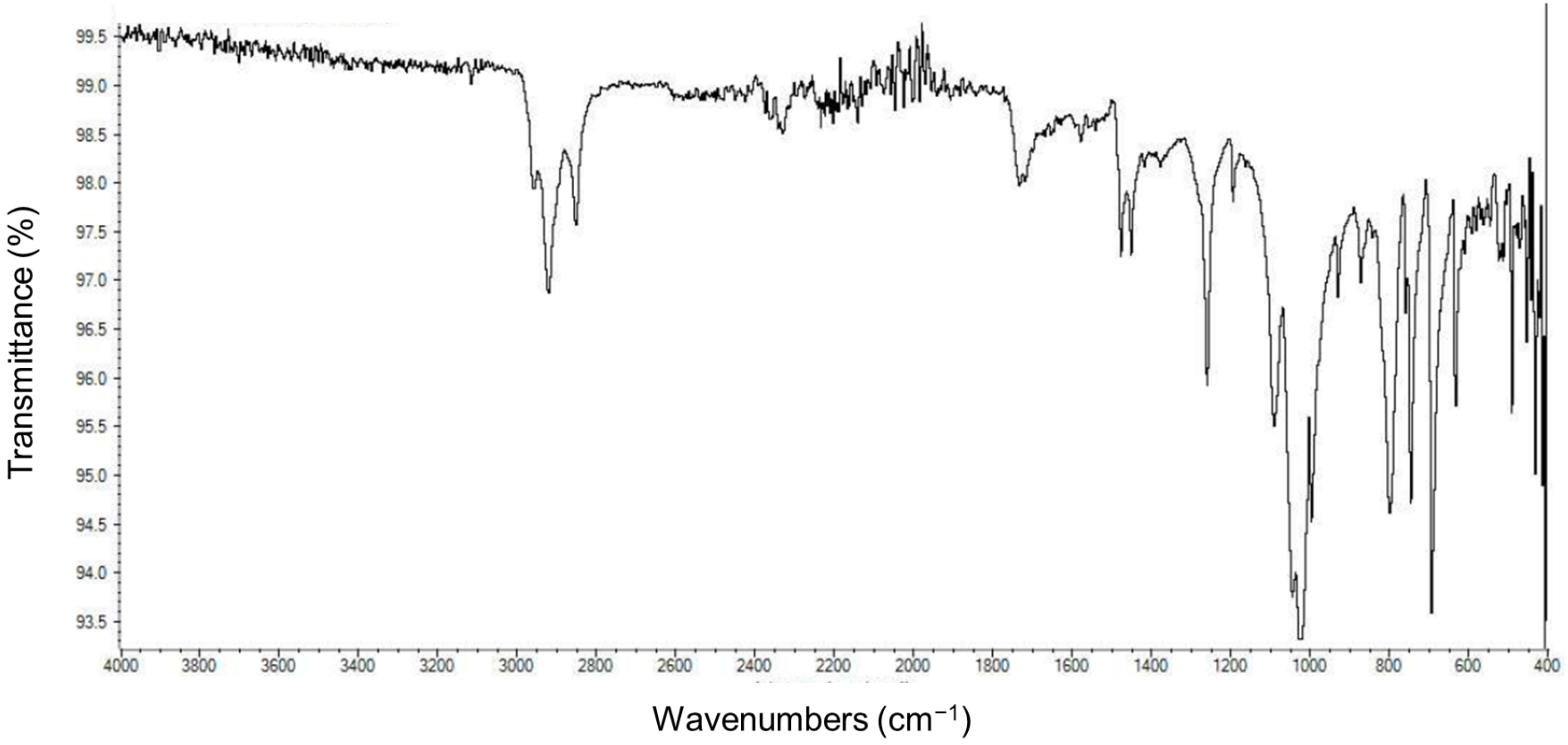

Supplementary Fig. 55 | IR spectrum of tetraphenylammonium tetrafluoroborate (23).

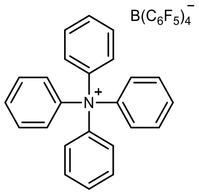

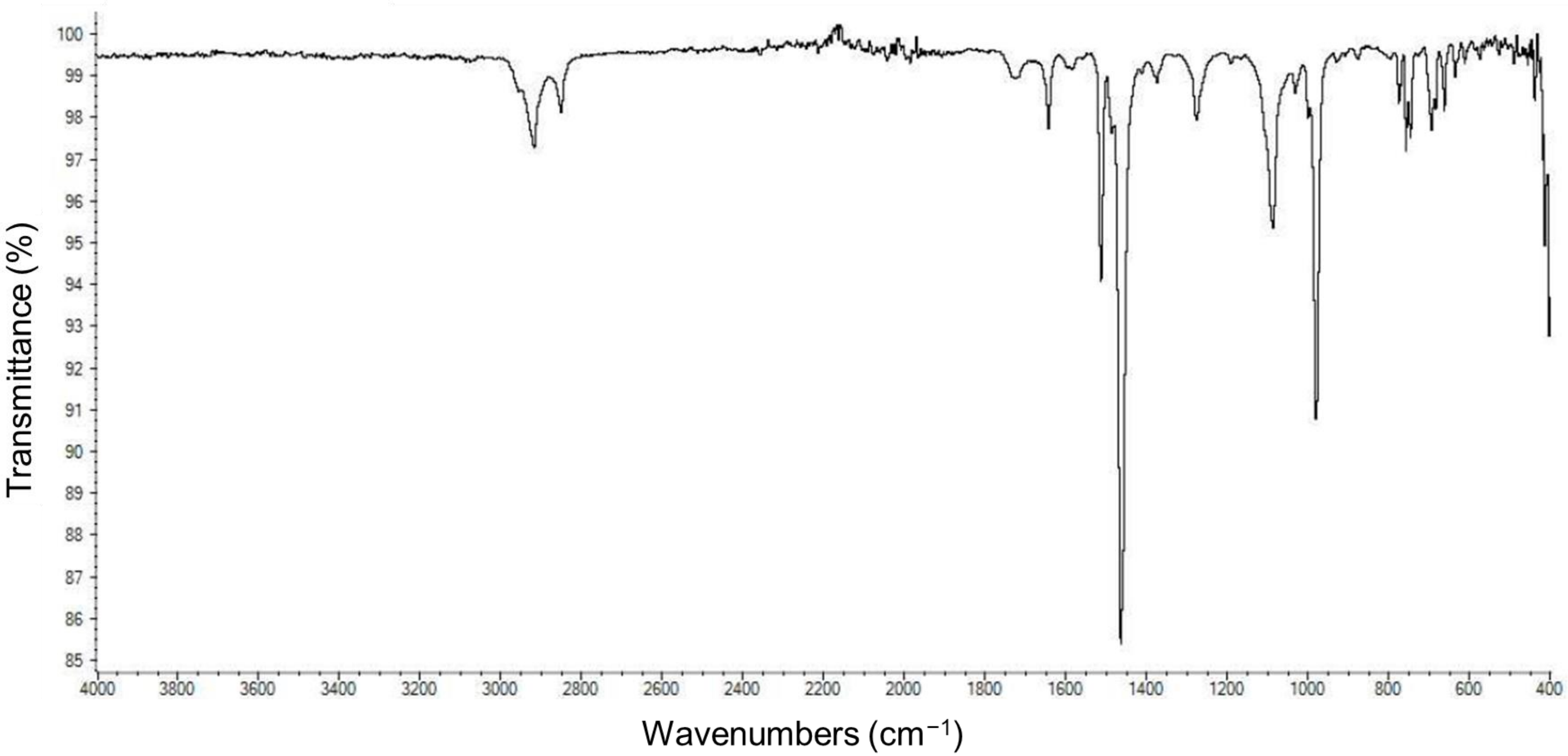

Supplementary Fig. 56 | IR spectrum of tetraphenylammonium tetrakis(pentafluorophenyl)borate (24).

4.3. NMR spectra

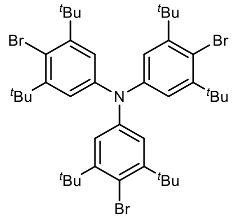

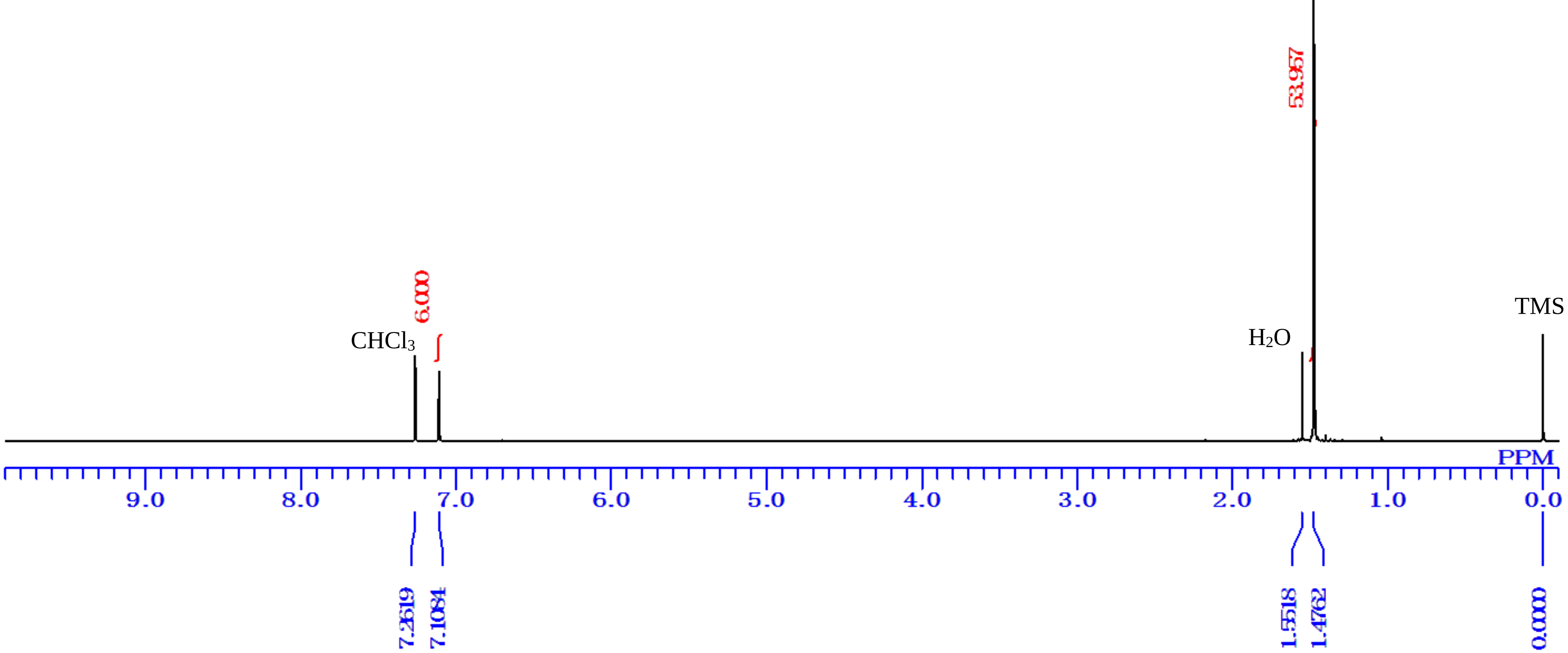

Supplementary Fig. 57 | ^{1}H NMR spectrum of tris[(4-bromo-3,5-di-*tert*-butyl)phenyl]amine (11) (600 MHz, 20 °C, $CDCl_3$).

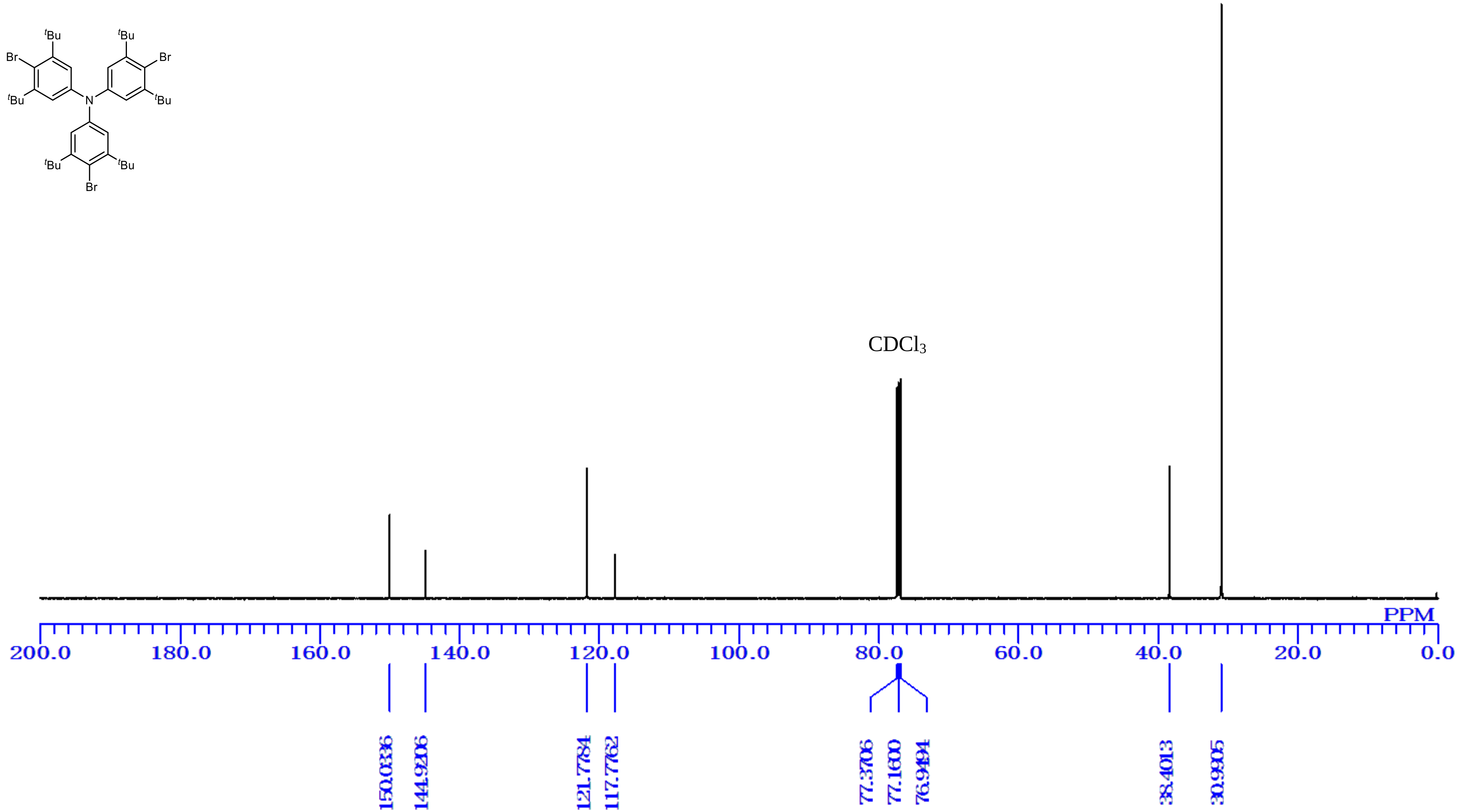

Supplementary Fig. 58 | ^{13}C NMR spectrum of tris[(4-bromo-3,5-di-*tert*-butyl)phenyl]amine (11) (150 MHz, 20 °C, $CDCl_3$).

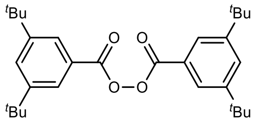

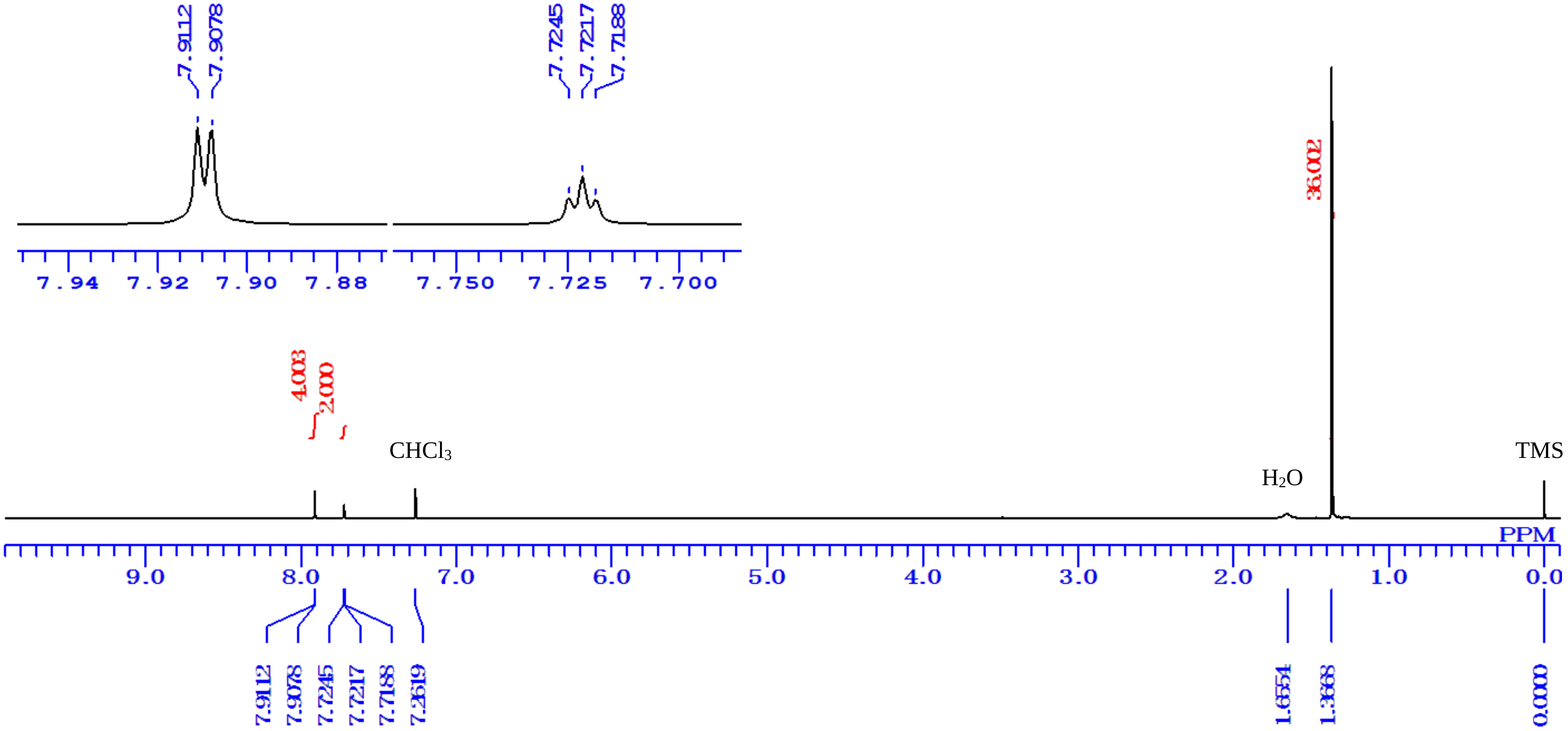

Supplementary Fig. 59 | ^{1}H NMR spectrum of bis(3,5-di-*tert*-butyl)benzoyl peroxide (13) (600 MHz, 20 °C, $CDCl_3$).

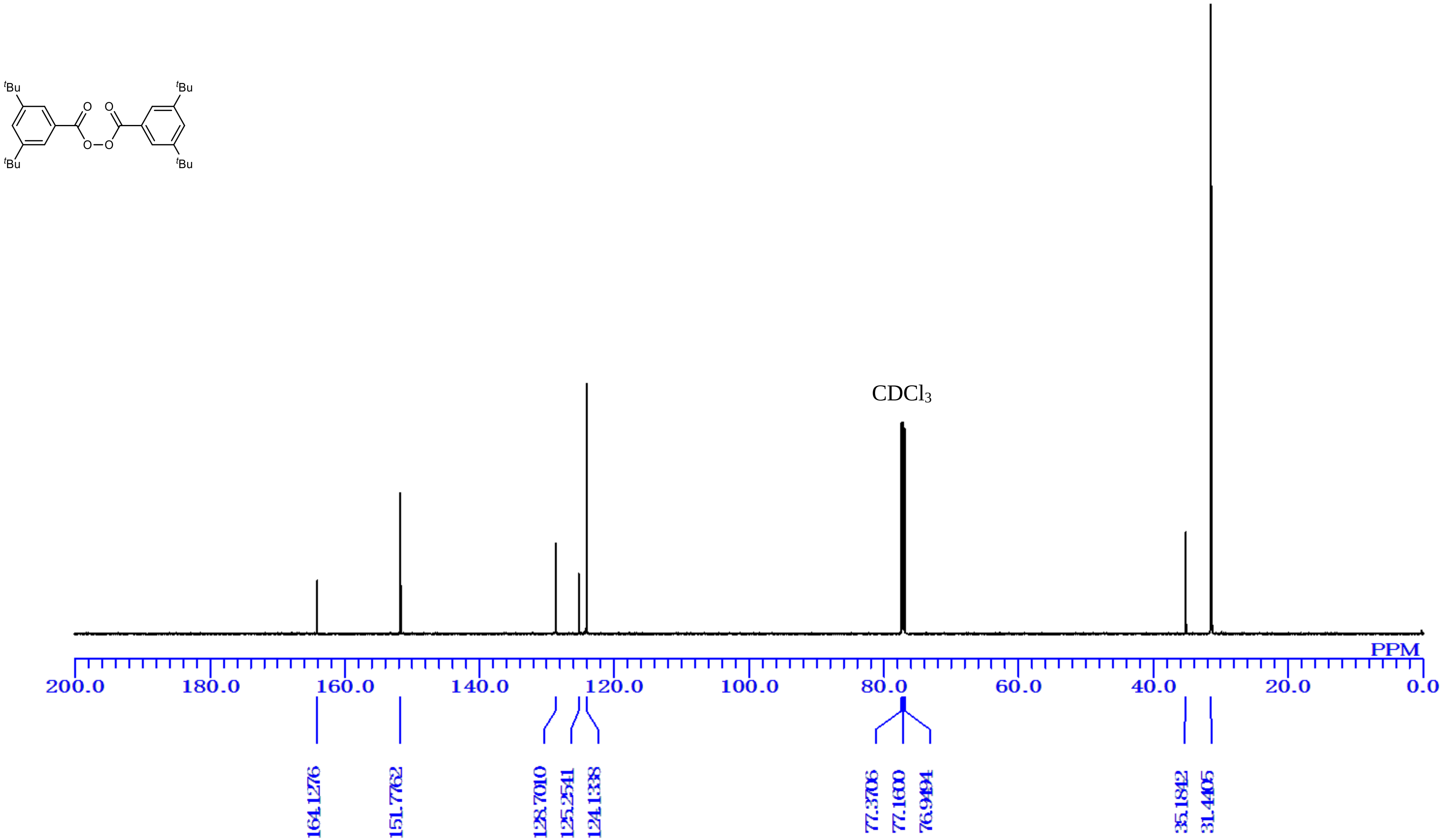

Supplementary Fig. 60 | ^{13}C NMR spectrum of bis(3,5-di-*tert*-butyl)benzoyl peroxide (13) (150 MHz, 20 °C, $CDCl_3$).

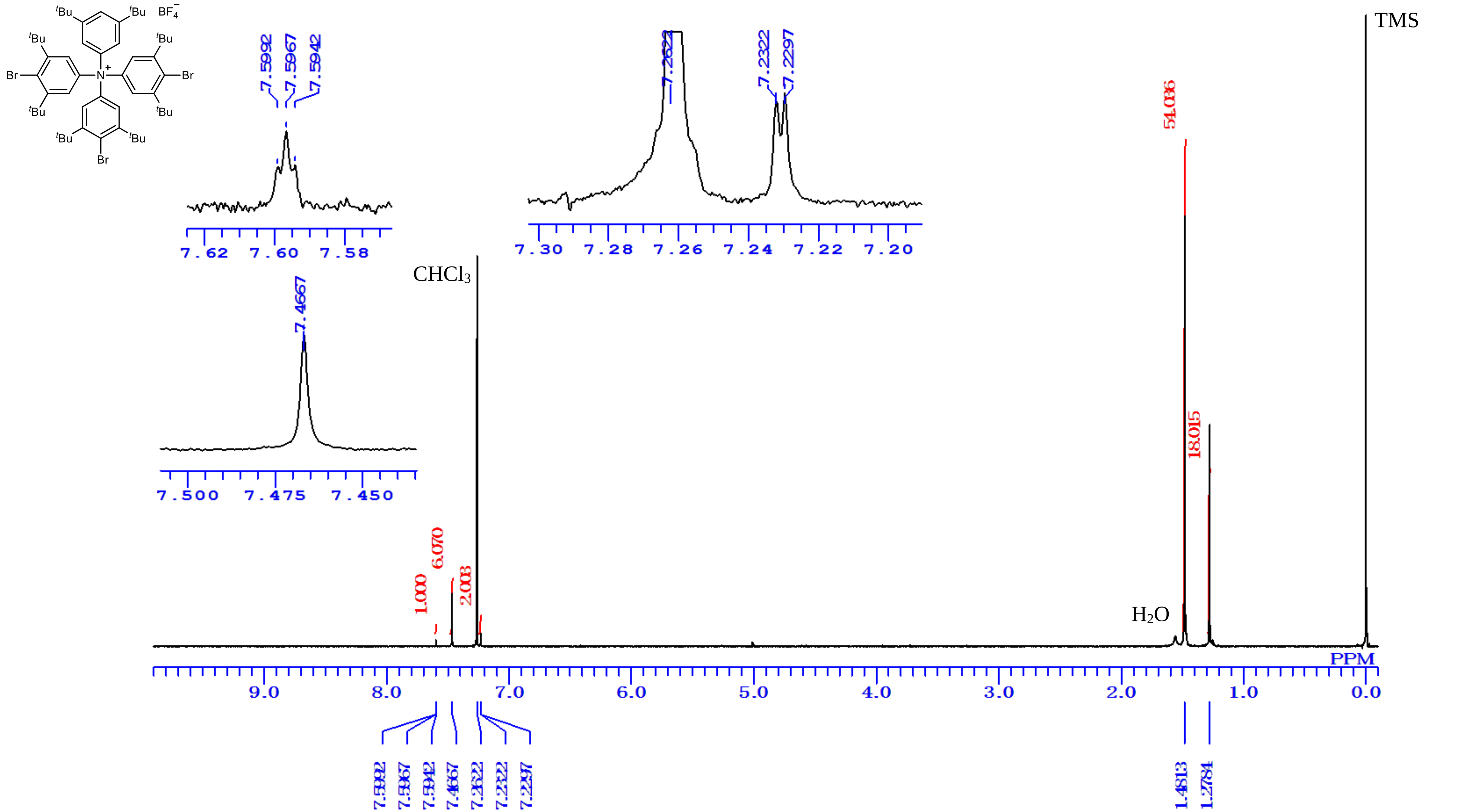

Supplementary Fig. 61 | ^{1}H NMR spectrum of tris[(4-bromo-3,5-di-*tert*-butyl)phenyl][(3,5-di-*tert*-butyl)phenyl]ammonium tetrafluoroborate (15) (600 MHz, 20 °C, $CDCl_3$).

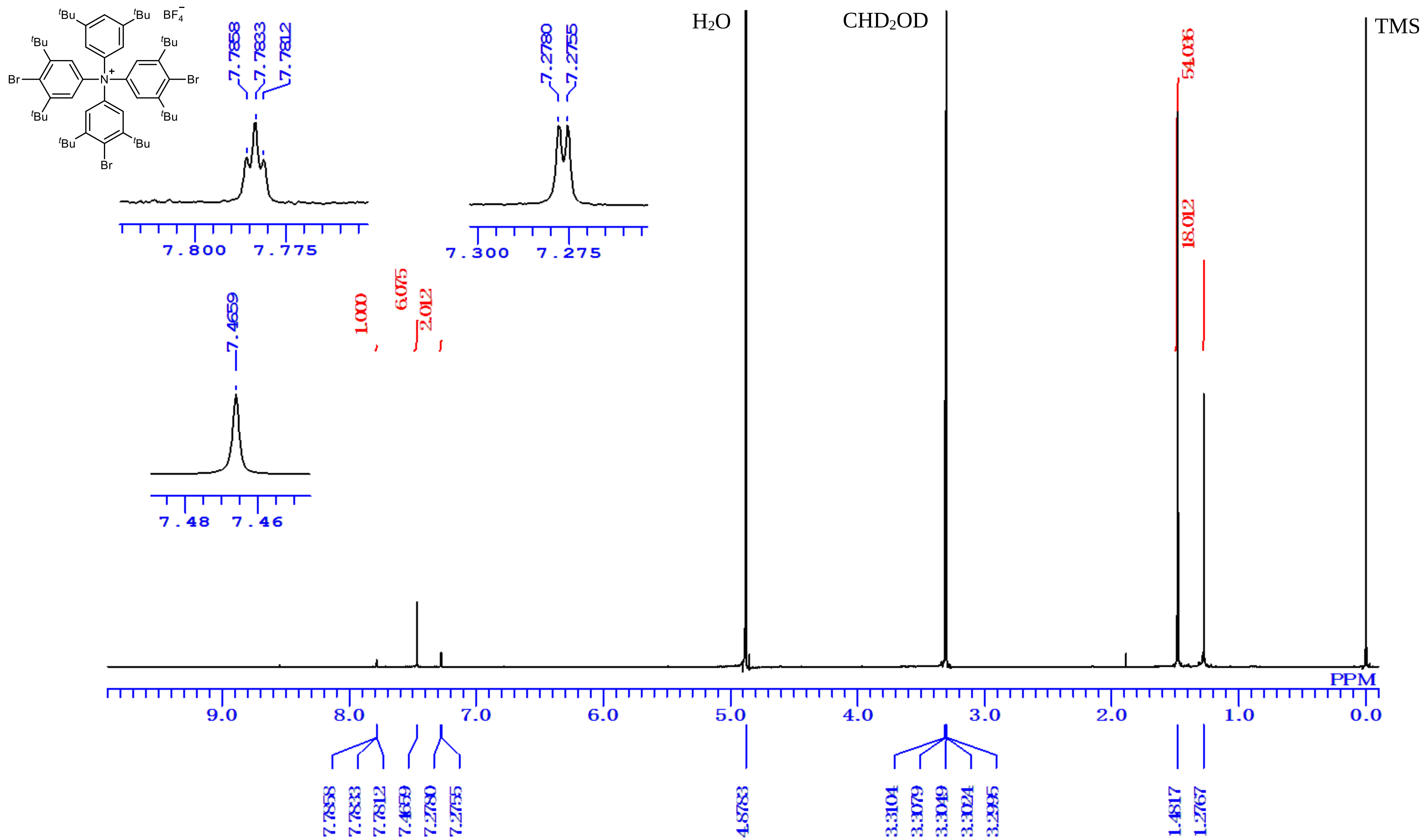

Supplementary Fig. 62 | ^{1}H NMR spectrum of tris[(4-bromo-3,5-di-*tert*-butyl)phenyl][(3,5-di-*tert*-butyl)phenyl]ammonium tetrafluoroborate (15) (600 MHz, 20 °C, CD_3OD).

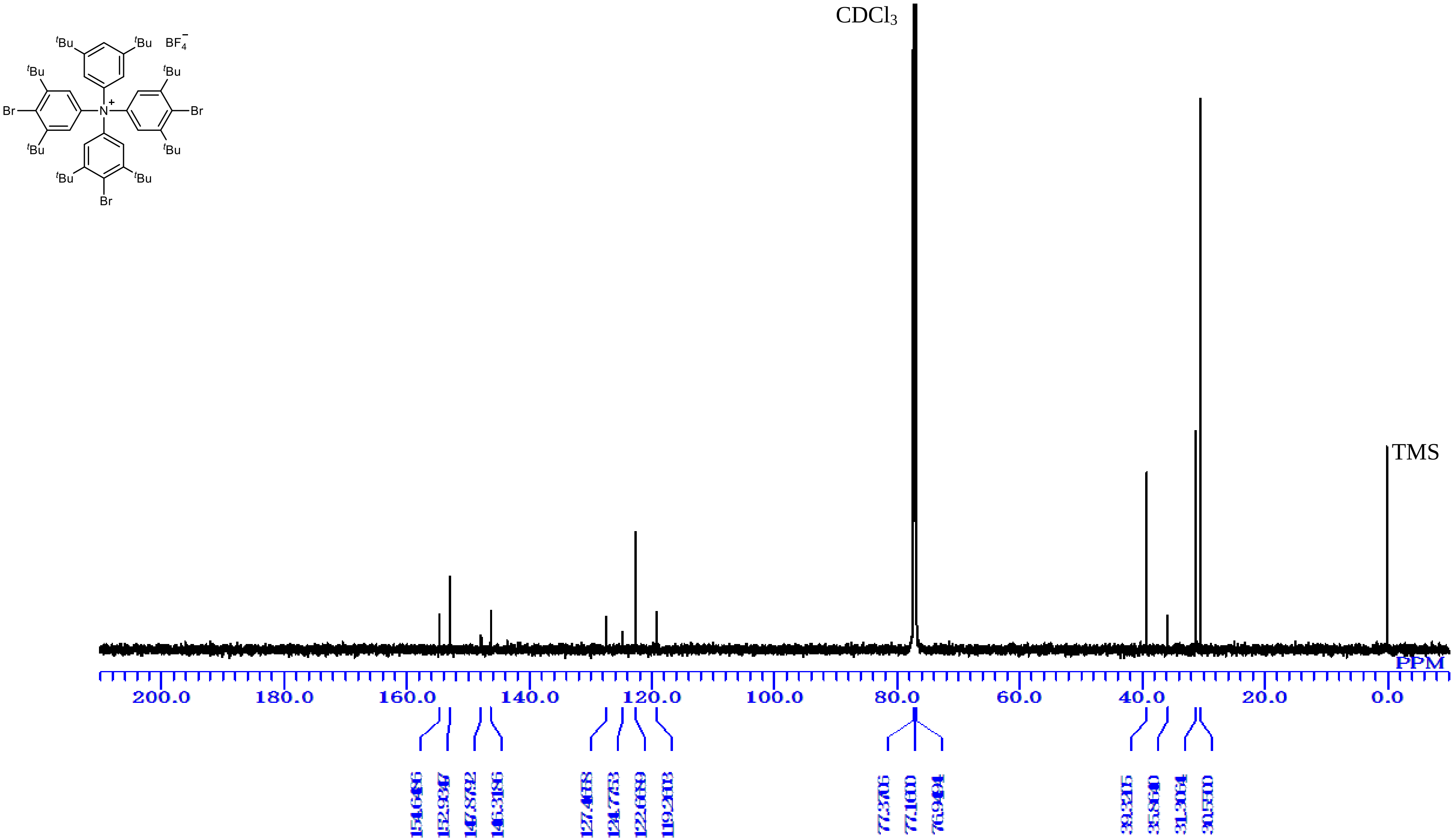

Supplementary Fig. 63 | ^{13}C NMR spectrum of tris[(4-bromo-3,5-di-*tert*-butyl)phenyl][(3,5-di-*tert*-butyl)phenyl]ammonium tetrafluoroborate (15) (150 MHz, 20 °C, $CDCl_3$).

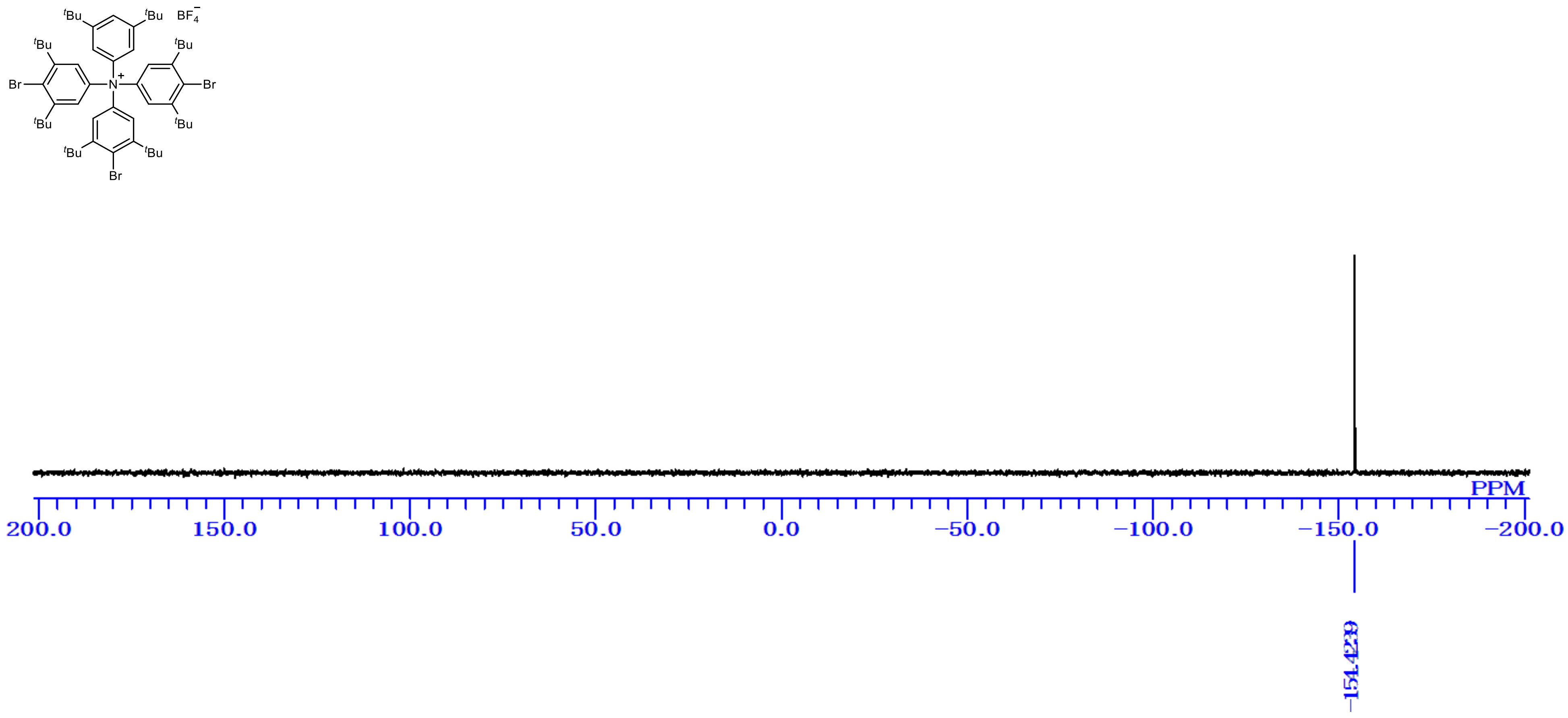

Supplementary Fig. 64 | ^{19}F NMR spectrum of tris[(4-bromo-3,5-di-*tert*-butyl)phenyl][(3,5-di-*tert*-butyl)phenyl]ammonium tetrafluoroborate (15) (376 MHz, 20 °C, $CDCl_3$).

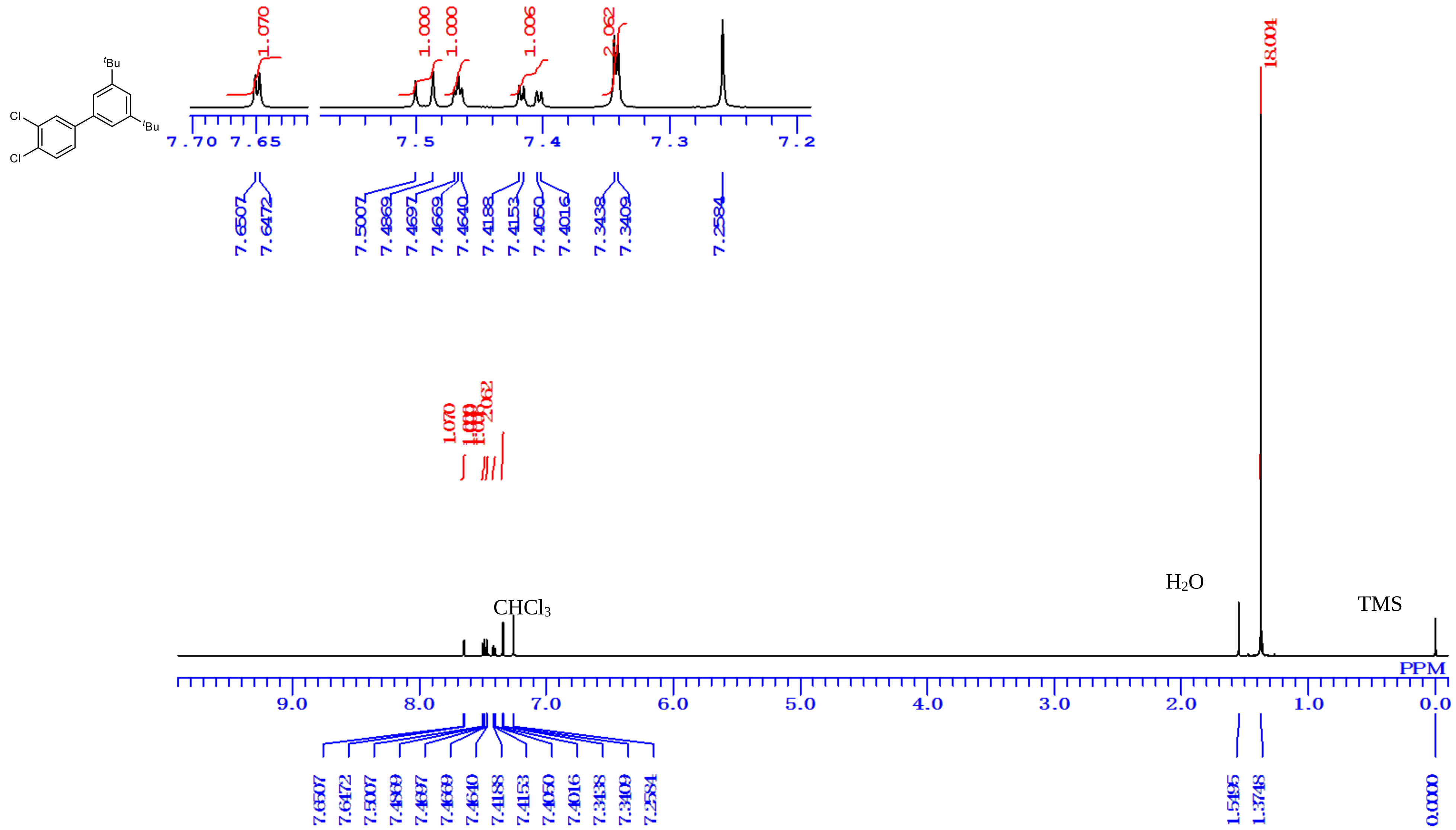

Supplementary Fig. 65 | ^{1}H NMR spectrum of 3',5'-di-*tert*-butyl-3,4-dichloro-1,1'-biphenyl (16) (600 MHz, 20 °C, $CDCl_3$).

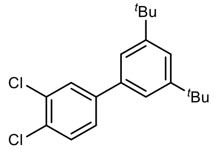

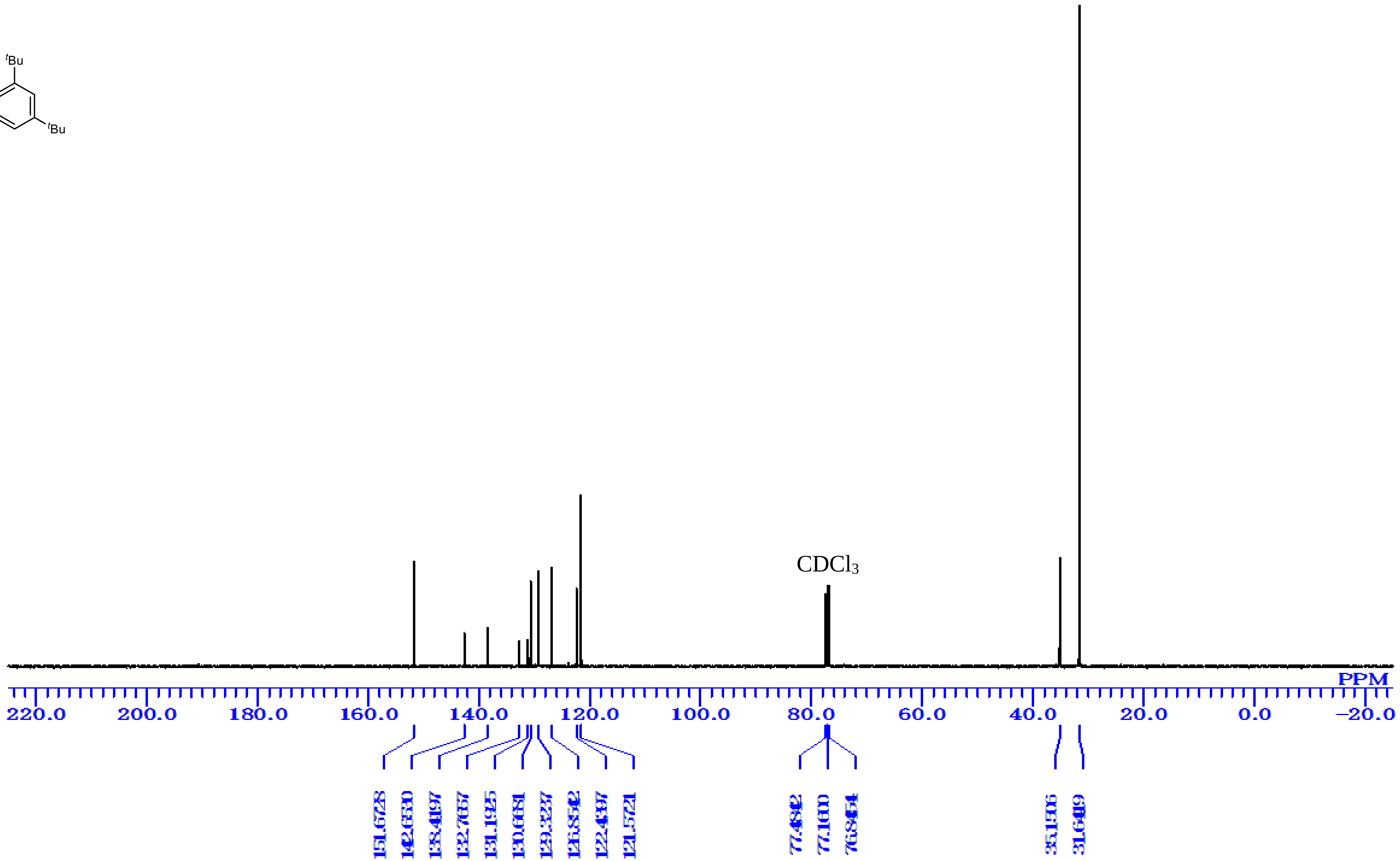

Supplementary Fig. 66 | ^{13}C NMR spectrum of 3',5'-di-*tert*-butyl-3,4-dichloro-1,1'-biphenyl (16) (100 MHz, 20 °C, $CDCl_3$).

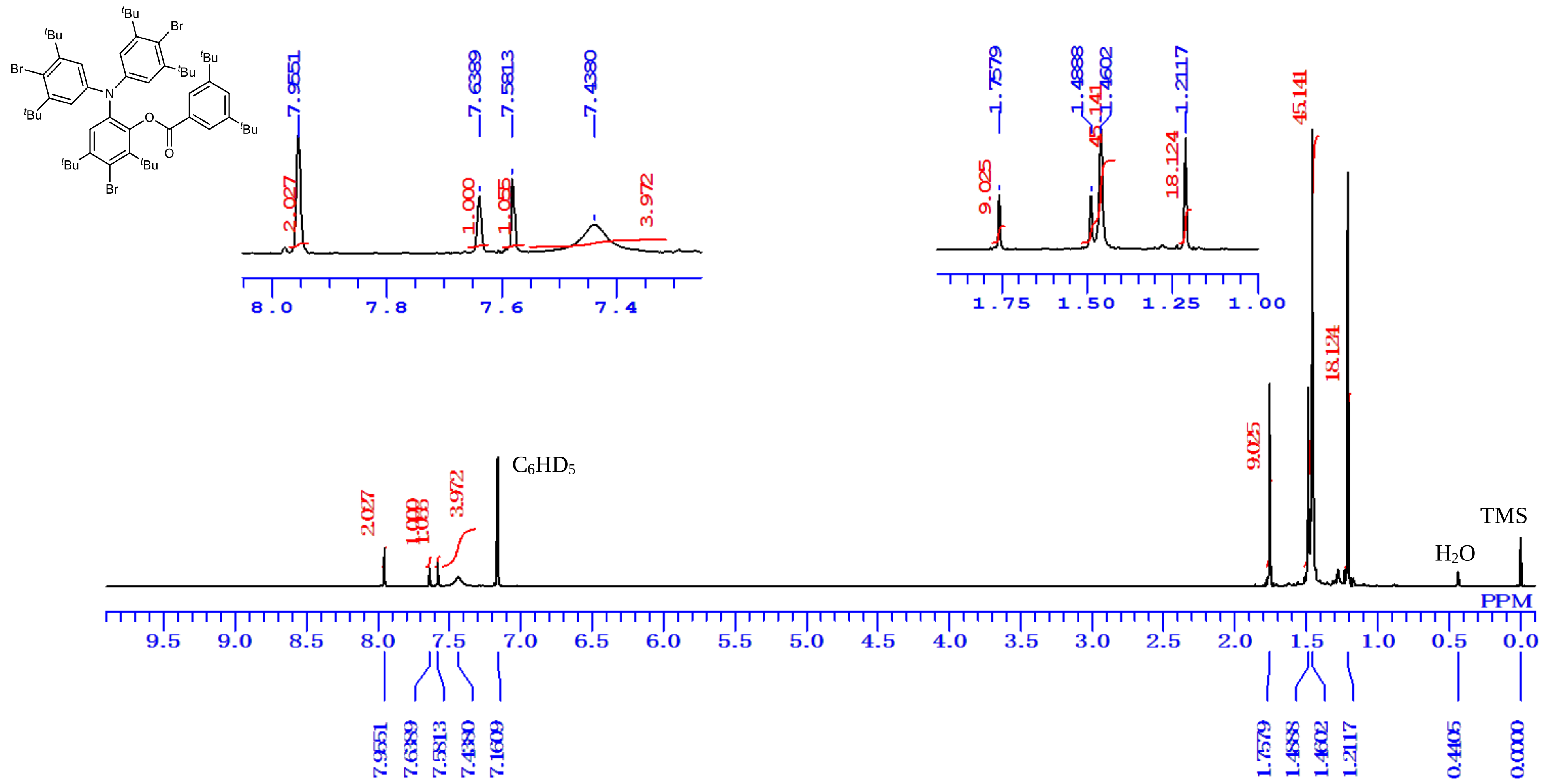

Supplementary Fig. 67 | ^{1}H NMR spectrum of {4-bromo-3,5-di-*tert*-butyl-2-[(3,5-di-*tert*-butyl)benzoyloxy]phenyl}bis[(4-bromo-3,5-di-*tert*-butyl)phenyl]amine (17) (600 MHz, 70 °C, C_6D_6).

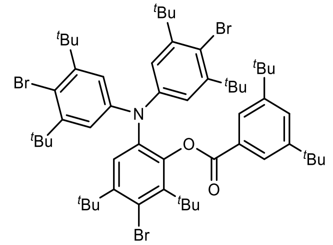

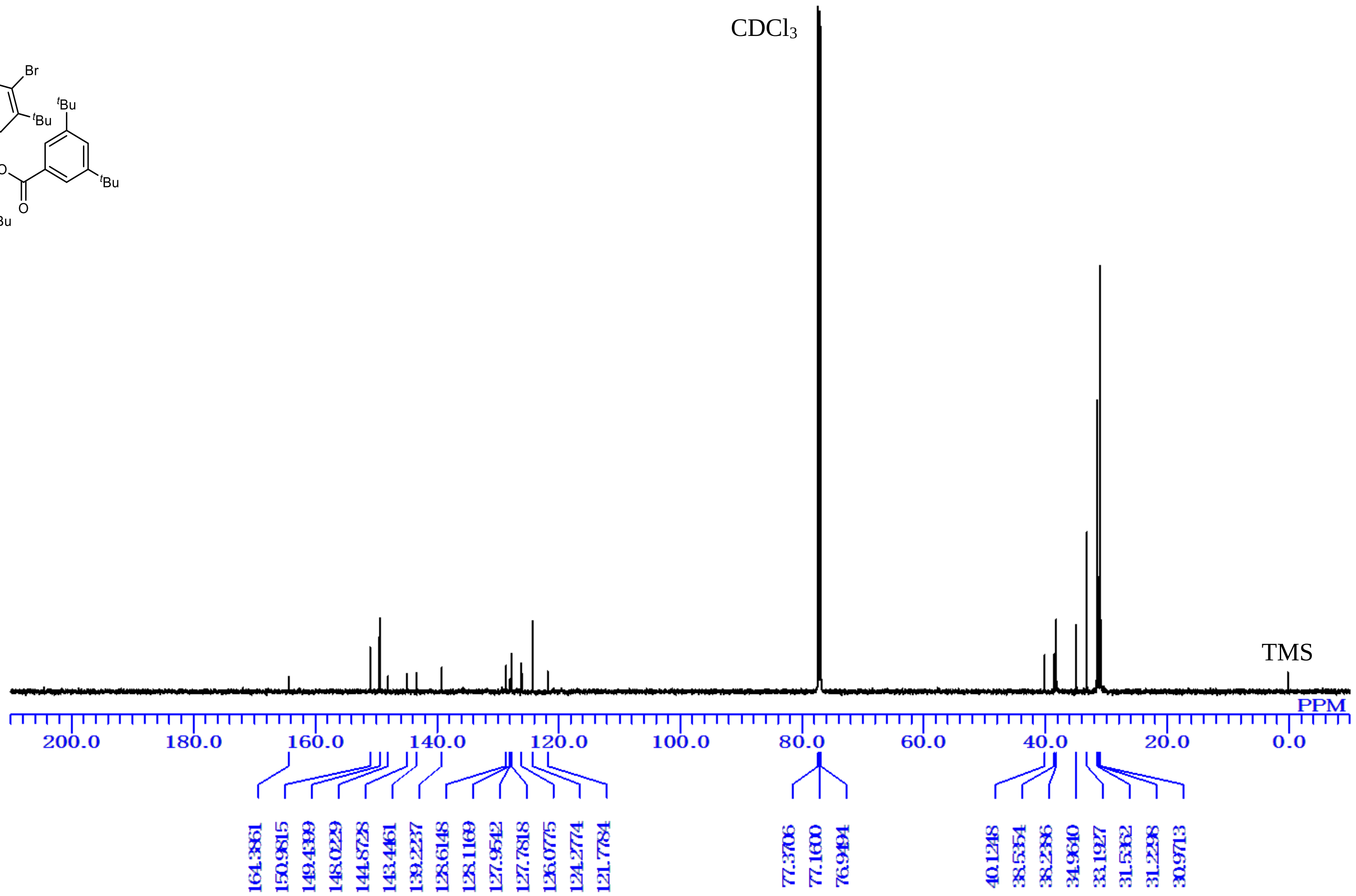

Supplementary Fig. 68 | ^{13}C NMR spectrum of {4-bromo-3,5-di-*tert*-butyl-2-[(3,5-di-*tert*-butyl)benzoyloxy]phenyl}bis[(4-bromo-3,5-di-*tert*-butyl)phenyl]amine (17) (150 MHz, 20 °C, $CDCl_3$).

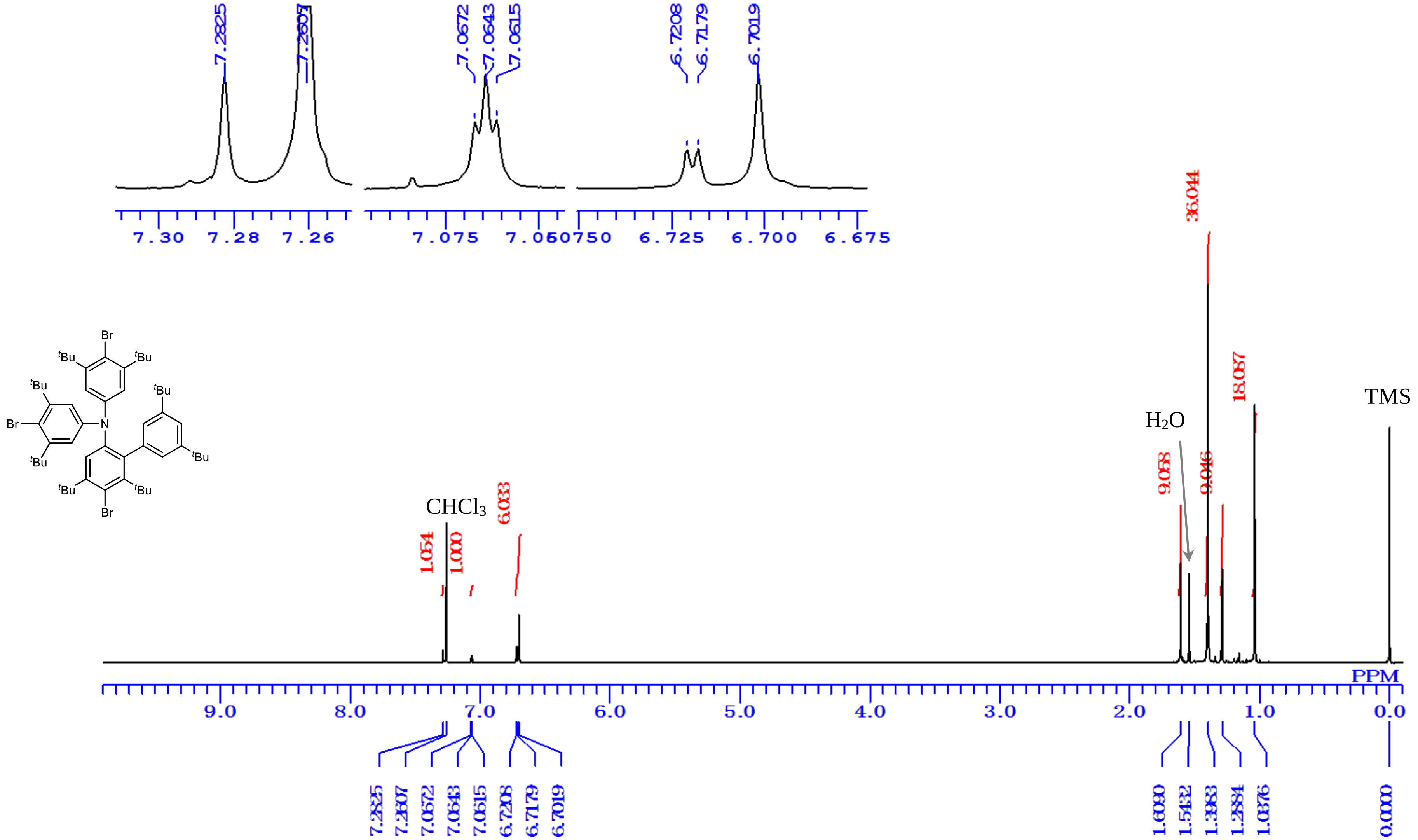

Supplementary Fig. 69 | ^{1}H NMR spectrum of bis[(4-bromo-3,5-di-*tert*-butyl)phenyl](5-bromo-3',4,5',6-tetra-*tert*-butyl-[1,1'-biphenyl]-2-yl)amine (18) (600 MHz, 20 °C, $CDCl_3$).

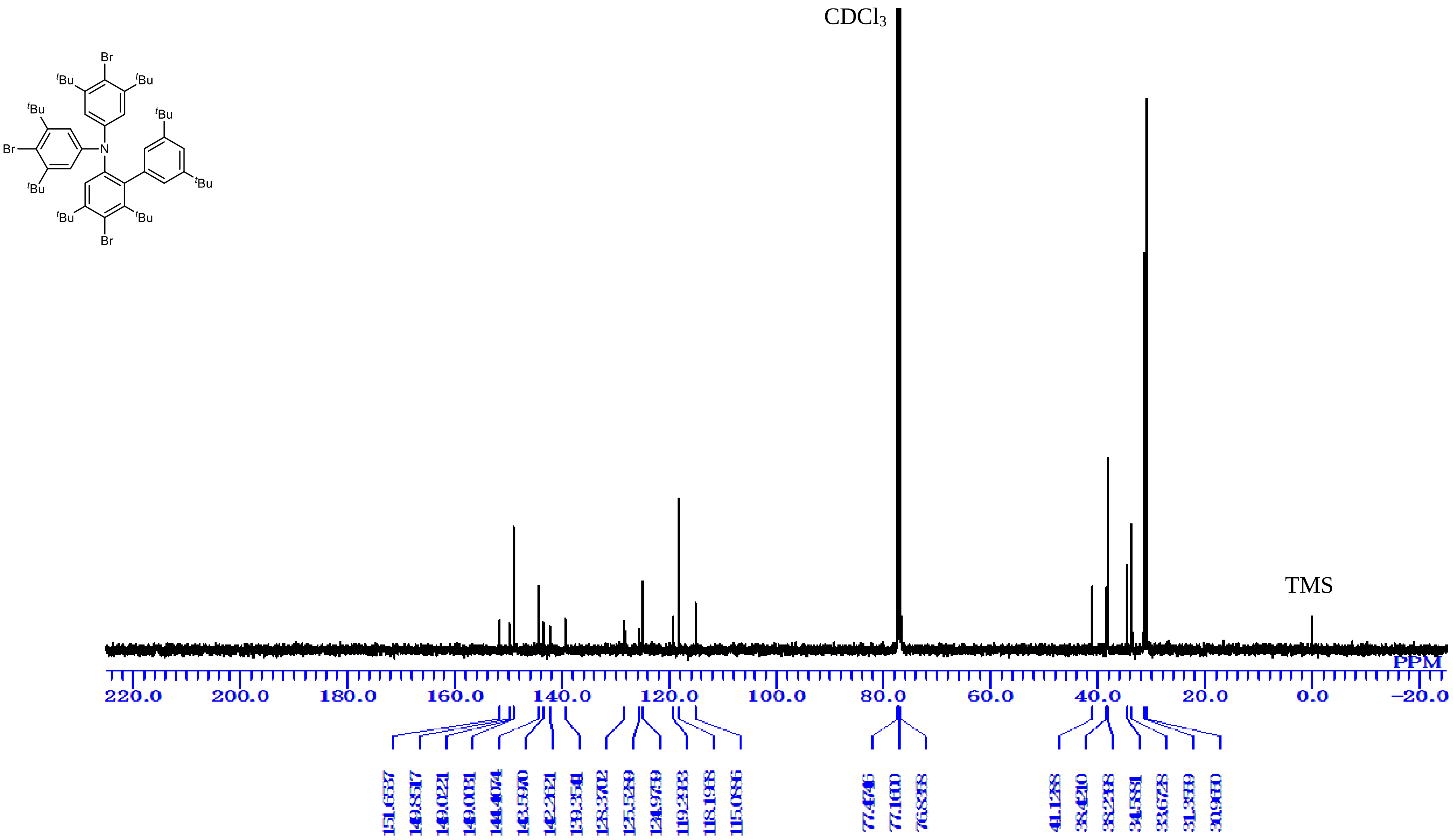

Supplementary Fig. 70 | ^{13}C NMR spectrum of bis[(4-bromo-3,5-di-*tert*-butyl)phenyl](5-bromo-3',4,5',6-tetra-*tert*-butyl-[1,1'-biphenyl]-2-yl)amine (18) (100 MHz, 20 °C, $CDCl_3$).

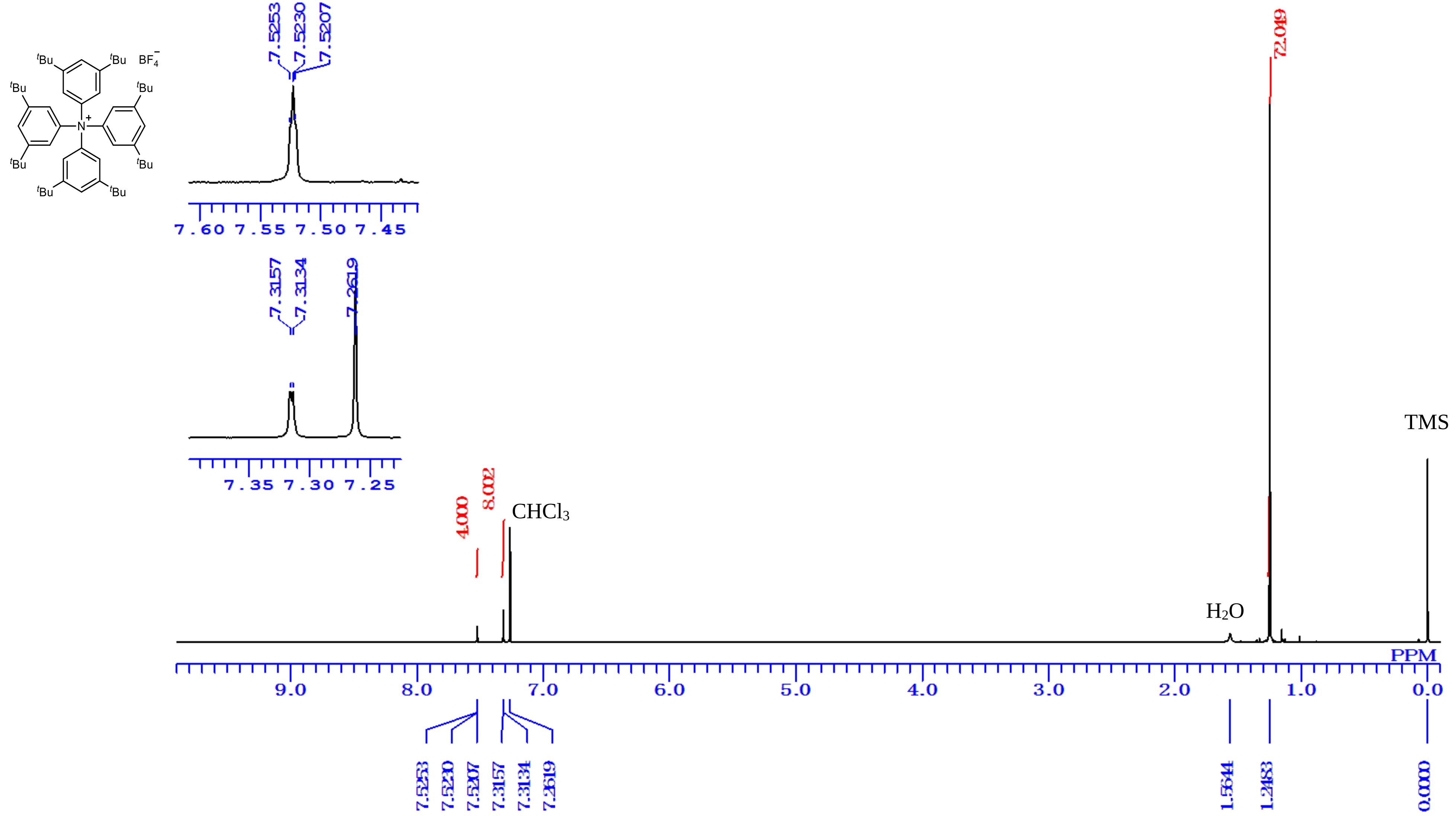

Supplementary Fig. 71 | ^{1}H NMR spectrum of tetrakis[(3,5-di-*tert*-butyl)phenyl]ammonium tetrafluoroborate (20) (600 MHz, 20 °C, $CDCl_3$).

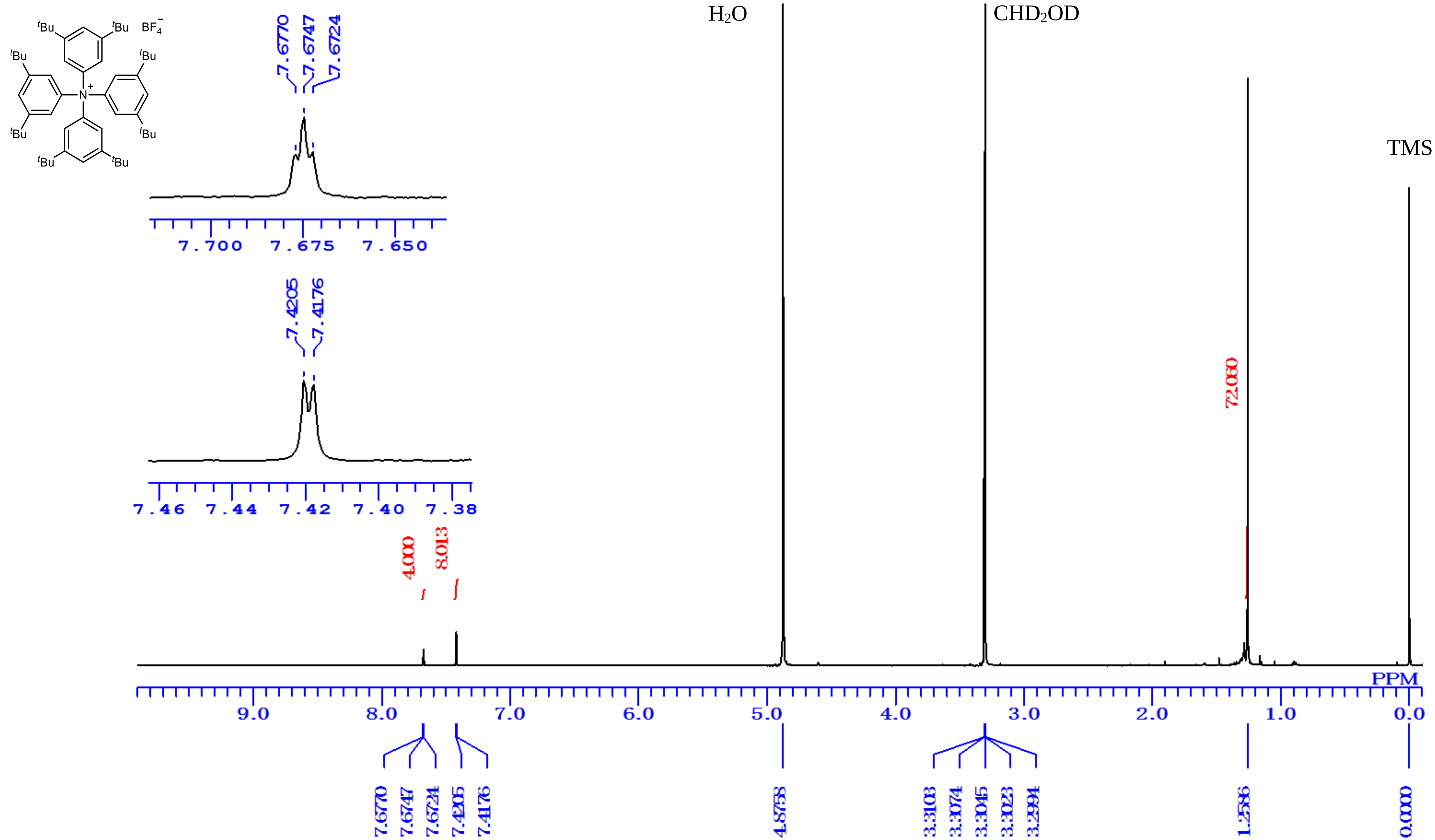

Supplementary Fig. 72 | ^{1}H NMR spectrum of tetrakis[(3,5-di-*tert*-butyl)phenyl]ammonium tetrafluoroborate (20) (600 MHz, 20 °C, CD_3OD).

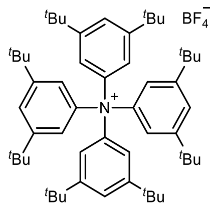

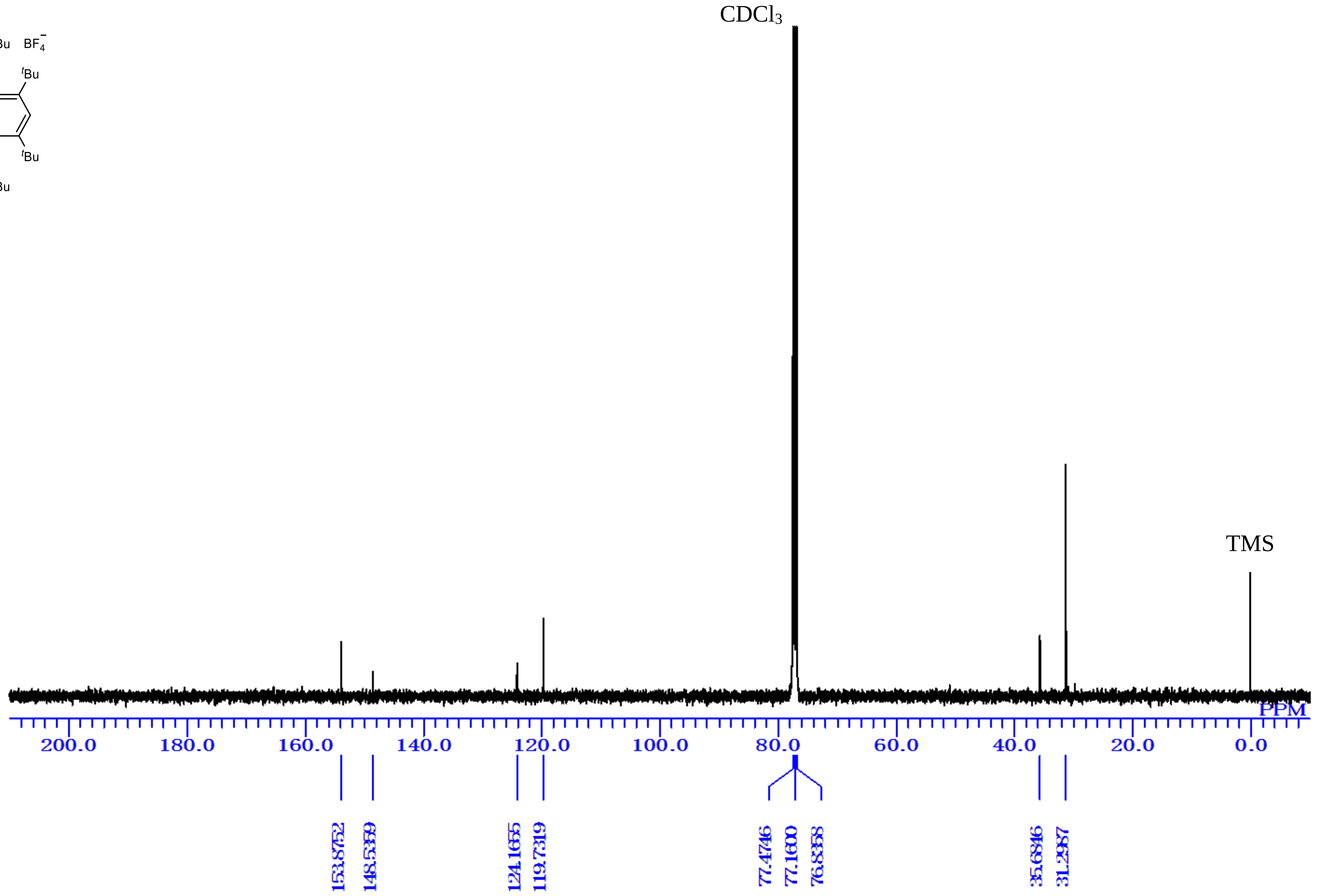

Supplementary Fig. 73 | ^{13}C NMR spectrum of tetrakis[(3,5-di-*tert*-butyl)phenyl]ammonium tetrafluoroborate (20) (100 MHz, 20 °C, $CDCl_3$).

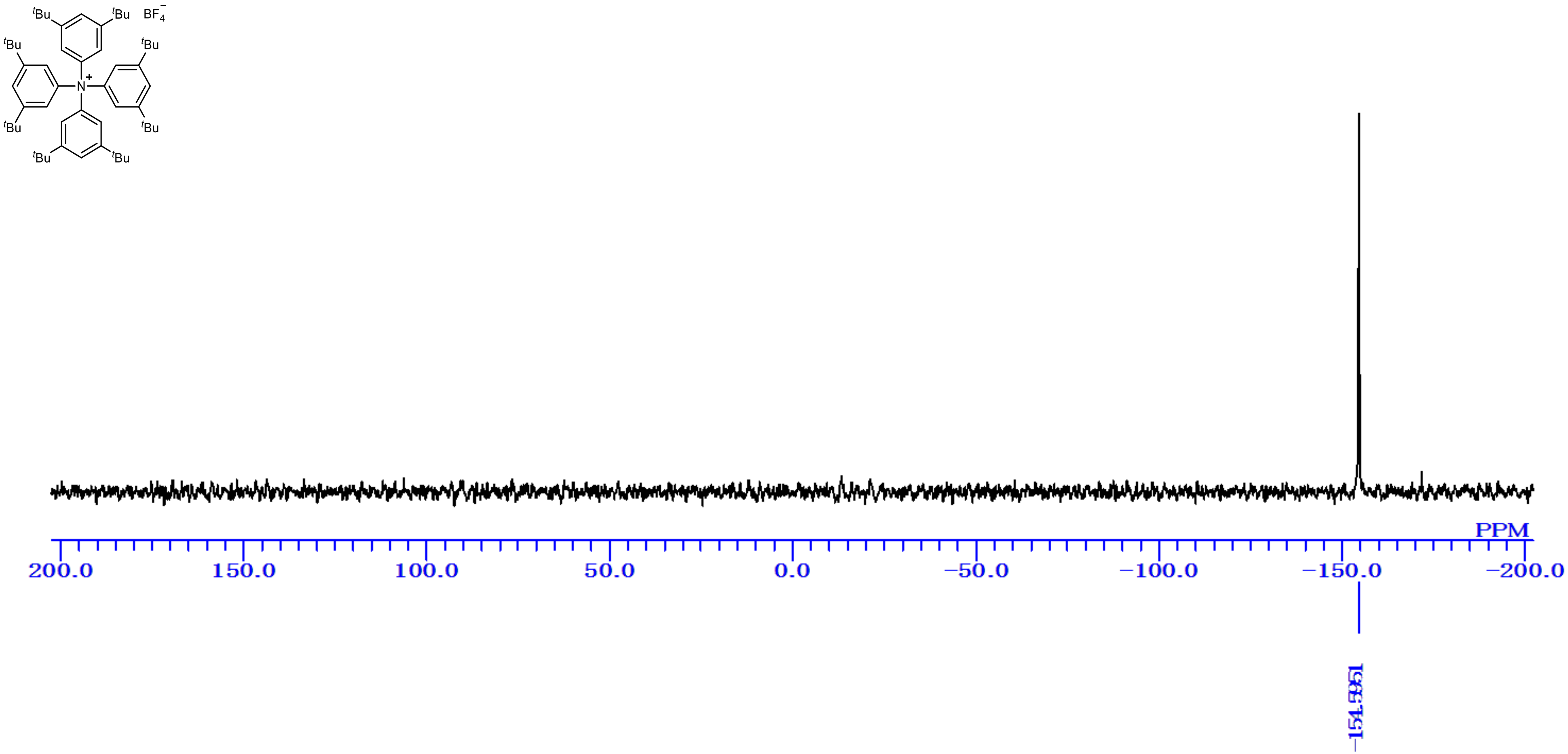

Supplementary Fig. 74 | ^{19}F NMR spectrum of tetrakis[(3,5-di-*tert*-butyl)phenyl]ammonium tetrafluoroborate (20) (565 MHz, 20 °C, CDCl$_3$).

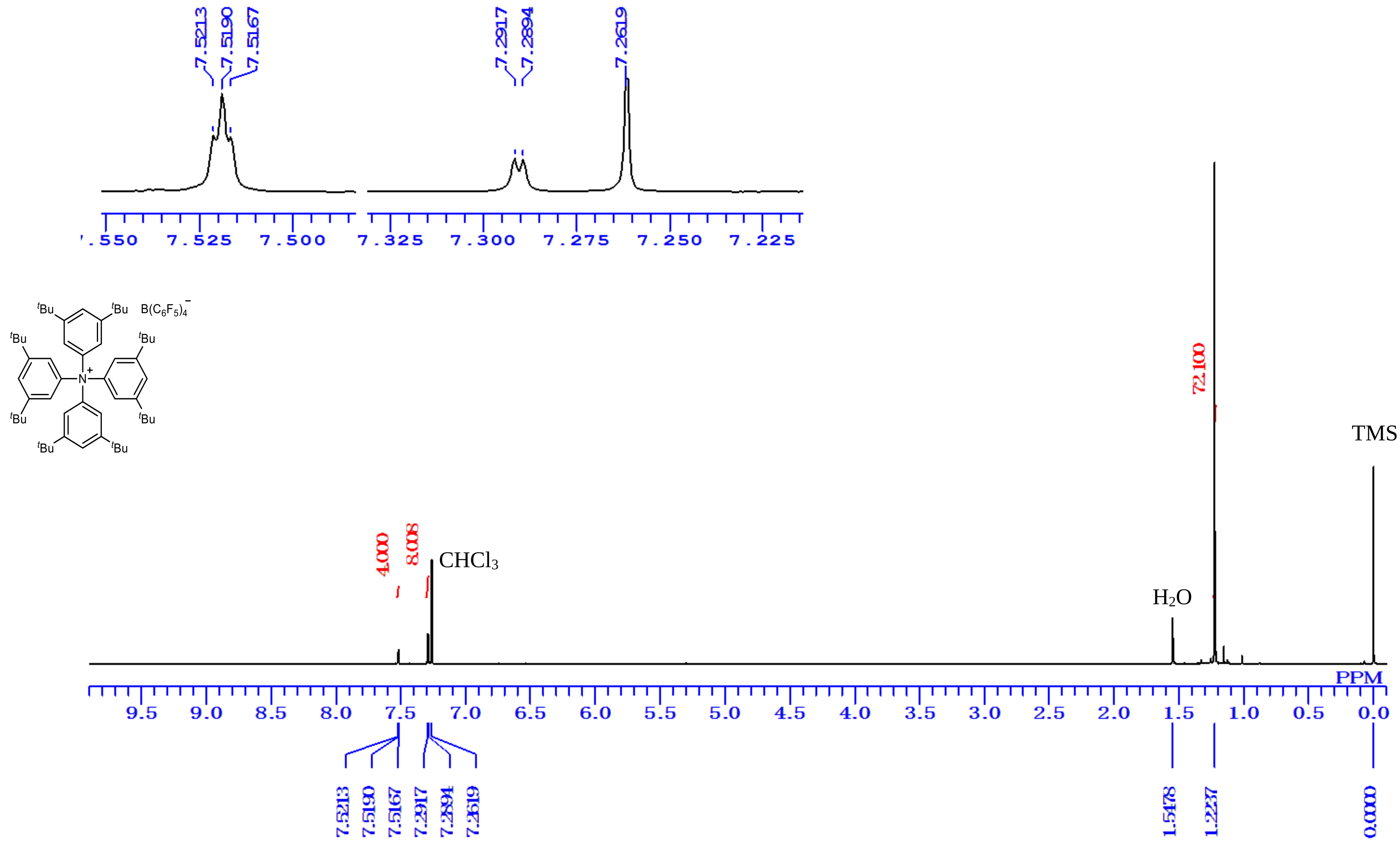

Supplementary Fig. 75 | ^{1}H NMR spectrum of tetrakis[(3,5-di-*tert*-butyl)phenyl]ammonium tetrakis(pentafluorophenyl)borate (22) (600 MHz, 20 °C, $CDCl_3$).

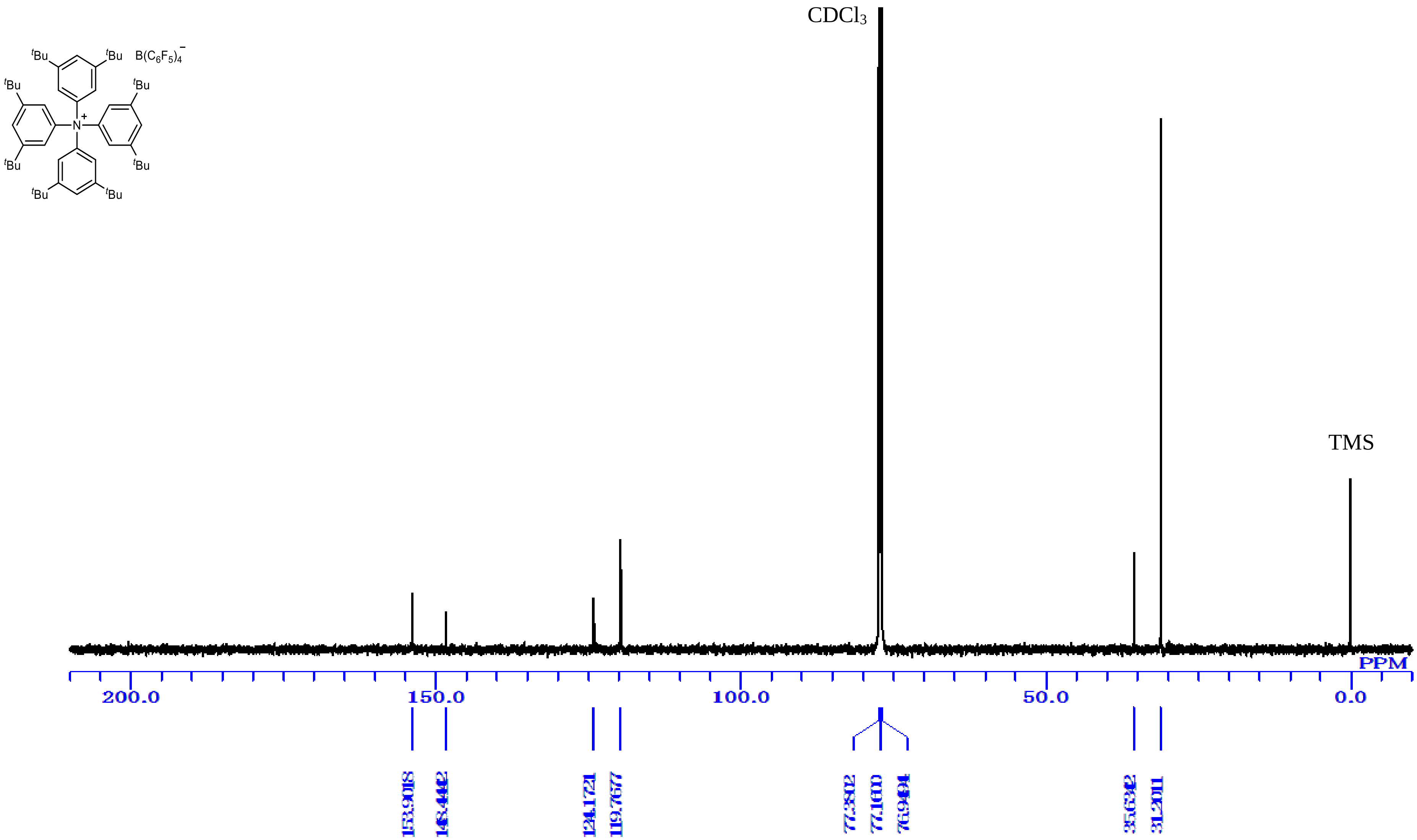

Supplementary Fig. 76 | ^{13}C NMR spectrum of tetrakis[(3,5-di-*tert*-butyl)phenyl]ammonium tetrakis(pentafluorophenyl)borate (22) (150 MHz, 20 °C, $CDCl_3$).

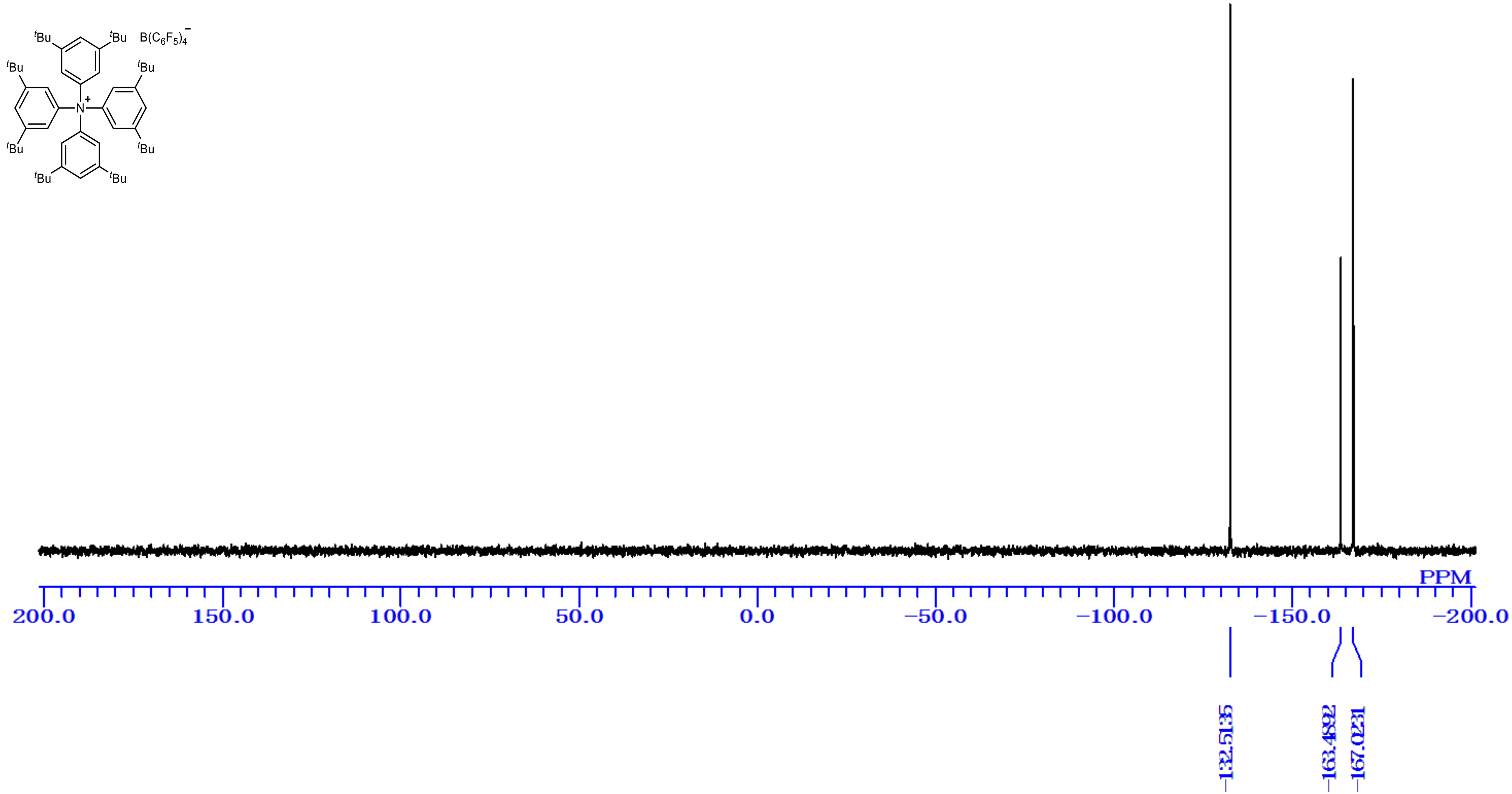

Supplementary Fig. 77 | ^{19}F NMR spectrum of tetrakis[(3,5-di-*tert*-butyl)phenyl]ammonium tetrakis(pentafluorophenyl)borate (22) (565 MHz, 20 °C, $CDCl_3$).

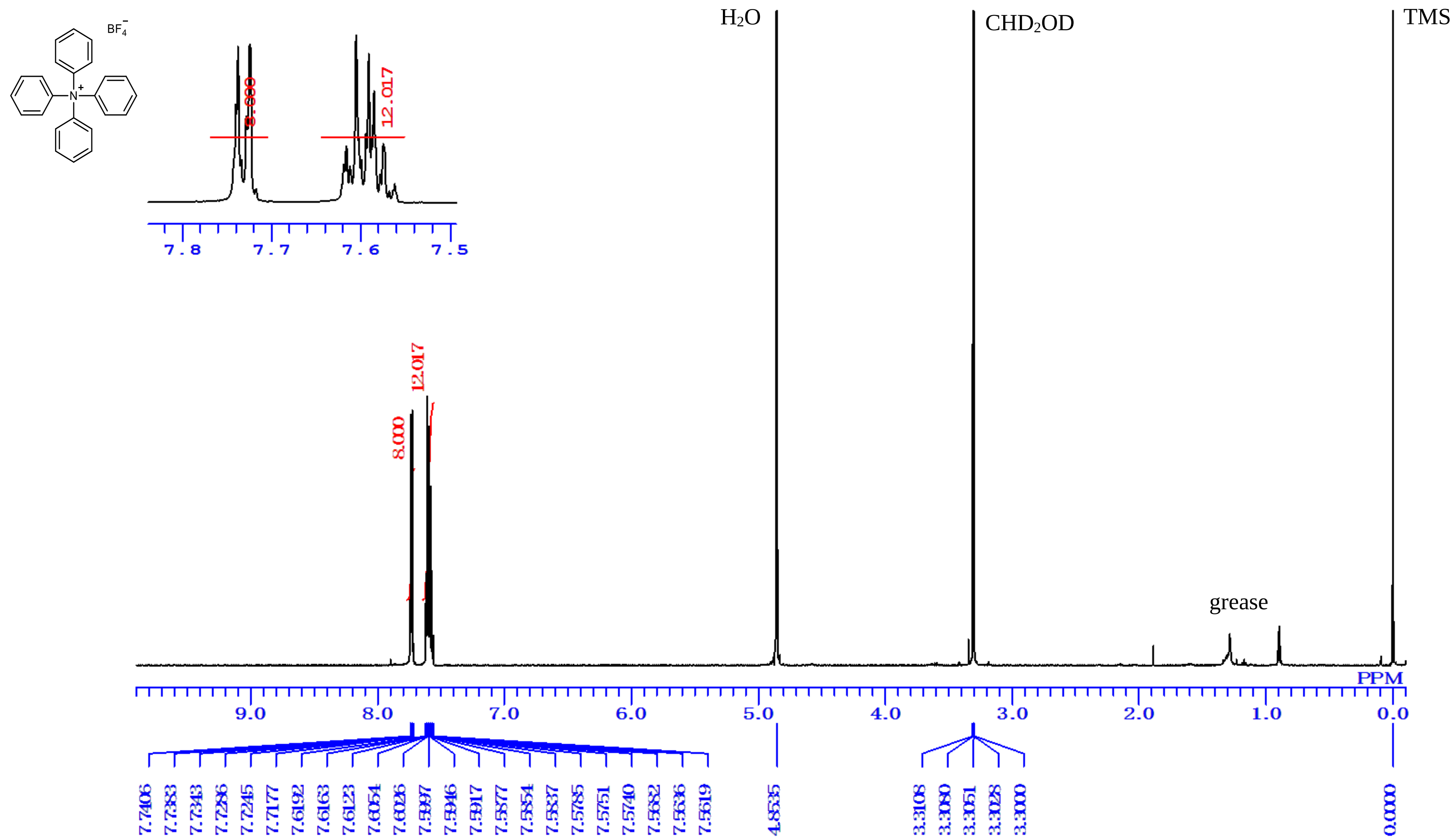

Supplementary Fig. 78 | ^{1}H NMR spectrum of tetraphenylammonium tetrafluoroborate (23) (600 MHz, 20 °C, CD_3OD).

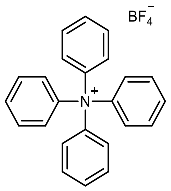

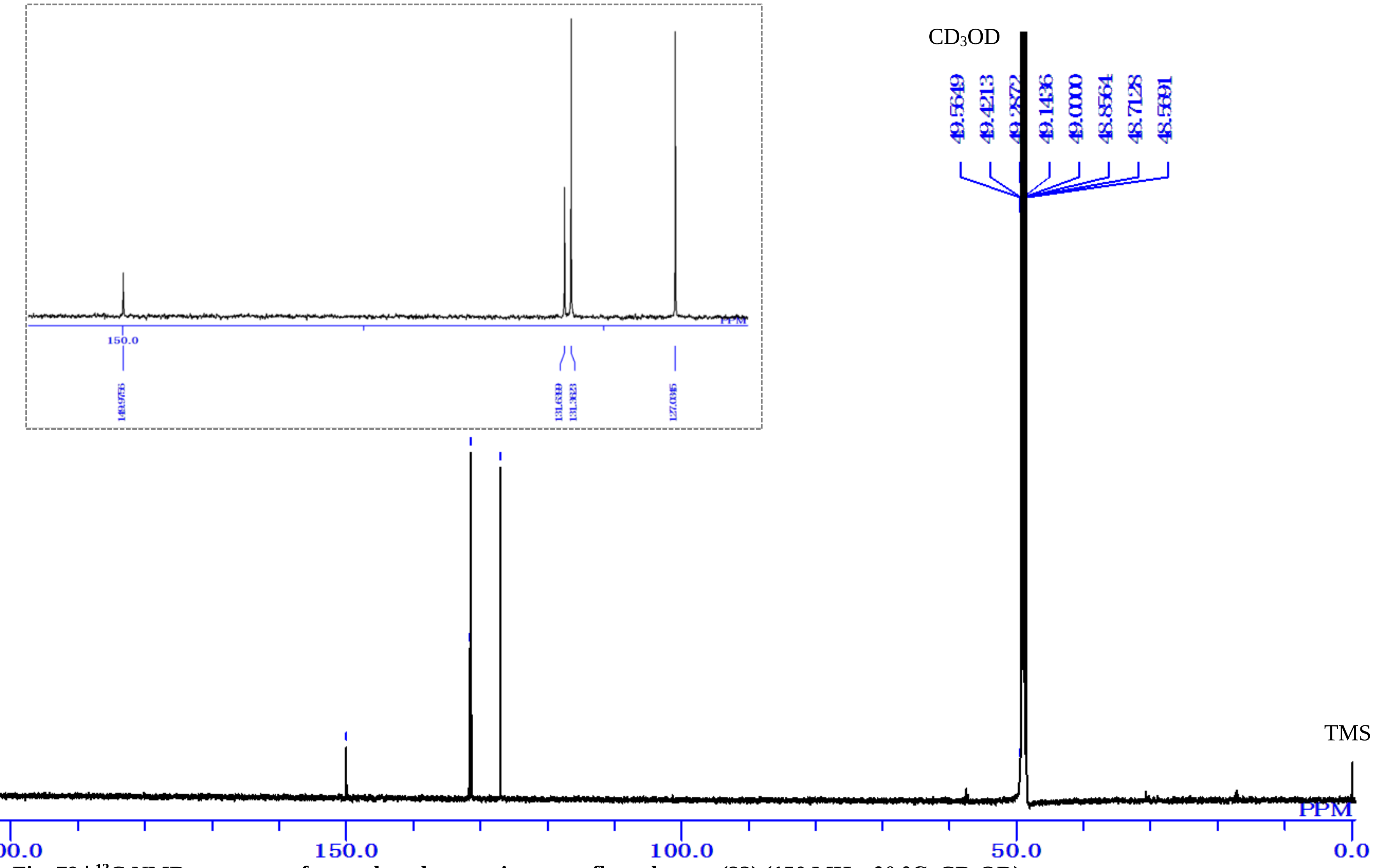

Supplementary Fig. 79 | ^{13}C NMR spectrum of tetraphenylammonium tetrafluoroborate (23) (150 MHz, 20 °C, CD_3OD).

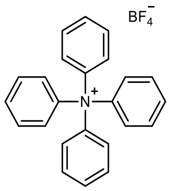

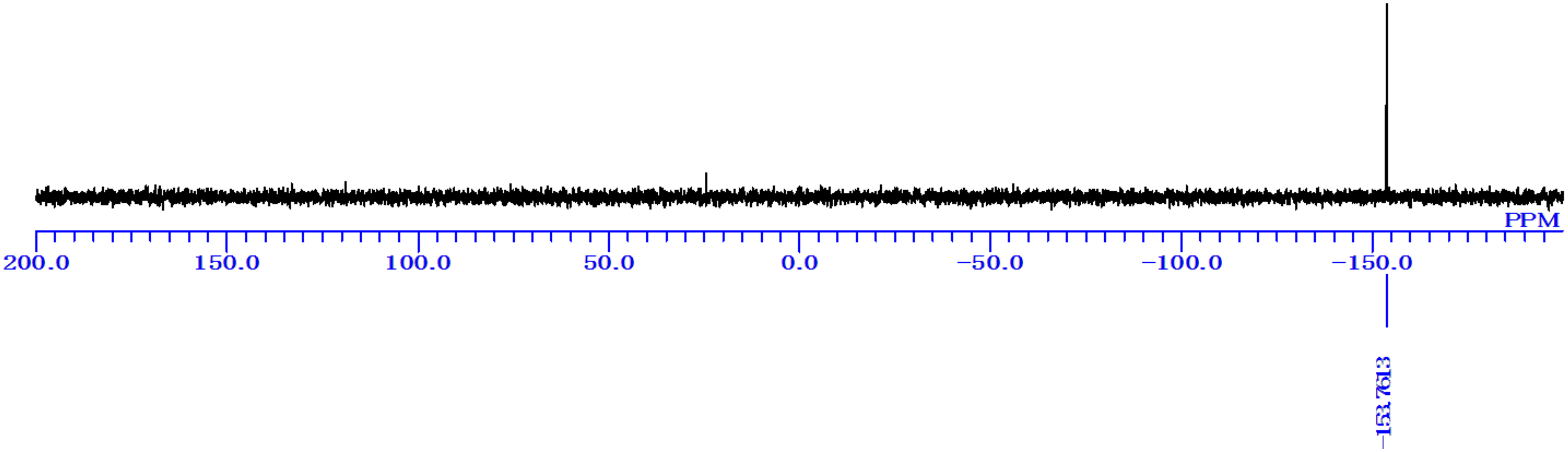

Supplementary Fig. 80 | ^{19}F NMR spectrum of tetraphenylammonium tetrafluoroborate (23) (565 MHz, 20 °C, $CDCl_3$).

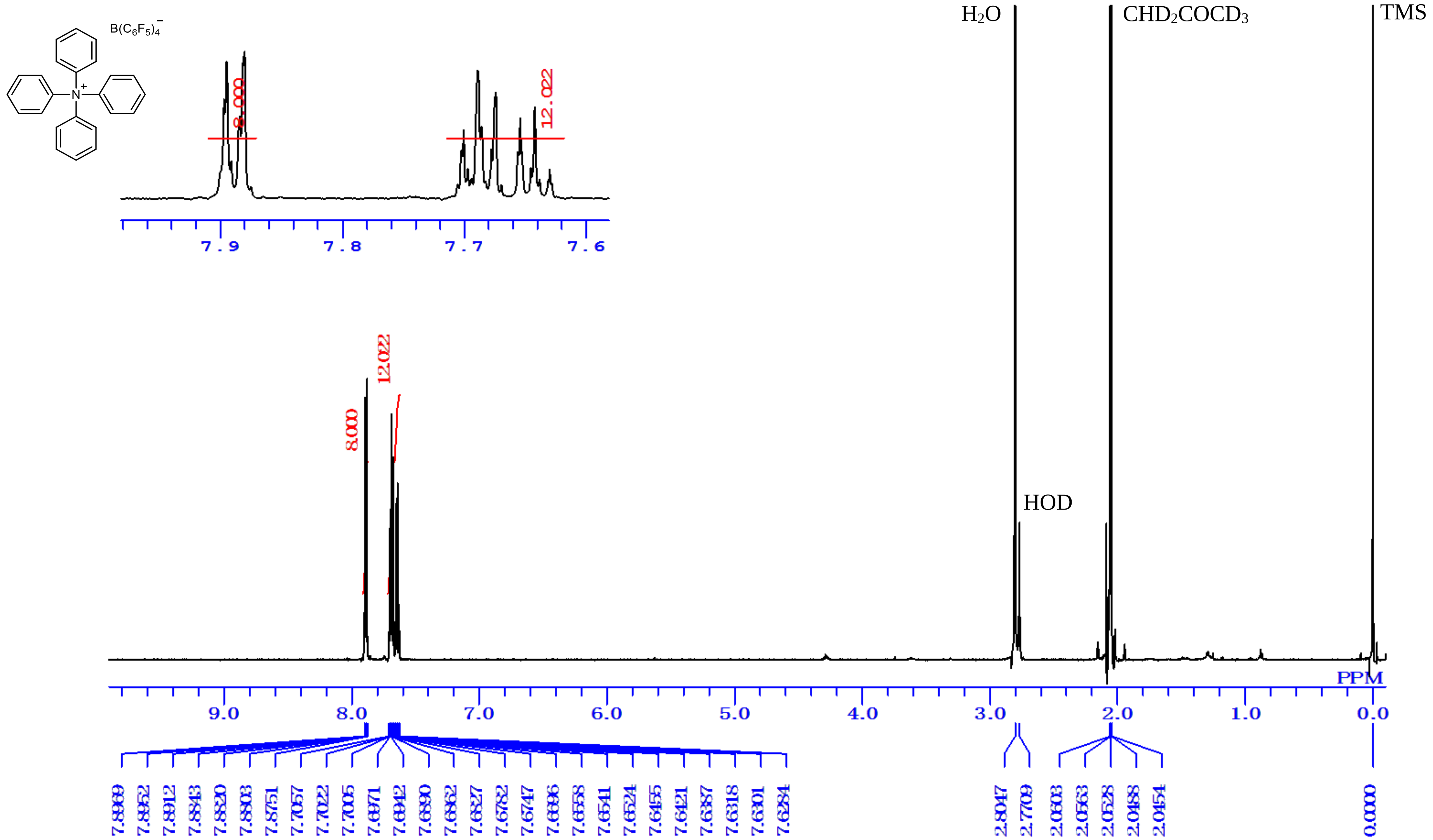

Supplementary Fig. 81 | ^{1}H NMR spectrum of tetraphenylammonium tetrakis(pentafluorophenyl)borate (24) [600 MHz, 20 °C, $(CD_3)_2CO$].

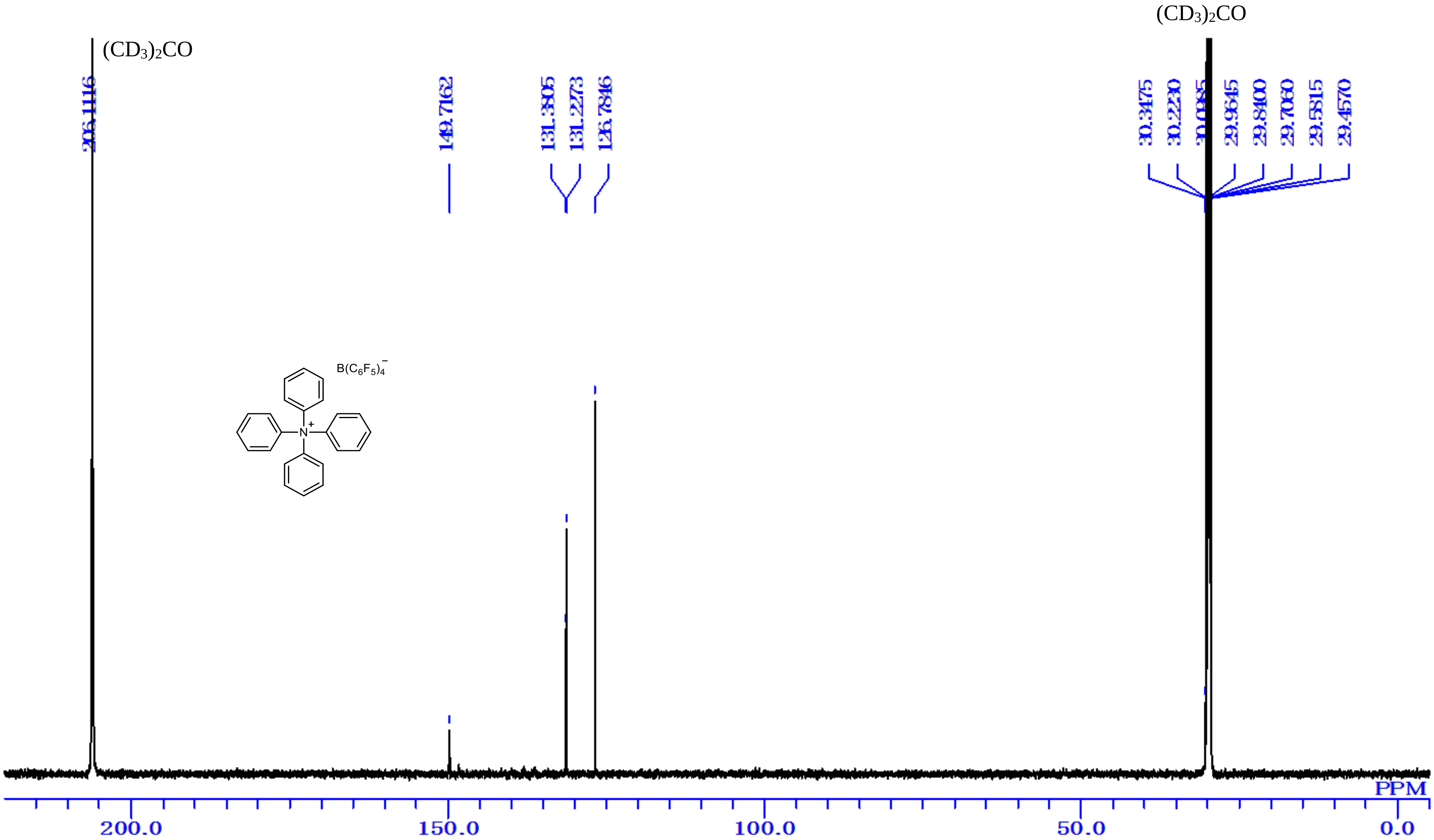

Supplementary Fig. 82 | ^{13}C NMR spectrum of tetraphenylammonium tetrakis(pentafluorophenyl)borate (24) [150 MHz, 20 °C, $(CD_3)_2CO$].

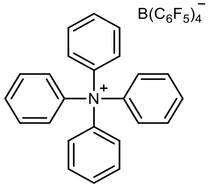

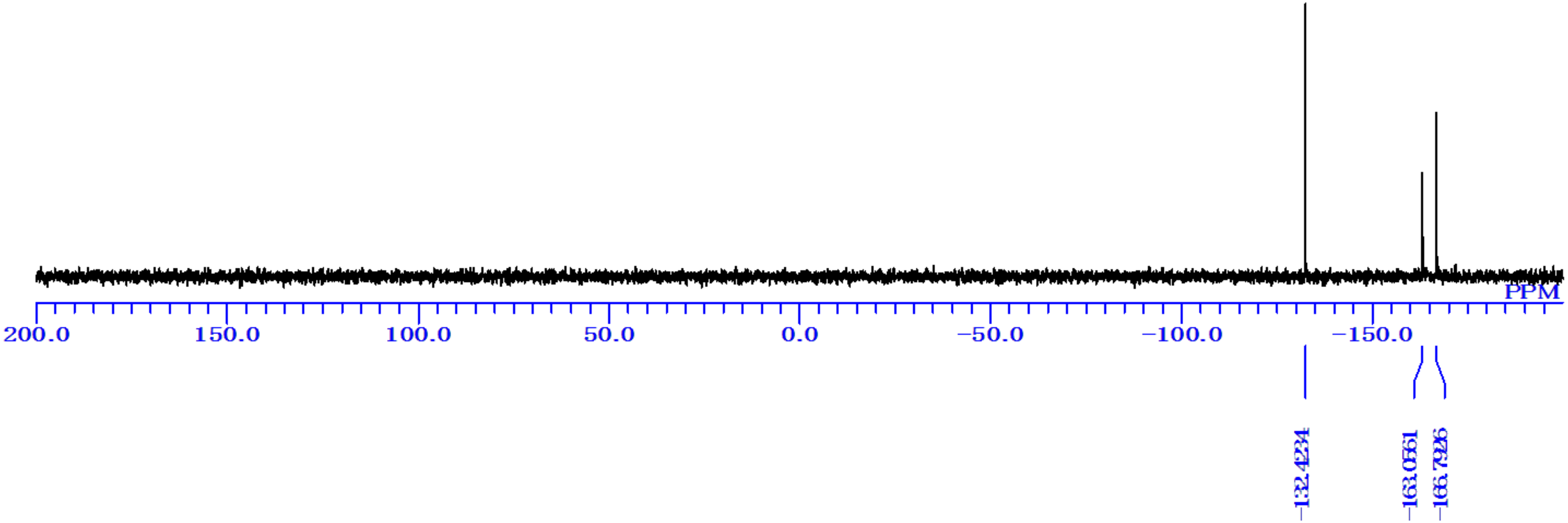

Supplementary Fig. 83 | ^{19}F NMR spectrum of tetraphenylammonium tetrakis(pentafluorophenyl)borate (24) (565 MHz, 20 °C, $CDCl_3$).

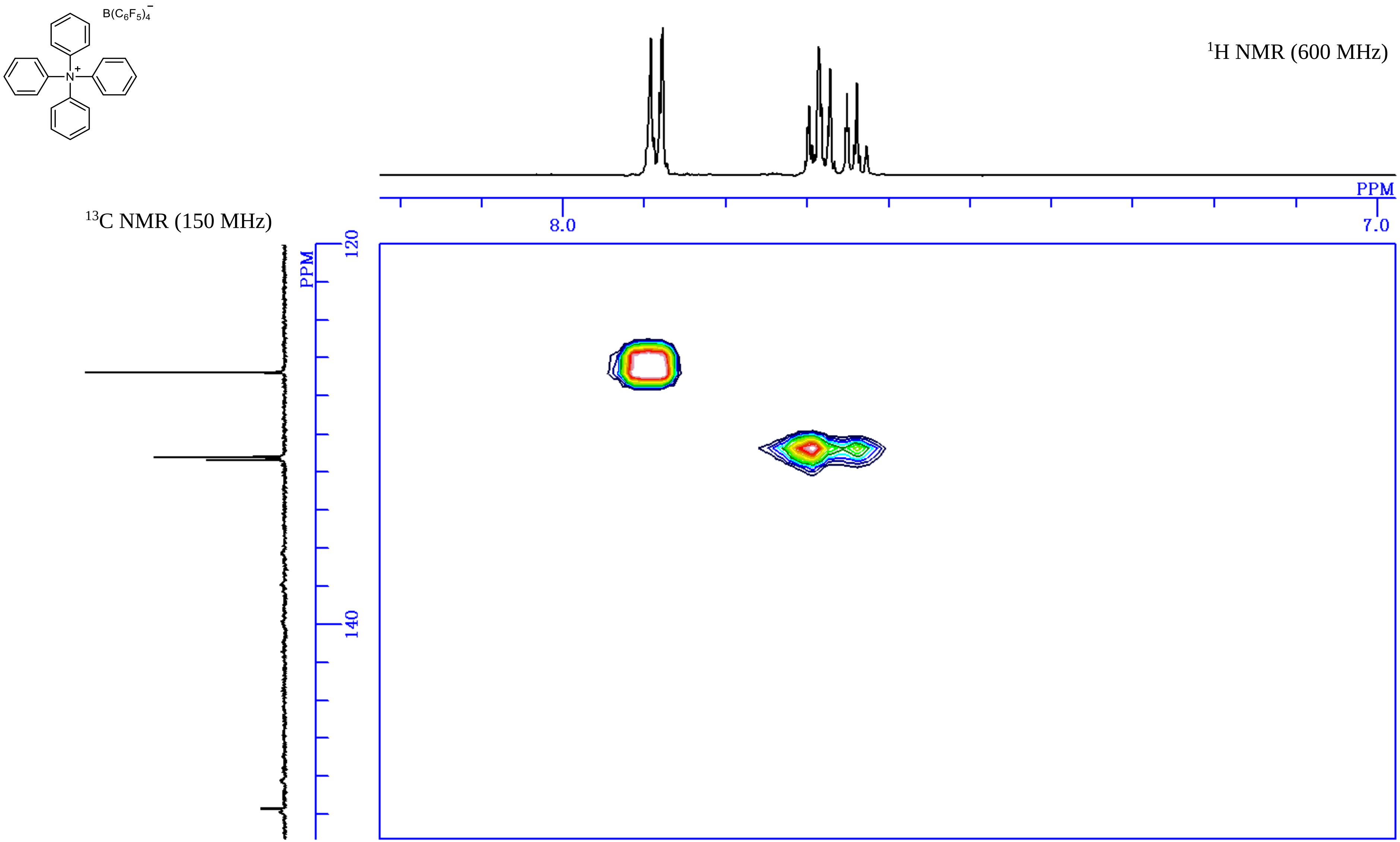

Supplementary Fig. 84 | HMQC spectrum of tetraphenylammonium tetrakis(pentafluorophenyl)borate (24) [20 °C, $(CD_3)_2CO$].

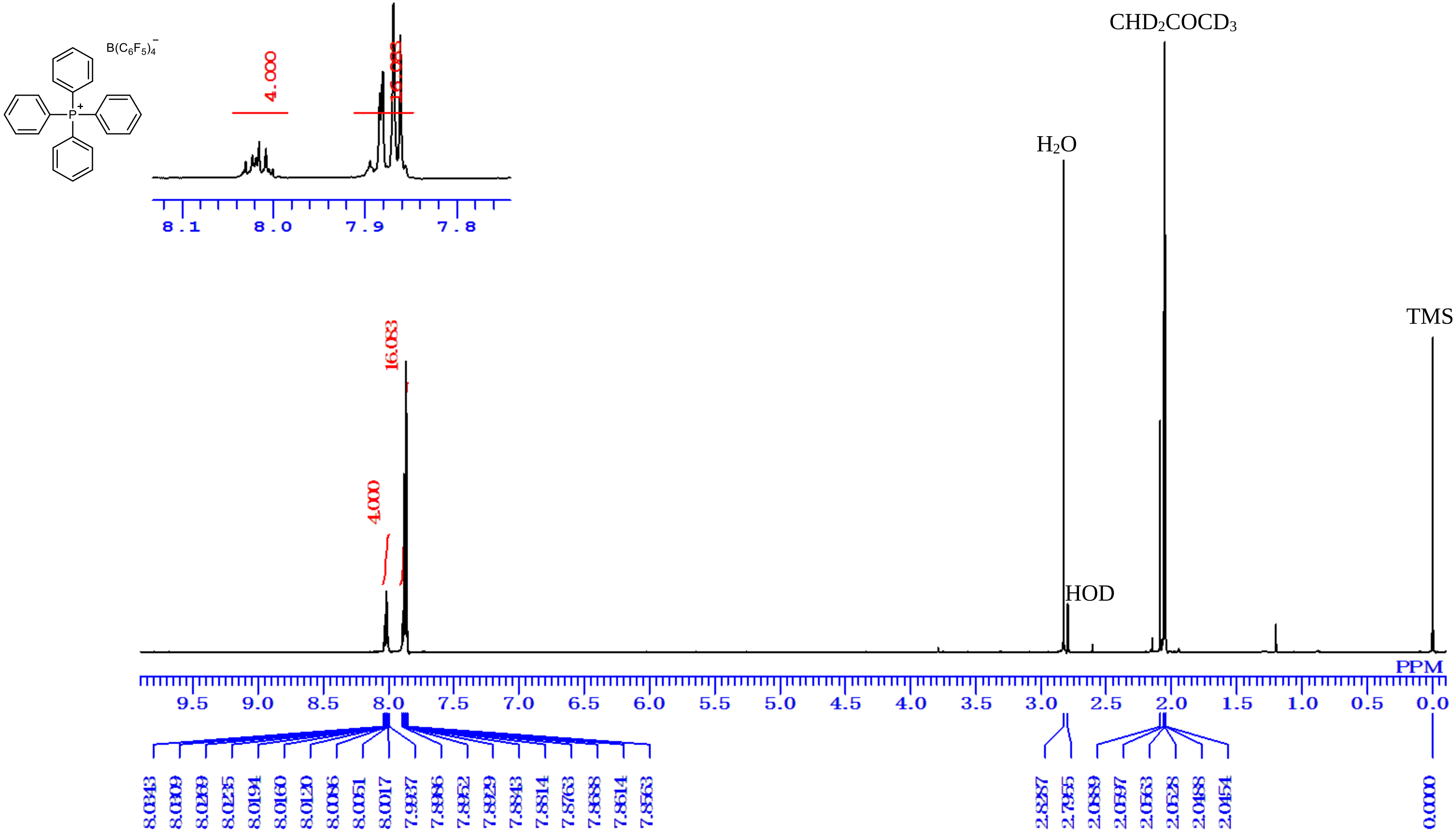

Supplementary Fig. 85 | ^{1}H NMR spectrum of tetraphenylphosphonium tetrakis(pentafluorophenyl)borate (25) [600 MHz, 20 °C, $(CD_3)_2CO$].

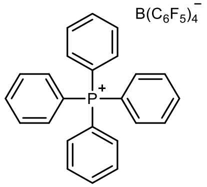

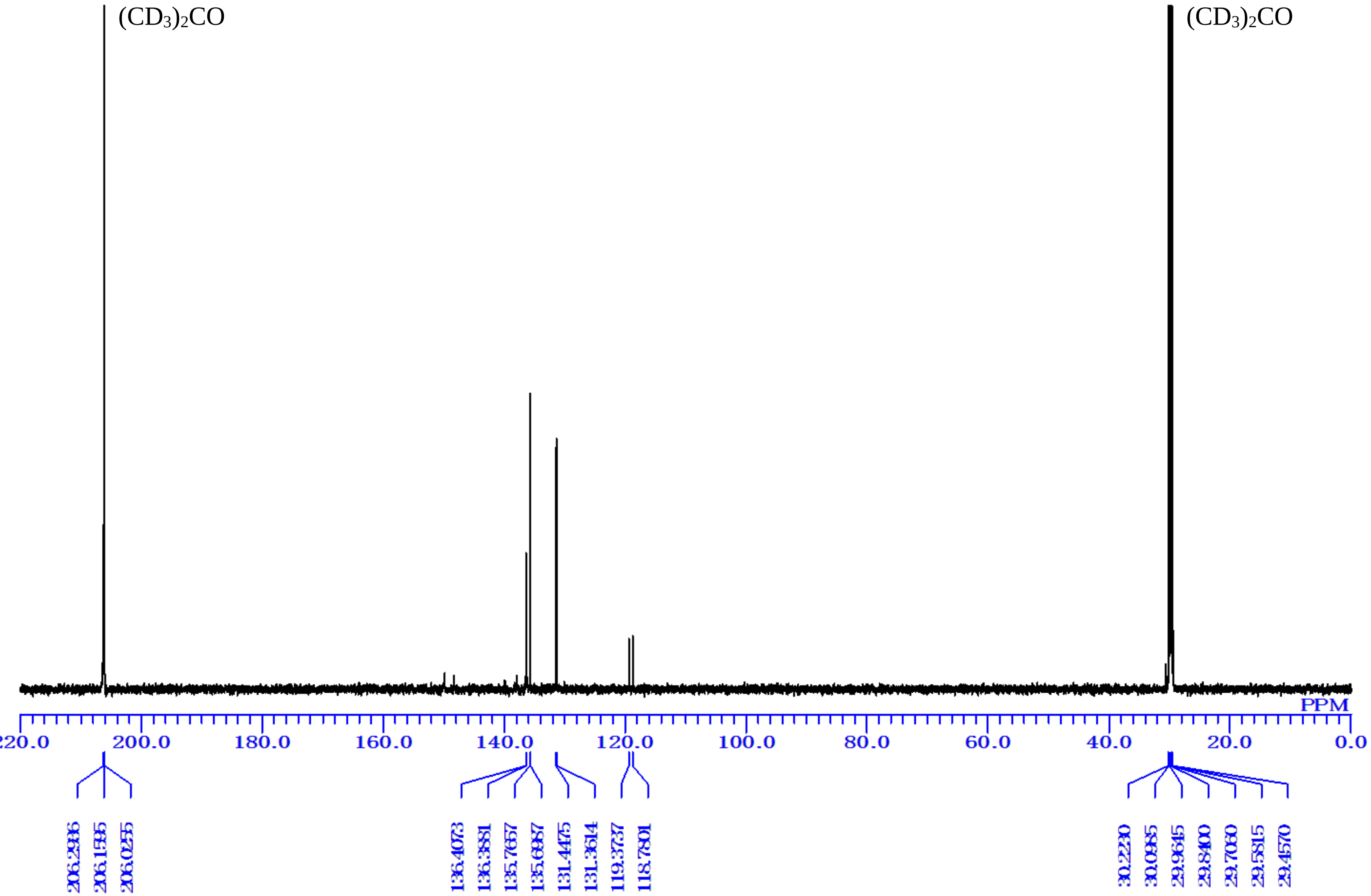

Supplementary Fig. 86 | ^{13}C NMR spectrum of tetraphenylphosphonium tetrakis(pentafluorophenyl)borate (25) [150 MHz, 20 °C, $(CD_3)_2CO$].

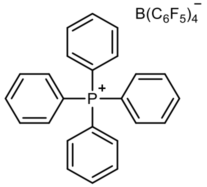

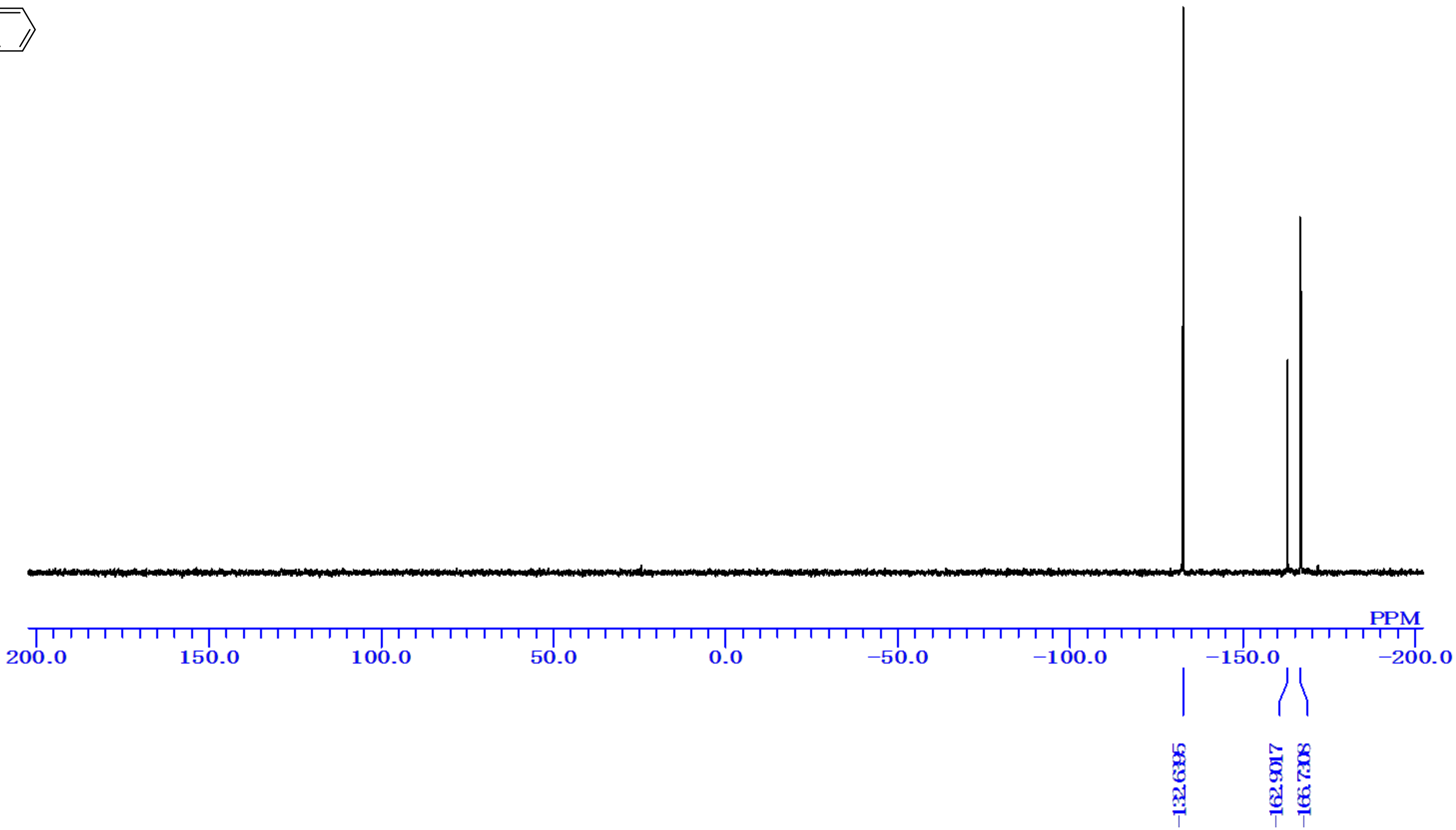

Supplementary Fig. 87 | ^{19}F NMR spectrum of tetraphenylphosphonium tetrakis(pentafluorophenyl)borate (25) [565 MHz, 20 °C, $CDCl_3$].

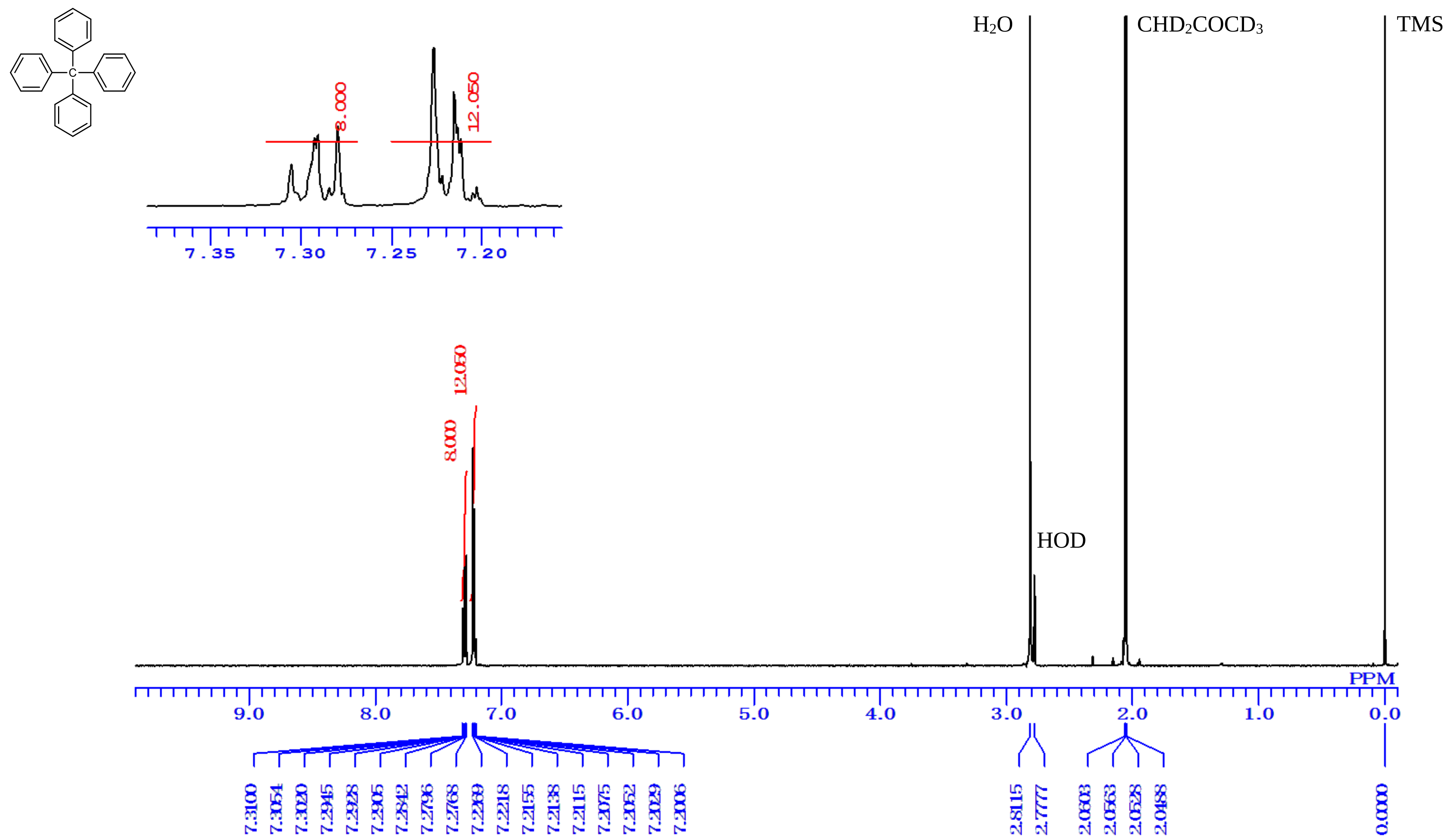

Supplementary Fig. 88 | ^{1}H NMR spectrum of tetraphenylmethane [600 MHz, 20 °C, $(CD_3)_2CO$].

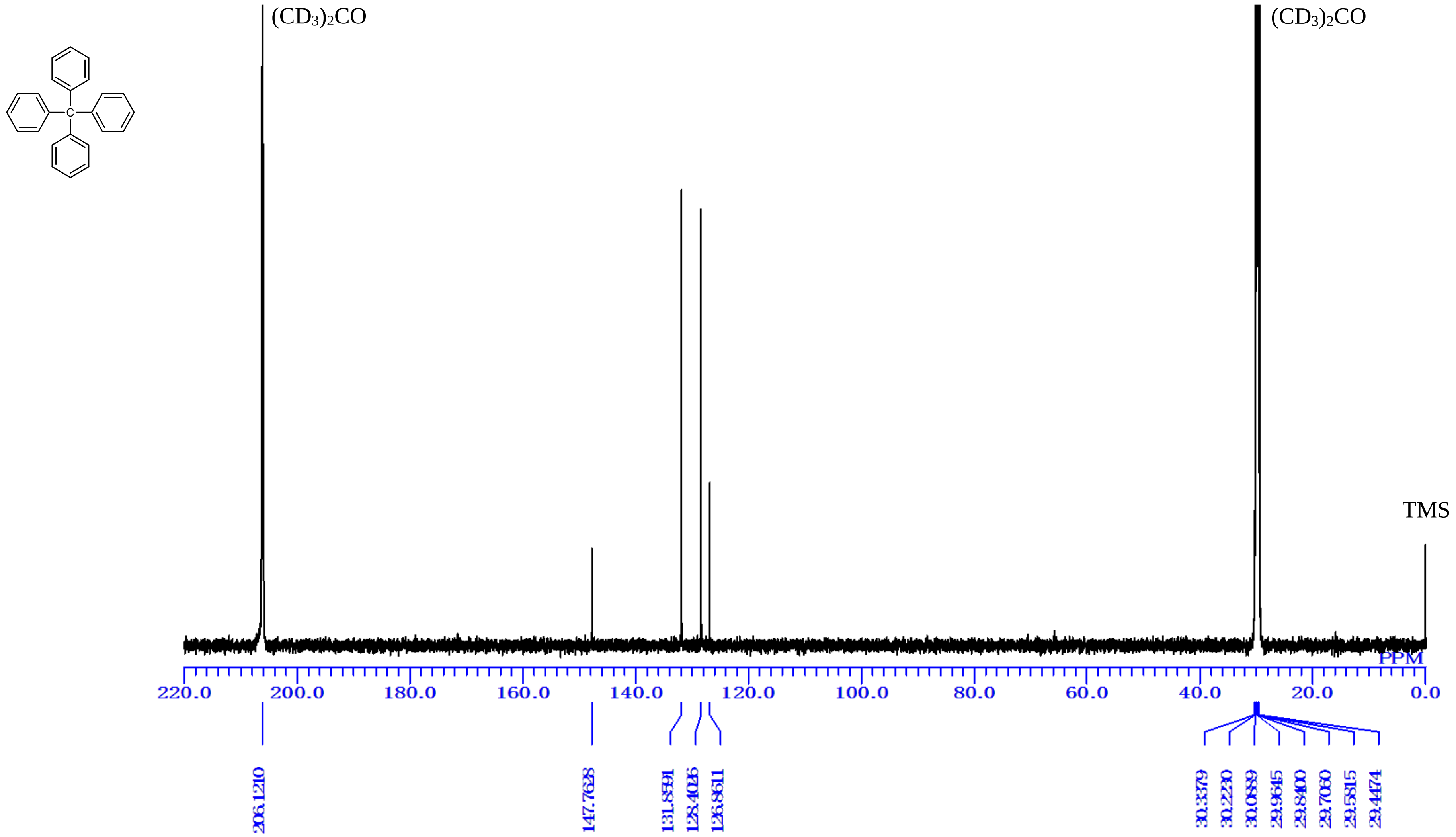

Supplementary Fig. 89 | ^{13}C NMR spectrum of tetraphenylmethane[150 MHz, 20 °C, $(CD_3)_2CO$].

(The sp^3 carbon signal of this compound was not clearly observed because of its limited solubility in the solvent.)

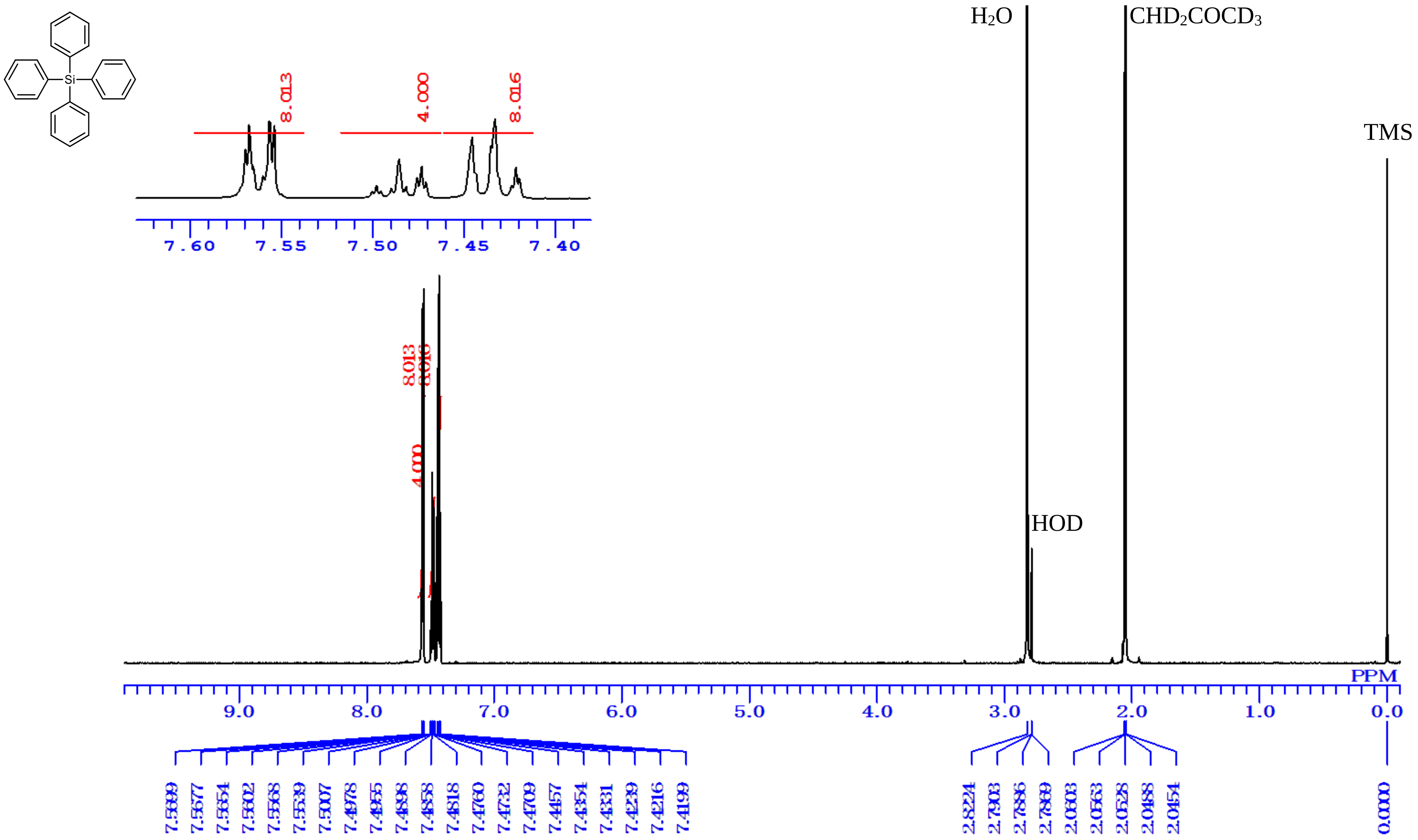

Supplementary Fig. 90 | ^{1}H NMR spectrum of tetraphenylsilane [600 MHz, 20 °C, $(CD_3)_2CO$].

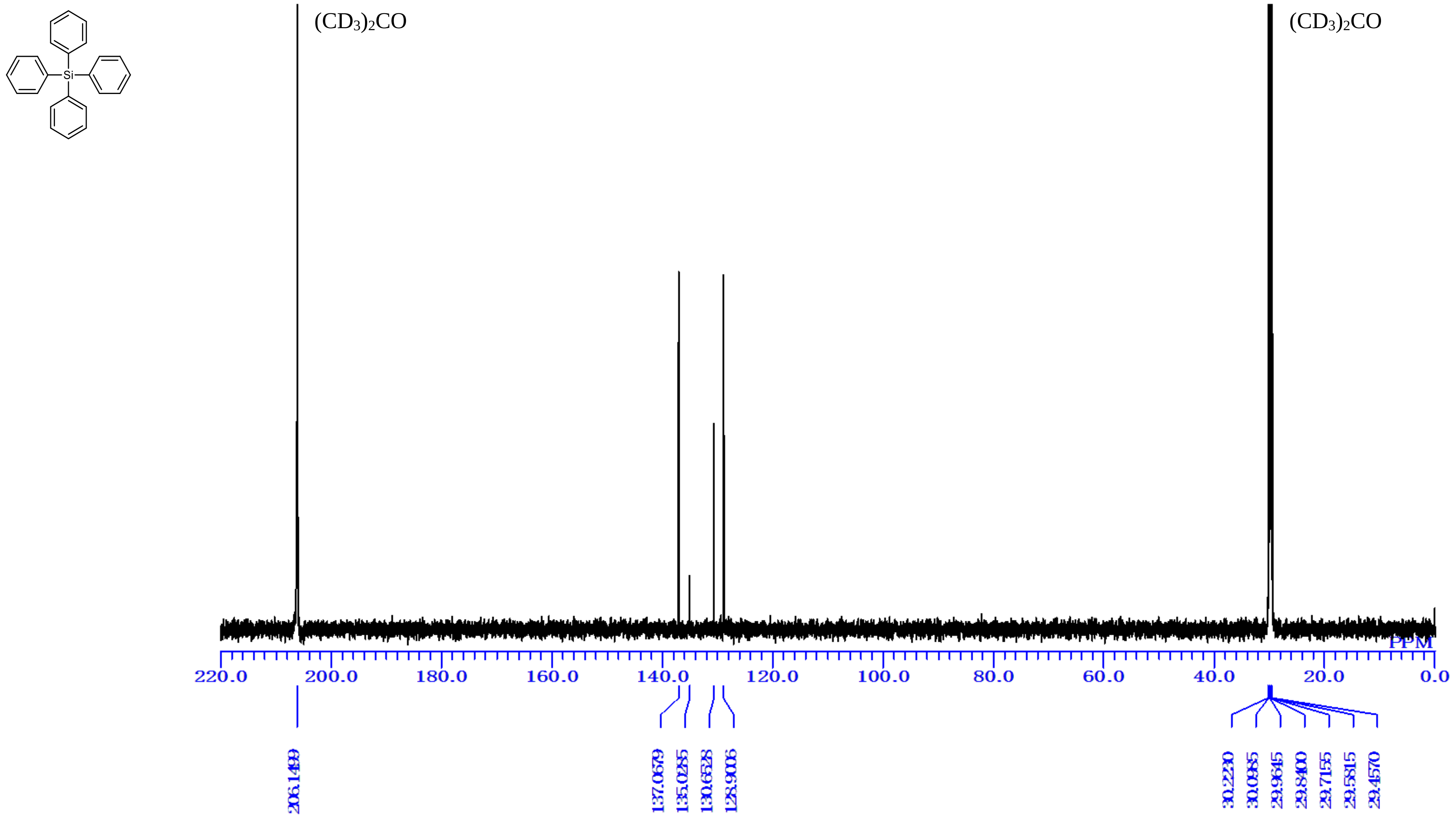

Supplementary Fig. 91 | ^{13}C NMR spectrum of tetraphenylsilane [150 MHz, 20 °C, $(CD_3)_2CO$].

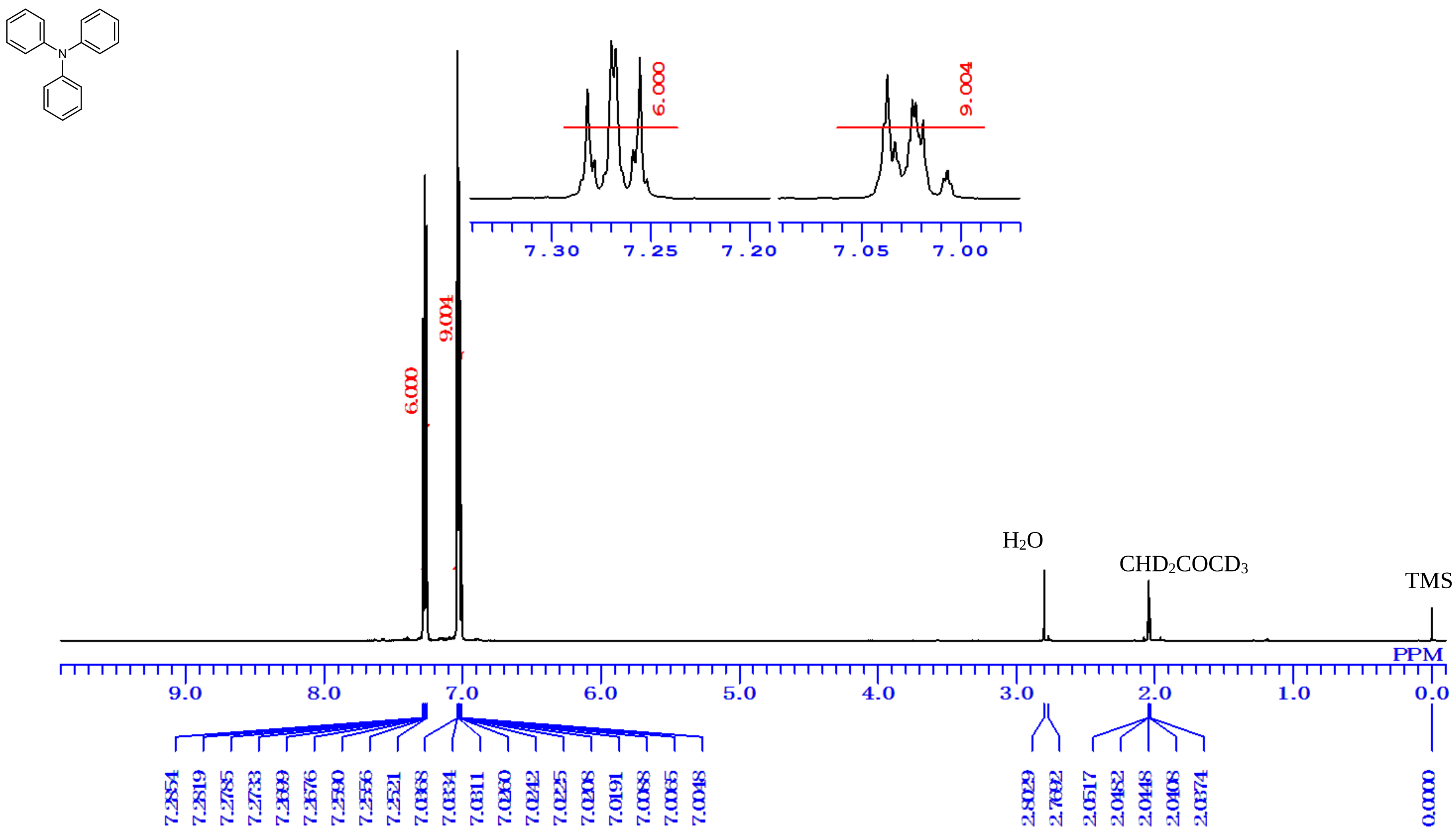

Supplementary Fig. 92 | ^{1}H NMR spectrum of triphenylamine (8) [600 MHz, 20 °C, $(CD_3)_2CO$].

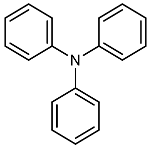

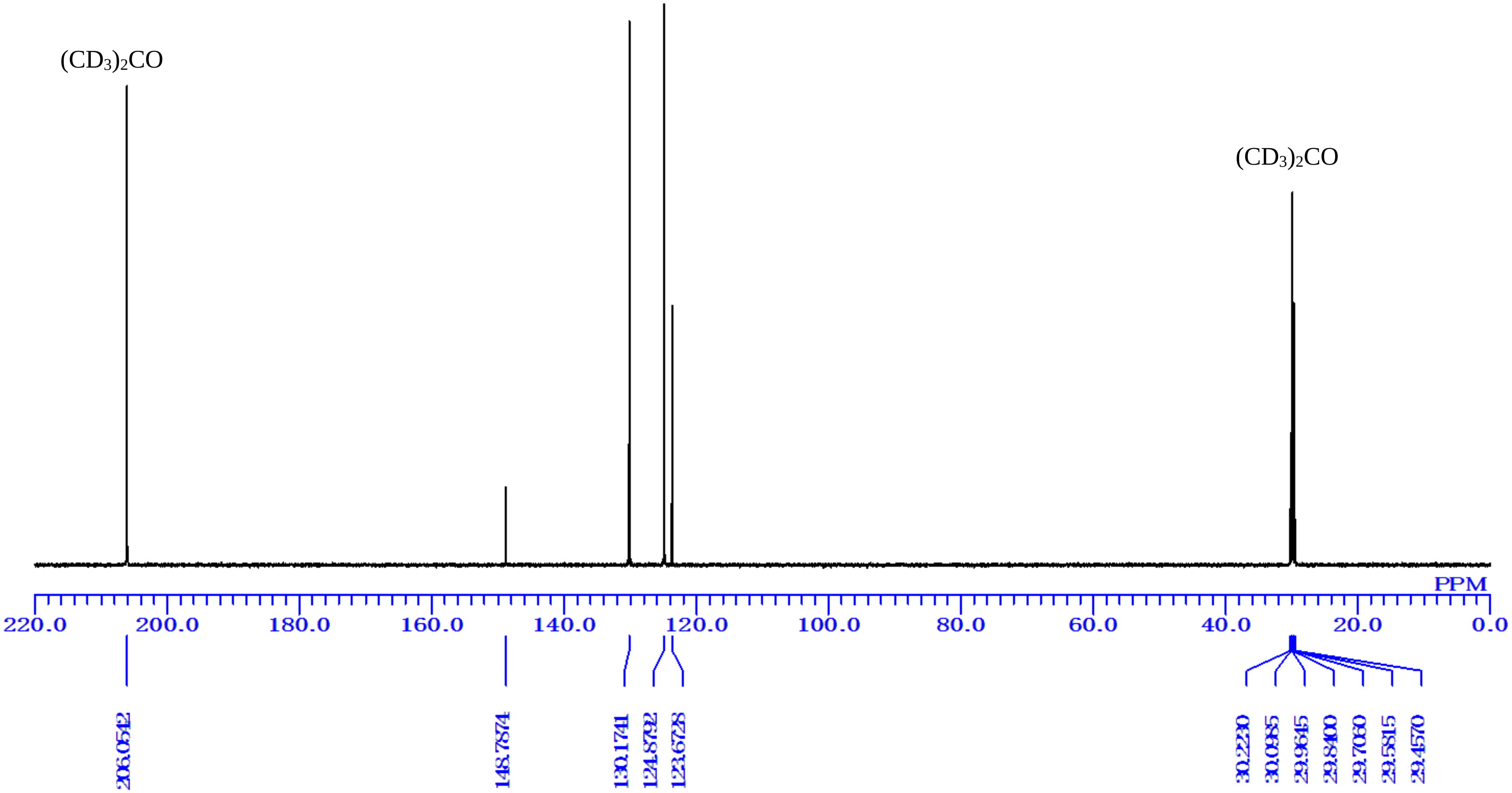

Supplementary Fig. 93 | ^{13}C NMR spectrum of triphenylamine (8) [150 MHz, 20 °C, $(CD_3)_2CO$].

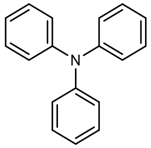

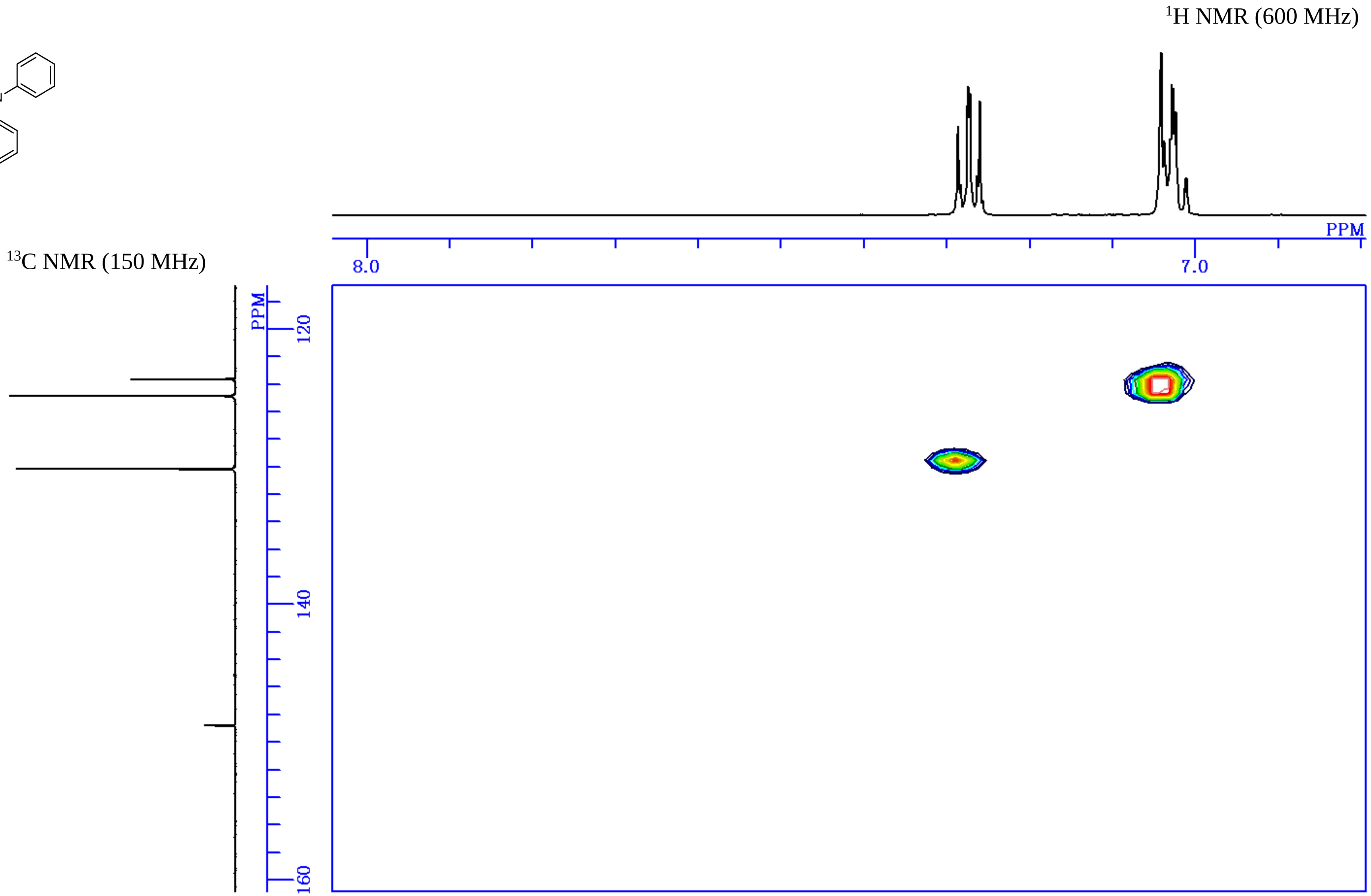

Supplementary Fig. 94 | HMQC spectrum of triphenylamine (8) [20 °C, $(CD_3)_2CO$].

5. Supplementary References

1. Deno, N. C. & Berkheimer, H. E. Solubilities of organic salts in hydrocarbons. *J. Org. Chem.* **28**, 2143–2144 (1963).
2. Evans, A. G., Evans, J. C. & Pomery, P. J. Reactions of radical anions. Part XIV. An electron spin resonance study of the radical anions of 9,9′-azophenanthrene and 9,9′-azoanthracene. *J. Chem. Soc., Perkin Trans. 2* 1385–1388 (1974).
3. Akutsu, H. & Seelig, J. Interaction of metal ions with phosphatidylcholine bilayer membranes. *Biochemistry* **20**, 7366–7373 (1981).
4. Röper, M., Loevenich, H. & Korff, J. Methanol homologation by cobalt-phosphine-iodine catalyst systems. *J. Mol. Catal.* **17**, 315–322 (1982).
5. Seelig, J. Binding of metal ions to phospholipid membranes. Application of deuterium magnetic resonance. *Inorganica Chim. Acta* **79**, 30 (1983).
6. Desir, G. V., Cragoe, E. J. & Aronson, P. S. High affinity binding of amiloride analogs at an internal site in renal microvillus membrane vesicles. *J. Biol. Chem.* **266**, 2267–2271 (1991).
7. El-Shahawi, M. S., Abu Zuhri, A. Z. & Al-Daheri, S. M. Spectrophotometric determination of ruthenium after extraction of perruthenate with benzyltributylammonium chloride. *Fresenius. J. Anal. Chem.* **350**, 674–677 (1994).
8. El-Shahawi, M. S. & Aldhaheri, S. M. Spectrophotometric determination of bismuth(III and V) in water after ion-pair liquid–liquid extraction using tetramethylammonium cation as counter ion. *Fresenius. J. Anal. Chem.* **354**, 200–203 (1996).
9. Stadlober, M., Kalcher, K., Raber, G. & Neuhold, C. Anodic stripping voltammetric determination of titanium(IV) using a carbon paste electrode modified with cetyltrimethylammonium bromide. *Talanta* **43**, 1915–1924 (1996).
10. Zeng, B. & Purdy, W. C. Influence of cetyltrimethylammonium bromide on the voltammetric behavior of thiopurines at a silver electrode. *Electroanalysis* **11**, 879–884 (1999).
11. Okamoto, M., Sugiyama, J.-I. & Takeuchi, K. Halogen-free solvent for oxidative carbonylation of bisphenol A to polycarbonate. *J. Appl. Polym. Sci.* **106**, 2840–2842 (2007).
12. Goudarzi, N. Silicon-29 NMR spectroscopy study of the effect of tetraphenylammonium (TPA) as a template on distribution of silicate species on alkaline aqueous and alcoholic silicate solutions. *Appl. Magn. Reson.* **44**, 469–478 (2013).
13. Goudarzi, N. ^{27}Al NMR study of the effect of aqueous and methanolic media on the distribution of tetraphenylammonium aluminosilicate species. *J. Struct. Chem.* **56**, 250–258 (2015).
14. Goudarzi, N. & Amin, A. H. Application of phosphorus-31 and aluminum-27 NMR spectroscopic techniques to study aqueous and methanolic solutions of tetraphenylammonium aluminophosphate. *J. Mol. Struct.* **1128**, 338–344 (2017).
15. Sheldrick, G. M. *SHELXT* – Integrated space-group and crystal-structure determination. *Acta Crystallogr. Sect. A Found. Crystallogr.* **71**, 3–8 (2015).

16. Burla, M. C. *et al.* Crystal structure determination and refinement via SIR2014. *J. Appl. Crystallogr.* **48**, 306–309 (2015).
17. Sheldrick, G. M. Crystal structure refinement with SHELXL. *Acta Crystallogr. Sect. C Struct. Chem.* **71**, 3–8 (2015).
18. Kabuto, C., Akine, S., Nemoto, T. & Kwon, E. Release of software (Yadokari-XG 2009) for crystal structure analyses. *J. Cryst. Soc. Jpn.* **51**, 218–224 (2009).
19. Jiang, Z. & Sen, A. Tailored cationic palladium(II) compounds as catalysts for highly selective dimerization and polymerization of vinylic mechanistic aspects. *Organometallics* **12**, 1406–1415 (1993).
20. Aharonovich, S., Gjineci, N., Dekel, D. & Diesendruck, C. An effective eynthesis of *N*,*N*-diphenyl carbazolium salts. *Synlett* **29**, 1314–1318 (2018).
21. Staab, H. A., Rohr, W. & Graf, F. Darstellung von Diacylperoxyden und Persäureestern nach der Imidazolidmethode. *Chem. Ber.* **98**, 1122–1127 (1965).
22. Zhuo, J., Zhang, Y., Li, Z. & Li, C. Nickel-catalyzed direct acylation of aryl and alkyl bromides with acylimidazoles. *ACS Catal.* **10**, 3895–3903 (2020).
23 Nakajima, M., Miyamoto, K., Hirano, K. & Uchiyama, M. Diaryl-λ^3-chloranes: Versatile synthesis and unique reactivity as aryl cation equivalent. *J. Am. Chem. Soc.* **141**, 6499–6503 (2019).
